# Supplementary material for: Flexibility-tuning of dual-display DNA-encoded chemical libraries facilitates cyclic peptide ligand discovery
Source: Nat Commun. 2025 Apr 5;16:3273. doi: 10.1038/s41467-025-58507-w (PMC11972359; doi:10.1038/s41467-025-58507-w)
Supplement: Supplementary file 1 — Supplementary Information [file 41467_2025_58507_MOESM1_ESM.pdf]

Supplementary Materials for

Flexibility-tuning of dual-display DNA-encoded Chemical Libraries facilitates cyclic peptide ligand discovery

Dimitar Petrov[1], Louise Plais[1], Kristina Schira[1], Junyu Cai[1], Michelle Keller[1], Alice Lessing[1], Gabriele Bassi[2], Samuele Cazzamalli[2], Dario Neri[2], Andreas Gloger[1], Jörg Scheuermann[1]*

[1]Institute of Pharmaceutical Sciences, Department of Chemistry and Applied Biosciences, ETH Zurich; 8093 Zurich, Switzerland.
[2]Philochem AG, Libernstrasse 3, 8112 Otelfingen, Switzerland

Supplementary Information
DP-DEL

1. Abbreviations ... 4
2. General Materials and Methods ... 5
   2.1. Materials and Instruments ... 5
   2.2. General on-DNA/LNA Protocols ... 7
   2.3. General Solid-Phase/Solution-Phase Synthesis Procedures ... 8
3. Library Synthesis Preparation ... 10
   3.1. Scaffold Synthesis ... 10
   3.2. On-DNA Scaffold Deprotection Optimization ... 12
   3.3. Positive Control & Dipeptide Building Block Synthesis ... 16
   3.4. Amino Acid Coupling Screening ... 20
   3.5. Dipeptide Coupling Optimization ... 22
   3.6. Model Compound Synthesis ... 25
4. Library Synthesis ... 32
   4.1. Library Architecture ... 32
   4.2. Scaffold Coupling ... 33
   4.3. Step 1 Headpiece 5’ (HP5) Sublibrary ... 34
   4.4. Step 1 Headpiece 3’ (HP3) Sublibrary ... 39
   4.5. Step 2 Headpiece 5’ (HP5) Sublibrary ... 43
   4.6. Step 2 Headpiece 3’ (HP3) Sublibrary ... 48
   4.7. ESAC Library Creation & Derivatization ... 50
5. Affinity Selections ... 54
   5.1. Selection Procedure ... 54
   5.2. PCR Amplification of Library ... 54
   5.3. Proteins for Selections ... 57
      5.3.1. CAIX ... 57
      5.3.2. NSP14 ... 57
      5.3.3. Thrombin ... 58
      5.3.4. PLAP ... 58
   5.4. Sequencing Fingerprints Analysis ... 59
      5.4.1. Notes of Fingerprint Analysis ... 59
      5.4.2. Statistical Analysis ... 60
      5.4.3. Library Fingerprints ... 62

6. Hit Resynthesis 65
6.1. On-LNA Hit Resynthesis 65
6.1.1. Negative Control and Scaffold Attachment 66
6.1.2. PLAP 67
6.1.3. Thrombin 74
6.1.4. Streptavidin 78
6.2. Off-DNA Hit Resynthesis 81
6.2.1. Absorbance-based Concentration Determination of Fluorescein Labeled Binders 81
6.2.2. General Synthesis Procedure 82
6.2.3. Thrombin (Compounds 19-24) 82
6.2.4. PLAP (Compounds 29 & 30) 88
6.2.5. Streptavidin (Compounds 34-40) 92
6.2.6. Negative Control 100
7. Hit Validation 101
7.1. Fluorescence Polarization 101
7.2. Inhibition Assays 104
7.2.1. Thrombin 104
7.2.2. PLAP 104
8. Unprocessed Gel Images 106

1. Abbreviations

DCM: dichloromethane
DIPEA: N,N-Diisopropylethylamine
DMF: Dimethylformamide
DMSO: Dimethyl sulfoxide
DMT-MM: (4-(4,6-dimethoxy-1,3,5-triazin-2-yl)-4-methyl-morpholinium chloride)
EDC: 1-Ethyl-3-(3-dimethylaminopropyl)carbodiimide
EF: enrichment factor
Equiv.: equivalent
Fmoc: fluorenylmethoxycarbonyl protecting group
Boc: tert-butyloxycarbonyl protecting group
Alloc: N-Allyloxycarbonyl protecting group
PLAP: Placental Alkaline Phosphatase
NSP14: nonstructural protein 14
CAIX: carbonic anhydrase IX
CuAAC: copper-catalysed azide–alkyne cycloaddition
TBTA: Tris((1-benzyl-4-triazolyl)methyl)amine
HATU: Hexafluorophosphate Azabenzotriazole Tetramethyl Uronium
HOBt: Hydroxybenzotriazole
HOAt: 1-Hydroxy-7-azabenzotriazole
HPLC: High-performance liquid chromatography
MPLC: Medium Pressure Liquid Chromatography
IC_{50}: Half-maximal inhibitory concentration
K_D: dissociation constant
LC-MS: Liquid chromatography–mass spectrometry
UPLC-MS: Ultra-performance liquid chromatography–mass spectrometry
S-NHS: N-hydroxysulfosuccinimide
mQ: Milli-Q® Ultrapure Water
PBS: Phosphate-buffered saline
MOPS: 3-(N-morpholino)propanesulfonic acid
RP: reverse phase
RT: room temperature
TCEP: tris(2-carboxyethyl)phosphine
TEAA: Triethylammonium acetate
TEA: triethylamine
TFA: trifluoroacetic acid
Tris: tris(hydroxymethyl)aminomethane
LNA: locked nucleic acid
TIPS: Triisopropyl silane
EDTA: Ethylenediaminetetraacetic acid
NMM: N-Methylmorpholine

2. General Materials and Methods

2.1. Materials and Instruments

Material

DNA material was obtained from LGC Biosearch Technologies (Denmark) and Microsynth AG (Switzerland). DNA and fluoresceinated compound concentrations were measured using NanoDrop 2000c Spectrophotometer. Reagents and solvents were purchased from commercial suppliers (ABCR, Sigma Aldrich, Fluorochem, TCI Europe, Alfa Aesar, Enamine, Acros Organics). Water was purified using a Millipore Milli-Q system from Merck. All reaction vessels used for library creation were DNA LoBind® obtained from Eppendorf: 96 well plates (Katalog-Nr. 0030503201), tubes (Katalog-Nr. 0030108078), falcon tubes (Katalog-Nr. 0030122208). Enzymes and other materials related to ligation and polymerization of DNA were purchased from New England Biolabs. Gel purification kits were purchased from Qiagen. Gel images were captured with Bio-Rad Chemidoc. Tentagel® resin was purchased from Rapp Polymere (# S30132).

HPLC Purification of Oligonucleotides

Preparative RP-HPLC was performed on DNA and LNA conjugates using a Water Alliance RP-HPLC instrument with PDA-UV detector and an XBridge BEH C18 OBD Prep 130 Å, 5 µm 10x150 mm column. Oligos were eluted at 4 mL/min using a gradient of 100 mM TEAA (triethylamine-acetic acid) buffer pH 7 as buffer 1 and a mixture of 80% Acetonitrile, 10% mQ and 10% 1M pH 7 TEAA buffer as buffer 2.

Reverse Phase Purification of Small Molecules

RP-HPLC purification was done on an Alliance HT instrument from Waters with a Synergi 4 µm Polar-RP 80 Å 10x150 mm column with a flow rate of 4 mL/min. RP-MPLC purification of dipeptides and scaffold was performed on a Büchi system equipped with a FlashPure C18 40 µm irregular column (40g) with a flow rate of 40 mL/min using settings as recommended by manufacturer. Elution was performed using a gradient of 0.1% Formic Acid in mQ as buffer 1 and 0.1% Formic Acid in Acetonitrile as buffer 2. Purified elution fractions were dried in a Christ freeze drier.

UPLC-MS Analysis of Oligonucleotides/Small Molecules

Oligonucleotide traces were recorded using a Xevo G2-XS Quadrupole Time of Flight (Qtof) Mass Spectrometer LC-MS from Waters on a Xbridge Oligonucleotide BEH C18 10 x 50 mm column at 60 C°. Elution was performed at a flow rate of 0.5 mL/min using 5 mM triethylamine and 400 mM hexafluoroisopropanol as buffer 1 and methanol as buffer 2. Elution was performed as a 0-50% gradient of buffer 2 over 7 minutes (termed "polar" method) or 0-70% (termed "nonpolar" method), in order to better elute conjugates of different hydrophobicity. Most oligonucleotidies were analyzed using the polar method, unless otherwise stated.

Small molecule traces were recorded on the same instrument on a BEH C18 2.1 x 50 mm column at 40 C° at a flow rate of 0.6 mL/min using a gradient 0-100% of 0.1% formic acid in mQ as buffer 1 and 0.1% formic acid in Acetonitrile as buffer 2. Samples were tested by

taking a small aliquot of resin, which was cleaved with 95% TFA/ 2.5% TIPS/2.5% water mixture. Obtained mixture was diluted with 50 µL methanol, filtered and measured in positive ion mode.

Agarose Gel Electrophoresis
Usual Conditions: Oligonucleotide size and hybridization was analyzed with gel electrophoresis. DNA was run on 3% agarose gel casted using TBE buffer (89 mM Tris, 89 mM boric acid, 0.4 mM EDTA) and stained with GelRed® (biotium). Gel was run for 40 min at 90 V and 200 mA using TBE running buffer. 6x Tritrack loading dye was used (Thermofisher) for DNA loading. These conditions were used in gel experiments unless otherwise noted.

Denaturing condition: 3% agarose gel was cast in 30 mM NaCl, 2mM EDTA pH 7.5 buffer, without addition of GelRed®. Gel immersed in running buffer (30 mM NaOH, 2 mM EDTA) for 15-30 min. Meanwhile 6x denaturing loading dye (180 mM NaOH, 6 mM EDTA, 18% Ficoll 400, 0.05% bromocresol green) was added to the test DNA. The DNA was then heated to 90 °C and run on the gel electrophoresis at 300 mA and 90V for 1 hour (90V was not reached due to running buffer, but this was the setting used). The gel was then stained for 20-30 min using GelRed® and analyzed. Conditions were adapted from Thermo Scientific [1]

Speed Vacuum
DNA samples and other water-containing samples were concentrated and dried using a Christ RVC 2-25 rotational-vacuum-concentrator.

Solid-phase Synthesis of Conjugates
Heidolph Reax 2 and innova 4000 incubator shaker was used for shaking/stirring solid-phase reactions in fritted syringes. Vac-Man® Laboratory Vacuum Manifold used for rinsing beads between reactions.

Binding & Inhibition Experiments
For fluorescence polarization and thrombin inhibition assays, 384-well non-binding black plates were used (Greiner Bio-one, 784900). Grenier 384 transparent, flat-bottom well plates were used for PLAP inhibition assays. Measurements were taken on a Spectra Max Paradigm multimode plate reader.

Nuclear Magnetic Resonance (NMR) Analysis
1H and 13C NMR spectra were recorded on a Bruker AV400 (400 MHz and 100 MHz respectively) instrument. Shifts are recorded in parts per million (ppm) and standardized according to residual employed NMR solvent. Coupling constants are reported in hertz (Hz) and the following abbreviations were employed for peak splitting: s = singlet, d = doublet, t = triplet, q = quartet, m = multiplet.

Software
Selection Fingerprint data was displayed and evaluated using MATLAB version R2023b (Mathworks). Selection triplicate count data was concatenated using a Python script. Fluorescence polarization and Inhibition Assay data was fitted and displayed using PRISM 10. NMR was evaluated using Mestrenova 14.2 software

2.2. General on-DNA/LNA Protocols

DNA Precipitation Protocol:

Dissolved DNA was precipitated via addition of 0.1x volume 5M NaCl, followed by the addition of 3 volumes of ethanol. The mixture was then left in a -20 °C freezer overnight. The resulting suspension was centrifuged (25 000 x g, 4 °C) for 30 minutes. The supernatant was discarded, the pellet dried using speed vacuum and recovered as precipitated DNA.

Scaffold Attachment Procedure

Up to 1 µmol of DNA was dissolved in 800 µL MOPS buffer (50 mM, 0.5 M NaCl, pH 8). In a separate vial a scaffold activation solution was prepared: 320 µL scaffold solution (100 mM, in DMSO) was mixed with 160 µL S-NHS (100mM, in 2:1 DMSO/mQ), 160 µL EDC (100 mM, in DMSO) and 720 µL DMSO. The activation solution was reacted for 30 min at 30 °C and then added to the DNA solution. The reaction was carried out overnight at RT and precipitated. The volumes were scaled accordingly when less DNA starting material was used.

General Amidation Procedure (DMT-MM)

Up to 60 nmol of DNA was dissolved in 242 µL of borate buffer (85 mM, pH 9.3). To this solution 135 µL of acetonitrile was added, followed by 45 µL of carboxylic acid solution (200 mM, in DMSO). 28 µL of DMT-MM (300 mM, in mQ) was finally added and the reaction was shaken at room temperature for 2 hours. 28 µL of additional DMT-MM solution were then added and the reaction was shaken for further 2 hours and precipitated. Protocol was adapted from Monty et al.[2]

Optimized Dipeptide Building Block Coupling Procedure (EDC/ HOAt)

Up to 60 nmol of DNA was dissolved in 100 µL MOPS buffer (200 mM, 3 M NaCl, pH 8). In a separate vial 60 µL of 200 mM carboxylic acid in DMSO was mixed with 65 µL of EDC/HOAt/NMM (100:20:100 mM) in DMSO and 170 µL DMSO. Consequently, the DMSO solution was immediately added to the DNA solution. The reaction was shaken for 1 hour and then a second batch of DMSO solution (60 µL carboxylic acid and 60 µL EDC/HOAt/NMM) was added to the reaction, which was further shaken overnight and precipitated.

Diazotransfer Procedure

Up to 100 nmol of DNA was dissolved in 20 µL mQ and added to a premixed solution of 20 µL $CuSO_4$ (10 mM, in mQ), 50 µL Imidazole-1-sulfonyl Azide.HCl (200 mM, in mQ) and 200 µL 50 mM K_2CO_3. The reaction was allowed to mix overnight at RT. The reaction was analyzed by LC-MS and precipitated.

Interstrand Click Reaction

To a pre-hybridized solution of up to 10 nmol of HP5 and HP3 strand were added 22 µL of TEAA buffer (1M, pH 7), 32 µL DMSO, 25 µL $CuSO_4$/TBTA complex (1 mM, in 55% DMSO) and 25 µL sodium ascorbate (10 mM, in mQ). The reaction was carried out overnight at RT and then precipitated with NaCl. Protocol was adapted from Puglioli et al. [3]

Scaffold Deprotection & Disulfide Formation.
1 nmol of HPLC-purified insterstrand clicked library was dissolved in 10 µL buffer consisting of sodium acetate (30 mM), ascorbic acid (10 mM), semicarbazide hydrochloride (25 mM) pH 4.75. It was then placed under an UV lamp at 365 nm for 10-15 min at RT.

Thioether Formation
Scaffold deprotected DNA was dissolved in 35 µL NH_4HCO_3 buffer (60 mM, pH 8), followed by 5.6 µL TCEP solution (10 mM, pH 7) and shaken for 1 hour at RT. 11.2 µL of bis-electrophile (10 mM) in acetonitrile was then added, 2.8 µL acetonitrile and 15.4 µL NH_4HCO_3 buffer. Reaction was mixed for 2 hours at 30 °C and precipitated. Protocol was adapted from Pham et al. [4]

Fmoc Deprotection Procedure
DNA was dissolved in 10% piperidine in mQ and the deprotection was carried out over 1 hour. The reaction was then quenched using 1.5x volume of 3M sodium acetate pH 4.5 buffer to neutralize the piperidine and the DNA precipitated via addition of ethanol. Alternatively, the deprotection mixture was dried using a speed vacuum, redissolved in water and precipitated with NaCl.

Encoding/Ligation Procedure
1-50 nmol of DNA starting material was dissolved in mQ. 1.5 equivalents of coding strand and 2 equivalents of adaptor strand were added to make a total volume of 180 µL. The solution was heated to 80 °C and cooled to RT over 1 hour. 20 µL of T4 DNA ligase buffer (10x, NEB) and 400U T4 DNA ligase (NEB) were then added and the ligation was incubated at 16 °C overnight.

Terminal Primer Installation/ Polymerization of Large encoding design

Protocol was followed based on Plais et al. [5]. Briefly 1 nmol of premixed HP5 + HP3 step 2 library pools in any configuration (open, clicked, etc.) was mixed with 2.5 nmol junction primer and 2.5 nmol terminal primer in a total of 100 µL mQ. The strands were annealed by raising temperature to 95 °C for 10 min and cooled to room temperature. 50 µL of T4 DNA ligase buffer (10x, NEB), DNA polymerase (10U, NEB) and dNTPs (250 nmol) were added and mixture was kept at 16 °C for 30 min. T4 ligase was then added (400U, NEB) and ATP (300 nmol) and kept at 16 °C for a further 1 hour.

2.3. General Solid-Phase/Solution-Phase Synthesis Procedures

General solid-phase synthesis procedure
Commercially available Tentagel® S NH2 base resin (Rapp polymere, # S30132) (500-1000 mg, 0.29 mmol/g loading) was swollen in DMF inside of a 10-25 mL fritted syringe. The resin was then manually modified with Fmoc-Rink Amide linker using general procedures for amide coupling and Fmoc deprotection. The resin was then used for hit resynthesis.

Amide coupling
DMF was added to free amino modified resin, followed by 2 equivalents of free carboxylic acid building block, 2 equivalents of HATU and 4 equivalents of DIPEA. The reaction was shaken for 2-12 hours and then quenched by removing the solution and washing the resin 5 times with DMF.

Fmoc deprotection
Resin was shaken for 1 hour with 20% Piperidine in DMF. The piperidine/DMF solution was then removed and the resin was washed 5 times with DMF.

Alloc deprotection
Resin was swollen in DCM. Alloc group at Lysine residue was treated with 0.2 equiv. $Pd(PPh_3)_4$ and 10 equiv. $PhSiH_3$ in dry DCM for 2-3 hours. The fritted syringe reaction vessel was vented every 10-15 min during the reaction. The resin was then washed 3x with DCM and 5x with DMF.

Resin cleavage and purification
The resin was shaken for one hour with a mixture of 95/2.5/2.5 % trifluoroacetic acid/water/triisopropylsilane. The cleavage solution was then removed from the resin and stored. The procedure was repeated one additional time with fresh cleavage solution. The cleaved material was then either precipitated or diluted with MeOH and directly purified via RP-HPLC.

Peptide precipitation
5-10 volumes of diethyl ether were added to the peptide cleavage solution. The resulting mixture was kept in a -20 ^{o}C freezer for 1-12h and centrifuged. The crude peptide product could then be isolated as a pellet and dried.

General procedure of CuAAC
The resin was treated with 3 equiv. of CuI, 21 equiv. L-ascorbic and 1.2 equiv. of alkyne in 20% piperidine/DMF for 20 h. The resin was then washed 5x with DMF.

General procedure of azido-transfer.
The resin was swollen in DMSO. It was then treated with 3 equiv. of 1H-imidazole1-sulfonyl azide hydrochloride and 9 equiv. DIPEA in dry DMSO overnight. The resin was then washed 3x with DMSO and 5x with DMF.

Stbu deprotection and cyclization via thioether formation
1-2 mM hit intermediate possessing 2 Cys(Stbu) amino acids in 50 µL DMSO was added to 350 µL DMF, 150 µL 1M NH_4CO_3 pH8 and 50µL of 160 mM TCEP in 1M NH_4CO_3 pH8. The deprotection was carried out over 1h and checked via LC-MS for completion. Upon completion 50 µL of 160 mM bis-electrophile in DMF was added. Reaction was then incubated further for 30 min and checked for completion via LC-MS. Reaction solution was then diluted with MeOH and directly purified via RP-HPLC

3. Library Synthesis Preparation

In the process of optimizing various areas of library synthesis, an amino linked 14-mer DNA strand (5'-C6-AminolinkGGAGCTTCTGAATT-3' Mass 4474 Da) was used for test on-DNA reactions. This DNA is referred to as "**test DNA**" in further parts of the supplementary.

Test model compounds were synthesized on two complementary amino linked DNA strands, which were also later used as headpiece strands in actual library synthesis.

HP5 strand (5'NH2-C6-GGAGCTTCTGAATTCTGTGTGCTG[dSpacer][dSpacer][dSpacer][dSpacer]CTGGTCACTC 3') Mass 11346 Da
HP3 strand (5' Phos-AGTCACCTCA[dSpacer][dSpacer][dSpacer][dSpacer]CAGCACACAGAATTCA GAAGCTCC-C6-NH2 3') Mass 11313 Da

3.1. Scaffold Synthesis

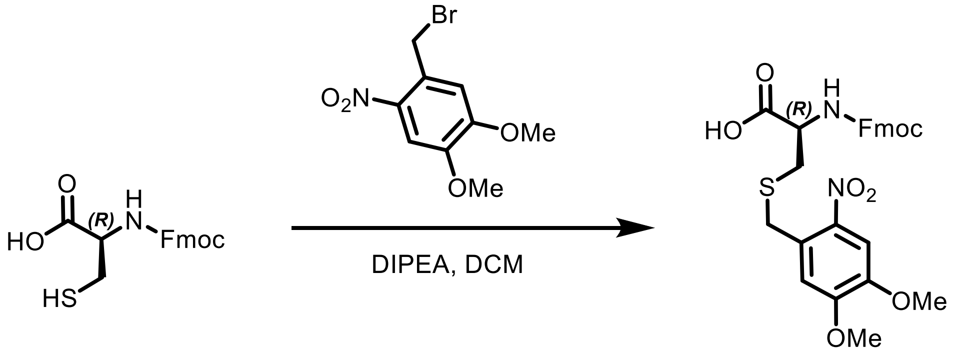

N-(((9H-fluoren-9-yl)methoxy)carbonyl)-S-(4,5-dimethoxy-2-nitrobenzyl)cysteine was synthesized as described in Karas et al. [6]. Briefly, 4,5-Dimethoxy-2-nitrobenzyl bromide (80 mg, 0.29 mmol) was dissolved in 5 mL DCM. 5 equivalents of DIPEA (250 µL, 1.45 mmol) were then slowly added, followed by a portion-wise addition of Fmoc-Cys-OH (100 mg, 0.29 mmol). The reaction was covered in aluminum foil to protect from light and left to react at rt overnight. The reaction solution was then reduced, redissolved in Acetonitrile/mQ and directly purified with RP-MPLC. A yellow crystalline powder was obtained after drying the eluted pure product (yields 40-60%). Reaction was scaled successfully with up to 3 times more starting material.

^{1}H NMR (400 MHz, DMSO) δ 7.89 (d, *J* = 7.5 Hz, 2H), 7.73 (dd, *J* = 8.0, 6.5 Hz, 2H), 7.65 (s, 1H), 7.41 (td, *J* = 7.5, 1.2 Hz, 2H), 7.32 (td, *J* = 7.4, 1.1 Hz, 2H), 7.15 (s, 1H), 6.97 (d, *J* = 8.5 Hz, 0.5H), 6.70 (d, *J* = 8.5 Hz, 0.5H), 4.35 – 4.02 (m, 6H), 3.87 (s, 3H), 3.83 (s, 3H), 2.87 (dd, *J* = 13.9, 4.5 Hz, 1H), 2.71 (dd, *J* = 13.9, 9.7 Hz, 1H).
^{13}C NMR (101 MHz, DMSO) δ 172.27, 156.03, 152.57, 147.43, 143.79, 140.71, 139.78, 130.45, 128.95, 127.64, 127.07, 125.27, 120.11, 115.17, 114.21, 108.86, 65.74, 56.16, 55.98, 53.89, 48.97, 46.60, 33.19, 32.47, 29.11.

ESI-MS: [M+H]+ 538.14 g/mol, found 539.56

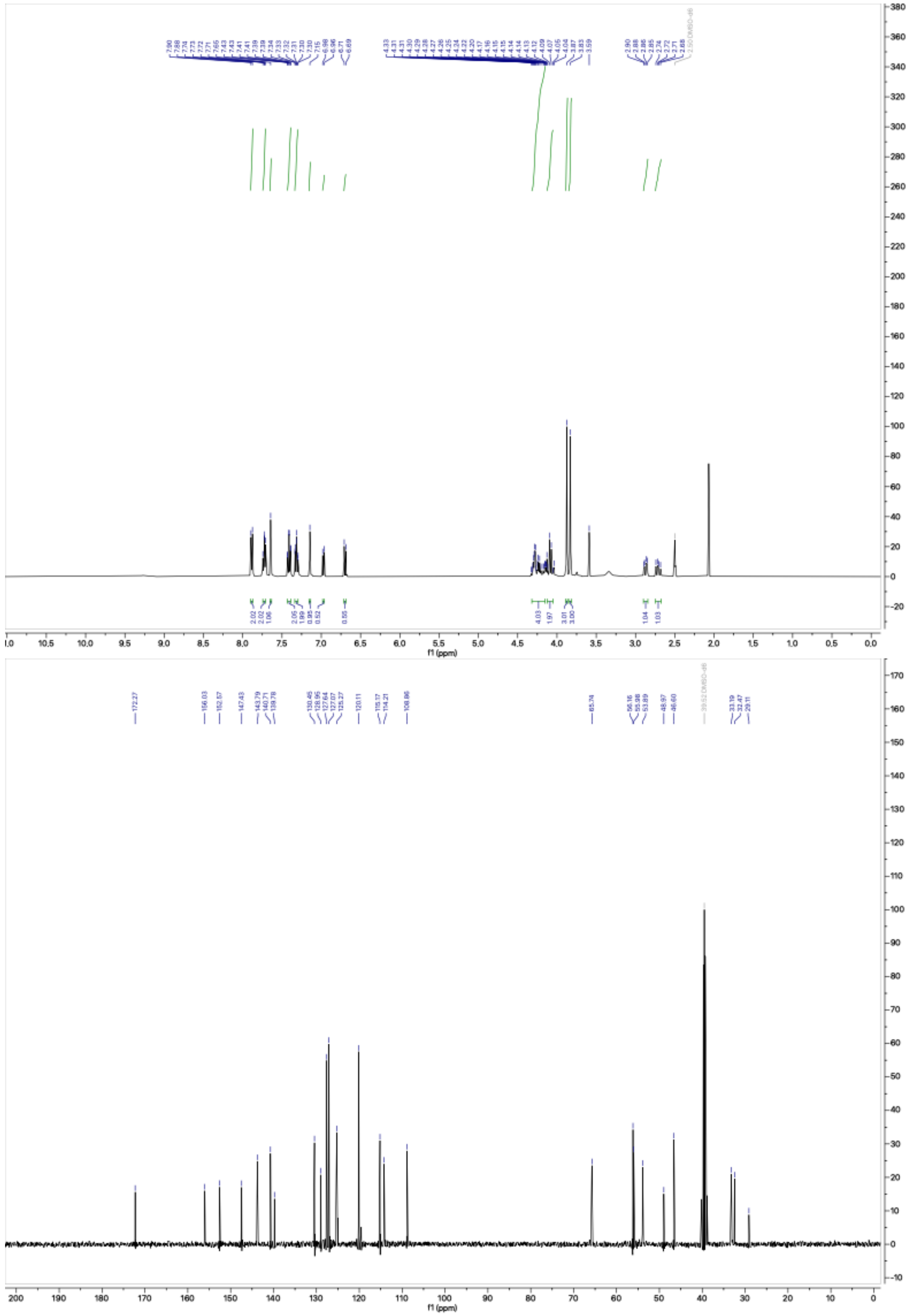

Figure S1: ^{1}H NMR and ^{13}C NMR analysis of protected cysteine scaffold

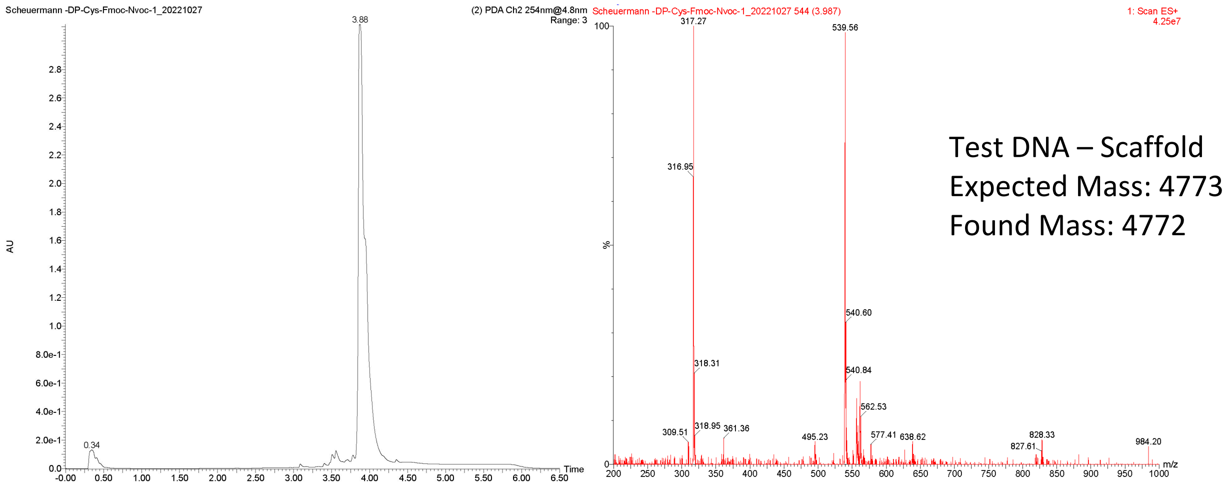

Figure S2: LC-MS chromatogram (left) and the corresponding deconvoluted MS spectra (right) of protected cysteine scaffold

3.2. On-DNA Scaffold Deprotection Optimization

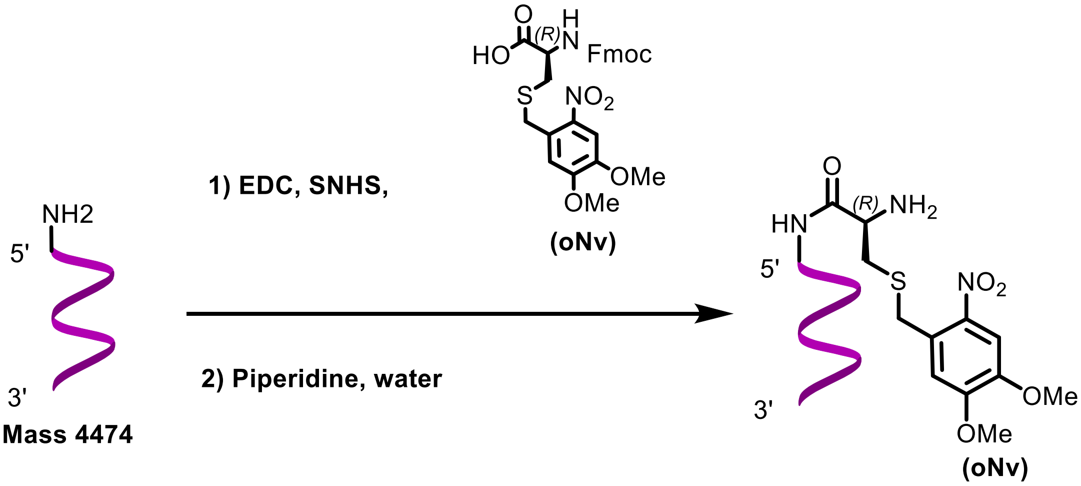

Scaffold was attached to “test DNA” according to general attachment procedure. The DNA was then precipitated and the Fmoc group was removed using general procedures. The product was HPLC purified and used in further experiments.

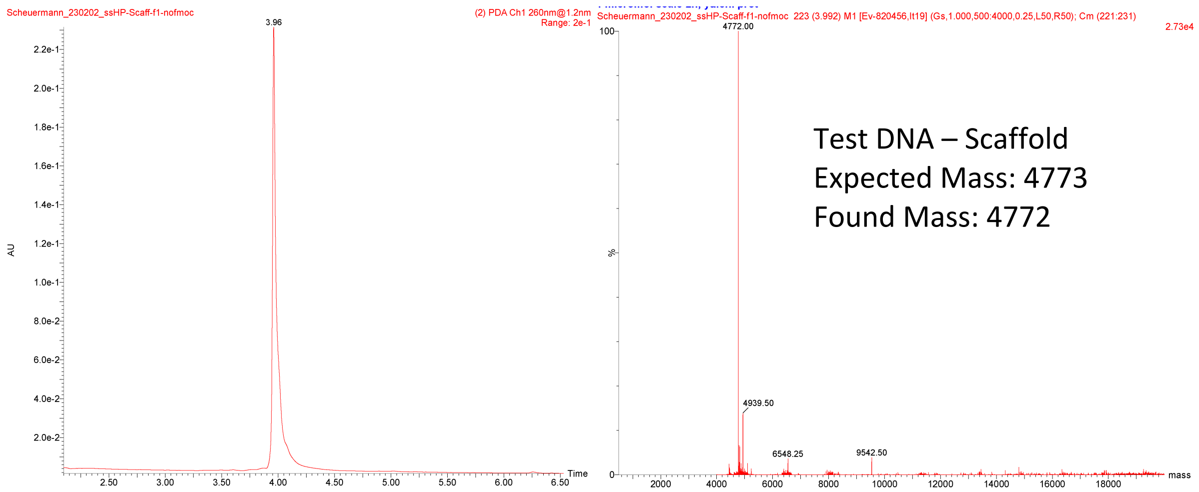

Figure S3: LC-MS chromatogram (left) and the corresponding deconvoluted MS spectra (right) of cysteine scaffold-test DNA conjugate

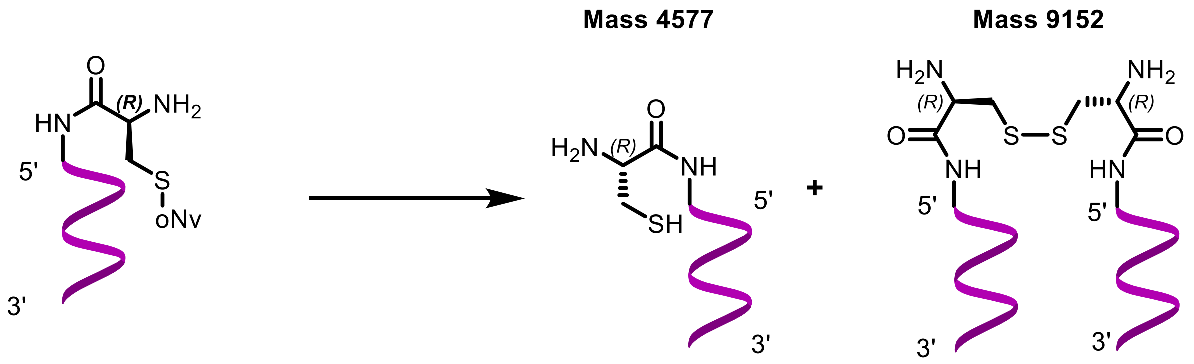

0.5 nmol of scaffold-linked “test DNA” was dissolved in 10-20 µL of buffer and the cysteine scaffold was deprotected using an UV lamp at 365 nm using conditions listed in table S1 and Figures S4-6.

Table S1: Optimization of on-DNA UV deprotection of cysteine 2-nitroveratryl protecting group

Condition	Buffer	Temp. (C)	Time (min)	% Starting Material	% Product Disulfide	% Product reduced
**1**	50 mM Phosphate Buffer pH 7	4	120	47.34	13.68	1.29
**2**	50 mM Phosphate Buffer pH 7	26	15	35.16	19.38	2.64
**3**	85 mM borate buffer pH 9.3	26	15	45.44	22.14	1.1
**4**	30 mM NaOAc buffer pH 4.75	26	15	0	35.20	12.49
**5**	30 mM NaOAc buffer + 10 mM ascorbic acid pH 4.75	26	15	0	46.74	22.67
**6**	15 mM NaOAc buffer + 5 mM ascorbic acid + 12.5	26	15	0	61.13	6.51

	mM semicarbazide pH 4.75					

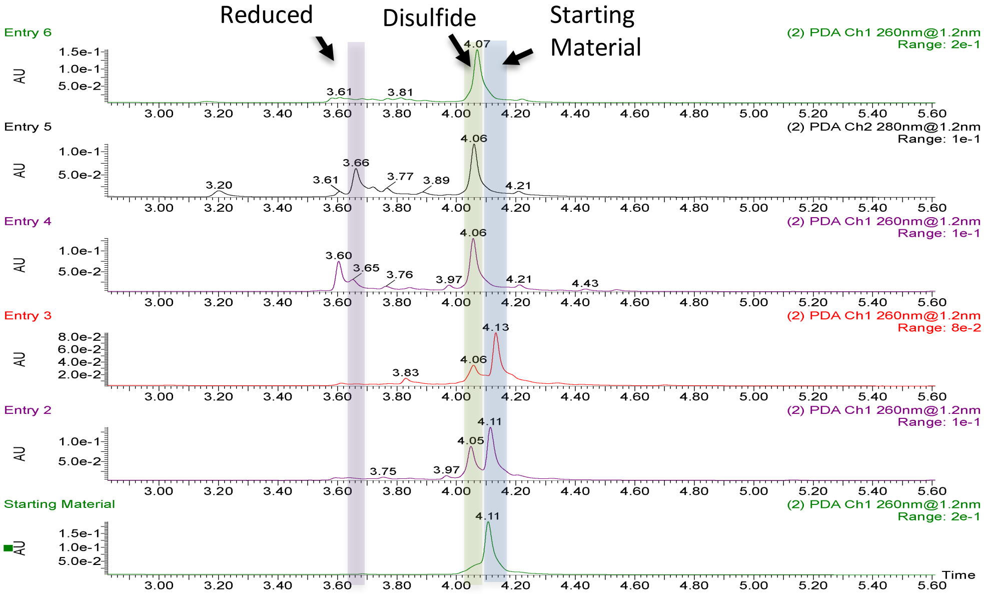

Figure S4: LC-MS chromatogram (left) and the corresponding deconvoluted MS spectra (right) of various Scaffold deprotection conditions

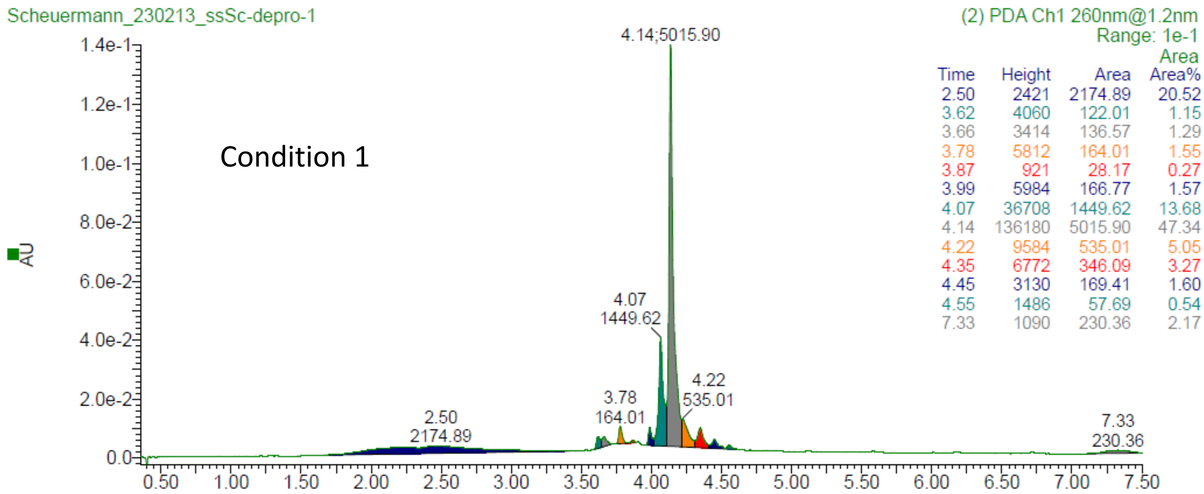

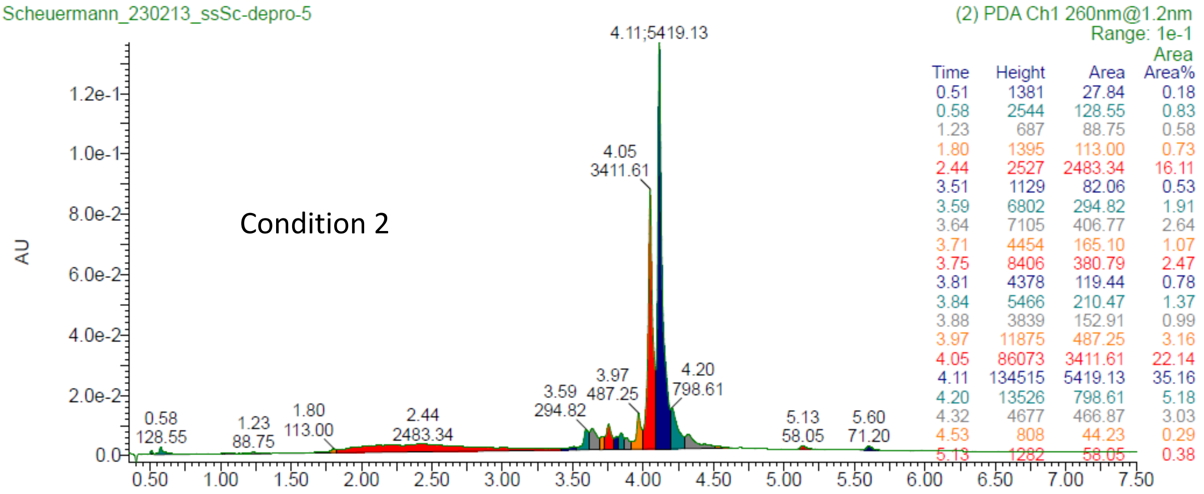

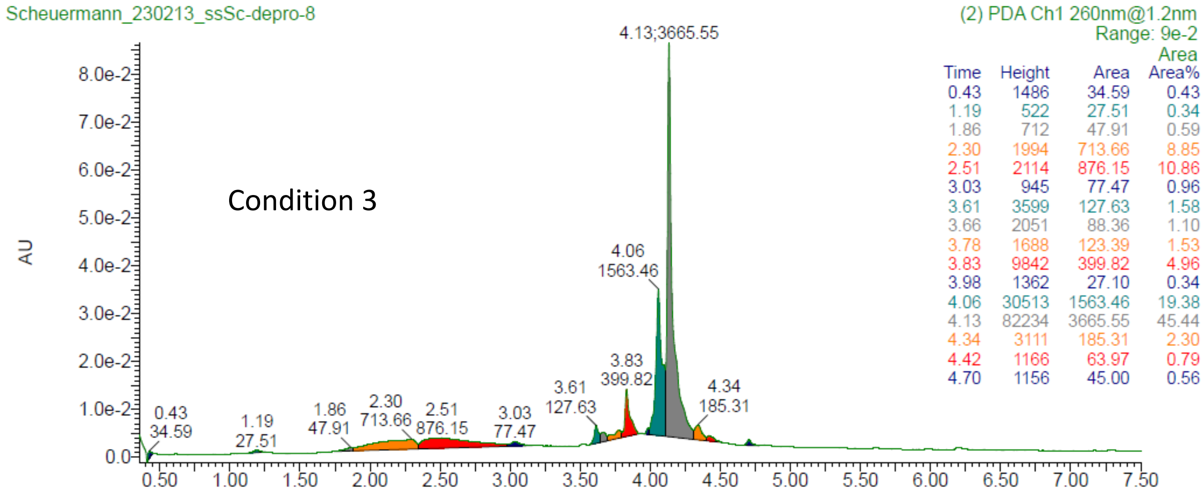
Scheuermann_230213_ssSc-depro-8
(2) PDA Ch1 260nm@1.2nm
Range: 9e-2
Condition 3
AU
4.13;3665.55
Time Height Area Area%
0.43 1486 34.59 0.43
1.19 522 27.51 0.34
1.86 712 47.91 0.59
2.30 1994 713.66 8.85
2.51 2114 876.15 10.86
3.03 945 77.47 0.96
3.61 3599 127.63 1.58
3.66 2051 88.36 1.10
3.78 1688 123.39 1.53
3.83 9842 399.82 4.96
3.98 1362 27.10 0.34
4.06 30513 1563.46 19.38
4.13 82234 3665.55 45.44
4.34 3111 185.31 2.30
4.42 1166 63.97 0.79
4.70 1156 45.00 0.56
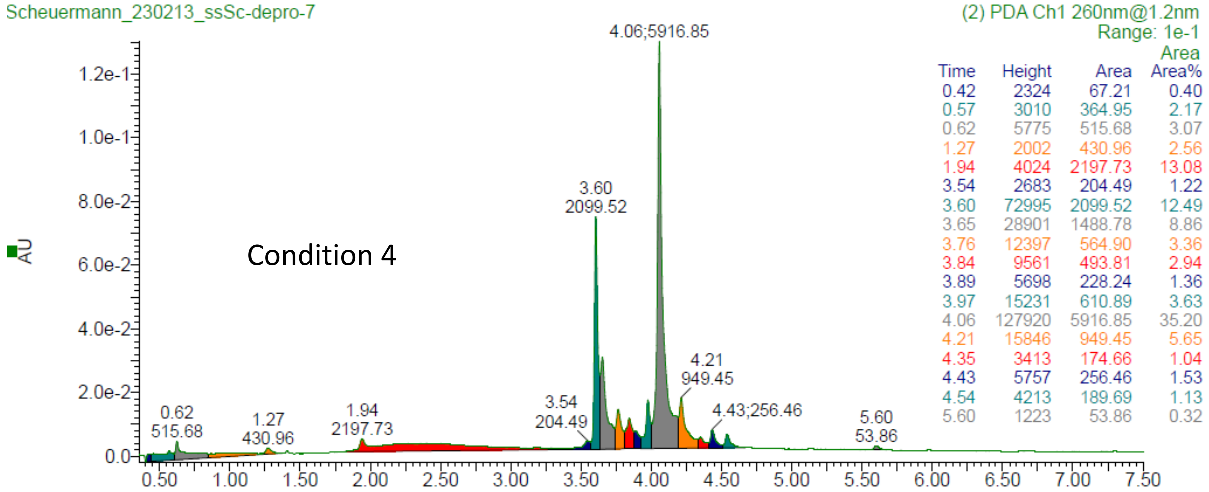
Scheuermann_230213_ssSc-depro-7
(2) PDA Ch1 260nm@1.2nm
Range: 1e-1
Condition 4
AU
4.06;5916.85
Time Height Area Area%
0.42 2324 67.21 0.40
0.57 3010 364.95 2.17
0.62 5775 515.68 3.07
1.27 2002 430.96 2.56
1.94 4024 2197.73 13.08
3.54 2683 204.49 1.22
3.60 72995 2099.52 12.49
3.65 28901 1488.78 8.86
3.76 12397 564.90 3.36
3.84 9561 493.81 2.94
3.89 5698 228.24 1.36
3.97 15231 610.89 3.63
4.06 127920 5916.85 35.20
4.21 15846 949.45 5.65
4.35 3413 174.66 1.04
4.43 5757 256.46 1.53
4.54 4213 189.69 1.13
5.60 1223 53.86 0.32
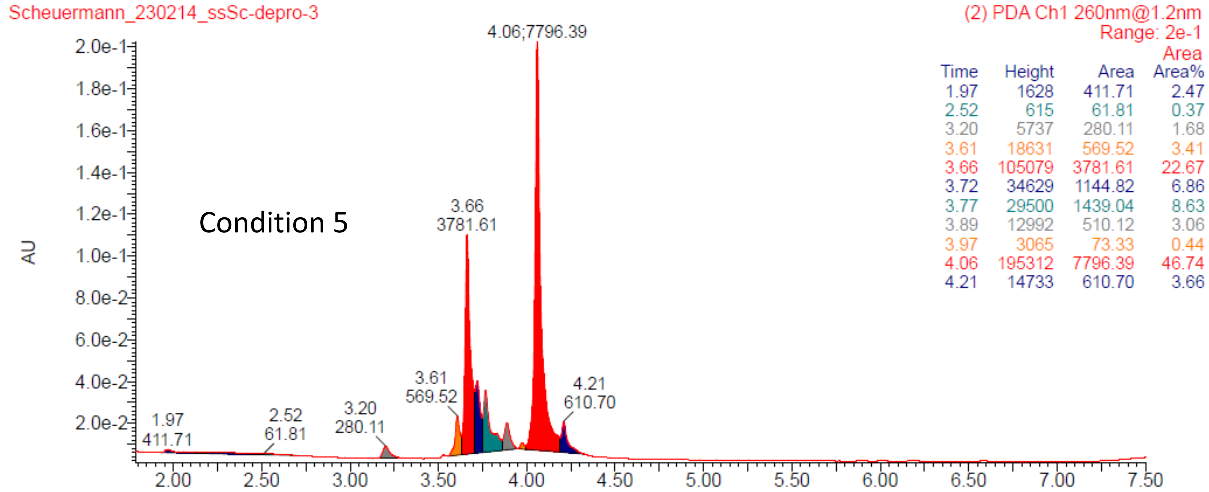
Scheuermann_230214_ssSc-depro-3
(2) PDA Ch1 260nm@1.2nm
Range: 2e-1
Condition 5
AU
4.06;7796.39
Time Height Area Area%
1.97 1628 411.71 2.47
2.52 615 61.81 0.37
3.20 5737 280.11 1.68
3.61 18631 569.52 3.41
3.66 105079 3781.61 22.67
3.72 34629 1144.82 6.86
3.77 29500 1439.04 8.63
3.89 12992 510.12 3.06
3.97 3065 73.33 0.44
4.06 195312 7796.39 46.74
4.21 14733 610.70 3.66

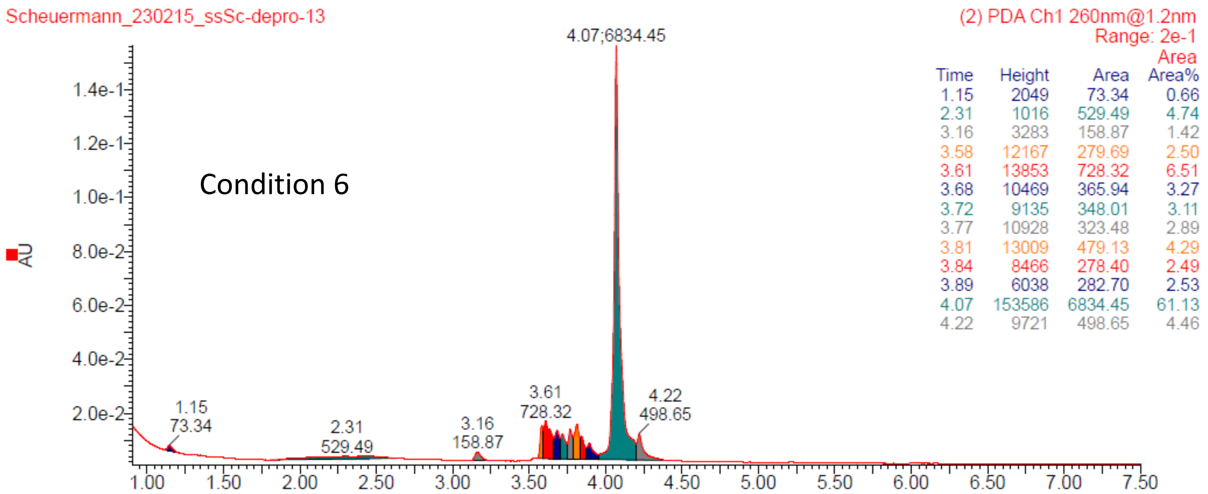

Figure S5: UV 260 nm chromatogram of oNv deprotection of test DNA according to conditions in table S1. Peaks are integrated and used for analysis

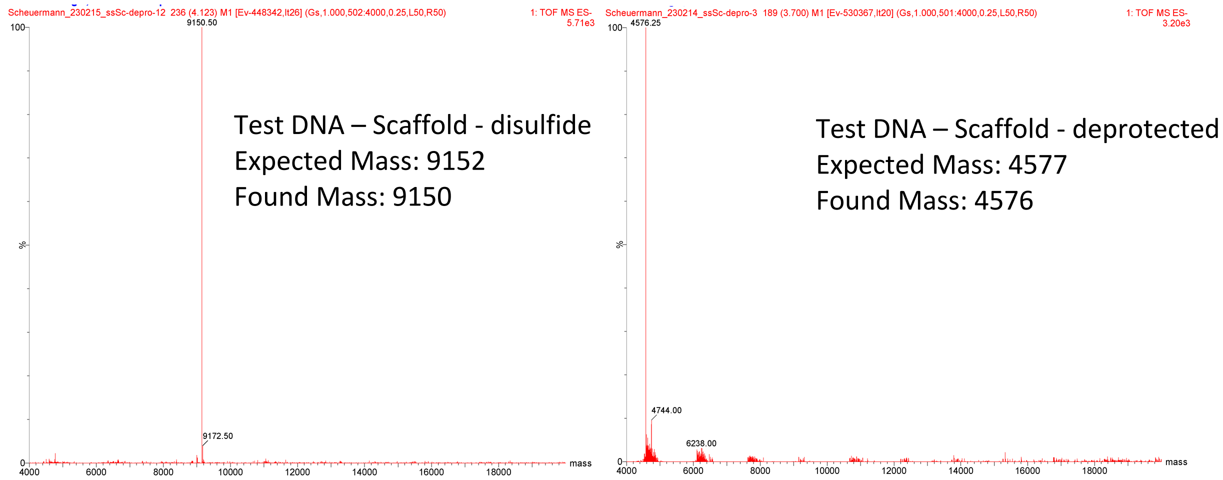

Figure S6: Deconvoluted MS of deprotected disulfide cysteine product and reduced cysteine product

3.3. Positive Control & Dipeptide Building Block Synthesis

A summary of all MS-based characterization of synthesized positive controls and dipeptides is given in table S2 and … .

Positive Controls:

N2-(((9H-fluoren-9-yl)methoxy)carbonyl)-N6-(4-sulfamoylbenzoyl)-L-lysine synthesis and characterization via NMR and LC-MS was described in the supplementary of Keller et al. [7]. The same product was used in DP-DEL.

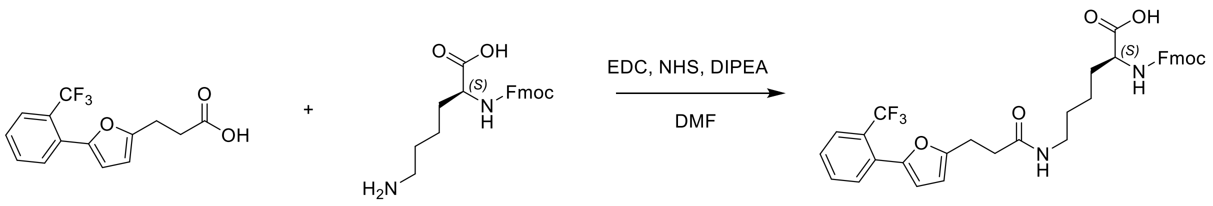

N2-(((9H-fluoren-9-yl)methoxy)carbonyl)-N6-(3-(5-(2-(trifluoromethyl)phenyl)furan-2-yl)propanoyl)-L-lysine was synthesized as listed above. Briefly 3-(5-(2-(trifluoromethyl)phenyl)furan-2-yl)propanoic acid (213 mg, 0.75 mmol) along with 1-hydroxypyrrolidine-2,5-dione (166 mg, 1.45 mmol) were dissolved in 10 mL DMF. EDC.HCl (222 mg, 1.16 mmol) was added and the mixture was stirred for 30-60 min. The resultant activated ester was added along with DIPEA (262 µL, 1.5 mmol) to Fmoc-L-Lys-OH (184 mg, 0.5 mmol) and stirred overnight. The reaction was then concentrated, redissolved in ethyl acetate and washed with 1M HCl, water and brine. The organic layer was dried and purified via RP-MPLC to generate a brownish powder (43 mg, 13% yield).

ESI-MS: [M+H]+ 634.22 g/mol, found 635.42

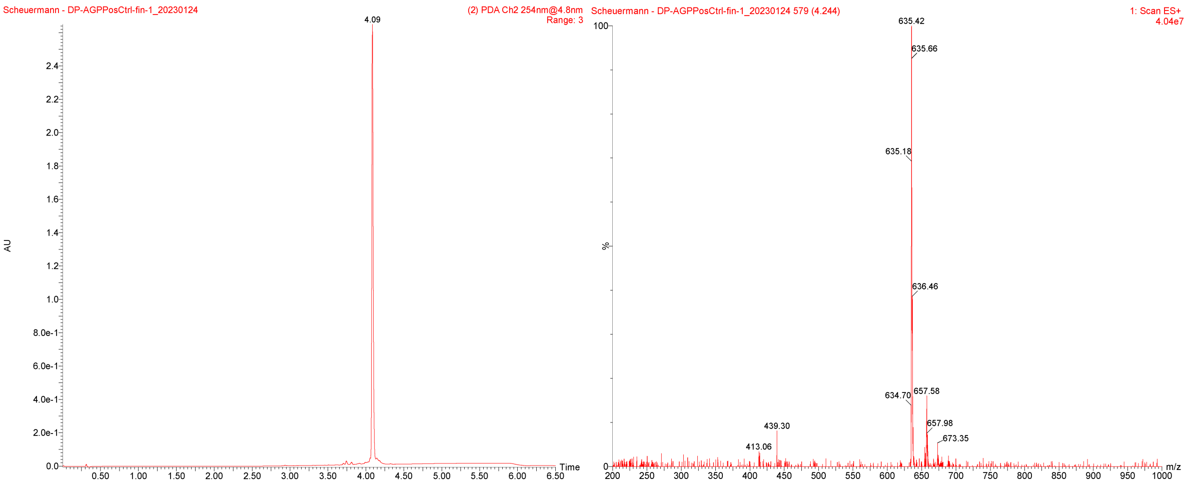

Figure S7: LC-MS chromatogram (left) and the corresponding deconvoluted MS spectra (right) of N2-(((9H-fluoren-9-yl)methoxy)carbonyl)-N6-(4-sulfamoylbenzoyl)-L-lysine

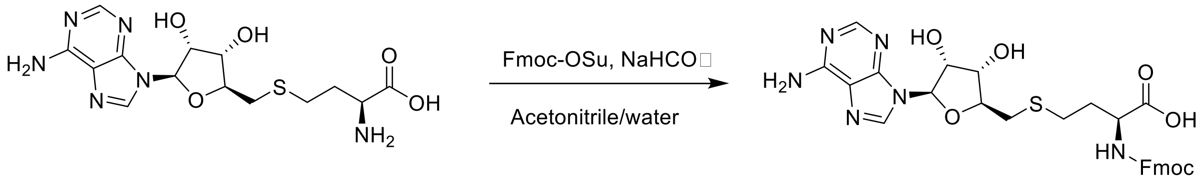

N-(((9H-fluoren-9-yl)methoxy)carbonyl)-S-(((2S,3S,4R,5R)-5-(6-amino-9H-purin-9-yl)-3,4-dihydroxytetrahydrofuran-2-yl)methyl)-L-homocysteine (40 mg,) was dissolved in 2.5 mL water in presence of 1 equiv. sodium bicarbonate. A solution of Fmoc-Osu (0.9 equiv) in 2.5 mL acetonitrile was added. Product formation was confirmed via LC-MS. Product was isolated via extraction with ethyl acetate, washing with water, brine and the organic layer was dried.

ESI-MS: [M+H]+ 606.18 g/mol, found 607.57

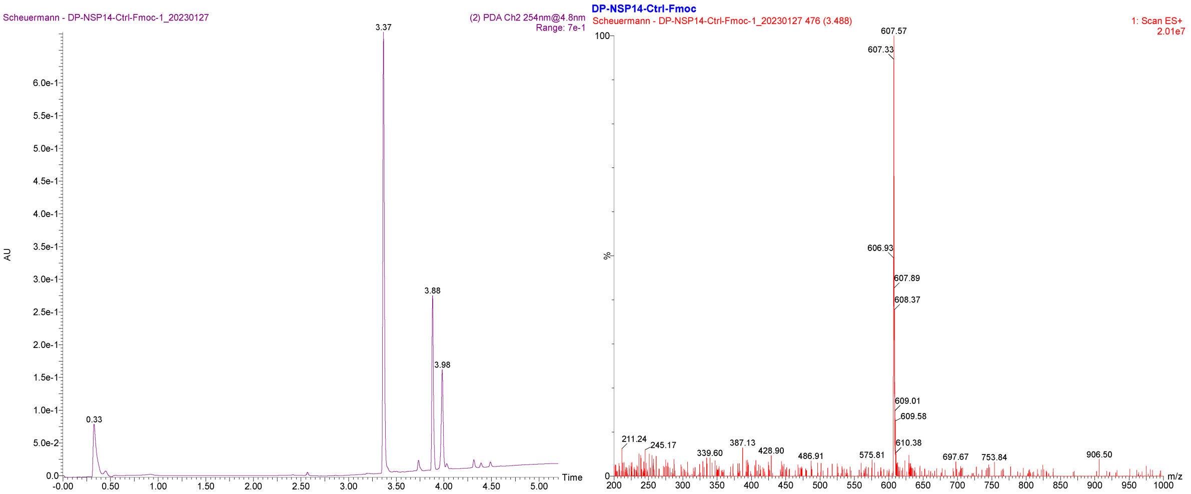

Figure S8: LC-MS chromatogram (left) and the corresponding deconvoluted MS spectra (right) of N-(((9H-fluoren-9-yl)methoxy)carbonyl)-S-(((2S,3S,4R,5R)-5-(6-amino-9H-purin-9-yl)-3,4-dihydroxytetrahydrofuran-2-yl)methyl)-L-homocysteine

Dipeptides:

A total of 75 Fmoc-N-protected dipeptides were produced manually in solution consisting of natural and artificial amino acids, to be used as building blocks in DP-DEL. Production of dipeptides consisted of an amidation of an N-Fmoc protected amino acid with a C-protected amino acid. The C terminus of the resulting crude dipeptide was then deprotected.

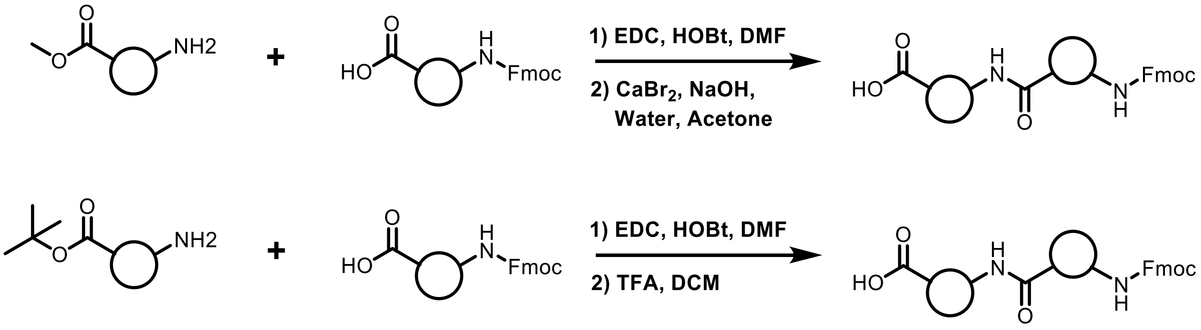

N-Fmoc protected amino acid (1-1.5 mmol) was dissolved in 15 mL DMF. 1 equivalent C-protected amino acid as free base or HCl salt, 1.1 equivalents of DIPEA, 1 equivalent of EDC.HCl and 1.1 equivalents of HOBt were consequently added to the solution. The amidation was performed overnight at rt and checked for completion via LC-MS. The reaction mixture was then dried, redissolved in ethyl acetate and washed with 1M HCl, 5% LiCl and brine. The organic layer was then reconcentrated and used as is for the next step.

If the C-protected dipeptide product was protected with a tBu group, the step 1 crude was dissolved in 1:1 TFA/DCM solution and deprotected over 2 hours and then concentrated. It was then redissolved in 1:1 acetronitrile/water and purified via RP-MPLC.

If the C-protected dipeptide produced was protected with a methyl ester, deprotection was carried as described by Binette et al. [8]. Briefly, the step 1 crude was redissolved via the

addition of 6 mL 5M $CaBr_2$, 750 µL 2M NaOH and 16 mL acetone. The deprotection was carried out overnight and checked for completion via LC-MS. The finished reaction was quenched via the addition of 1M HCl, extracted into ethyl acetate and concentrated. The resulting crude was redissolved in 1:1 acetonitrile/water and purified via RP-MPLC.

Table S2: List of synthesized dipeptides and positive controls and their MS characterization.

#	Dipeptide (N-Fmoc Protected)	Expected Mass	Found Mass
1	L-phenylalanylglycine	444.16	445.06
2	(*S*)-(2-amino-3-(thiazol-4-yl)propanoyl)glycine	451.11	452.18
3	L-glutaminylglycine	425.15	426.26
4	D-leucyl-L-alanine	424.19	424.9
5	((*R*)-2-aminohexanoyl)-*L*-alanine	424.19	424.98
6	((*S*)-2-amino-3-(thiazol-4-yl)propanoyl)-*L*-alanine	465.13	466.22
7	((*R*)-2-amino-3-cyclohexylpropanoyl)-*L*-serine	480.22	481.07
8	L-tyrosyl-L-serine	490.17	491.47
9	D-valyl-L-serine	426.17	427.3
10	L-prolyl-L-threonine	438.17	439.38
11	D-threonyl-L-threonine	442.17	443.38
12	((*S*)-2-amino-2-(2,3-dihydro-1*H*-inden-2-yl)acetyl)-*L*-threonine	514.2	515.32
13	L-valyl-L-leucine	452.22	453.24
14	N^6-acetyl-L-lysyl-L-leucine	523.26	524.04
15	L-glutaminyl-L-leucine	481.21	482.43
16	L-threonyl-L-valine	440.19	440.9
17	L-alanyl-L-valine	410.18	410.82
18	((R)-2-amino-3-cyanopropanoyl)-L-valine	435.17	436.26
19	((*S*)-2-amino-4-methoxy-4-oxobutanoyl)-*L*-valine	468.18	469.47
20	(*S*)-(2-amino-4-methoxy-4-oxobutanoyl)glycine	426.13	427.38
21	L-glutaminyl-L-phenylalanine	515.2	516.6
22	((*S*)-2-amino-2-(2,3-dihydro-1*H*-inden-2-yl)acetyl)-*L*-phenylalanine	560.22	561.41
23	L-leucyl-L-phenylalanine	500.22	501.24
24	D-valyl-L-tyrosine	502.2	503.22
25	((*S*)-2-amino-5-ureidopentanoyl)-*L*-tyrosine	560.22	561.49
26	((*R*)-2-amino-3-(4-chlorophenyl)propanoyl)-*L*-tyrosine	584.16	585.18
27	D-glutaminyl-L-tryptophan	554.21	555.48
28	L-seryl-L-tryptophan	513.18	514.52
29	((*S*)-2-amino-3-(4-chlorophenyl)propanoyl)-*L*-tryptophan	607.18	608.45
30	L-asparaginyl-L-isoleucine	467.2	468.21
31	L-threonyl-L-isoleucine	454.2	455.22
32	D-alanyl-L-isoleucine	424.2	425.38
33	L-threonyl-L-glutamine	469.18	470.11
34	((*R*)-2-amino-3-cyanopropanoyl)-*L*-glutamine	464.16	465.31
35	((*S*)-2-amino-3-cyclohexylpropanoyl)-*L*-glutamine	521.24	522.52
36	N-glycyl-N-methylglycine	368.14	368.73
37	(*S*)-*N*-(2-aminohexanoyl)-*N*-methylglycine	424.2	425.22
38	N-(N^6,N^6-dimethyl-L-lysyl)-N-methyl-L-alanine	481.25	482.35
39	N-(L-tryptophyl)-N-methyl-L-alanine	511.2	512.52
40	(*R*)-1-(*N*^{6}-acetyl-*L*-lysyl)-4,4-difluoropyrrolidine-2-carboxylic acid	543.21	544.52
41	N⌉,N⌉-dimethylarginylglycine	481.22	482.51
42	((*S*)-2-aminohexanoyl)-*L*-tyrosine	516.22	517.56
43	(*S*)-2-(2-aminoacetamido)-4-(*tert*-butoxy)-4-oxobutanoic acid	468.18	491.18 (+ Na)
44	D-seryl-D-alanine	398.14	399.15
45	((*S*)-2-amino-3-(pyridin-3-yl)propanoyl)-*D*-alanine	459.17	461.19
46	D-alanyl-D-alanine	382.14	405.14
47	D-leucyl-D-serine	440.19	441.06
48	D-tryptophyl-D-serine	513.18	514.04
49	L-alanyl-L-threonine	412.16	413.17
50	((*S*)-2-aminohexanoyl)-*D*-threonine	454.2	456.27
51	((*R*)-2-amino-3-(thiazol-4-yl)propanoyl)-*D*-serine	481.12	481.55
52	((*S*)-3-amino-3-(3-(trifluoromethyl)phenyl)propanoyl)-*D*-threonine	556.17	557.17
53	D-tyrosyl-D-leucine	516.22	517.48
54	glycyl-*D*-leucine	410.18	411.22
55	D-phenylalanyl-D-leucine	500.22	501.47
56	L-leucyl-D-valine	452.22	453.14
57	((*R*)-3-amino-3-(3-(trifluoromethyl)phenyl)propanoyl)-*D*-valine	554.19	555.41

58	((*R*)-2-amino-3-(pyridin-3-yl)propanoyl)-*D*-valine	487.2	488.51
59	((*R*)-2-amino-5-ureidopentanoyl)-*D*-phenylalanine	544.22	545.4
60	L-tyrosyl-L-phenylalanine	550.2	551.48
61	L-alanyl-D-tyrosine	474.17	475.19
62	D-threonyl-D-tyrosine	504.18	505.2
63	L-isoleucyl-D-tyrosine	516.22	517
64	L-valyl-D-tryptophan	525.22	526.6
65	L-asparaginyl-L-glutamine	482.17	483.55
66	2-((*S*)-2-amino-3-phenylpropanamido)-3-hydroxy-2-methylpropanoic acid	488.19	489.63
67	2-((*S*)-2-amino-4-methylpentanamido)-3-hydroxy-2-methylpropanoic acid	454.2	455.3
68	2-((*S*)-2-amino-3-phenylpropanamido)-3-(1*H*-imidazol-1-yl)propanoic acid	524.2	525.56
69	2-((*S*)-2-amino-5-ureidopentanamido)-3-(1*H*-imidazol-1-yl)propanoic acid	534.21	535.48
70	D-seryl-L-proline	424.16	425.3
71	L-prolyl-L-proline	434.18	435.3
72	D-prolyl-D-proline	434.18	434.84
73	glycyl-*D*-proline	394.14	395.21
74	D-alanyl-D-proline	408.16	409.13
75	glycyl-L-proline	394.14	395.37
76	N6-(4-sulfamoylbenzoyl)-L-lysine	551.16	552.44
77	N6-(3-(5-(2-(trifluoromethyl)phenyl)furan-2-yl)propanoyl)-L-lysine	634.22	635.42
78	S-(5'-adenosyl)-L-homocysteine	606.18	607.57

3.4. Amino Acid Coupling Screening

The 78 synthesized dipeptides & positive controls, as well as 146 commercially obtained natural and unnatural amino acids were screened for successful amidation with scaffold-linked “test DNA”. The purpose of the experiment was to identify highest yield, generally applicable amidation conditions for library synthesis as well as pre-select building blocks for higher reaction yield and thus library quality.

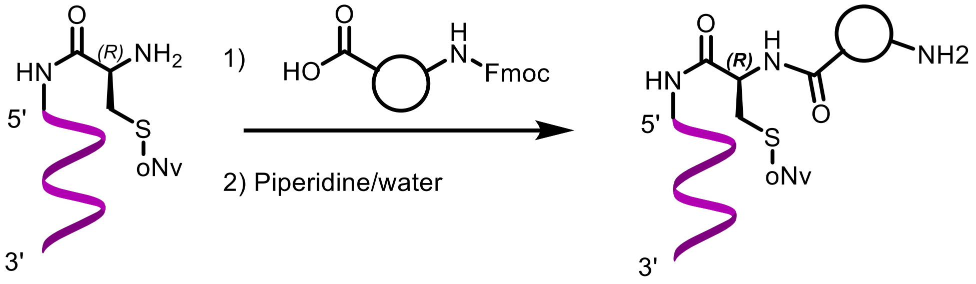

0.5 nmol of scaffold-linked test DNA was inserted into 1 mL 96-well plates and reacted with each respective building block according to general procedures for DMT-MM & diazotransfer in section 2.2, which were scaled accordingly and the following procedure for EDC/HOAt:

Unoptimized Dipeptide Building Block Coupling Procedure (EDC/ HOAt)
Dissolve DNA in 10 µL MOPS buffer (100 mM, 1M NaCl). In a separate vial add 6 µL 200 mM carboxylic acid in DMSO with 6 µL EDC/HOAt/NMM (100:20:100 mM) and 17 µL DMSO, stir for 10 min at rt. Add this mixture to DNA solution and stir at 37°C for 1 h. Second activation of carboxylic acid was repeated again (6 µL Fmoc AA OH and 6 µL EDC/HOAt/NMM) and added to DNA mixture, stirred at 37°C for another 1 h.

After reaction completion, DNA was precipitated. The Fmoc group was then removed from all newly installed building blocks using the general procedure. The piperidine was evaporated, DNA precipitated, redissolved and checked via LC-MS. Results are summarized in figure S9.

Based on screening data, DMT-MM was chosen for coupling of non-dipeptide building blocks in the library due to its optimal general performance. For dipeptide coupling the DMT-MM protocol resulted in low yields, yet the EDC/HOAt protocol produced substantially better results. Hence it was chosen for use for dipeptides during library synthesis. Other protocols, mainly utilizing EDC/S-NHS produced considerably worse results than both DMT-MM and EDC/HOAt and thus were not investigated fully (data not shown).

For step 1 of library synthesis all building blocks with at least partial conversion were considered for inclusion. For step 2 only building blocks showing full conversion were considered for inclusion.

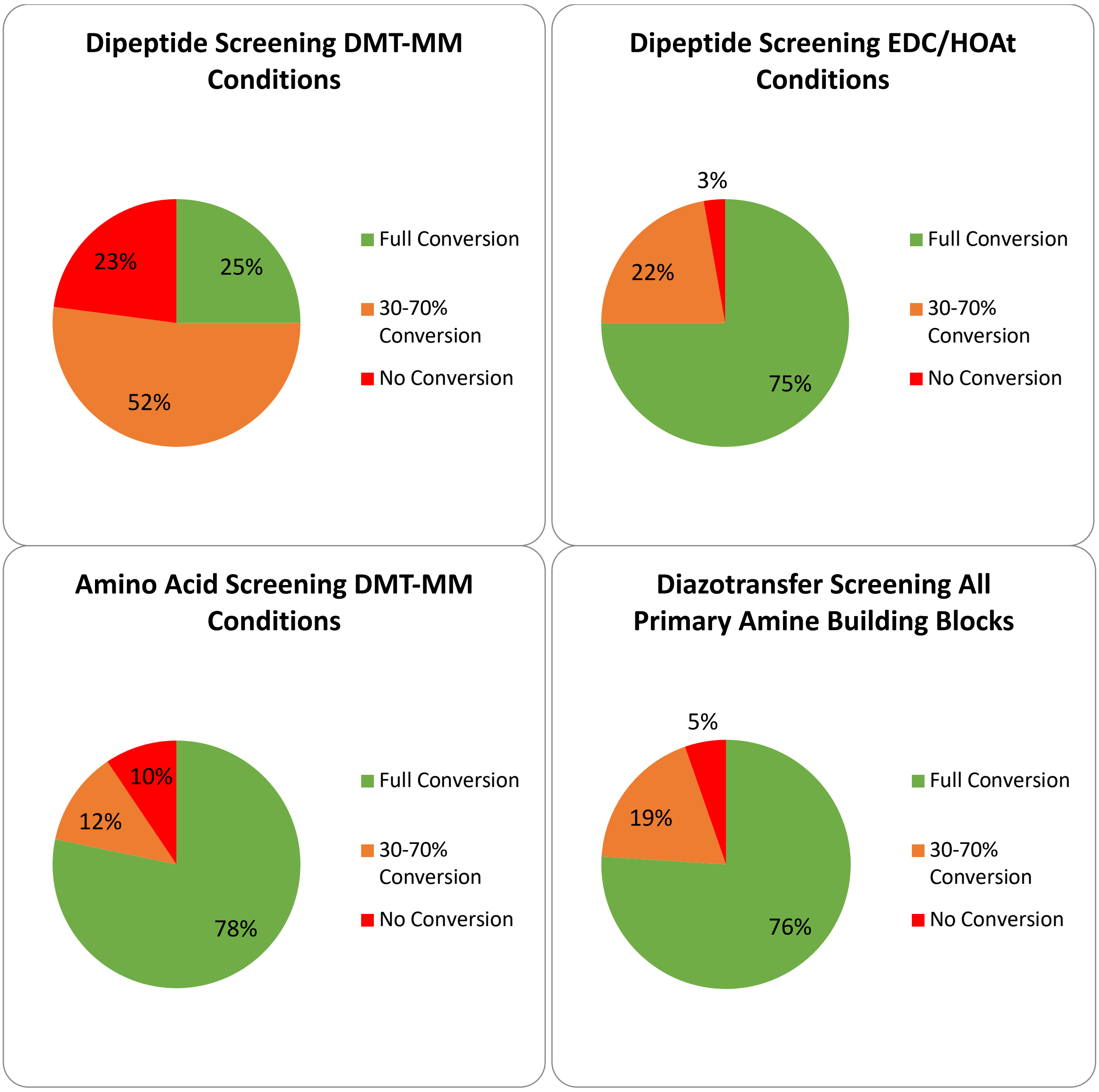

Figure S9: Summary of Reaction Screening for amino acid and dipeptide building blocks. Amidation on top of scaffold amino group of all building blocks was tested using general conditions described in section 2.2 and diazotransfer was tested on all building blocks possessing a primary amine

3.5. Dipeptide Coupling Optimization

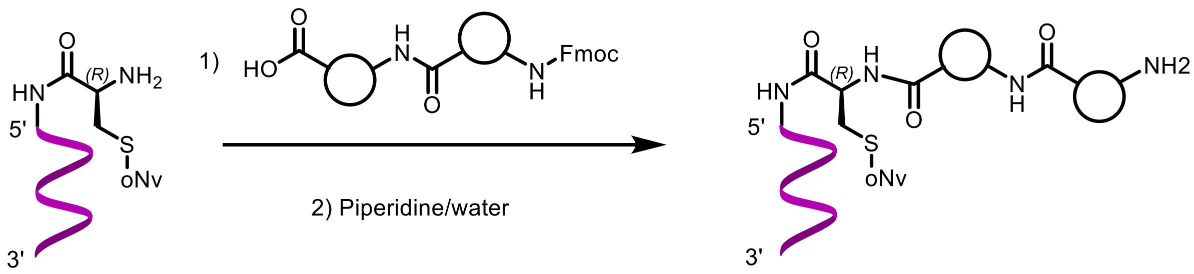

During dipeptide amidation screening, an observation was made that most dipeptide couplings result in two product peaks with identical MS signal once attached to the test DNA, contrary to single Fmoc amino acids. Purified dipeptides exhibited only single peaks after purification in HPLC, indicating lack of diastereomers. This meant that the appearance of the two peak products could be interpreted as racemization during amidation, necessitating optimization.

Reaction condition screening was performed according to section 3.4 on 0.5 nmol of scaffold-linked test DNA per test. Aim of the optimization was to decrease the appearance of two product peaks towards a single peak during LC-MS analysis, indicating reduced formation of diastereomers. Formation of secondary peaks could not be completely prevented, although was markedly decreased. During step 1 library purification, only the main peak product was isolated whenever possible.

Table S3: Optimization of on-DNA dipeptide amidation using "test DNA" as starting material. Peak sizes are compared by integrating their UV peak at 260 nM and using the given numbers for comparison.

Condition	Details	% Main Peak Product	% Secondary Peak Product	Dipeptide
**1**	MOPS pH8 (100 mM, 1M NaCl) EDC/HOAt/NMM (100:20:100 mM) 37 C Reaction, 10 min preactivation	67.35	32.65	L-leucyl-L-phenylalanine
**2**	MOPS pH7 (100 mM, 1M NaCl) EDC/HOAt/NMM (100:20:100 mM) 26 C Reaction, no preactivation	74.29	25.71	L-leucyl-L-phenylalanine
**3**	MOPS pH8 (100 mM, 1M NaCl) EDC/HOAt/NMM (100:20:100 mM)	81.23	18.77	L-leucyl-L-phenylalanine

	26 C Reaction, no preactivation			
**4**	MOPS pH8 (200 mM, 1M NaCl)<br>EDC/HOAt/NMM (100:20:100 mM)<br>26 C Reaction, no preactivation	82.12	17.88	L-leucyl-L-phenylalanine
**5**	MOPS pH8 (200 mM, 3M NaCl)<br>EDC/HOAt/NMM (100:20:100 mM)<br>26 C Reaction, no preactivation	90.05	9.95	L-leucyl-L-phenylalanine

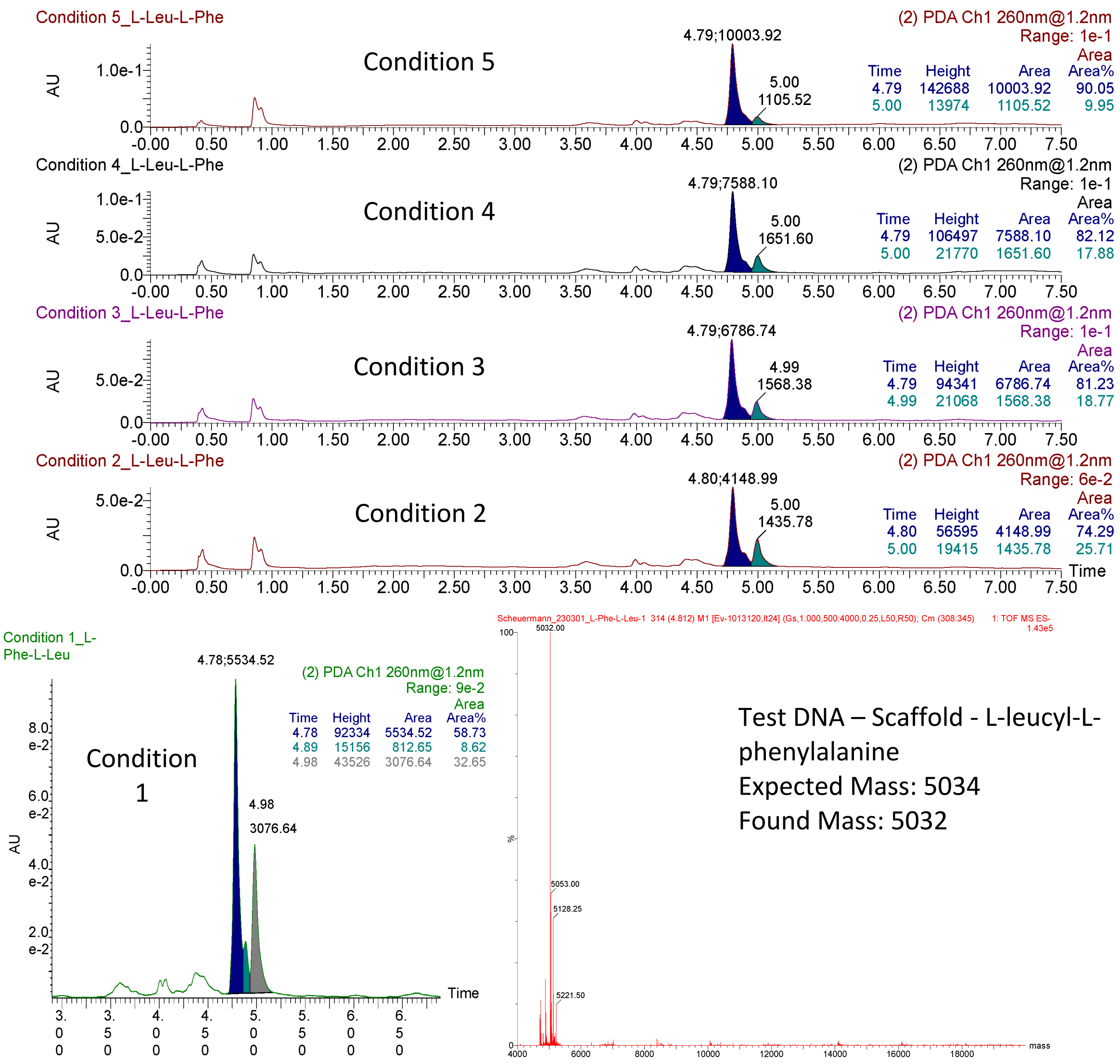

Figure S10: UV 260 nm chromatogram and MS characterization of dipeptide amidation using conditions in table S3. Product peaks are integrated and used for analysis

Table S4: Comparison of unoptimized amidation procedure 1 vs optimized procedure 5 for dipeptide amidation on DNA. Peak sizes are compared by integrating their UV peak at 260 nM and using the given numbers for comparison.

Condition	Dipeptide	% Main Peak Product	% Secondary Peak Product
1	L-seryl-L-tryptophan	56.26	42.96
5	L-seryl-L-tryptophan	76.83	23.17
1	L-glutaminyl-L-phenylalanine	69.06	30.26
5	L-glutaminyl-L-phenylalanine	81.21	18.79
1	((*S*)-2-amino-5-ureidopentanoyl)-*L*-tyrosine	59.8	39.15
5	((*S*)-2-amino-5-ureidopentanoyl)-*L*-tyrosine	73.85	17.55

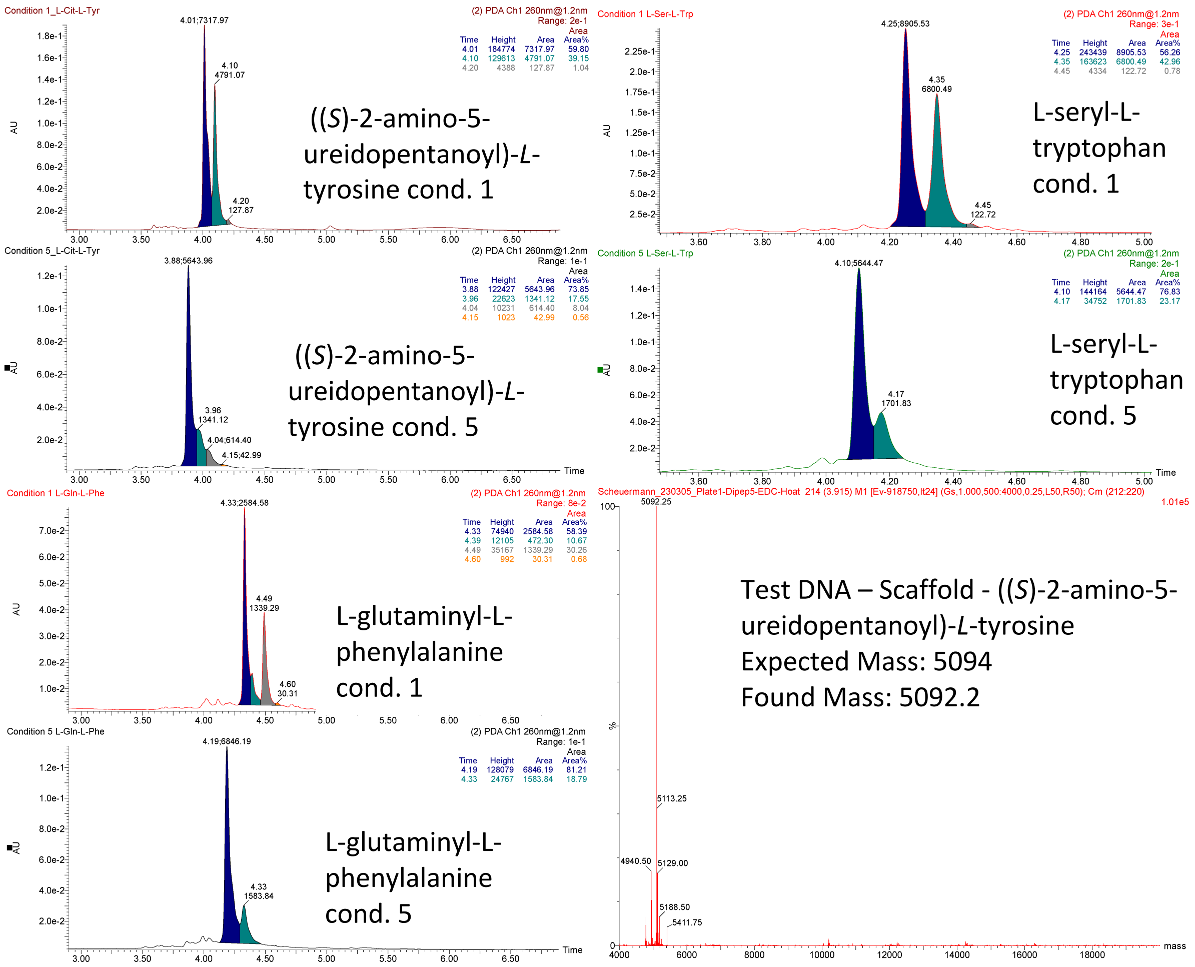

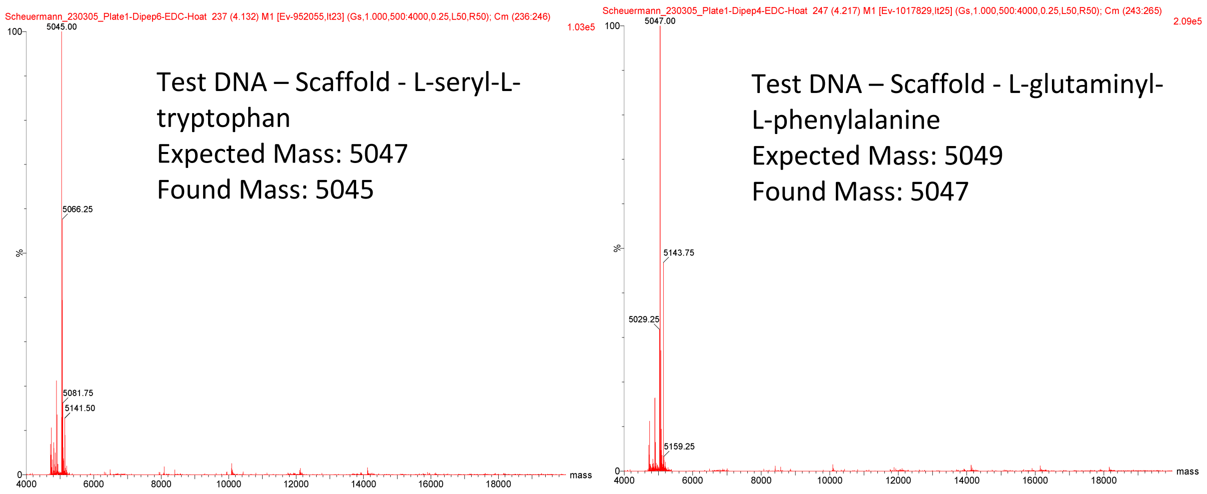

Figure S11: UV 260 nm chromatogram and MS characterization of dipeptide amidation using conditions in table S3 and results summarized in table S4. Product peaks are integrated and used for analysis

3.6. Model Compound Synthesis

Model Compounds were synthesized by consecutive reactions according to general methods listed in section 2.2. 50 nmol was taken as starting material for each 3’ NH2 HP3 and 5’ NH2 HP5 strand respectively. Fmoc deprotections were quenched by evaporating the deprotection solution via speed vacuum, followed by DNA precipitation. Upon completion of the respective HP5/HP3 model compound synthesis, each strand was individually purified via HPLC and precipitated. Yields after HPLC-purification ranged between 2-5 nmol for each strand. Both strands were then mixed equimolarly to participate in an interstrand click reaction. The click reaction product was confirmed via LC-MS and purified with HPLC again to remove copper and other impurities. The covalently linked strands were then placed in UV chamber to participate in scaffold deprotection and thioether formation using 1,3-bis(bromomethyl)benzene as a prototypical bis-electrophile. Formation of disulfide after scaffold deprotection could be inferred indirectly by attempting thioether formation without addition of TCEP or other reductant, in which case no reaction could be observed.

Model Compound 1:

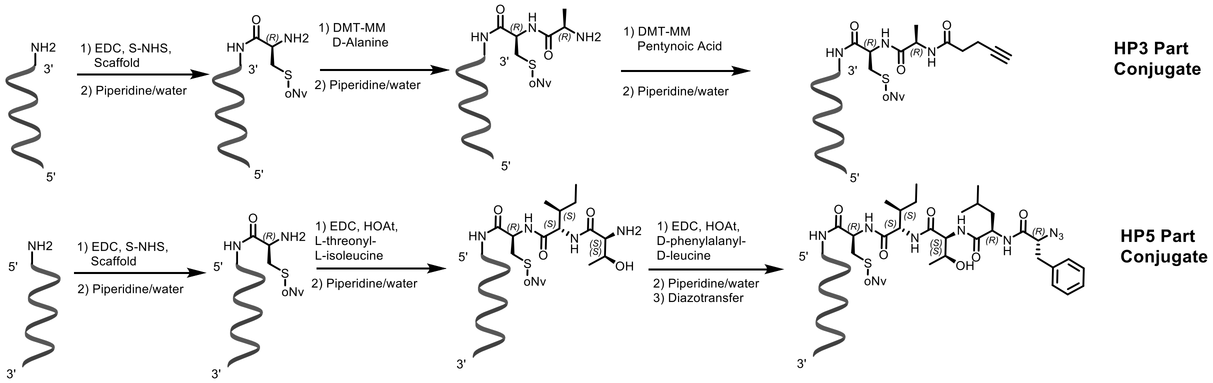

Figure S12: Synthetic scheme for the synthesis of model compound 1 through the combination of HP5 & HP3 strands

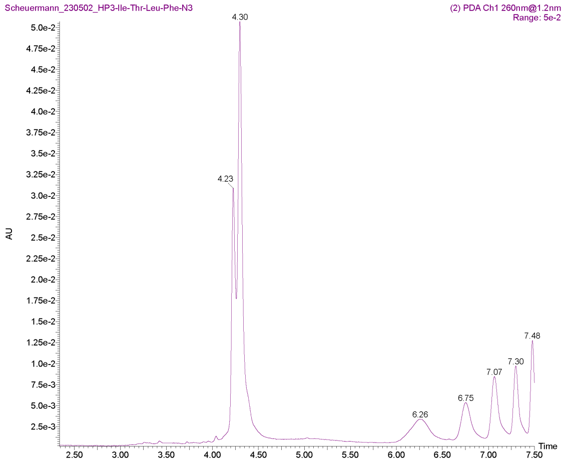
Scheuermann_230502_HP3-Ile-Thr-Leu-Phe-N3
(2) PDA Ch1 260nm@1.2nm
Range: 5e-2
AU
Time
4.30
4.23
6.26
6.75
7.07
7.30
7.48

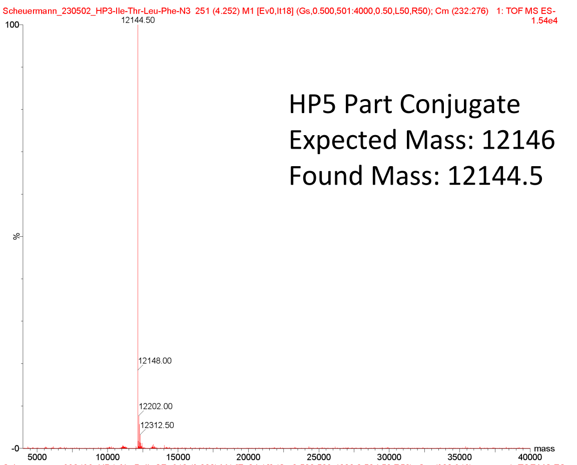
Scheuermann_230502_HP3-Ile-Thr-Leu-Phe-N3 251 (4.252) M1 [Ev0,It18] (Gs,0.500,501:4000,0.50,L50,R50); Cm (232:276) 1: TOF MS ES-
1.54e4
12144.50
12148.00
12202.00
12312.50
%
mass
HP5 Part Conjugate
Expected Mass: 12146
Found Mass: 12144.5

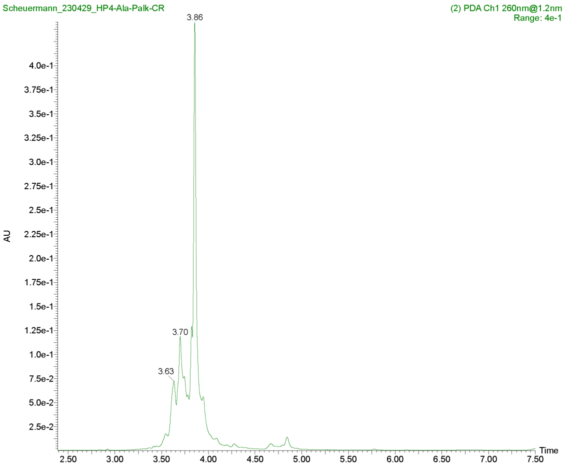
Scheuermann_230429_HP4-Ala-Palk-CR
(2) PDA Ch1 260nm@1.2nm
Range: 4e-1
AU
Time
3.86
3.70
3.63

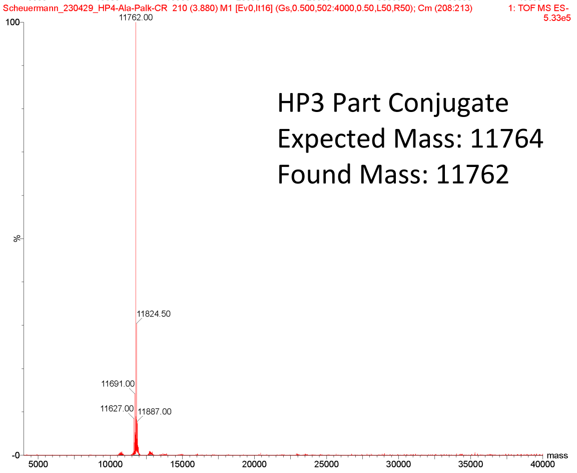
Scheuermann_230429_HP4-Ala-Palk-CR 210 (3.880) M1 [Ev0,It16] (Gs,0.500,502:4000,0.50,L50,R50); Cm (208:213) 1: TOF MS ES-
5.33e5
11762.00
11824.50
11691.00
11627.00
11887.00
%
mass
HP3 Part Conjugate
Expected Mass: 11764
Found Mass: 11762

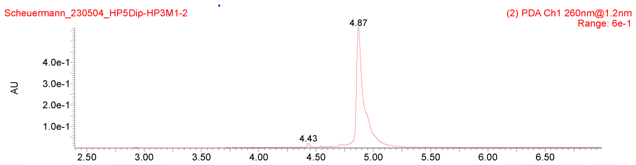
Scheuermann_230504_HP5Dip-HP3M1-2
(2) PDA Ch1 260nm@1.2nm
Range: 6e-1
AU
4.87
4.43

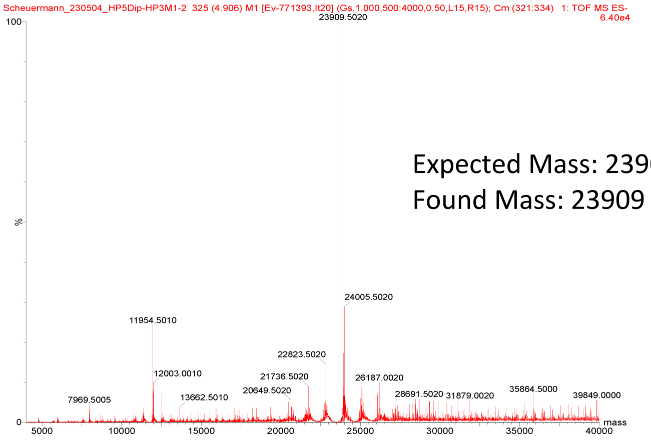
Scheuermann_230504_HP5Dip-HP3M1-2 325 (4.906) M1 [Ev-771393,It20] (Gs,1.000,500:4000,0.50,L15,R15); Cm (321:334) 1: TOF MS ES-
6.40e4
23909.5020
24005.5020
11954.5010
12003.0010
22823.5020
21736.5020
20649.5020
26187.0020
28691.5020
31879.0020
35864.5000
39849.0000
13662.5010
7969.5005
%
mass
Expected Mass: 23907
Found Mass: 23909

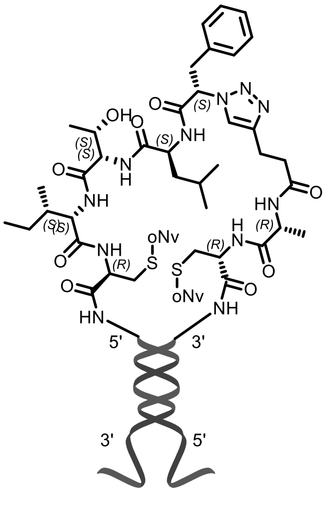
OH
NH
oNv
S
HN
5'
3'

“clicked”

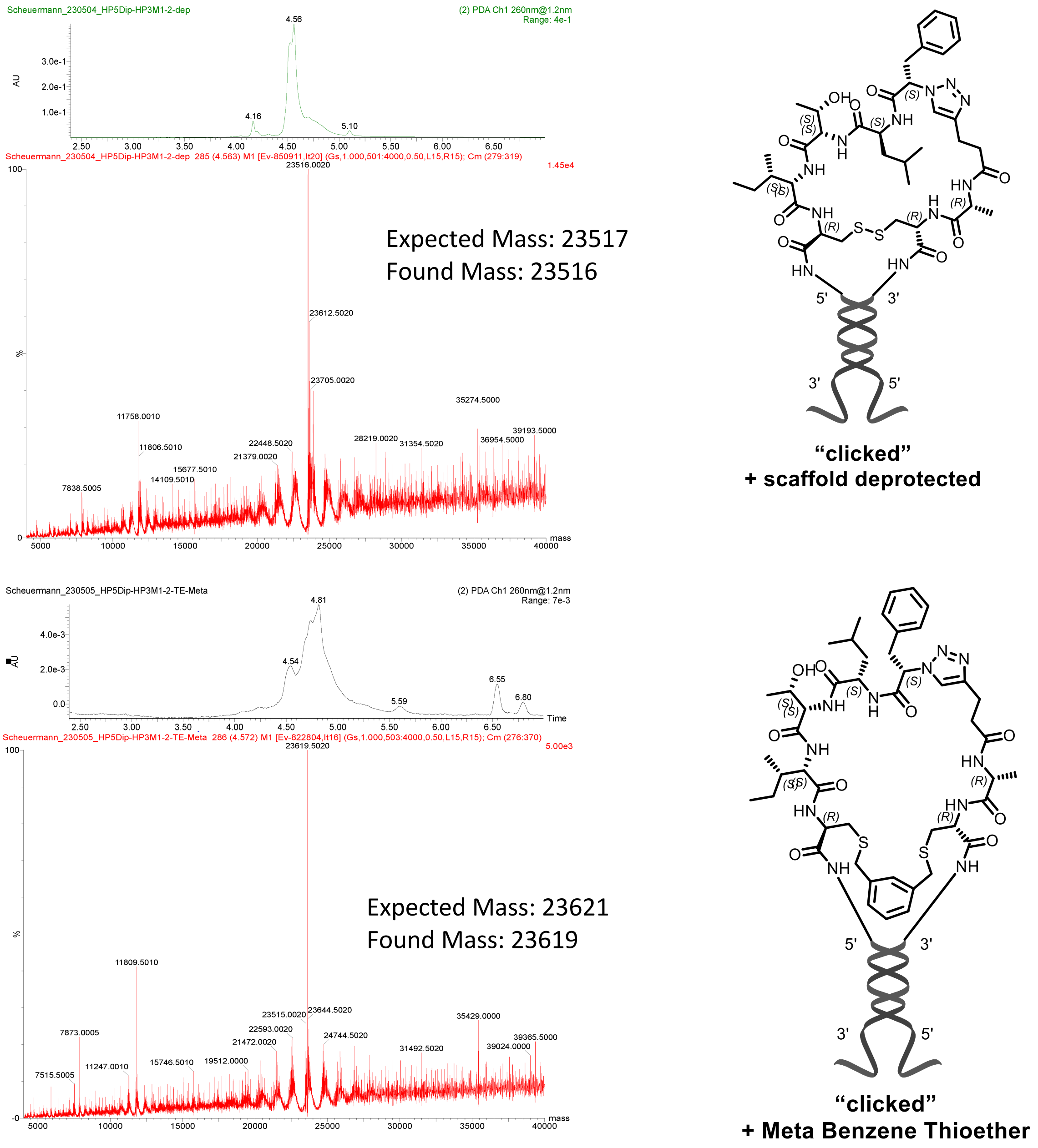

Figure S13: UV 260 nm chromatogram and MS characterization of the synthesis of Model Compound 1

Model Compound 2:

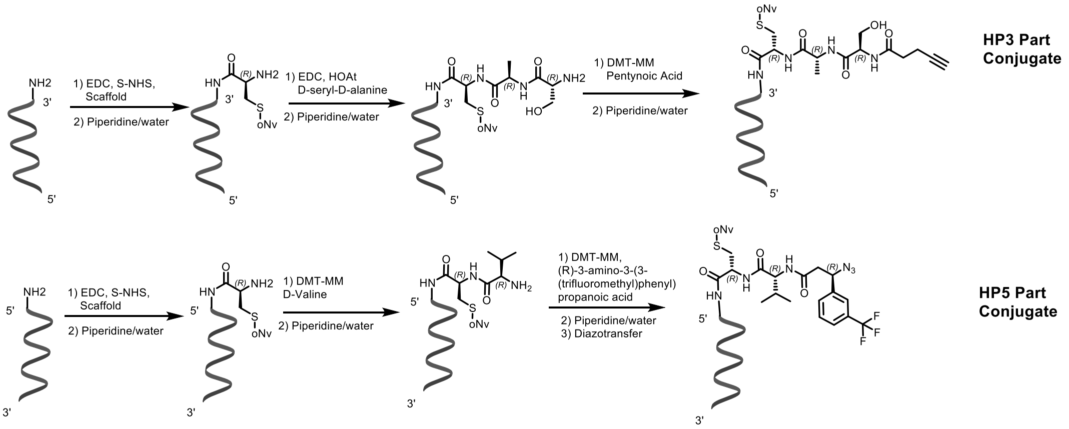

Figure S14: Synthetic scheme for the synthesis of model compound 2 through the combination of HP5 & HP3 strands

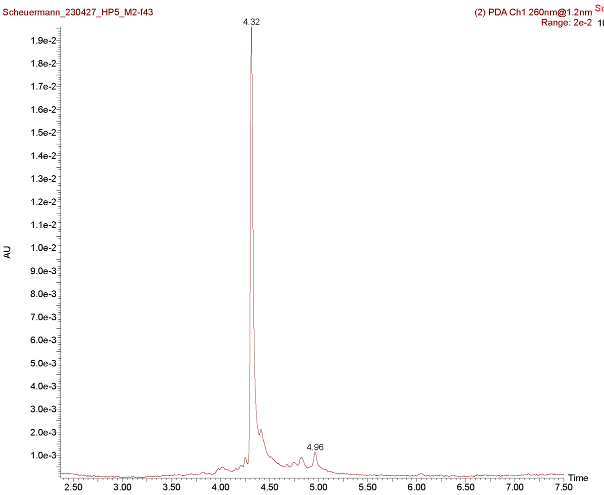

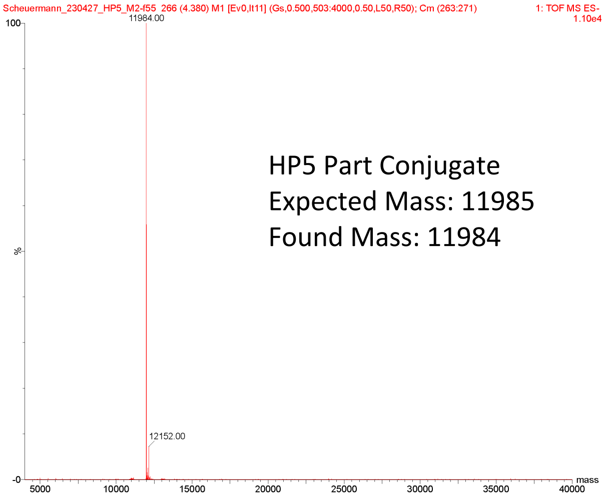

HP5 Part Conjugate
Expected Mass: 11985
Found Mass: 11984

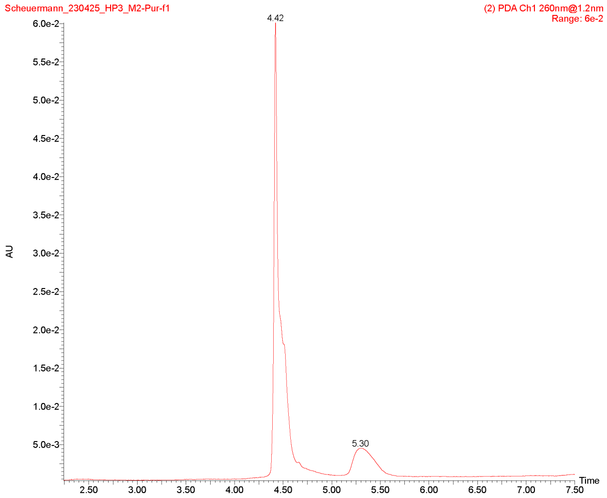

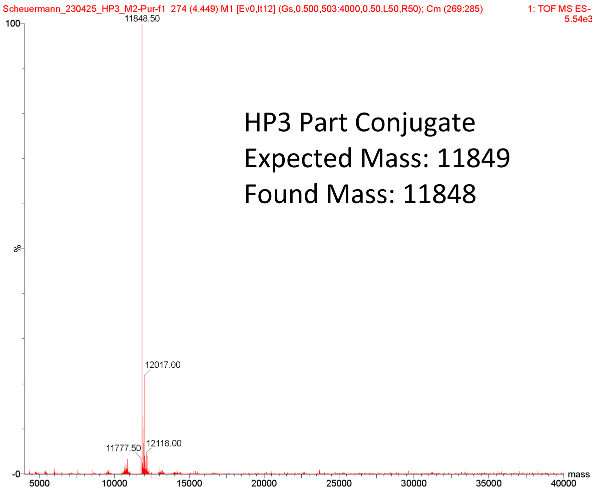

HP3 Part Conjugate
Expected Mass: 11849
Found Mass: 11848

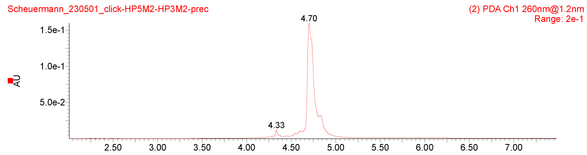

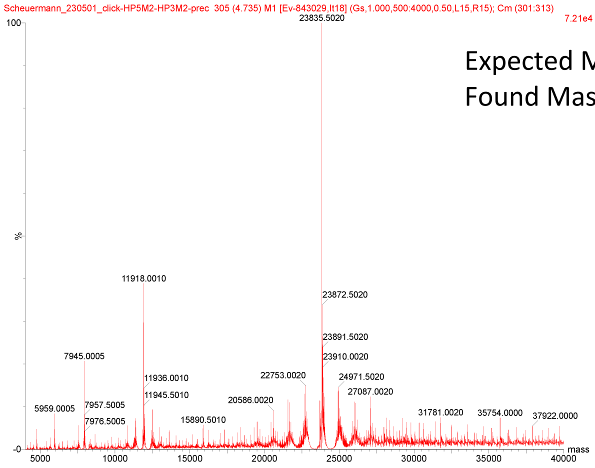

Expected Mass: 23833
Found Mass: 23835

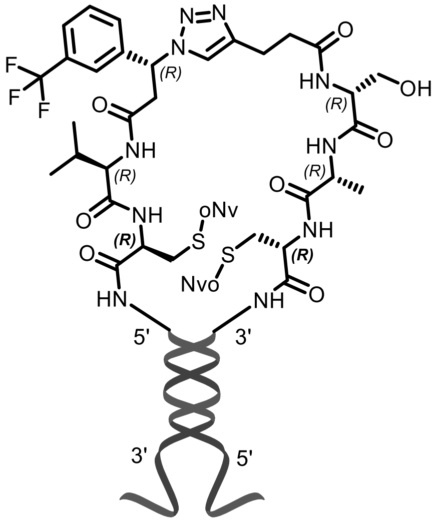

“clicked”

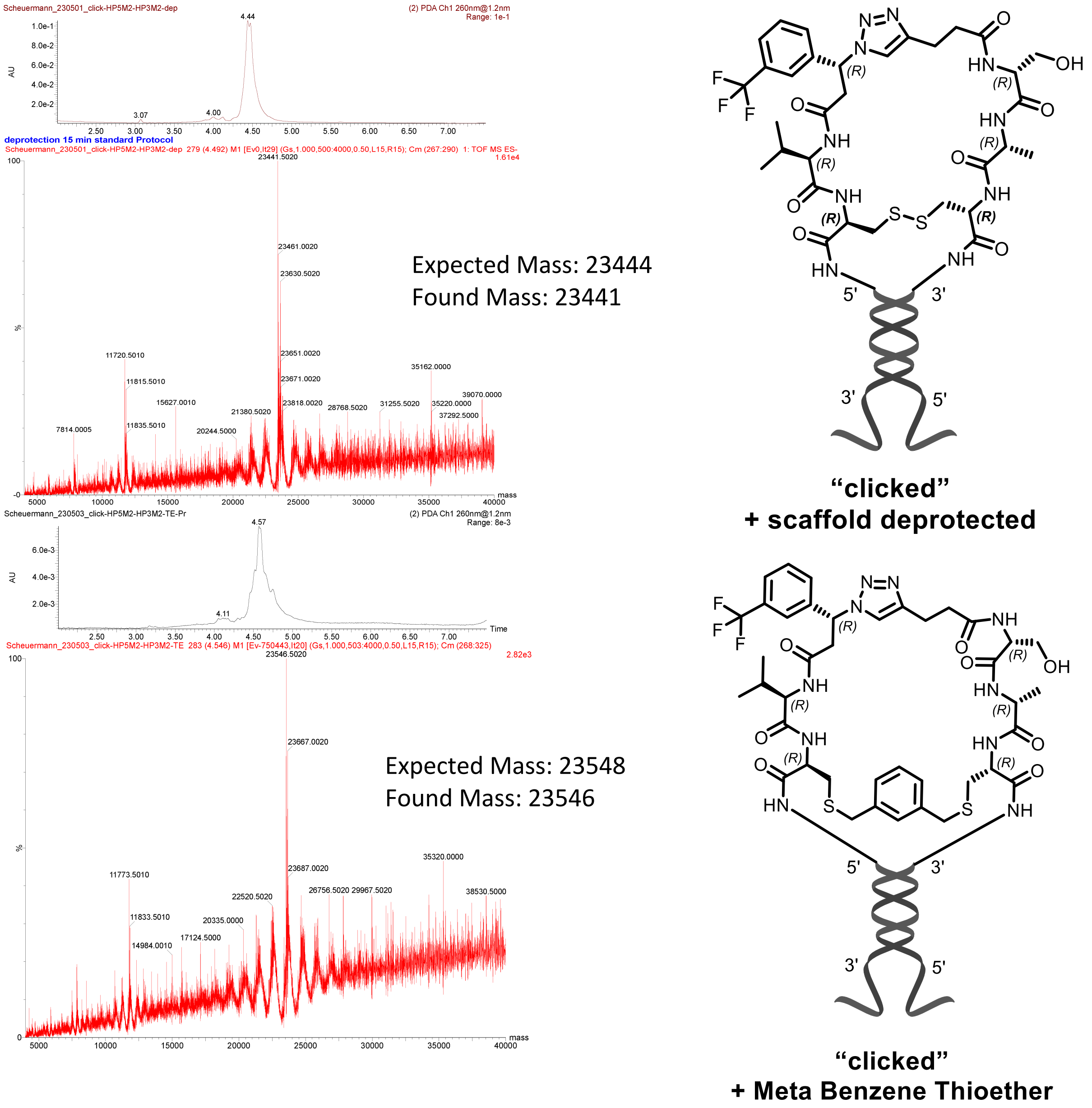

Figure S 15: UV 260 nm chromatogram and MS characterization of the synthesis of Model Compound 2

Model Compound 3:

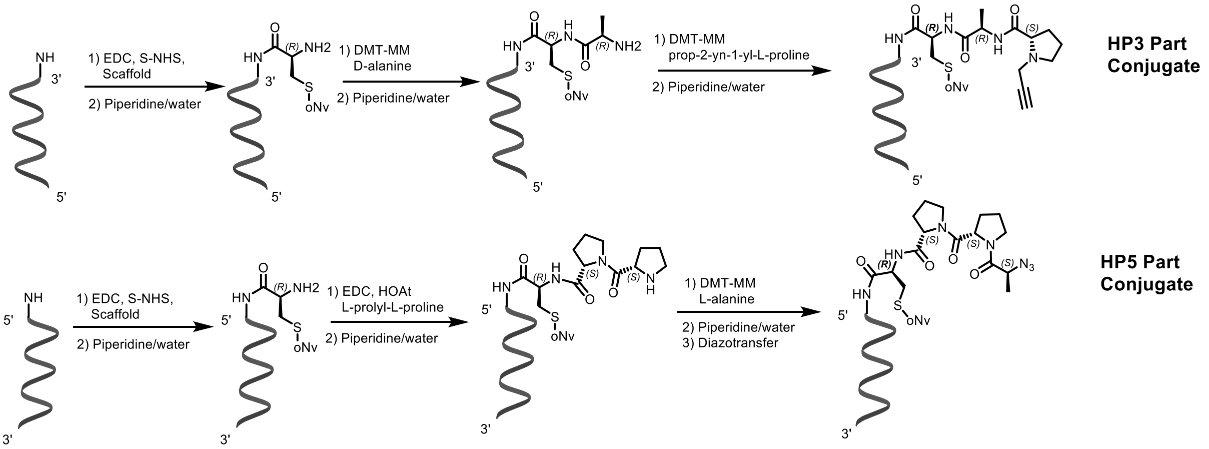

Figure S16: Synthetic scheme for the synthesis of model compound 3 through the combination of HP5 & HP3 strands

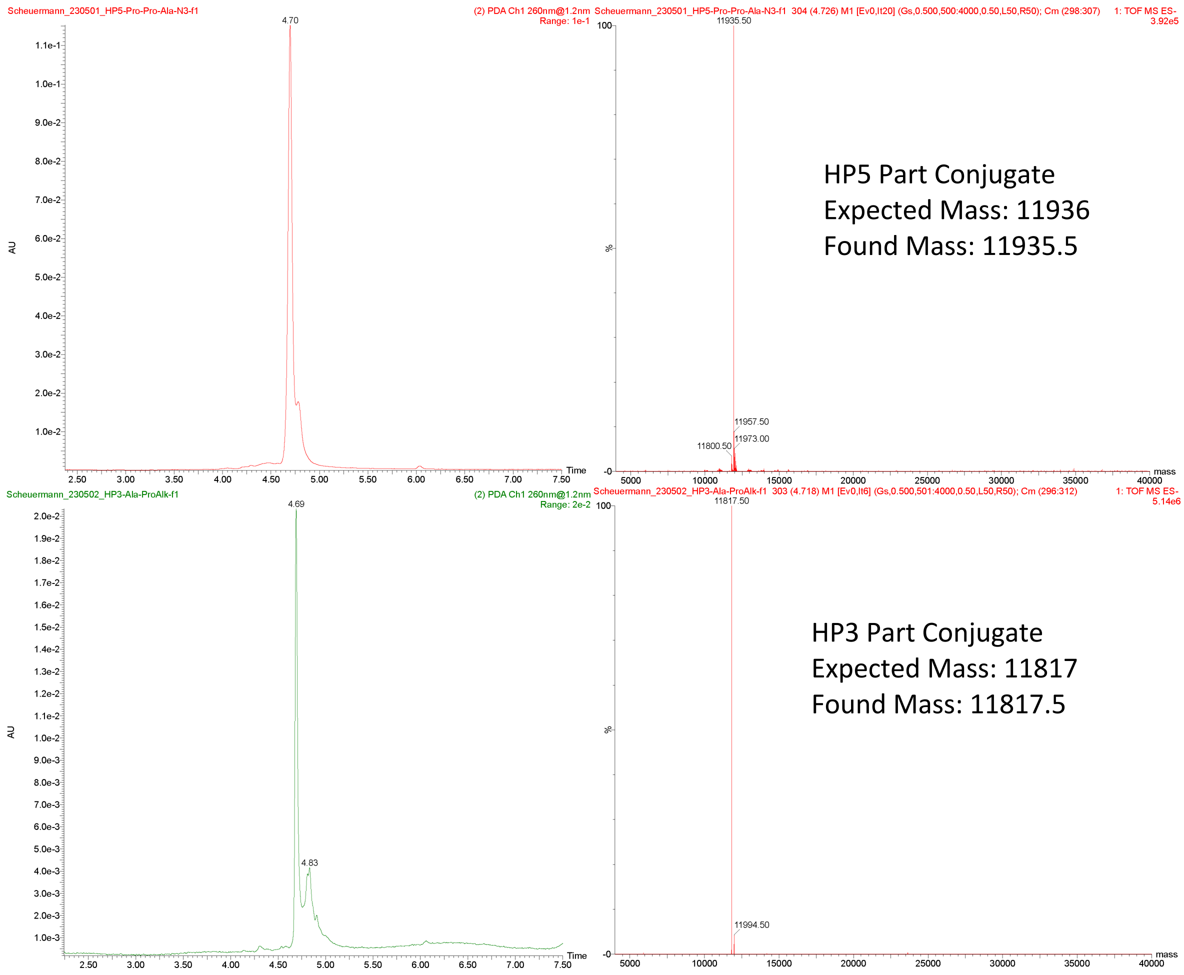

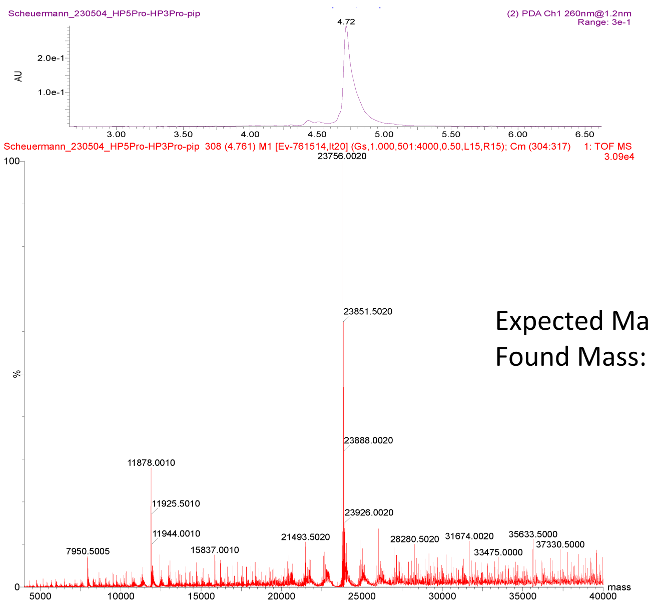

Expected Mass: 23753
Found Mass: 23756

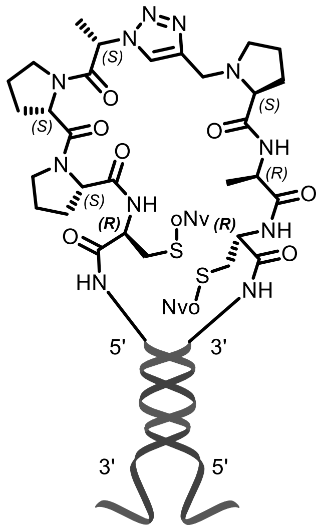

“clicked”

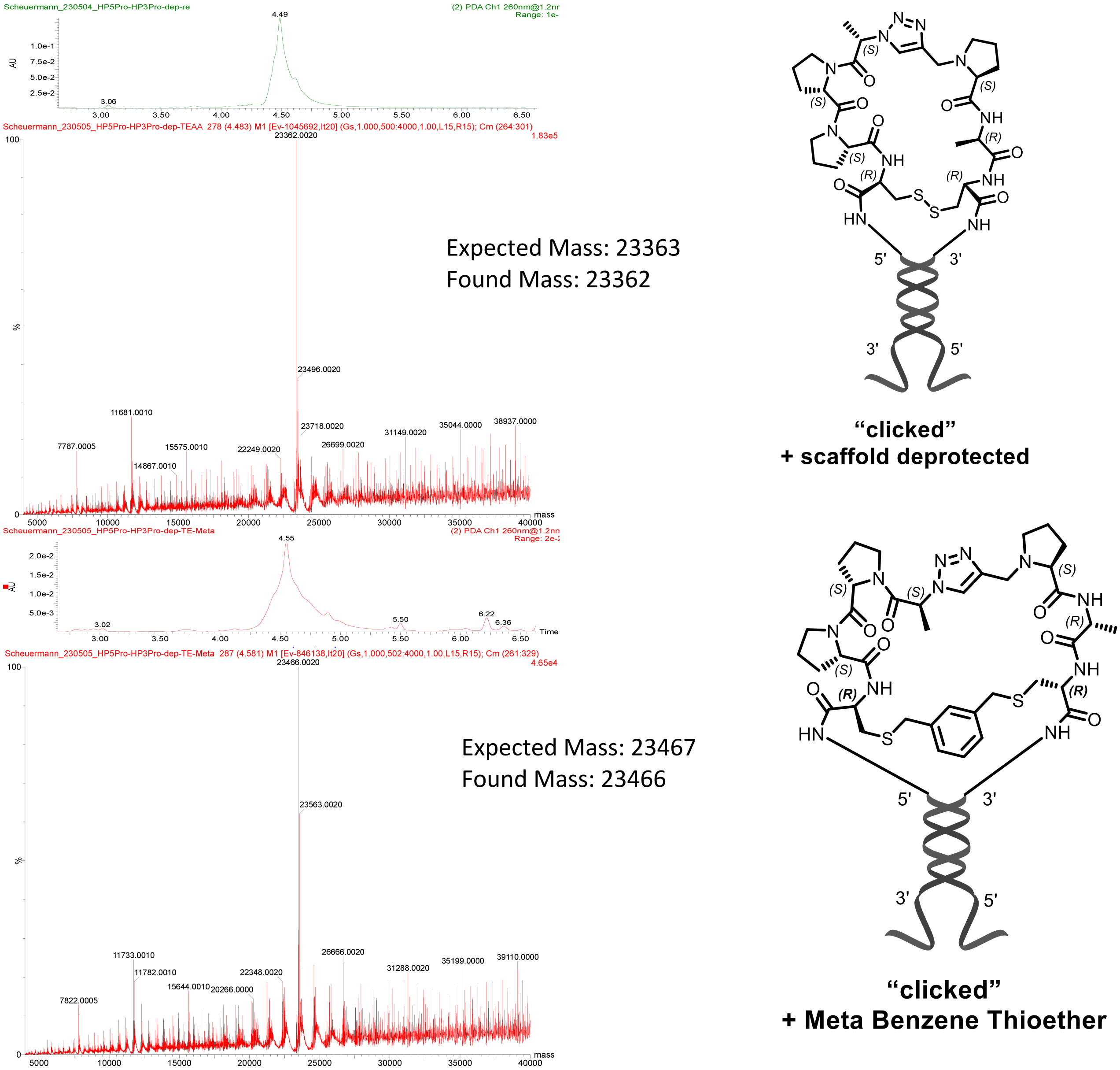

Figure S17: UV 260 nm chromatogram and MS characterization of the synthesis of Model Compound 3

4. Library Synthesis

4.1. Library Architecture

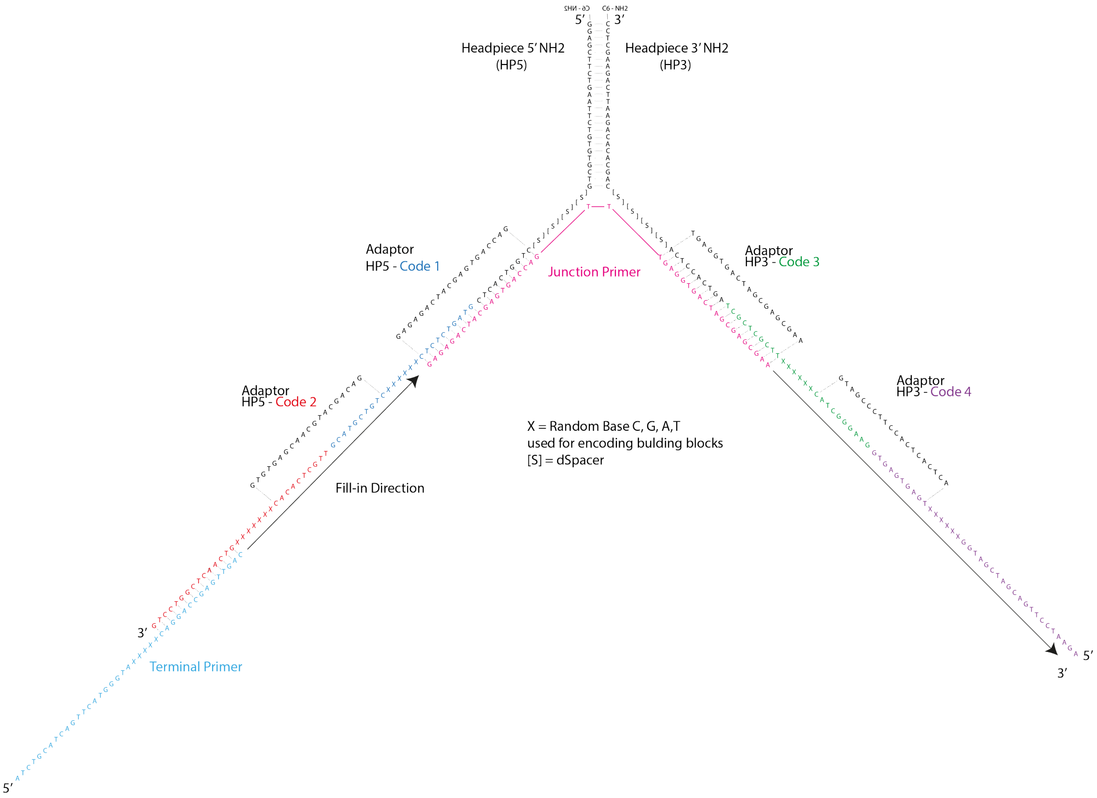

Figure S18: Library Architecture prior to post-selection amplification

Table S5: Sequences of oligonucleotides used for DP-DEL creation. X position denotes variable base used for library encoding of specific members.

Sequence Name	Sequence	Mass (Da)
Headpiece 5′ NH2 (**HP5**)	5′ **NH2-C6**-GGAGCTTCTGAATTCTGTGTGCTG**[dSpacer][dSpacer][dSpacer][dSpacer]**CTGGTCACTC 3′	11346
Headpiece 3′ NH2 (**HP3**)	5′ **Phos**-AGTCACCTCA**[dSpacer][dSpacer][dSpacer][dSpacer]**CAGCACACAGAATTCA GAAGCTCC-C6-**NH2** 3′	11313
Code 1 HP5 (BB1 code)	5′ **Phos**-GTAGTCTCTC XXXXXX CTGTCGTACG 3′	
Code 3 HP3 (BB3 code)	5′**Phos**-GAAGGGCTAC XXXXXX TTCGCTCGCT 3′	
Code 2 HP5 (BB2 code)	5′**Phos**-TTGCTCACAC XXXXXXX GTCAACTCGGTCCTG 3′	
Code 4 HP3 (BB4 code)	5′-AGAATCCTTGACGATCGATGG XXXXXXX TGAGTGAGTG-3′	
HP5 Code 1 Adaptor	5′ GAGAGACTACGAGTGACCAG 3′	6200
HP3 Code 3 Adaptor	5′ TGAGGTGACTAGCGAGCGAA 3′	6231
HP5 Code 2 Adaptor	5′ GTGTGAGCAACGTACGACAG 3′	6191
HP3 Code 4 Adaptor	5′ GTAGCCCTTCCACTCACTCA 3′	5972
Terminal Primer (BB5 code)	5′ ATCTGCATCAGTTCATGGGTA XXXXX CAGGACCGAGTTGAC 3′	
Junction Primer	5′ Phos-GAGAGACTACGAGTGACCAGTTTGAGGTGACTAGCGAGCGAA 3′	13180

4.2. Scaffold Coupling

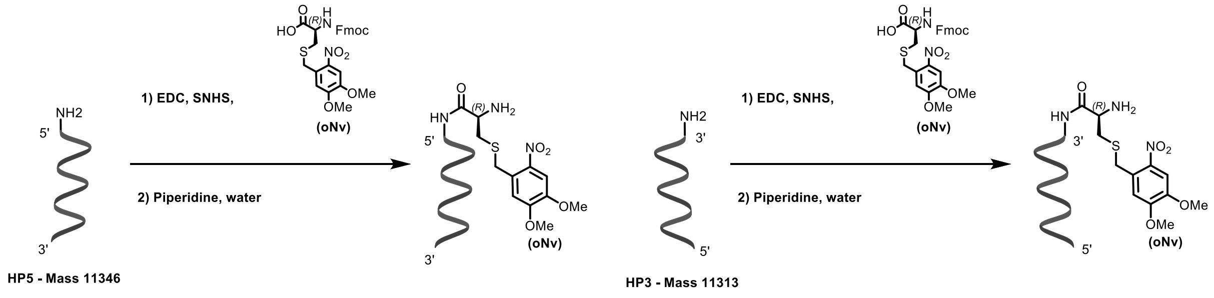

17.5 µmol of each respective headpiece HP5 and HP3 was taken as starting material and reacted with the cysteine scaffold according to outlined protocol in section 2.2. 17 reaction batches of 1 µmol and 1 batch of 0.5 µmol were performed for each headpiece. The resulting crude product was Fmoc deprotected on a per-batch basis, the piperidine was evaporated via speed vacuum and the resultant DNA was redissolved and precipitated. The precipitated DNA pellets were dissolved in mQ and the batch products were joined together. Scaffold attachment for HP5 afforded 16.5 µmol (94%) yield and for HP3 afforded 16.062 µmol (91%) yield. The product for both headpieces was deemed of sufficient quality and used for step 1 library synthesis without further purification.

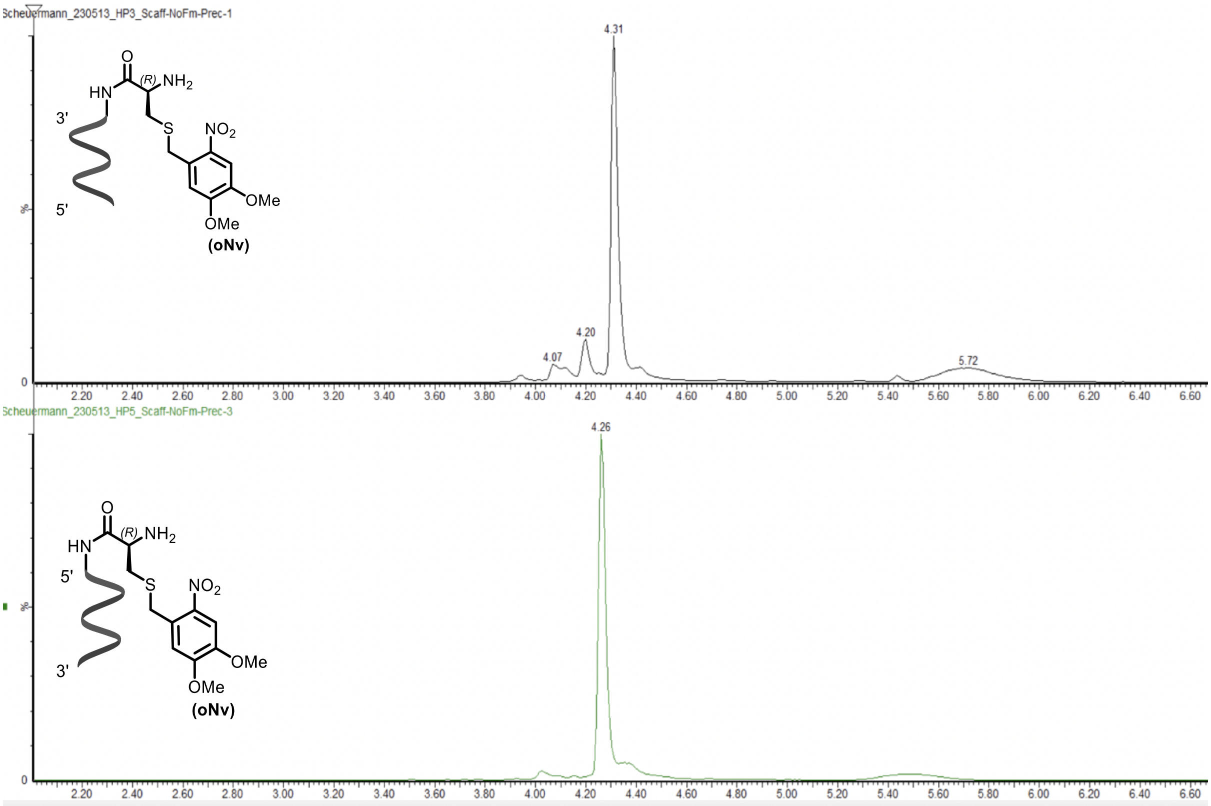

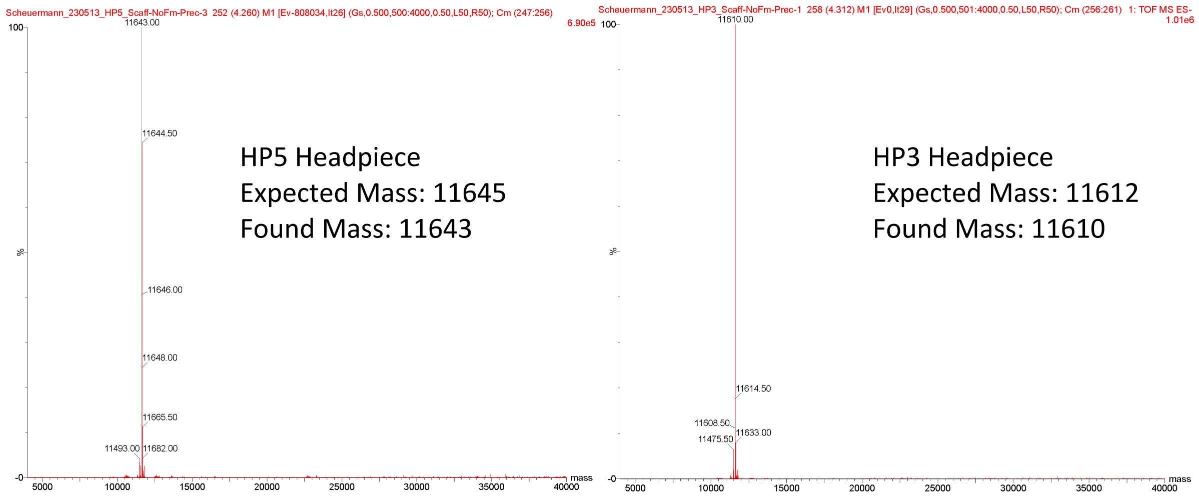

Figure S19: UV 260 nm and MS characterizations of conjugating cysteine scaffold onto HP5 and HP3 library strands

4.3. Step 1 Headpiece 5' (HP5) Sublibrary

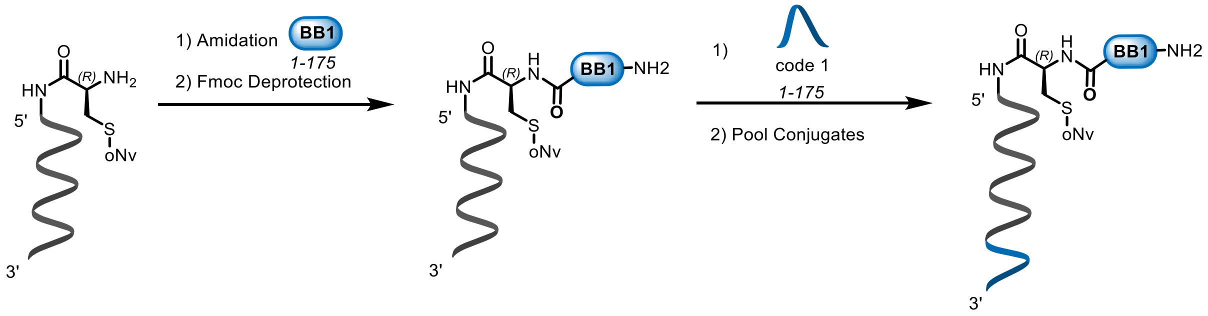

Scaffold modified HP5 DNA was split in 60 nmol portions in 2 mL Eppendorf tubes. 175 amidation reactions were then individually performed for the attachment of all BB1 building blocks. Dipeptide amidations were performed according the optimized EDC/HOAt protocol in section 2.2 and the rest of the amidations were performed using the DMT-MM protocol in the same section. The reaction products were then redissolved and individually Fmoc deprotected. The deprotection was quenched with sodium acetate buffer, followed by the addition of ethanol to precipitate the DNA product. All 175 reaction products were then individually HPLC-purified, precipitated and their yield was measured. 29 nmol for each HPLC-purified conjugate was taken for encoding step.

Each conjugate was individually encoded using code 1 strands according to procedure in section 2.2. After confirmation of ligation completion via LC-MS all 175 encoded conjugates were pooled and precipitated. The conjugate pool was then purified via HPLC at 60 °C, which enabled separation from excess codes and adaptor left over from the encoding step. The purified pool was precipitated again and analyzed. This afforded a final step 1 HP5 pool of 4400 nmol.

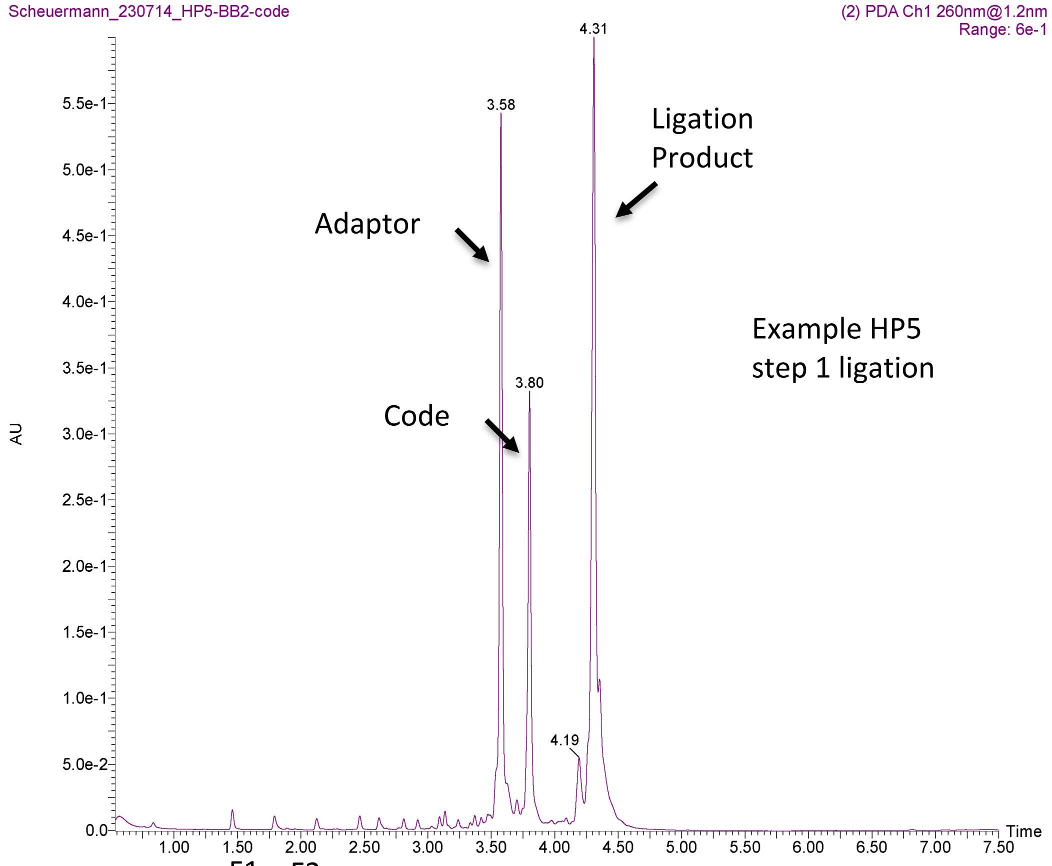
Scheuermann_230714_HP5-BB2-code
(2) PDA Ch1 260nm@1.2nm
Range: 6e-1
Ligation Product
Adaptor
Code
Example HP5 step 1 ligation
3.58
3.80
4.19
4.31
AU
Time

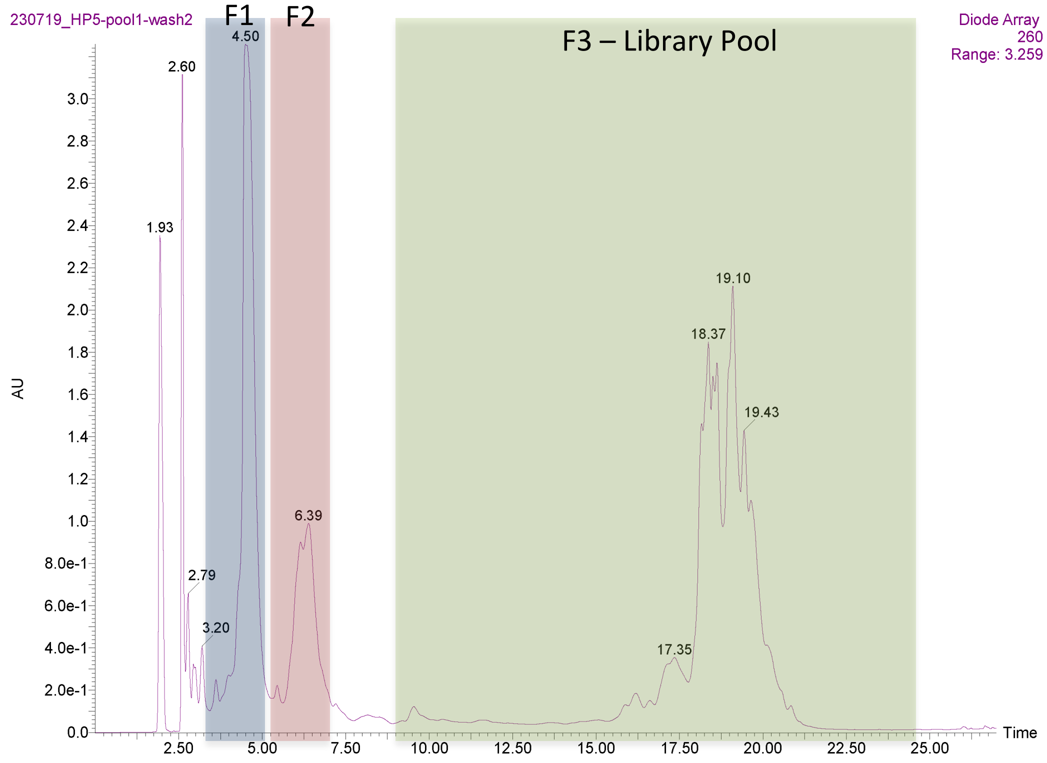
230719_HP5-pool1-wash2
Diode Array
260
Range: 3.259
F1
F2
F3 – Library Pool
1.93
2.60
2.79
3.20
4.50
6.39
17.35
18.37
19.10
19.43
AU
Time

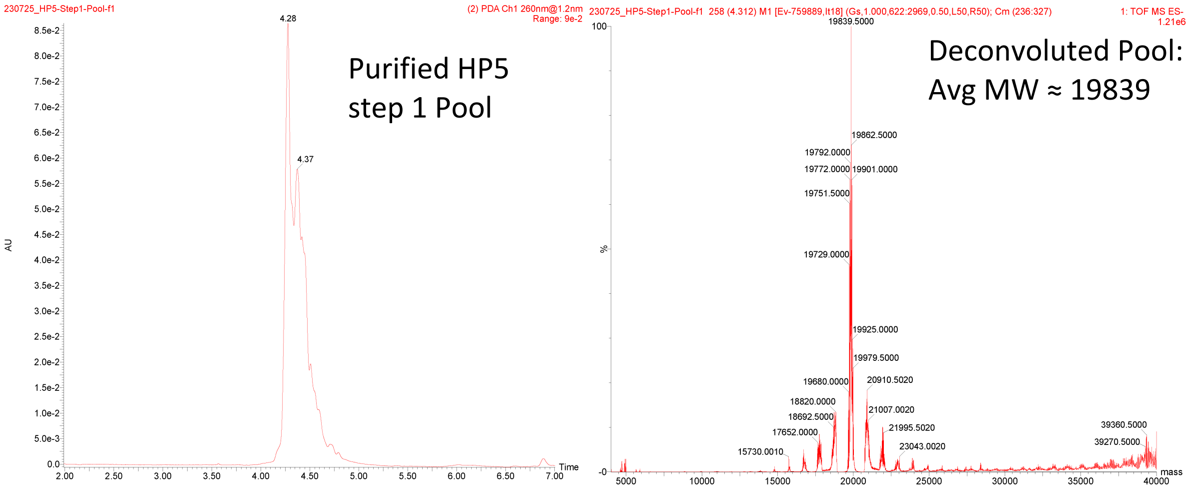

Figure S20: Characterization of HP5 step 1 library synthesis. Top: Example ligation of code 1 onto building block 2 HP5 conjugate. Middle: HPLC purification of library pool after finished ligation. Bottom: UV chromatogram of purified step 1 HP5 pool and MS deconvolution giving an average mass

Table S6: List building blocks 1 and oligonucleotide codons encoding them in the code 1 position

BB #	SMILES	Code (5'-3')
1	C[C@@H](C(=O)O)N	TGGTGT
2	C[C@H](C(=O)O)N	CCTTGA
3	C([C@@H](C(=O)O)N)O	ACGAGT
4	C([C@H](C(=O)O)N)O	AGCTCA
5	C[C@H]([C@@H](C(=O)O)N)O	CTTGGA
6	C[C@@H]([C@H](C(=O)O)N)O	CTCTCT
7	CC(C)C[C@@H](C(=O)O)N	TATAGA
8	CC(C)C[C@H](C(=O)O)N	GTATAA
9	CC(C)[C@@H](C(=O)O)N	CATAAC
10	CC(C)[C@H](C(=O)O)N	CATATG
11	C1=CC=C(C=C1)C[C@@H](C(=O)O)N	TGCGCC
12	C1=CC=C(C=C1)C[C@H](C(=O)O)N	TATGAA
13	C1=CC(=CC=C1C[C@@H](C(=O)O)N)O	AGGCTC
14	C1=CC(=CC=C1C[C@H](C(=O)O)N)O	GTCACG
15	C1=CC=C2C(=C1)C(=CN2)C[C@@H](C(=O)O)N	CGATTG
16	C1=CC=C2C(=C1)C(=CN2)C[C@H](C(=O)O)N	TGAATC
17	CC[C@H](C)[C@@H](C(=O)O)N	CCAGTT
18	CC[C@@H](C)[C@H](C(=O)O)N	AGCTGT
19	NULL	TAGCGA
20	C(C(=O)O)N	GTTATT
21	C([C@@H](C(=O)O)N)C(=O)O	GTACGT
22	C([C@H](C(=O)O)N)C(=O)O	TGCACG
23	C1CCC(CC1)[C@@H](C(=O)O)N	GTGCTA
24	C1CC1C[C@@H](C(=O)O)N	TCGCGC
25	C1CC(C1)C[C@H](C(=O)O)N	TTACTT
26	O=C(O)[C@@H](N)CC1CCCCC1	GTCGTC
27	O=C(O)[C@H](N)CC1CCCCC1	CTGGTC
28	C1CC1[C@@H](C(=O)O)N	CGTTAA
29	CC(C)(C)[C@H](N)C(O)=O	TTGCAC
30	O=C(O)[C@@H](N)CC1=CSC=N1	ACTGCG
31	O=C(O)[C@H](N)CC1=CSC=N1	GTCAGT
32	N[C@@H](CCCNC(N)=O)C(O)=O	TCTATC
33	N[C@H](CCCNC(N)=O)C(O)=O	AGAACT
34	O=C(C(S1)=CC2=C1CNC2)O	ACGTCT
35	N[C@@H](CCCNC(N(C)C)=N)C(O)=O	TTAGCG
36	C1=COC(=C1)C[C@@H](C(=O)O)N	ACACAG
37	CCCC[C@@H](C(=O)O)N	AGTCGA
38	CCCC[C@H](C(=O)O)N	GACAGC
39	C1CCC(CC1)CC[C@@H](C(=O)O)N	CCACCG
40	O=C(O)C(N)CC1=CC(NC2=C1C=CC=C2)=O	GCCGCT
41	CC1=CC(=NO1)NC(=O)CSCC(C(=O)O)N	GACATA
42	CC(=O)NCCCC[C@@H](C(=O)O)N	CATCTT
43	CC(C)(C)C1=CC=C(C=C1)C[C@@H](C(=O)O)N	TCCATT

44	CC(C)(C)C1=CC=C(C=C1)C[C@H](C(=O)O)N	TAGACT
45	C1=CC(=CC(=C1)Cl)CC(C(=O)O)N	ATCTGC
46	C1=CC(=CC=C1C[C@@H](C(=O)O)N)Cl	TATTAC
47	C1=CC(=CC=C1C[C@H](C(=O)O)N)Cl	GCGGAT
48	C1=CC=C(C(=C1)C[C@@H](C(=O)O)N)Cl	AAGTAC
49	O=C(O)[C@@H](N)CC1=CC=C(Cl)C(Cl)=C1	CGGCTA
50	C1=CC=C(C=C1)[C@@H](C(=O)O)N	ATCCAC
51	C1=CC(=CC(=C1)Cl)[C@@H](C(=O)O)N	AAGCAA
52	C1=CC(=CC=C1C[C@H](C(=O)O)N)[N+](=O)[O-]	TTGGTG
53	C1=CC(=CC=C1C[C@@H](C(=O)O)N)C(=O)N	GTAAGA
54	COC1=CC=C(C=C1)C[C@H](C(=O)O)N	AATCTG
55	C1=CC=C2C(=C1)C=CC=C2C[C@@H](C(=O)O)N	CAATAA
56	C1=CC=C(C=C1)C(=O)C2=CC=C(C=C2)C[C@@H](C(=O)O)N	GTATGC
57	C(C1=C(C(=C(C(=C1F)F)F)F)F)[C@@H](C(=O)O)N	GCTACT
58	O=C(O)[C@@H](N)C(C1=CC=CC=C1)C2=CC=CC=C2	CTGCAA
59	C1=CC(=CC=C1C[C@@H](C(=O)O)N)CP(=O)(O)O	AGGTCC
60	C1=CC(=CN=C1)C[C@@H](C(=O)O)N	TTATCC
61	C1=CC(=CN=C1)C[C@H](C(=O)O)N	CAACTA
62	C1=CC=C(C=C1)COC[C@H](C(=O)O)N	GAGCCT
63	C1=CC(=CC=C1CC(C(=O)O)N)C#N	TGTACA
64	O=C(O)[C@@H](N)C/C=C/C1=CC=CC=C1	AGTCAT
65	O=C(O)[C@@H](N)CC1=CC=C(C2=CC=CC=C2)C=C1	ACCGTA
66	O=C(O)C(N)CC1=CC=C(OCO2)C2=C1	CTCAAC
67	O=C(O)[C@@H](N)C1CC2=C(C=CC=C2)C1	AATGCC
68	C1=CC=C(C(=C1)CC(C(=O)O)N)O	GCGTCA
69	O=C(O)[C@@H](N)CC1=CC=C(OCC2=C(Cl)C=CC=C2Cl)C=C1	TATCAG
70	O=C(O)[C@H](CC1=CC=CS1)N	TTCACA
71	C1=C(NC=N1)C[C@@H](C(=O)O)N	TCTTAG
72	C[C@@H](CC(=O)O)N	CGCACT
73	CC(C)C[C@@H](CC(=O)O)N	AGATGA
74	CC(C)[C@H](CC(=O)O)N	GCTAAC
75	C1=CC=C(C=C1)C[C@@H](CC(=O)O)N	GATGAT
76	C(CN)C(=O)O	CCAGAG
77	C1=CC(=CC(=C1)C(F)(F)F)[C@H](CC(=O)O)N	CTTCTC
78	C1=CC(=CC(=C1)C(F)(F)F)[C@@H](CC(=O)O)N	GATTGC
79	C1=CC(=CC=C1C[C@@H](CC(=O)O)N)O	CGCGAC
80	CC1=CC=C(C=C1)C[C@H](CC(=O)O)N	TATTGT
81	N[C@H](CC1=CC=CC=C1Cl)CC(O)=O	CCACAC
82	O=C(O)C[C@H](N)CC1=CC=C(Cl)C(Cl)=C1	CTGCGC
83	C1=CC(=CC=C1C[C@@H](CC(=O)O)N)Cl	CGTCAC
84	N[C@H](CCC(N)=O)CC(O)=O	AGTGTT
85	C1C[C@H](NC1)C(=O)O	CGCTAG
86	C1C[C@@H](NC1)C(=O)O	TCAATG
87	C1CNC[C@H]1C(=O)O	GACTTG
88	O=C([C@H]1NC[C@H](O)C1)O	AATTGA
89	C1CCN[C@@H](C1)C(=O)O	AACCGA
90	C1C[C@H](CNC1)C(=O)O	GTCCGC
91	C1CNCCC1C(=O)O	TGACTG
92	C1CNC(=O)[C@@H](N1)CC(=O)O	CTTGAG
93	O=C(C1=CN(C2CNCC2)N=N1)O	GTGAAT
94	C1CN[C@@H]1C(=O)O	TAACAA
95	O=C([C@H]1CNC[C@@H]1C2=CC=C(Cl)C=C2)O	GCGGTA
96	O=C([C@H]1NCC2=C(C=CC=C2)C1)O	GCGTAG
97	OC(C[C@H]1NCCC1)=O	TGGTCA
98	O=C([C@]12CNC[C@@]1([H])CCO2)O	CTAGCT
99	O=C(C1=CC=NC2=C1CNCC2)O	AGTAGT
100	C1CC2(CNC2)OC1C(=O)O	CCTGTC
101	C1=CC=C(C=C1)CC(C(=O)NCC(=O)O)N	CGTACGC
102	O=C(O)CNC([C@@H](N)CC1=CSC=N1)=O	CGACGA
103	C[C@@H](C(O)=O)NC([C@@H](CC(C)C)N)=O	TGAGAA
104	C[C@@H](C(O)=O)NC([C@H](N)CCCC)=O	ATAGAT
105	C[C@@H](C(O)=O)NC([C@@H](N)CC1=CSC=N1)=O	CTCTTA
106	OC[C@@H](C(O)=O)NC([C@H](N)CC1CCCCC1)=O	ATGCCG
107	C1=CC(=CC=C1C[C@@H](C(=O)N[C@@H](CO)C(=O)O)N)O	TGATCG
108	CC(C)[C@H](C(=O)N[C@@H](CO)C(=O)O)N	AATGGT
109	C[C@@H]([C@@H](C(=O)O)NC(=O)[C@@H]1CCCN1)O	TAGTTC
110	C[C@@H](O)[C@@H](C(O)=O)NC([C@@H]([C@@H](O)C)N)=O	CAGATA

111	C[C@@H](O)[C@@H](C(O)=O)NC([C@@H](N)C1CC2=C(C=CC=C2)C1)=O	AGATAT
112	CC(C)C[C@@H](C(=O)O)NC(=O)[C@H](C(C)C)N	CAATCT
113	CC(C)C[C@@H](C(O)=O)NC([C@H](CCCCNC(C)=O)N)=O	GACTAA
114	C[C@H]([C@@H](C(=O)N[C@@H](C(C)C)C(=O)O)N)O	GAACAC
115	C[C@@H](C(=O)N[C@@H](C(C)C)C(=O)O)N	ATGCGT
116	CC(C)[C@@H](C(O)=O)NC([C@H](N)CC#N)=O	TTCGGC
117	CC(C)[C@@H](C(O)=O)NC([C@@H](N)CC(OC)=O)=O	ACAGGC
118	O=C(O)CNC([C@@H](N)CC(OC)=O)=O	GCGCGA
119	C1=NC(=C2C(=N1)N(C=N2)[C@H]3[C@@H]([C@@H]([C@H](O3)CSCC[C@@H](C(=O)O)N)O)O)N	TCAGAT
120	CC(C)C[C@@H](C(=O)N[C@@H](CC1=CC=CC=C1)C(=O)O)N	TCTGTG
121	OC1=CC=C(C=C1)C[C@@H](C(O)=O)NC([C@@H](C(C)C)N)=O	ATCTAG
122	OC1=CC=C(C=C1)C[C@@H](C(O)=O)NC([C@@H](N)CCCNC(N)=O)=O	GCCTCC
123	OC1=CC=C(C=C1)C[C@@H](C(O)=O)NC([C@H](N)CC2=CC=C(Cl)C=C2)=O	TGCATA
124	C1=CC=C2C(=C1)C(=CN2)C[C@@H](C(=O)O)NC(=O)[C@H](CO)N	ATACTC
125	O=C(O)[C@H](CC1=CNC2=C1C=CC=C2)NC([C@@H](N)CC3=CC=C(Cl)C=C3)=O	GCTCCG
126	CC[C@H](C)[C@@H](C(=O)O)NC(=O)[C@H](CC(=O)N)N	TATACG
127	CC[C@H](C)[C@@H](C(=O)O)NC(=O)[C@H]([C@@H](C)O)N	ACTGAC
128	CC[C@H](C)[C@@H](C(O)=O)NC([C@@H](C)N)=O	TCGAAC
129	C[C@H]([C@@H](C(=O)N[C@@H](CCC(=O)N)C(=O)O)N)O	CACCTG
130	O=C(N)CC[C@@H](C(O)=O)NC([C@H](N)CC#N)=O	ACATTG
131	O=C(N)CC[C@@H](C(O)=O)NC([C@@H](N)CC1CCCCC1)=O	CGACTC
132	CN(CC(=O)O)C(=O)CN	TGCGAG
133	O=C(O)CN(C([C@@H](N)CCCC)=O)C	GACCGT
134	C[C@@H](C(O)=O)N(C([C@H](CC1=CNC2=C1C=CC=C2)N)=O)C	TTAGGA
135	O=C([C@@H]1N(C([C@H](CCCCNC(C)=O)N)=O)CC(F)(F)C1)O	AGCGGC
136	O=C(O)CNC([C@H](CCCNC(N(C)C)=N)N)=O	AGCATC
137	OC1=CC=C(C=C1)C[C@@H](C(O)=O)NC([C@@H](N)CCCC)=O	GAGGTC
138	O=C(O)[C@@H](NC(CN)=O)CC(OC(C)(C)C)=O	GTGTCT
139	C[C@H](C(O)=O)NC([C@@H](CO)N)=O	CTAGTG
140	C[C@H](C(O)=O)NC([C@@H](N)CC1=CC=CN=C1)=O	CGGAGC
141	C[C@H](C(=O)N[C@H](C)C(=O)O)N	GAGTCG
142	OC[C@H](C(O)=O)NC([C@@H](CC(C)C)N)=O	GAAGTG
143	OC[C@H](C(O)=O)NC([C@@H](CC1=CNC2=C1C=CC=C2)N)=O	CGTATA
144	C[C@H]([C@@H](C(=O)O)NC(=O)[C@H](C)N)O	AATACA
145	C[C@H](O)[C@H](C(O)=O)NC([C@@H](N)CCCC)=O	CTCCTT
146	OC[C@H](C(O)=O)NC([C@H](N)CC1=CSC=N1)=O	TCTGCT
147	C[C@H](O)[C@H](C(O)=O)NC(C[C@H](N)C1=CC=CC(C(F)(F)F)=C1)=O	CCTTCG
148	CC(C)C[C@H](C(O)=O)NC([C@@H](CC1=CC=C(O)C=C1)N)=O	GCTCAT
149	CC(C)C[C@H](C(=O)O)NC(=O)CN	CAGGAA
150	CC(C)C[C@H](C(O)=O)NC([C@@H](CC1=CC=CC=C1)N)=O	TGTTGC
151	CC(C)C[C@@H](C(=O)N[C@H](C(C)C)C(=O)O)N	AGCTTG
152	CC(C)[C@H](C(O)=O)NC(C[C@@H](N)C1=CC=CC(C(F)(F)F)=C1)=O	CAGTGT
153	CC(C)[C@H](C(O)=O)NC([C@H](N)CC1=CC=CN=C1)=O	GCGAGC
154	O=C(O)[C@@H](CC1=CC=CC=C1)NC([C@H](N)CCCNC(N)=O)=O	TGCCAA
155	C1=CC=C(C=C1)C[C@@H](C(=O)O)NC(=O)[C@H](CC2=CC=C(C=C2)O)N	GTAGAG
156	OC1=CC=C(C=C1)C[C@H](C(O)=O)NC([C@H](C)N)=O	GAGAAG
157	OC1=CC=C(C=C1)C[C@H](C(O)=O)NC([C@@H]([C@@H](O)C)N)=O	TCTCGA
158	OC1=CC=C(C=C1)C[C@H](C(O)=O)NC([C@H]([C@@H](C)CC)N)=O	ATGGCC
159	O=C(O)[C@@H](CC1=CNC2=C1C=CC=C2)NC([C@H](C(C)C)N)=O	GATAGT
160	C(CC(=O)N)[C@@H](C(=O)O)NC(=O)[C@H](CC(=O)N)N	TACGTT
161	O=C(O)C(C)(NC([C@@H](N)CC1=CC=CC=C1)=O)CO	CCTCTG
162	O=C(O)C(C)(NC([C@@H](N)CC(C)C)=O)CO	CGCTCC
163	O=C(O)C(NC([C@@H](N)CC1=CC=CC=C1)=O)CN2C=CN=C2	TTCTAA
164	O=C(O)C(NC([C@@H](N)CCCNC(N)=O)=O)CN1C=CN=C1	ATGATA
165	O=C(O)[C@H]1N(C([C@@H](CO)N)=O)CCC1	TCGAGA
166	C1C[C@H](NC1)C(=O)N2CCC[C@H]2C(=O)O	CATCAG
167	O=C(O)[C@@H]1N(C([C@@H]2NCCC2)=O)CCC1	GAATCT
168	C1C[C@@H](N(C1)C(=O)CN)C(=O)O	GAACCC
169	[2H]N[C@H](C)C(=O)N1CCC[C@H]1C(=O)O	TGTGAA
170	O=C([C@H]1N(C([C@H](CC2=CC=CC=C2)N)=O)C(C)(C)OC1)O	TGTGGG
171	O=C([C@H]1N(C([C@H](CC(C)C)N)=O)C(C)(C)OC1)O	AAGACC
172	O=C([C@@H]1N(C([C@@H](C(C)C)N)=O)C(C)(C)O[C@H]1C)O	GCGCAT
173	C1C[C@H](N(C1)C(=O)CN)C(=O)O	AGACCT
174	O=C([C@H]1N(C([C@H](C)N)=O)C(C)(C)OC1)O	TTATCT
175	N[C@@H](CCCCNC(C1=CC=C(S(=O)(N)=O)C=C1)=O)C(O)=O	CCGGTT

4.4. Step 1 Headpiece 3' (HP3) Sublibrary

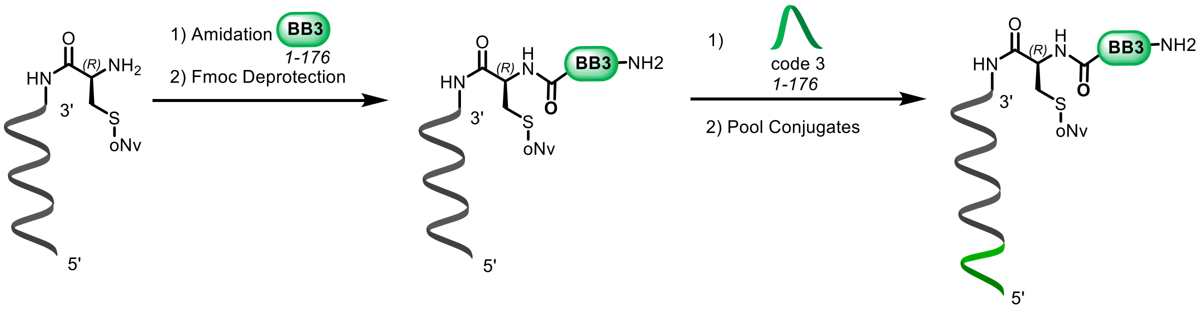

Scaffold modified HP3 DNA was split in 60 nmol portions in 2 mL Eppendorf tubes. 176 amidation reactions were then individually performed for the attachment of all BB3 building blocks. Dipeptide amidations were performed according the optimized EDC/HOAt protocol in section 2.2 and the rest of the amidations were performed using the DMT-MM protocol in the same section. The reaction products were then redissolved and individually Fmoc deprotected. The deprotection was quenched with sodium acetate buffer, followed by the addition of ethanol to precipitate the DNA product. All 176 reaction products were then individually HPLC-purified, precipitated and their yield was measured. 18 nmol for each HPLC-purified conjugate was taken for encoding step.

Each conjugate was individually encoded using code 3 strands according to procedure in section 2.2. After confirmation of ligation completion via LC-MS all 176 encoded conjugates were pooled and precipitated. The conjugate pool was then purified via HPLC at 60 °C, which enabled separation from excess codes and adaptor left over from the encoding step. The purified pool was precipitated again and analyzed. This afforded a final step 1 HP3 pool of 2240 nmol.

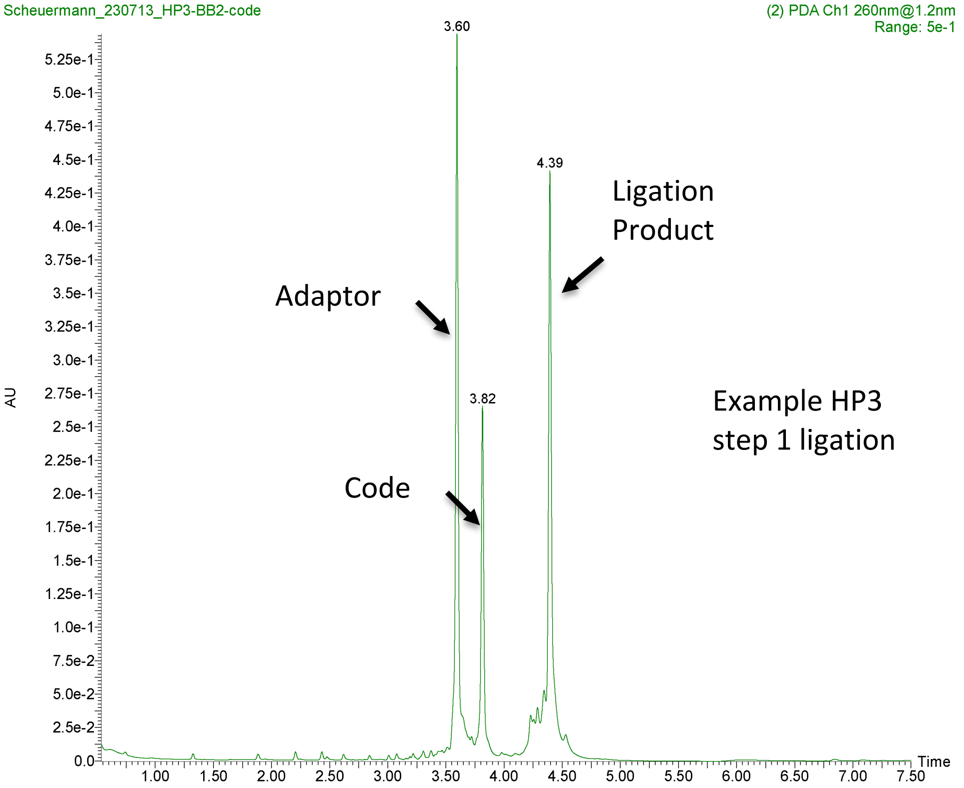

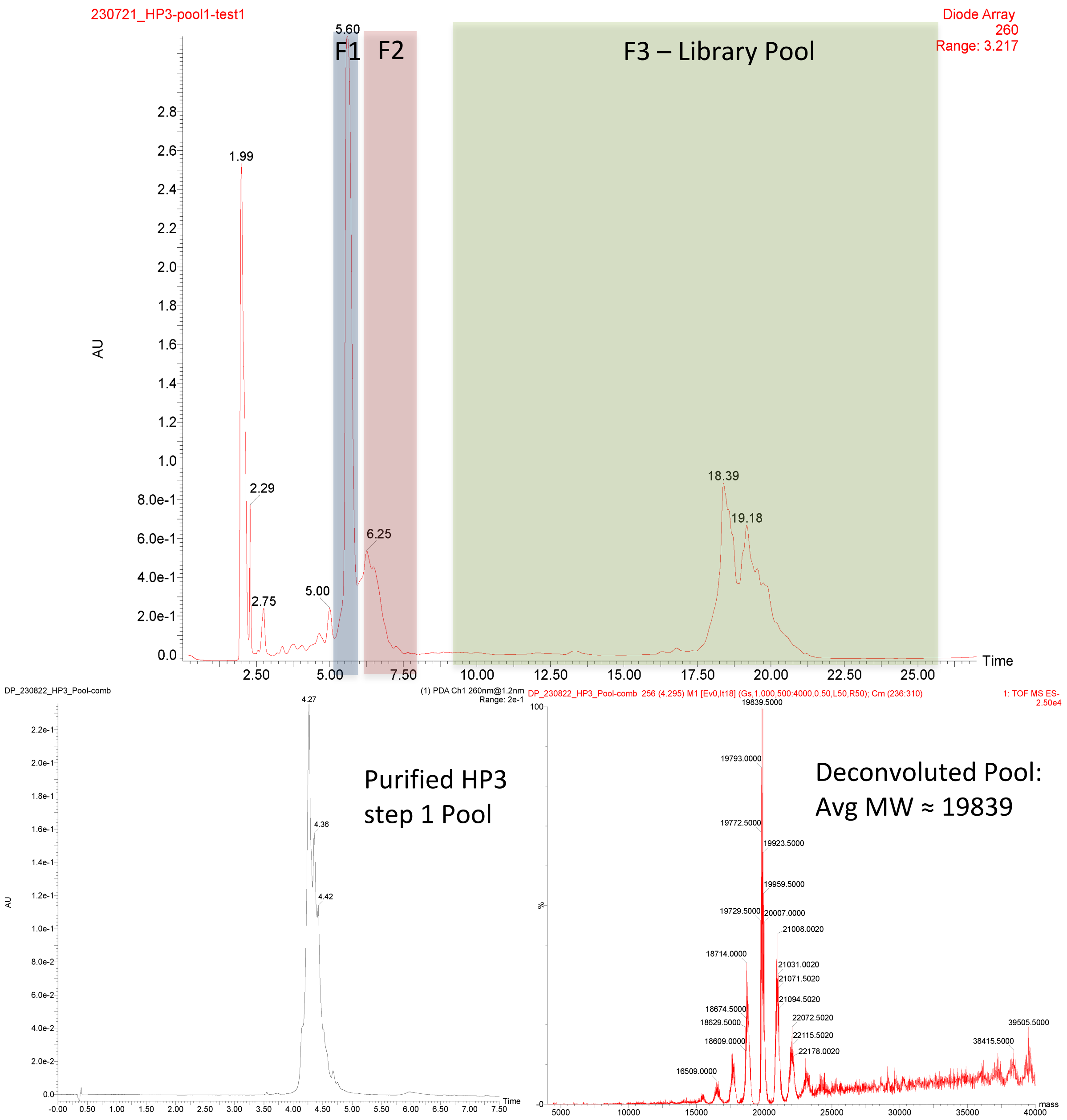

Figure S21: Characterization of HP3 step 1 library synthesis. Top: Example ligation of code 3 onto building block 2 HP3 conjugate. Middle: HPLC purification of library pool after finished ligation. Bottom: UV chromatogram of purified step 1 HP3 pool and MS deconvolution giving an average mass

Table S7: List building blocks 3 and oligonucleotide codons encoding them in the code 3 position

BB #	SMILES	Code (5'-3')
1	C[C@@H](C(=O)O)N	TGGTGT
2	C[C@H](C(=O)O)N	CCTTGA
3	C([C@@H](C(=O)O)N)O	ACGAGT
4	C([C@H](C(=O)O)N)O	AGCTCA
5	C[C@H]([C@@H](C(=O)O)N)O	CTTGGA
6	C[C@@H]([C@H](C(=O)O)N)O	CTCTCT
7	CC(C)C[C@@H](C(=O)O)N	TATAGA
8	CC(C)C[C@H](C(=O)O)N	GTATAA
9	CC(C)[C@@H](C(=O)O)N	CATAAC
10	CC(C)[C@H](C(=O)O)N	CATATG
11	C1=CC=C(C=C1)C[C@@H](C(=O)O)N	TGCGCC
12	C1=CC=C(C=C1)C[C@H](C(=O)O)N	TATGAA
13	C1=CC(=CC=C1C[C@@H](C(=O)O)N)O	AGGCTC

14	C1=CC(=CC=C1C[C@H](C(=O)O)N)O	GTCACG
15	C1=CC=C2C(=C1)C(=CN2)C[C@@H](C(=O)O)N	CGATTG
16	C1=CC=C2C(=C1)C(=CN2)C[C@H](C(=O)O)N	TGAATC
17	CC[C@H](C)[C@@H](C(=O)O)N	CCAGTT
18	CC[C@@H](C)[C@H](C(=O)O)N	AGCTGT
19	O=C([C@H]1[C@@H](CN)OCC1)O	AAGACC
20	NULL	TAGCGA
21	C(C(=O)O)N	GTTATT
22	C([C@@H](C(=O)O)N)C(=O)O	GTACGT
23	C([C@H](C(=O)O)N)C(=O)O	TGCACG
24	C1CCC(CC1)[C@@H](C(=O)O)N	GTGCTA
25	C1CC1C[C@@H](C(=O)O)N	TCGCGC
26	C1CC(C1)C[C@H](C(=O)O)N	TTACTT
27	O=C(O)[C@@H](N)CC1CCCCC1	GTCGTC
28	O=C(O)[C@H](N)CC1CCCCC1	CTGGTC
29	C1CC1[C@@H](C(=O)O)N	CGTTAA
30	CC(C)(C)[C@H](N)C(O)=O	TTGCAC
31	O=C(O)[C@@H](N)CC1=CSC=N1	ACTGCG
32	O=C(O)[C@H](N)CC1=CSC=N1	GTCAGT
33	N[C@@H](CCCNC(N)=O)C(O)=O	TCTATC
34	N[C@H](CCCNC(N)=O)C(O)=O	AGAACT
35	O=C(C(S1)=CC2=C1CNC2)O	ACGTCT
36	N[C@@H](CCCNC(N(C)C)=N)C(O)=O	TTAGCG
37	C1=COC(=C1)C[C@@H](C(=O)O)N	ACACAG
38	CCCC[C@@H](C(=O)O)N	AGTCGA
39	CCCC[C@H](C(=O)O)N	GACAGC
40	C1CCC(CC1)CC[C@@H](C(=O)O)N	CCACCG
41	O=C(O)C(N)CC1=CC(NC2=C1C=CC=C2)=O	GCCGCT
42	CC1=CC(=NO1)NC(=O)CSCC(C(=O)O)N	GACATA
43	CC(=O)NCCCC[C@@H](C(=O)O)N	CATCTT
44	CC(C)(C)C1=CC=C(C=C1)C[C@@H](C(=O)O)N	TCCATT
45	CC(C)(C)C1=CC=C(C=C1)C[C@H](C(=O)O)N	TAGACT
46	C1=CC(=CC(=C1)Cl)CC(C(=O)O)N	ATCTGC
47	C1=CC(=CC=C1C[C@@H](C(=O)O)N)Cl	TATTAC
48	C1=CC(=CC=C1C[C@H](C(=O)O)N)Cl	GCGGAT
49	C1=CC=C(C(=C1)C[C@@H](C(=O)O)N)Cl	AAGTAC
50	O=C(O)[C@@H](N)CC1=CC=C(Cl)C(Cl)=C1	CGGCTA
51	C1=CC=C(C=C1)[C@@H](C(=O)O)N	ATCCAC
52	C1=CC(=CC(=C1)Cl)[C@@H](C(=O)O)N	AAGCAA
53	C1=CC(=CC=C1C[C@H](C(=O)O)N)[N+](=O)[O-]	TTGGTG
54	C1=CC(=CC=C1C[C@@H](C(=O)O)N)C(=O)N	GTAAGA
55	COC1=CC=C(C=C1)C[C@H](C(=O)O)N	AATCTG
56	C1=CC=C2C(=C1)C=CC=C2C[C@@H](C(=O)O)N	CAATAA
57	C1=CC=C(C=C1)C(=O)C2=CC=C(C=C2)C[C@@H](C(=O)O)N	GTATGC
58	C(C1=C(C(=C(C(=C1F)F)F)F)F)[C@@H](C(=O)O)N	GCTACT
59	O=C(O)[C@@H](N)C(C1=CC=CC=C1)C2=CC=CC=C2	CTGCAA
60	C1=CC(=CC=C1C[C@@H](C(=O)O)N)CP(=O)(O)O	AGGTCC
61	C1=CC(=CN=C1)C[C@@H](C(=O)O)N	TTATCC
62	C1=CC(=CN=C1)C[C@H](C(=O)O)N	CAACTA
63	C1=CC=C(C=C1)COC[C@H](C(=O)O)N	GAGCCT
64	C1=CC(=CC=C1CC(C(=O)O)N)C#N	TGTACA
65	O=C(O)[C@@H](N)C/C=C/C1=CC=CC=C1	AGTCAT
66	O=C(O)[C@@H](N)CC1=CC=C(C2=CC=CC=C2)C=C1	ACCGTA
67	O=C(O)C(N)CC1=CC=C(OCO2)C2=C1	CTCAAC
68	O=C(O)[C@@H](N)C1CC2=C(C=CC=C2)C1	AATGCC
69	C1=CC=C(C(=C1)CC(C(=O)O)N)O	GCGTCA
70	O=C(O)[C@@H](N)CC1=CC=C(OCC2=C(Cl)C=CC=C2Cl)C=C1	TATCAG
71	O=C(O)[C@H](CC1=CC=CS1)N	TTCACA
72	C1=C(NC=N1)C[C@@H](C(=O)O)N	TCTTAG
73	C[C@@H](CC(=O)O)N	CGCACT
74	CC(C)C[C@@H](CC(=O)O)N	AGATGA
75	CC(C)[C@H](CC(=O)O)N	GCTAAC
76	C1=CC=C(C=C1)C[C@@H](CC(=O)O)N	GATGAT
77	C(CN)C(=O)O	CCAGAG
78	C1=CC(=CC(=C1)C(F)(F)F)[C@H](CC(=O)O)N	CTTCTC
79	C1=CC(=CC(=C1)C(F)(F)F)[C@@H](CC(=O)O)N	GATTGC
80	C1=CC(=CC=C1C[C@@H](CC(=O)O)N)O	CGCGAC

81	CC1=CC=C(C=C1)C[C@H](CC(=O)O)N	TATTGT
82	N[C@H](CC1=CC=CC=C1Cl)CC(O)=O	CCACAC
83	O=C(O)C[C@H](N)CC1=CC=C(Cl)C(Cl)=C1	CTGCGC
84	C1=CC(=CC=C1C[C@@H](CC(=O)O)N)Cl	CGTCAC
85	N[C@H](CCC(N)=O)CC(O)=O	AGTGTT
86	C1C[C@H](NC1)C(=O)O	CGCTAG
87	C1C[C@@H](NC1)C(=O)O	TCAATG
88	C1CNC[C@H]1C(=O)O	GACTTG
89	O=C([C@H]1NC[C@H](O)C1)O	AATTGA
90	C1CCN[C@@H](C1)C(=O)O	AACCGA
91	C1C[C@H](CNC1)C(=O)O	GTCCGC
92	C1CNCCC1C(=O)O	TGACTG
93	C1CNC(=O)[C@@H](N1)CC(=O)O	CTTGAG
94	O=C(C1=CN(C2CNCC2)N=N1)O	GTGAAT
95	C1CN[C@@H]1C(=O)O	TAACAA
96	O=C([C@H]1CNC[C@@H]1C2=CC=C(Cl)C=C2)O	GCGGTA
97	O=C([C@H]1NCC2=C(C=CC=C2)C1)O	GCGTAG
98	OC(C[C@H]1NCCC1)=O	TGGTCA
99	O=C([C@]12CNC[C@@]1([H])CCO2)O	CTAGCT
100	O=C(C1=CC=NC2=C1CNCC2)O	AGTAGT
101	C1CC2(CNC2)OC1C(=O)O	CCTGTC
102	C1=CC=C(C=C1)CC(C(=O)NCC(=O)O)N	CGTACG
103	O=C(O)CNC([C@@H](N)CC1=CSC=N1)=O	CGACGA
104	O=C(C1=NOC2(CCNCC2)C1)O	CGCGCG
105	C[C@@H](C(O)=O)NC([C@@H](CC(C)C)N)=O	TGAGAA
106	C[C@@H](C(O)=O)NC([C@H](N)CCCC)=O	ATAGAT
107	C[C@@H](C(O)=O)NC([C@@H](N)CC1=CSC=N1)=O	CTCTTA
108	OC[C@@H](C(O)=O)NC([C@H](N)CC1CCCCC1)=O	ATGCCG
109	C1=CC(=CC=C1C[C@@H](C(=O)N[C@@H](CO)C(=O)O)N)O	TGATCG
110	CC(C)[C@H](C(=O)N[C@@H](CO)C(=O)O)N	AATGGT
111	C[C@@H]([C@@H](C(=O)O)NC(=O)[C@@H]1CCCN1)O	TAGTTC
112	C[C@@H](O)[C@@H](C(O)=O)NC([C@@H]([C@@H](O)C)N)=O	CAGATA
113	C[C@@H](O)[C@@H](C(O)=O)NC([C@@H](N)C1CC2=C(C=CC=C2)C1)=O	AGATAT
114	CC(C)C[C@@H](C(=O)O)NC(=O)[C@H](C(C)C)N	CAATCT
115	CC(C)C[C@@H](C(O)=O)NC([C@H](CCCCNC(C)=O)N)=O	GACTAA
116	C[C@H]([C@@H](C(=O)N[C@@H](C(C)C)C(=O)O)N)O	GAACAC
117	C[C@@H](C(=O)N[C@@H](C(C)C)C(=O)O)N	ATGCGT
118	CC(C)[C@@H](C(O)=O)NC([C@@H](N)CC(OC)=O)=O	ACAGGC
119	O=C(O)CNC([C@@H](N)CC(OC)=O)=O	GCGCGA
120	O=C(O)[C@H](CC1=CC=CC=C1)NC([C@@H](N)C2CC3=C(C=CC=C3)C2)=O	TCAGAT
121	CC(C)C[C@@H](C(=O)N[C@@H](CC1=CC=CC=C1)C(=O)O)N	TCTGTG
122	OC1=CC=C(C=C1)C[C@@H](C(O)=O)NC([C@@H](C(C)C)N)=O	ATCTAG
123	OC1=CC=C(C=C1)C[C@@H](C(O)=O)NC([C@@H](N)CCCNC(N)=O)=O	GCCTCC
124	OC1=CC=C(C=C1)C[C@@H](C(O)=O)NC([C@H](N)CC2=CC=C(Cl)C=C2)=O	TGCATA
125	C1=CC=C2C(=C1)C(=CN2)C[C@@H](C(=O)O)NC(=O)[C@H](CO)N	ATACTC
126	O=C(O)[C@H](CC1=CNC2=C1C=CC=C2)NC([C@@H](N)CC3=CC=C(Cl)C=C3)=O	GCTCCG
127	CC[C@H](C)[C@@H](C(=O)O)NC(=O)[C@H](CC(=O)N)N	TATACG
128	CC[C@H](C)[C@@H](C(=O)O)NC(=O)[C@H]([C@@H](C)O)N	ACTGAC
129	CC[C@H](C)[C@@H](C(O)=O)NC([C@@H](C)N)=O	TCGAAC
130	C[C@H]([C@@H](C(=O)N[C@@H](CCC(=O)N)C(=O)O)N)O	CACCTG
131	O=C(N)CC[C@@H](C(O)=O)NC([C@H](N)CC#N)=O	ACATTG
132	O=C(N)CC[C@@H](C(O)=O)NC([C@@H](N)CC1CCCCC1)=O	CGACTC
133	CN(CC(=O)O)C(=O)CN	TGCGAG
134	O=C(O)CN(C([C@@H](N)CCCC)=O)C	GACCGT
135	C[C@@H](C(O)=O)N(C([C@H](CC1=CNC2=C1C=CC=C2)N)=O)C	TTAGGA
136	O=C([C@@H]1N(C([C@H](CCCCNC(C)=O)N)=O)CC(F)(F)C1)O	AGCGGC
137	C1=NC(=C2C(=N1)N(C=N2)[C@H]3[C@@H]([C@@H]([C@H](O3)CSCC[C@@H](C(=O)O)N)O)O)N	AGCATC
138	OC1=CC=C(C=C1)C[C@@H](C(O)=O)NC([C@@H](N)CCCC)=O	GAGGTC
139	O=C(O)[C@@H](NC(CN)=O)CC(OC(C)(C)C)=O	GTGTCT
140	C[C@H](C(O)=O)NC([C@@H](CO)N)=O	CTAGTG
141	C[C@H](C(O)=O)NC([C@@H](N)CC1=CC=CN=C1)=O	CGGAGC
142	C[C@H](C(=O)N[C@H](C)C(=O)O)N	GAGTCG
143	OC[C@H](C(O)=O)NC([C@@H](CC(C)C)N)=O	GAAGTG
144	OC[C@H](C(O)=O)NC([C@@H](CC1=CNC2=C1C=CC=C2)N)=O	CGTATA
145	C[C@H]([C@@H](C(=O)O)NC(=O)[C@H](C)N)O	AATACA
146	C[C@H](O)[C@H](C(O)=O)NC([C@@H](N)CCCC)=O	CTCCTT
147	OC[C@H](C(O)=O)NC([C@H](N)CC1=CSC=N1)=O	TCTGCT

148	C[C@H](O)[C@H](C(O)=O)NC(C[C@H](N)C1=CC=CC(C(F)(F)F)=C1)=O	CCTTCG
149	CC(C)C[C@H](C(O)=O)NC([C@@H](CC1=CC=C(O)C=C1)N)=O	GCTCAT
150	CC(C)C[C@H](C(=O)O)NC(=O)CN	CAGGAA
151	CC(C)C[C@H](C(O)=O)NC([C@@H](CC1=CC=CC=C1)N)=O	TGTTGC
152	CC(C)C[C@@H](C(=O)N[C@H](C(C)C)C(=O)O)N	AGCTTG
153	CC(C)[C@H](C(O)=O)NC(C[C@@H](N)C1=CC=CC(C(F)(F)F)=C1)=O	CAGTGT
154	CC(C)[C@H](C(O)=O)NC([C@H](N)CC1=CC=CN=C1)=O	GCGAGC
155	O=C(O)[C@@H](CC1=CC=CC=C1)NC([C@H](N)CCCNC(N)=O)=O	TGCCAA
156	C1=CC=C(C=C1)C[C@@H](C(=O)O)NC(=O)[C@H](CC2=CC=C(C=C2)O)N	GTAGAG
157	OC1=CC=C(C=C1)C[C@H](C(O)=O)NC([C@H](C)N)=O	GAGAAG
158	OC1=CC=C(C=C1)C[C@H](C(O)=O)NC([C@@H]([C@@H](O)C)N)=O	TCTCGA
159	OC1=CC=C(C=C1)C[C@H](C(O)=O)NC([C@H]([C@@H](C)CC)N)=O	ATGGCC
160	O=C(O)[C@@H](CC1=CNC2=C1C=CC=C2)NC([C@H](C(C)C)N)=O	GATAGT
161	C(CC(=O)N)[C@@H](C(=O)O)NC(=O)[C@H](CC(=O)N)N	TACGTT
162	O=C(O)C(C)(NC([C@@H](N)CC1=CC=CC=C1)=O)CO	CCTCTG
163	O=C(O)C(C)(NC([C@@H](N)CC(C)C)=O)CO	CGCTCC
164	O=C(O)C(NC([C@@H](N)CC1=CC=CC=C1)=O)CN2C=CN=C2	TTCTAA
165	O=C(O)C(NC([C@@H](N)CCCNC(N)=O)=O)CN1C=CN=C1	ATGATA
166	O=C(O)[C@H]1N(C([C@@H](CO)N)=O)CCC1	TCGAGA
167	C1C[C@H](NC1)C(=O)N2CCC[C@H]2C(=O)O	CCTACA
168	O=C(O)[C@@H]1N(C([C@@H]2NCCC2)=O)CCC1	CTGCTC
169	C1C[C@@H](N(C1)C(=O)CN)C(=O)O	GAAGTA
170	[2H]N[C@H](C)C(=O)N1CCC[C@H]1C(=O)O	AATATG
171	O=C([C@H]1N(C([C@H](CC2=CC=CC=C2)N)=O)C(C)(C)OC1)O	GGAAGC
172	O=C([C@H]1N(C([C@H](CC(C)C)N)=O)C(C)(C)OC1)O	GCGCGG
173	O=C([C@@H]1N(C([C@@H](C(C)C)N)=O)C(C)(C)O[C@H]1C)O	CCTGGC
174	C1C[C@H](N(C1)C(=O)CN)C(=O)O	CCGAGG
175	O=C([C@H]1N(C([C@H](C)N)=O)C(C)(C)OC1)O	CGTTCC
176	N[C@@H](CCCCNC(C1=CC=C(S(=O)(N)=O)C=C1)=O)C(O)=O	GCGCCG

4.5. Step 2 Headpiece 5' (HP5) Sublibrary

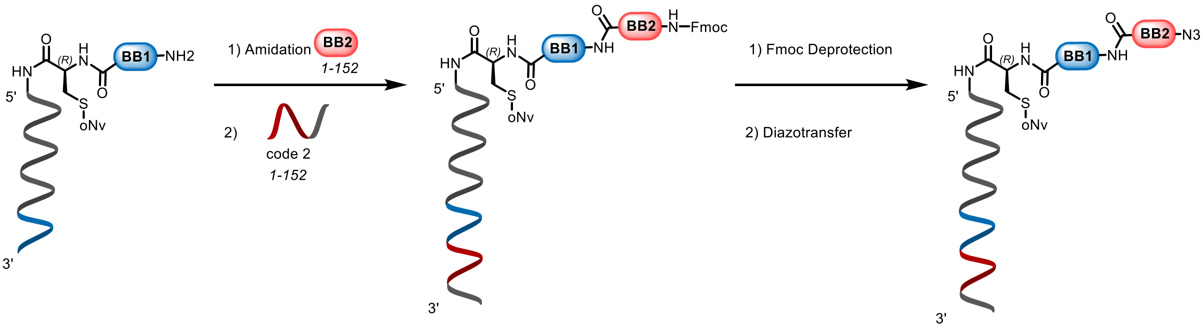

Purified step 1 HP5 pool was split in 19 nmol portions 152 times in 2 mL Eppendorf tubes. Step 2 amidations were performed according to general protocols in section 2.2 (volumes not scaled down due to less starting material). After reaction precipitation, all pool conjugates were ligated with code 2 strands according to general procedure. Ligation progress was checked via LC-MS. After successful ligation, all conjugates were repooled and precipitated. The pellets were redissolved and stored in a -20 °C freezer.

20% of the resulting product was transferred to a new 15 mL falcon tube and subjected to Fmoc deprotection. The deprotection was quenched with sodium acetate and precipitated with ethanol. The product was then split into 10 2 mL Eppendorf tubes and diazotransfer was carried out according to general conditions. The azido-modified product was then HPLC-purified at 60 °C to remove excess codes/adaptor, precipitated and analyzed. This afforded a final yield of 350 nmol of HP5 step 2 pool.

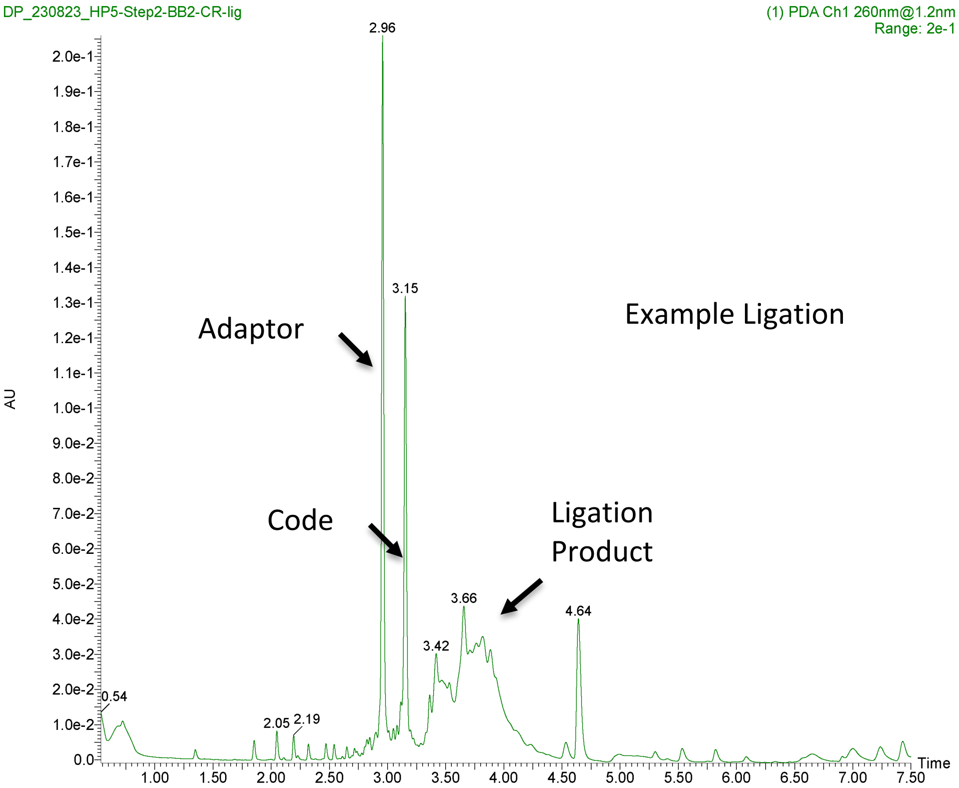
DP_230823_HP5-Step2-BB2-CR-lig
(1) PDA Ch1 260nm@1.2nm
Range: 2e-1
AU
Time
Example Ligation
Adaptor
Code
Ligation Product
2.96
3.15
3.42
3.66
4.64
0.54
2.05
2.19

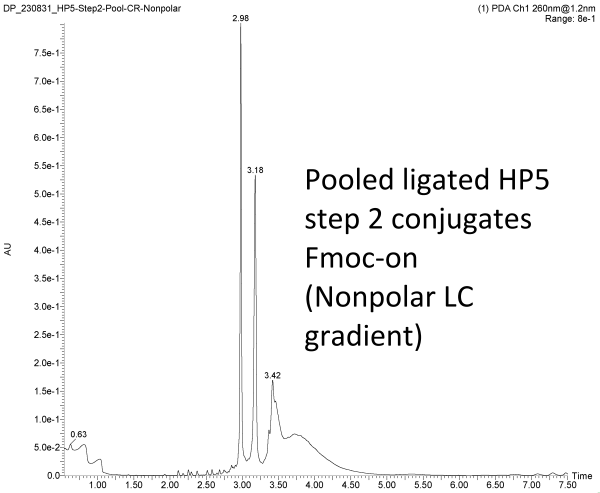
DP_230831_HP5-Step2-Pool-CR-Nonpolar
(1) PDA Ch1 260nm@1.2nm
Range: 8e-1
AU
Time
Pooled ligated HP5 step 2 conjugates Fmoc-on (Nonpolar LC gradient)
2.98
3.18
3.42
0.63

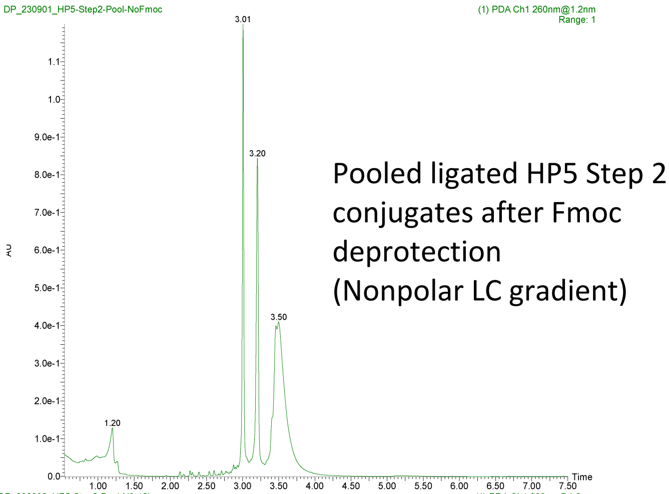
DP_230901_HP5-Step2-Pool-NoFmoc
(1) PDA Ch1 260nm@1.2nm
Range: 1
AU
Time
Pooled ligated HP5 Step 2 conjugates after Fmoc deprotection (Nonpolar LC gradient)
3.01
3.20
3.50
1.20

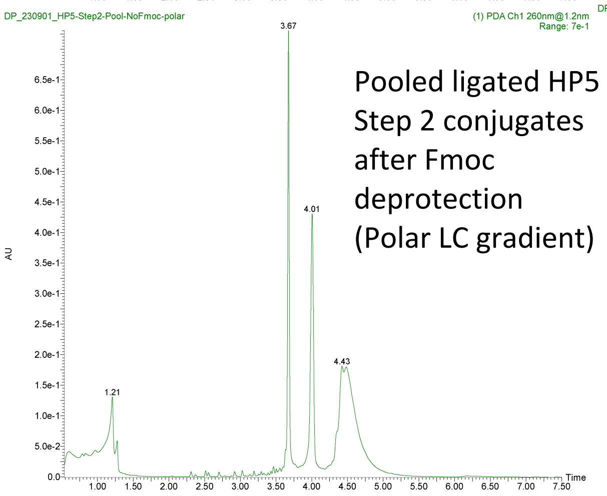
DP_230901_HP5-Step2-Pool-NoFmoc-polar
(1) PDA Ch1 260nm@1.2nm
Range: 7e-1
AU
Time
Pooled ligated HP5 Step 2 conjugates after Fmoc deprotection (Polar LC gradient)
3.67
4.01
4.43
1.21

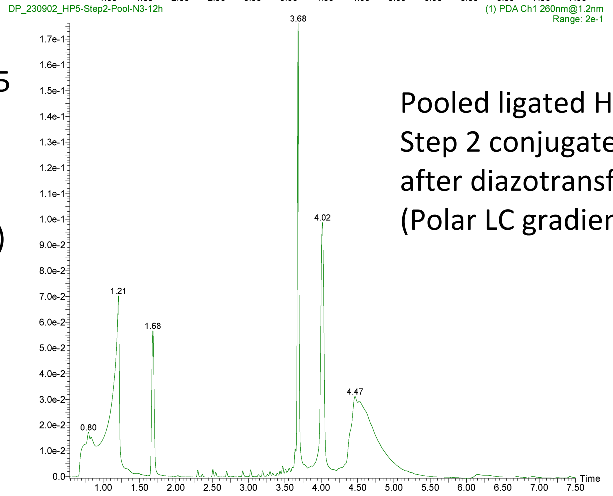
DP_230902_HP5-Step2-Pool-N3-12h
(1) PDA Ch1 260nm@1.2nm
Range: 2e-1
Time
Pooled ligated HP5 Step 2 conjugates after diazotransfer (Polar LC gradient)
3.68
4.02
4.47
1.21
1.68
0.80

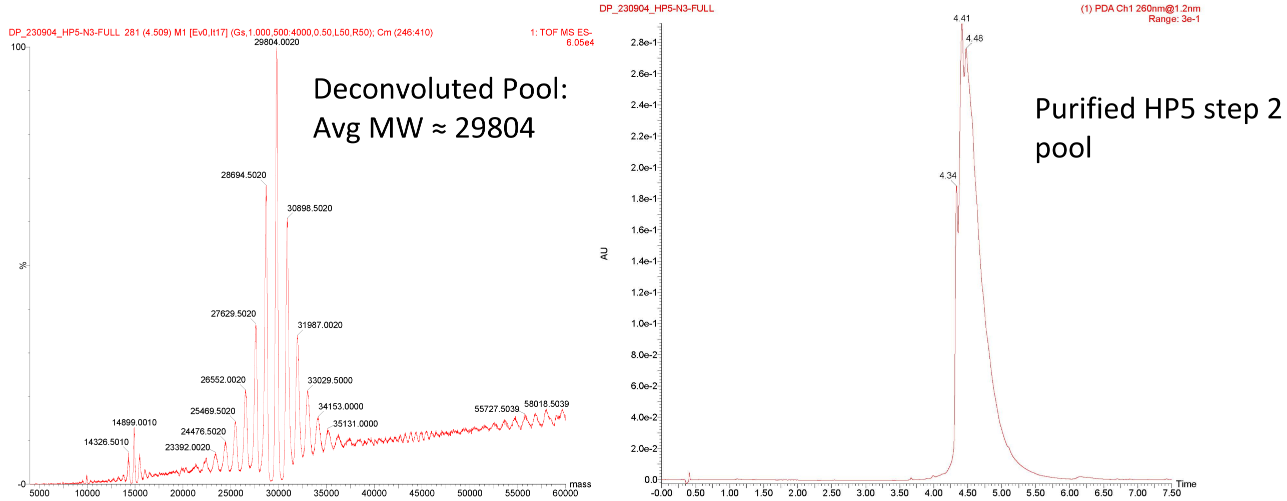

Figure S22: Characterization of HP5 step 2 library synthesis. Top: Example ligation of code 2 onto building block 2 HP5 pool 1 conjugates. Middle: Characterization of reaction steps performed on HP5 step 2 pool. Bottom: UV 260 nm of purified HP5 step 2 pool and average deconvoluted mass

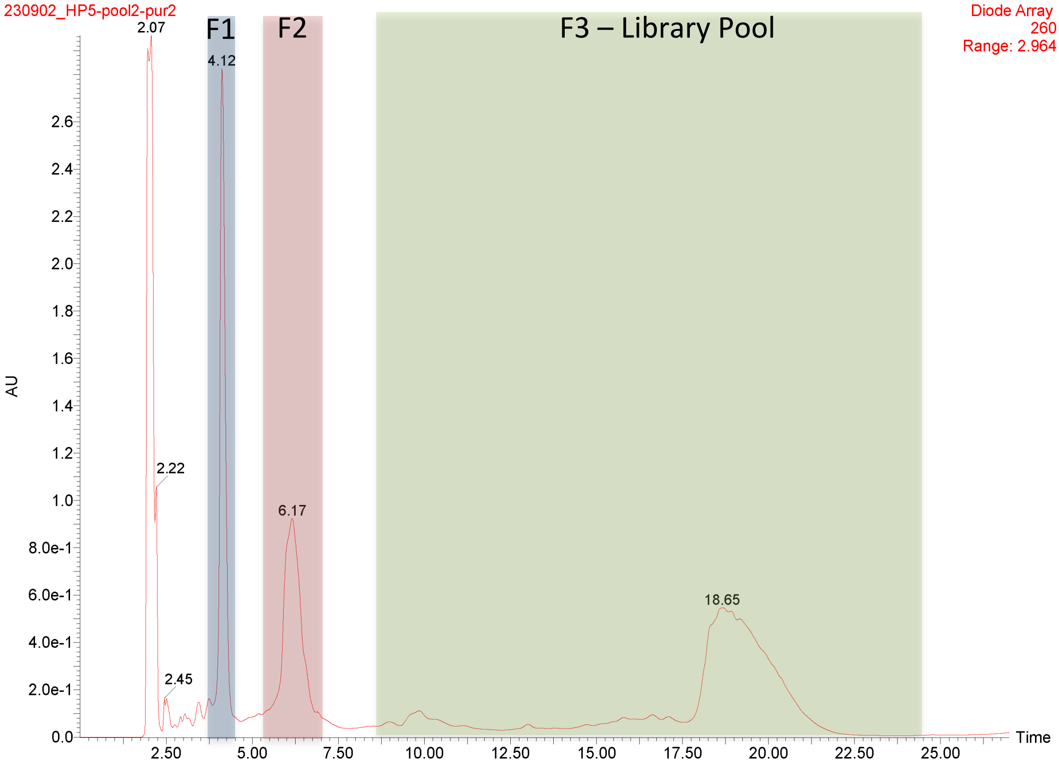

Figure S23: HPLC purification of HP5 step 2 library pool after finished ligation

Table S8: List building blocks 2 and oligonucleotide codons encoding them in the code 2 position

BB #	SMILES	Code (5'-3')
1	C[C@@H](C(=O)O)N	GTCTTCA
2	C[C@H](C(=O)O)N	CGCAGAG
3	C([C@@H](C(=O)O)N)O	GCTTGAA
4	C([C@H](C(=O)O)N)O	ATAACTA
5	C[C@H]([C@@H](C(=O)O)N)O	GTAGGCC
6	C[C@@H]([C@H](C(=O)O)N)O	CCAGCTC
7	CC(C)C[C@@H](C(=O)O)N	TGTTCTC
8	CC(C)C[C@H](C(=O)O)N	TTGATCG
9	CC(C)[C@@H](C(=O)O)N	AAGTTGA
10	CC(C)[C@H](C(=O)O)N	GTCGAGT
11	C1=CC=C(C=C1)C[C@@H](C(=O)O)N	CATTAGC
12	C1=CC=C(C=C1)C[C@H](C(=O)O)N	ATGTCCA
13	C1=CC(=CC=C1C[C@@H](C(=O)O)N)O	TCTTAAT

14	C1=CC(=CC=C1C[C@H](C(=O)O)N)O	GATACAC
15	C1=CC=C2C(=C1)C(=CN2)C[C@@H](C(=O)O)N	GAATCAG
16	C1=CC=C2C(=C1)C(=CN2)C[C@H](C(=O)O)N	AGCGAAG
17	CC[C@H](C)[C@@H](C(=O)O)N	ATTCCAA
18	CC[C@@H](C)[C@H](C(=O)O)N	TCTGCGA
19	C(CC(=O)N)[C@@H](C(=O)O)N	GCGCATA
20	C(CC(=O)N)[C@H](C(=O)O)N	TTGACAC
21	C(C(=O)O)N	CGTGGTT
22	C([C@@H](C(=O)O)N)C(=O)O	AACTGCC
23	C([C@H](C(=O)O)N)C(=O)O	CAGGCTG
24	C1CCC(CC1)[C@@H](C(=O)O)N	TTCACGA
25	C1CC1C[C@@H](C(=O)O)N	GTGAACG
26	C1CC(C1)C[C@H](C(=O)O)N	AAGTTAT
27	O=C(O)[C@@H](N)CC1CCCCC1	AGCACTG
28	O=C(O)[C@H](N)CC1CCCCC1	TTCGGAG
29	C1CC1[C@@H](C(=O)O)N	TTGCGGT
30	O=C(O)[C@@H](N)CC1=CSC=N1	GATTACC
31	O=C(O)[C@H](N)CC1=CSC=N1	CGACTAC
32	N[C@@H](CCCNC(N)=O)C(O)=O	CTAGACC
33	N[C@H](CCCNC(N)=O)C(O)=O	ATGCTCT
34	C=CC[C@H](CC(=O)O)N	CGTCATA
35	N[C@@H](CCCNC(N(C)C)=N)C(O)=O	CACTCTT
36	C1=COC(=C1)C[C@@H](C(=O)O)N	CACAATA
37	CCCC[C@@H](C(=O)O)N	TCGATAT
38	CCCC[C@H](C(=O)O)N	CCGCCGC
39	C1CCC(CC1)CC[C@@H](C(=O)O)N	TTAACAG
40	O=C(O)C(N)CC1=CC(NC2=C1C=CC=C2)=O	GCTGGTT
41	CC(=O)NCCCC[C@@H](C(=O)O)N	GCAGCCG
42	CC(C)(C)C1=CC=C(C=C1)C[C@@H](C(=O)O)N	GAACCAT
43	CC(C)(C)C1=CC=C(C=C1)C[C@H](C(=O)O)N	TTCCTAT
44	C1=CC(=CC(=C1)Cl)CC(C(=O)O)N	AGTATTC
45	C1=CC(=CC=C1C[C@@H](C(=O)O)N)Cl	GAGATAC
46	C1=CC(=CC=C1C[C@H](C(=O)O)N)Cl	GAAGGAC
47	C1=CC=C(C(=C1)C[C@@H](C(=O)O)N)Cl	GTAATCA
48	O=C(O)[C@@H](N)CC1=CC=C(Cl)C(Cl)=C1	CATCCTA
49	C1=CC=C(C=C1)[C@@H](C(=O)O)N	CTATACA
50	C1=CC(=CC(=C1)Cl)[C@@H](C(=O)O)N	GAACGAG
51	C1=CC(=CC=C1C[C@H](C(=O)O)N)[N+](=O)[O-]	CAATCGT
52	C1=CC(=CC=C1C[C@@H](C(=O)O)N)C(=O)N	CACAGGC
53	COC1=CC=C(C=C1)C[C@H](C(=O)O)N	ACAGGCA
54	C1=CC=C2C(=C1)C=CC=C2C[C@@H](C(=O)O)N	AGAGTGC
55	C1=CC=C(C=C1)C(=O)C2=CC=C(C=C2)C[C@@H](C(=O)O)N	ACTTGCG
56	C(C1=C(C(=C(C(=C1F)F)F)F)F)[C@@H](C(=O)O)N	CAAGTCT
57	O=C(O)[C@@H](N)C(C1=CC=CC=C1)C2=CC=CC=C2	AATCAGT
58	C1=CC(=CC=C1C[C@@H](C(=O)O)N)CP(=O)(O)O	CCATTAC
59	C1=CC(=CN=C1)C[C@@H](C(=O)O)N	TGATAAG
60	C1=CC(=CN=C1)C[C@H](C(=O)O)N	TAGAGTT
61	C1=CC=C(C=C1)COC[C@H](C(=O)O)N	AAGTATA
62	C1=CC(=CC=C1CC(C(=O)O)N)C#N	ATGCCTG
63	O=C(O)[C@@H](N)C/C=C/C1=CC=CC=C1	TTCCGCG
64	O=C(O)[C@@H](N)CC1=CC=C(C2=CC=CC=C2)C=C1	GCCAATC
65	O=C(O)C(N)CC1=CC=C(OCO2)C2=C1	GTAAGAA
66	O=C(O)[C@@H](N)C1CC2=C(C=CC=C2)C1	CAGGCGA
67	C1=CC=C(C(=C1)CC(C(=O)O)N)O	TGTCGAG
68	O=C(O)[C@H](CC1=CC=CS1)N	CGTATAT
69	C[C@@H](CC(=O)O)N	TGCAAGC
70	CC(C)C[C@@H](CC(=O)O)N	TAAGGCT
71	CC(C)[C@H](CC(=O)O)N	GACTCCT
72	C1=CC=C(C=C1)C[C@@H](CC(=O)O)N	TGTCGCT
73	C(CN)C(=O)O	CTTCCGA
74	C1=CC(=CC(=C1)C(F)(F)F)[C@H](CC(=O)O)N	TTCGCCT
75	C1=CC(=CC(=C1)C(F)(F)F)[C@@H](CC(=O)O)N	CTAGCAT
76	C1=CC(=CC=C1C[C@@H](CC(=O)O)N)O	AGAGGAT
77	CC1=CC=C(C=C1)C[C@H](CC(=O)O)N	AAGCCAG
78	N[C@H](CC1=CC=CC=C1Cl)CC(O)=O	AATGGAG
79	N[C@H](CCC(N)=O)CC(O)=O	TCCAGTT
80	C1=CC(=CC=C1CC(=O)O)CN	CTGTGAG

81	C1=CC(=CC(=C1)CN)CC(=O)O	GTCTTGC
82	C1=CC=C(C(=C1)CC(=O)O)CN	ATTAGTA
83	O=C([C@H]1CC[C@@H](N)CC1)O	CGTAGCT
84	O=C([C@H]1C[C@@H](N)CCC1)O	AGTTCTG
85	O=C([C@H]1[C@H](N)CCCC1)O	CTATGAA
86	O=C([C@H]1[C@@H](N)CCCC1)O	ATCCACG
87	C(COCCN)C(=O)O	TCACGGT
88	C(CC(=O)O)CN	CACCGAC
89	O=C(O)CC(C)(C)CN	AACTGGA
90	C1=CC(=CC=C1C(=O)O)OCCN	TCAAGAT
91	O=C(O)C1=CC=CC(CN)=C1	TGTGTAT
92	C1=CC=C2C(=C1)C(C3=CC=CC=C32)COC(=O)NCC4=CC=C(C=C4)C(=O)O	ACAATCG
93	O=C([C@H]1[C@@H](CN)OCC1)O	AAGCAAT
94	C1=CC(=CC=C1C(=O)O)N	GTAGCAG
95	O=C(O)C(N)C1(C2)CC2(C(F)(F)F)C1	TGGCCTC
96	C1=CC=C(C=C1)CC(C(=O)NCC(=O)O)N	GATAACT
97	O=C(O)CNC([C@@H](N)CC1=CSC=N1)=O	CGGTCAT
98	C(CC(=O)N)[C@@H](C(=O)NCC(=O)O)N	CATAAGT
99	C[C@@H](C(O)=O)NC([C@@H](CC(C)C)N)=O	ACGCGAT
100	C[C@@H](C(O)=O)NC([C@H](N)CCCC)=O	CATAGGA
101	C[C@@H](C(O)=O)NC([C@@H](N)CC1=CSC=N1)=O	TAATGGT
102	CC(C)C[C@@H](C(=O)O)NC(=O)[C@H](C(C)C)N	AGCTCGC
103	CC(C)C[C@@H](C(O)=O)NC([C@H](CCCCNC(C)=O)N)=O	CCTTGAC
104	CC(C)C[C@@H](C(=O)O)NC(=O)[C@H](CCC(=O)N)N	AATCGTA
105	C[C@H]([C@@H](C(=O)N[C@@H](C(C)C)C(=O)O)N)O	CACCTCT
106	C[C@@H](C(=O)N[C@@H](C(C)C)C(=O)O)N	CATTGAG
107	CC(C)[C@@H](C(O)=O)NC([C@H](N)CC#N)=O	TTGGTAA
108	CC(C)[C@@H](C(O)=O)NC([C@@H](N)CC(OC)=O)=O	TCAGCAG
109	O=C(O)CNC([C@@H](N)CC(OC)=O)=O	TAGGTGA
110	CC(C)C[C@@H](C(=O)N[C@@H](CC1=CC=CC=C1)C(=O)O)N	AATACTT
111	OC1=CC=C(C=C1)C[C@@H](C(O)=O)NC([C@@H](N)CCCNC(N)=O)=O	GATGTCG
112	OC1=CC=C(C=C1)C[C@@H](C(O)=O)NC([C@H](N)CC2=CC=C(Cl)C=C2)=O	ATCTTAC
113	C1=CC=C2C(=C1)C(=CN2)C[C@@H](C(=O)O)NC(=O)[C@@H](CCC(=O)N)N	CCTACCG
114	C1=CC=C2C(=C1)C(=CN2)C[C@@H](C(=O)O)NC(=O)[C@H](CO)N	TTAATTA
115	O=C(O)[C@H](CC1=CNC2=C1C=CC=C2)NC([C@@H](N)CC3=CC=C(Cl)C=C3)=O	GAGCGGT
116	CC[C@H](C)[C@@H](C(=O)O)NC(=O)[C@H](CC(=O)N)N	CTCATGA
117	CC[C@H](C)[C@@H](C(=O)O)NC(=O)[C@H]([C@@H](C)O)N	AGAGCCG
118	CC[C@H](C)[C@@H](C(O)=O)NC([C@@H](C)N)=O	GCTACTT
119	C[C@H]([C@@H](C(=O)N[C@@H](CCC(=O)N)C(=O)O)N)O	GAACATA
120	O=C(N)CC[C@@H](C(O)=O)NC([C@H](N)CC#N)=O	TGCCTAG
121	O=C(N)CC[C@@H](C(O)=O)NC([C@@H](N)CC1CCCCC1)=O	ACTAGAA
122	CN(CC(=O)O)C(=O)CN	ACAAGGC
123	O=C(O)CN(C([C@@H](N)CCCC)=O)C	TTATCCA
124	O=C([C@@H]1N(C([C@H](CCCCNC(C)=O)N)=O)CC(F)(F)C1)O	TGGCCAT
125	OC1=CC=C(C=C1)C[C@@H](C(O)=O)NC([C@@H](N)CCCC)=O	CAACAAT
126	O=C(O)[C@@H](NC(CN)=O)CC(OC(C)(C)C)=O	GCCAGTA
127	C[C@H](C(O)=O)NC([C@@H](CO)N)=O	TTCCGAC
128	C[C@H](C(O)=O)NC([C@@H](N)CC1=CC=CN=C1)=O	TGACCGA
129	C[C@H](C(=O)N[C@H](C)C(=O)O)N	GTCTGAG
130	C[C@H]([C@@H](C(=O)O)NC(=O)[C@H](C)N)O	GTCGTCG
131	O=C(O)CC(N)C1=CC=C(C(C)C)C=C1	GAACAGC
132	CC(C)C[C@H](C(O)=O)NC([C@@H](CC1=CC=C(O)C=C1)N)=O	GAAGAGT
133	CC(C)C[C@H](C(=O)O)NC(=O)CN	AACGCGT
134	CC(C)C[C@H](C(O)=O)NC([C@@H](CC1=CC=CC=C1)N)=O	AATCTAG
135	CC(C)C[C@@H](C(=O)N[C@H](C(C)C)C(=O)O)N	TCGACTC
136	CC(C)[C@H](C(O)=O)NC([C@H](N)CC1=CC=CN=C1)=O	ACGAAGC
137	O=C(O)[C@@H](CC1=CC=CC=C1)NC([C@H](N)CCCNC(N)=O)=O	ATCGTGC
138	C1=CC=C(C=C1)C[C@@H](C(=O)O)NC(=O)[C@H](CC2=CC=C(C=C2)O)N	CGCTCTG
139	OC1=CC=C(C=C1)C[C@H](C(O)=O)NC([C@H](C)N)=O	GCATCTC
140	OC1=CC=C(C=C1)C[C@H](C(O)=O)NC([C@@H]([C@@H](O)C)N)=O	ATGGTCG
141	O=C(O)[C@@H](CC1=CNC2=C1C=CC=C2)NC([C@H](C(C)C)N)=O	CAGGAGC
142	C(CC(=O)N)[C@@H](C(=O)O)NC(=O)[C@H](CC(=O)N)N	GAGGCTA
143	O=C(O)[C@H]1N(C([C@@H](CO)N)=O)CCC1	TGTCCAA
144	C1C[C@@H](N(C1)C(=O)CN)C(=O)O	TCTGTTC
145	[2H]N[C@H](C)C(=O)N1CCC[C@H]1C(=O)O	ATCGCAT
146	O=C([C@H]1N(C([C@H](CC2=CC=CC=C2)N)=O)C(C)(C)OC1)O	AGCAAGT
147	O=C([C@H]1N(C([C@H](CC(C)C)N)=O)C(C)(C)OC1)O	CGCGCAT

148	O=C([C@@H]1N(C([C@@H](C(C)C)N)=O)C(C)(C)O[C@H]1C)O	TCGGAGT
149	C1C[C@H](N(C1)C(=O)CN)C(=O)O	TCTCTCA
150	O=C([C@H]1N(C([C@@H](C)N)=O)C(C)(C)OC1)O	TATTCCA
151	N[C@@H](CCCCNC(CCC1=CC=C(C2=CC=CC=C2C(F)(F)F)O1)=O)C(O)=O	TGGTCCT
152	*NULL*	TATATCT

4.6. Step 2 Headpiece 3' (HP3) Sublibrary

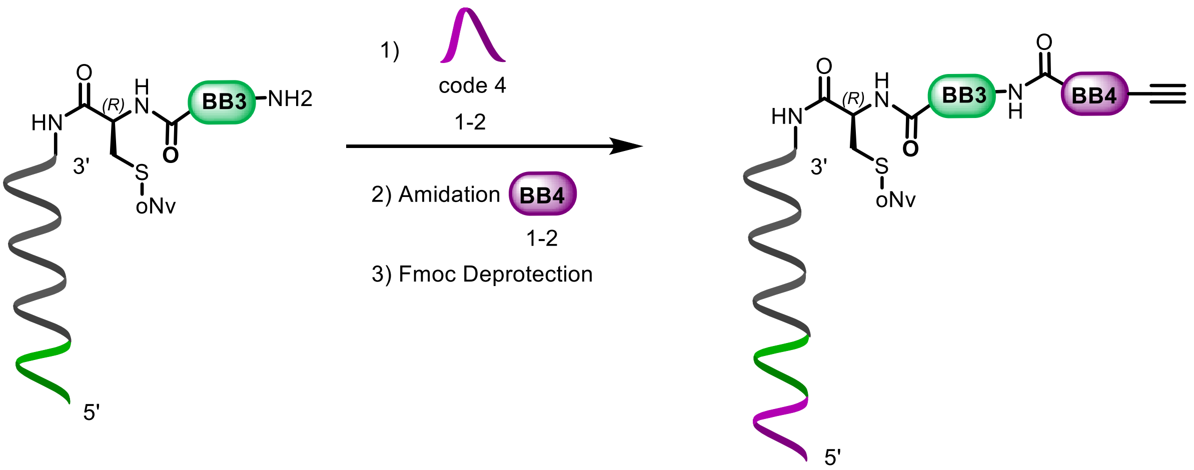

500 nmol was taken from HP3 step 1 purified pool, and divided in two. Code 4 was ligated to the pool in 5 50 nmol batches for each code performed in 2 mL Eppendorf tubes according to general procedure. After ligation completion, the conjugates were precipitated, redissolved and reacted with respective BB4 building blocks using the DMT-MM general protocol. After the reaction. the step 2 conjugates were then pooled together and treated with piperidine (conditions identical to Fmoc deprotection), in order to remove any DMT-MM capping that could have occurred during amidation. The piperidine was quenched with sodium acetate and the pool precipitated with ethanol. The HP3 step 2 pool was finally purified using 60 °C HPLC to remove excess codes/adaptor, precipitated and analyzed. A final yield of 284 nmol was obtained after purification.

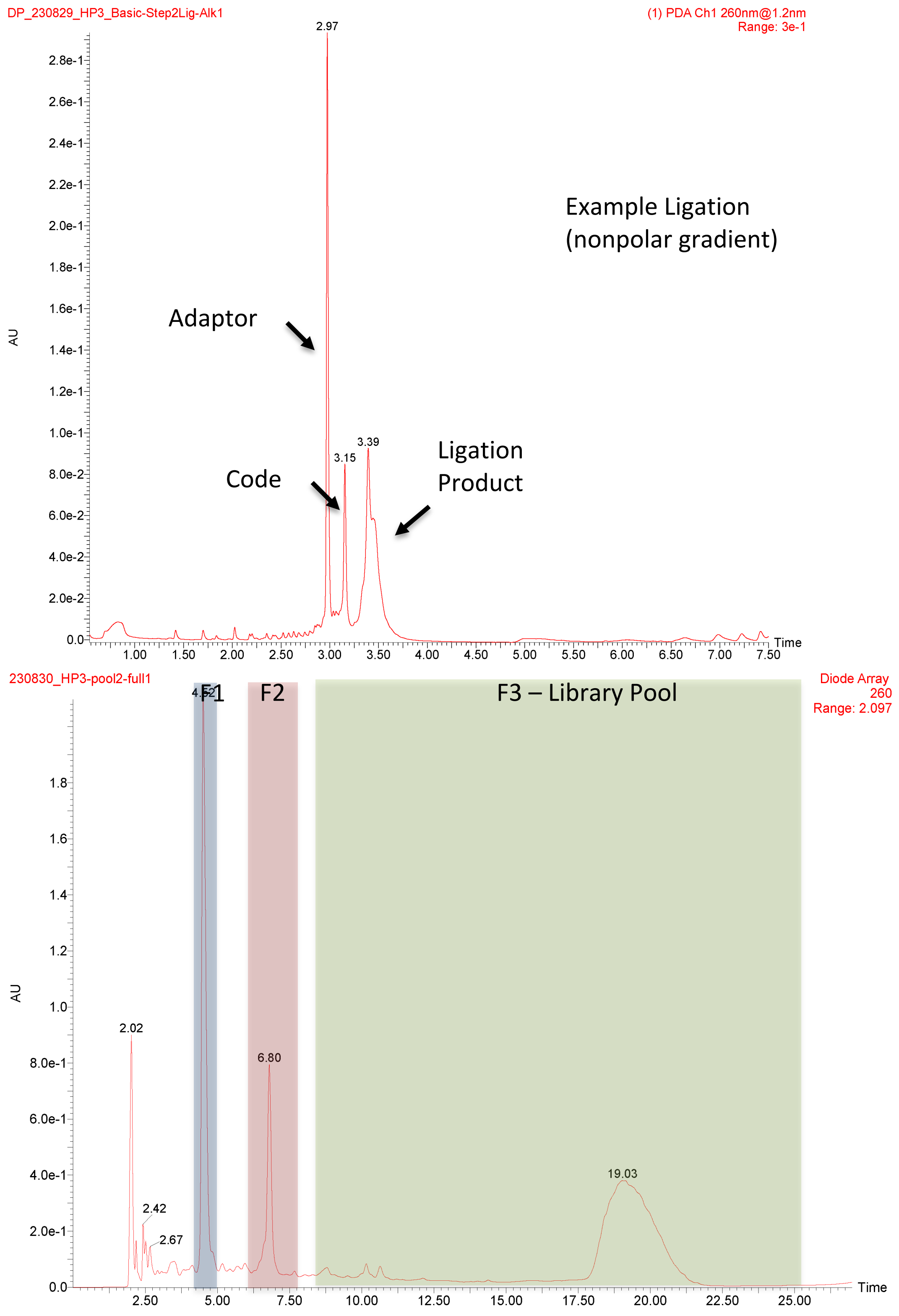

DP_230829_HP3_Basic-Step2Lig-Alk1
(1) PDA Ch1 260nm@1.2nm
Range: 3e-1
Example Ligation
(nonpolar gradient)
Adaptor
Code
Ligation
Product
2.97
3.15
3.39
AU
Time
230830_HP3-pool2-full1
Diode Array
260
Range: 2.097
F1
F2
F3 – Library Pool
2.02
2.42
2.67
6.80
19.03
AU
Time

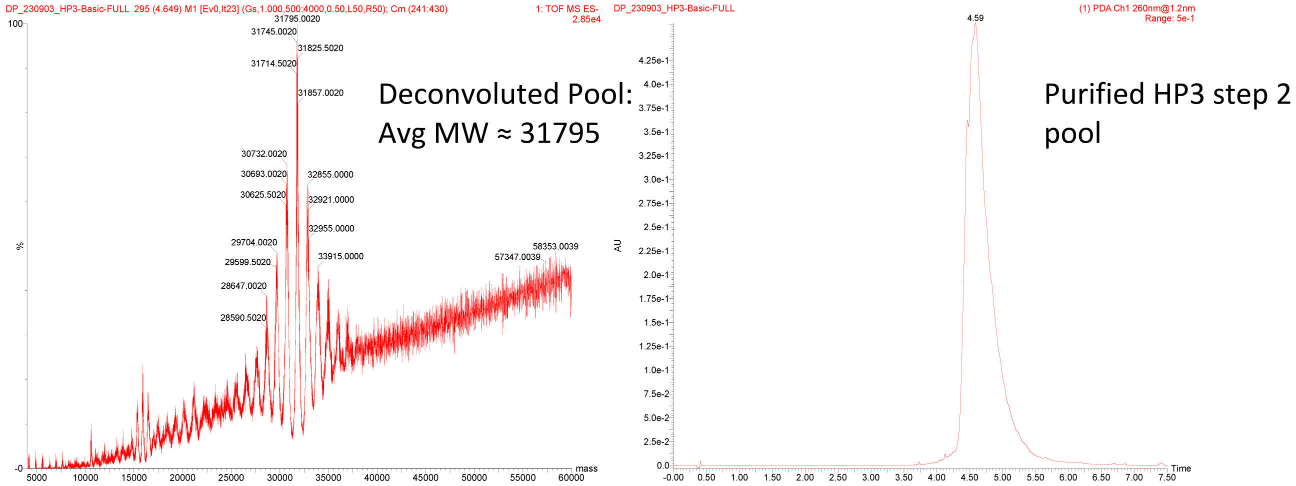

Figure S24: Characterization of HP3 step 2 library synthesis. Top: Example ligation of code 4 onto building block 2 HP3 pool 1 conjugates. Middle: HPLC purification of library pool after finished ligation. Bottom: UV chromatogram of purified step 2 HP3 pool and MS deconvolution giving an average mass

Table S9: List building blocks 4 and oligonucleotide codons encoding them in the code 4 position

BB #	SMILES	Code (5'-3')
1	C#CCCC(O)=O	CATTGCG
2	O=C(O)[C@H]1N(CC#C)CCC1	AGTAGTC

4.7. ESAC Library Creation & Derivatization

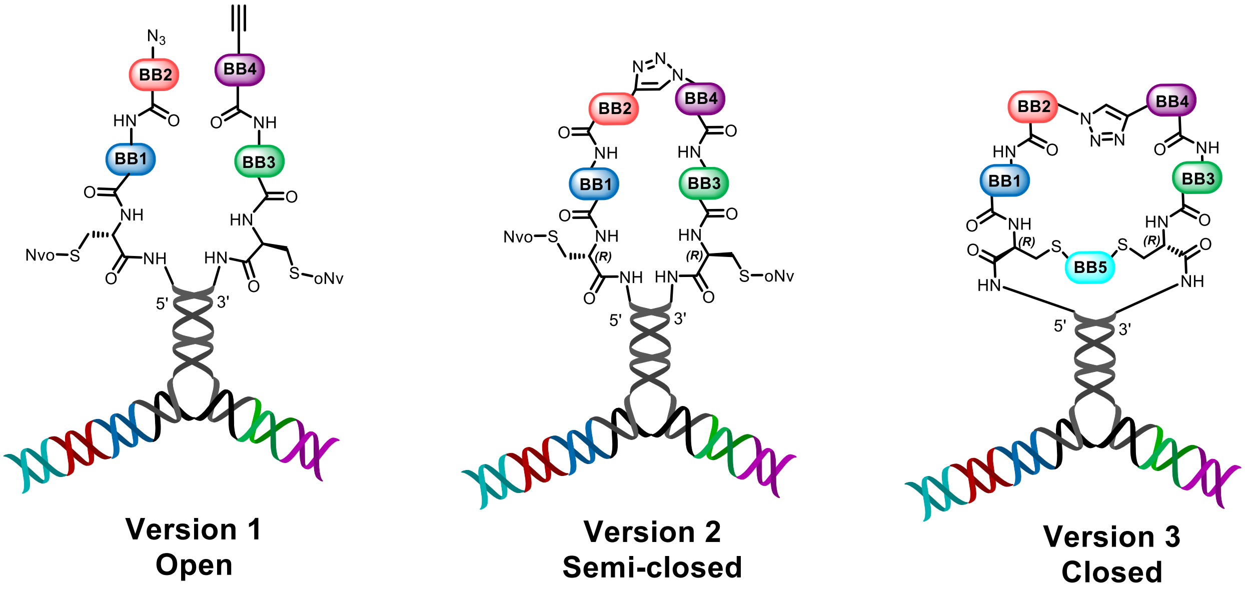

Figure S25: Summary of different ways through which the 2 finished sub libraries were combined expressed in 3 different library versions of DP-DEL.

A total of 3 versions of DP-DEL were generated via different combinations of the HP5 and HP3 step 2 pool sublibraries through mixing and interstrand reactions. Library versions were encoded through 6 different library terminal primers, sometimes also referred to as code 5.

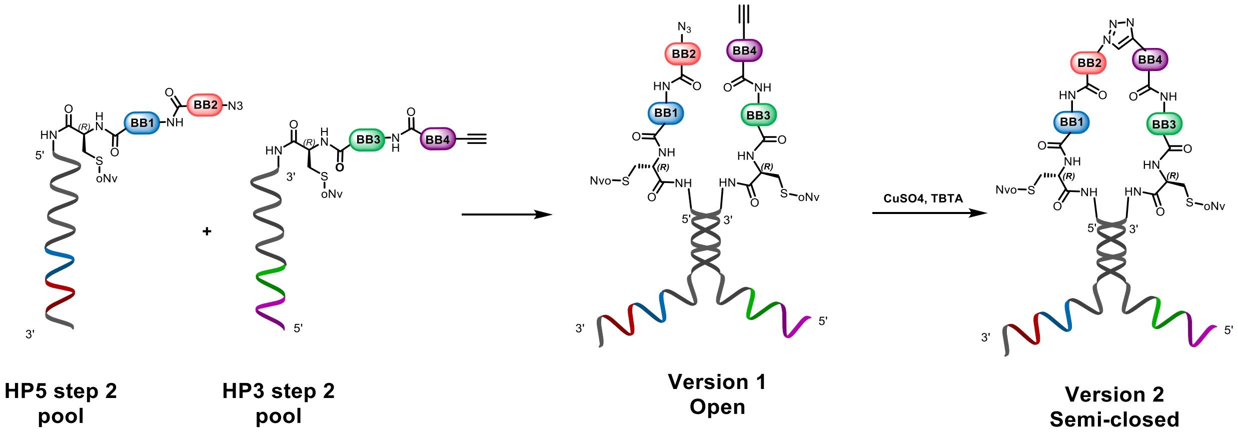

Figure S26: Scheme for the generation of versions 1 and 2 of DP-DEL

Generation of Library Versions 1 and 2

Version 1 of library was created by mixing Step 2 HP5 & HP3 sublibrary pools in equimolar amounts, and then directly proceeding to the terminal primer encoding step.

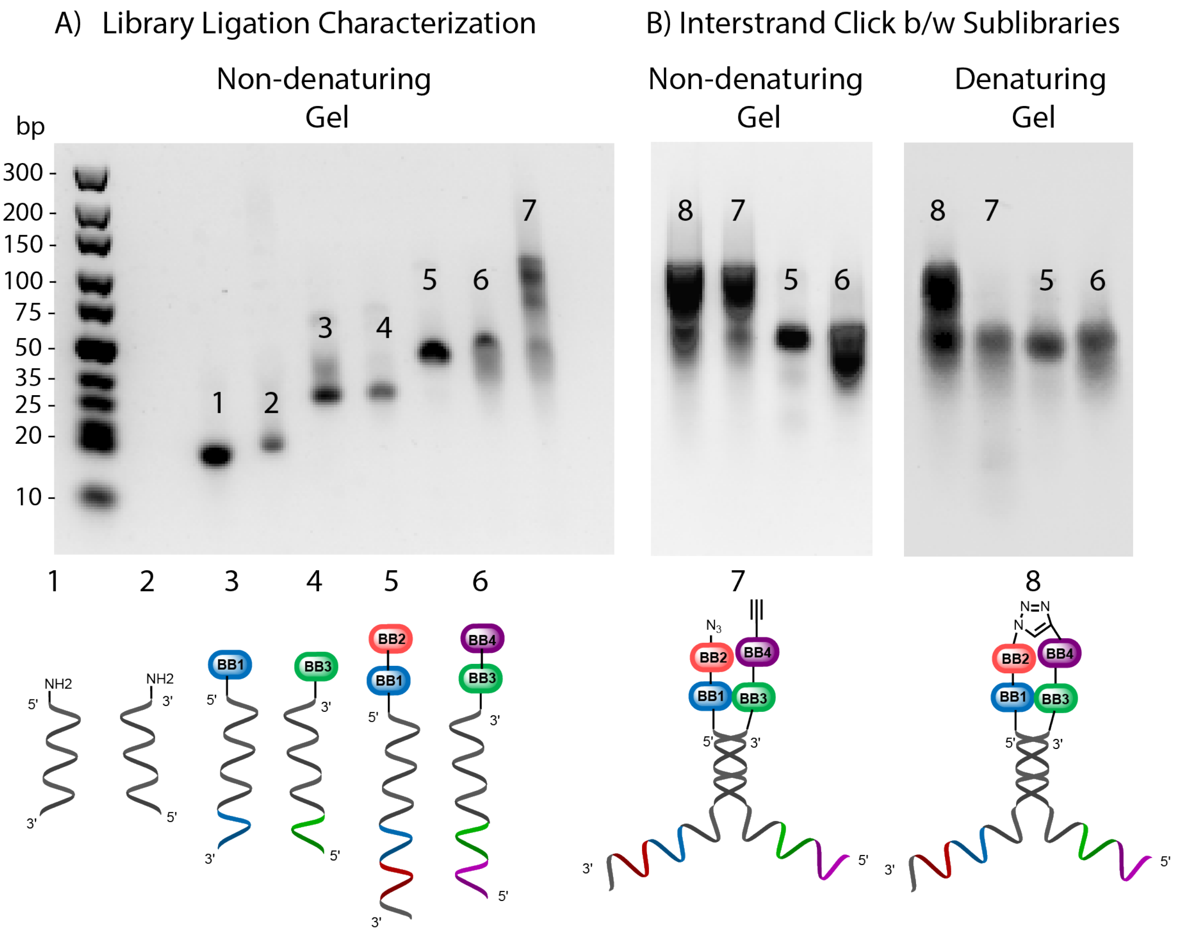

Figure S27: Agarose gel electrophoresis-based analysis of DP-DEL synthesis. A) Gel analysis of HP5 & HP3 headpieces (1 & 2), HP5 & HP3 pool step 1 (3 & 4), HP5 & HP3 pool step 2 (5 & 6) and hybridization of finished step 2 pools (7). B) Gel electrophoresis-based proof for the occurrence of an interstrand click reaction to form version 2 of DP-DEL. Denaturing gel separates complementary DNA strands, thus complementary sublibaries occur as one heavier band in non-denaturing gels (7). Covalently clicked sub-libraries should be inseparable, thus not showing a clear difference in denaturing & non-denaturing conditions (8).

Version 2 of the library was created when 7 10 nmol batches of Step 2 HP5 & HP3 sublibrary pools respectively were mixed in 22 µL mQ and hybridized by raising temperature to 80 °C and cooling to room temperature. Then the interstrand click reaction was carried out as described in section 2.2. The clicked pools were then HPLC purified at 60 °C to remove any contaminants from the click reaction and precipitated. A final yield of 41 nmol version 2 clicked library was obtained from the 70 nmol starting material. Part of the clicked library product was then encoded via terminal primer while the rest was carried over for generation of DP-DEL version 3.

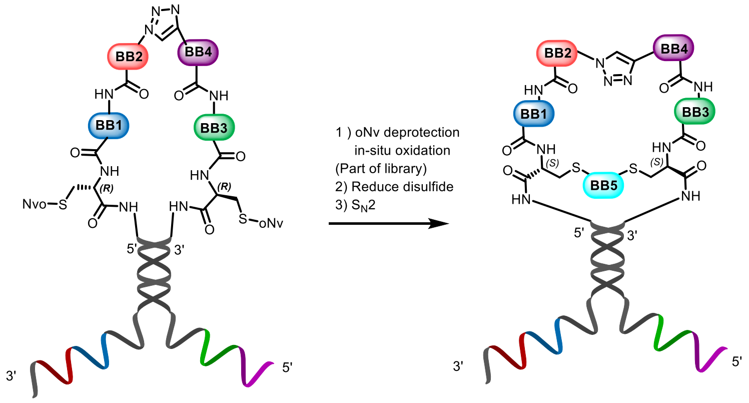

Figure S28: Scheme for the generation of version 3 of DP-DEL

Generation of Library Versions 3

Library scaffold deprotection performed on 1 nmol scale in 1.5 mL Eppendorf tubes using unencoded version 2 (clicked) of DP-DEL. Scaffold deprotection was performed based on general protocol. Part of the library (version 3 - disulfide) was then precipitated and encoded, while the rest of the product was carried over and directly treated under thioether formation conditions as described in section 2.2 and precipitated to form electrophile derivatized versions 3 of DP-DEL.

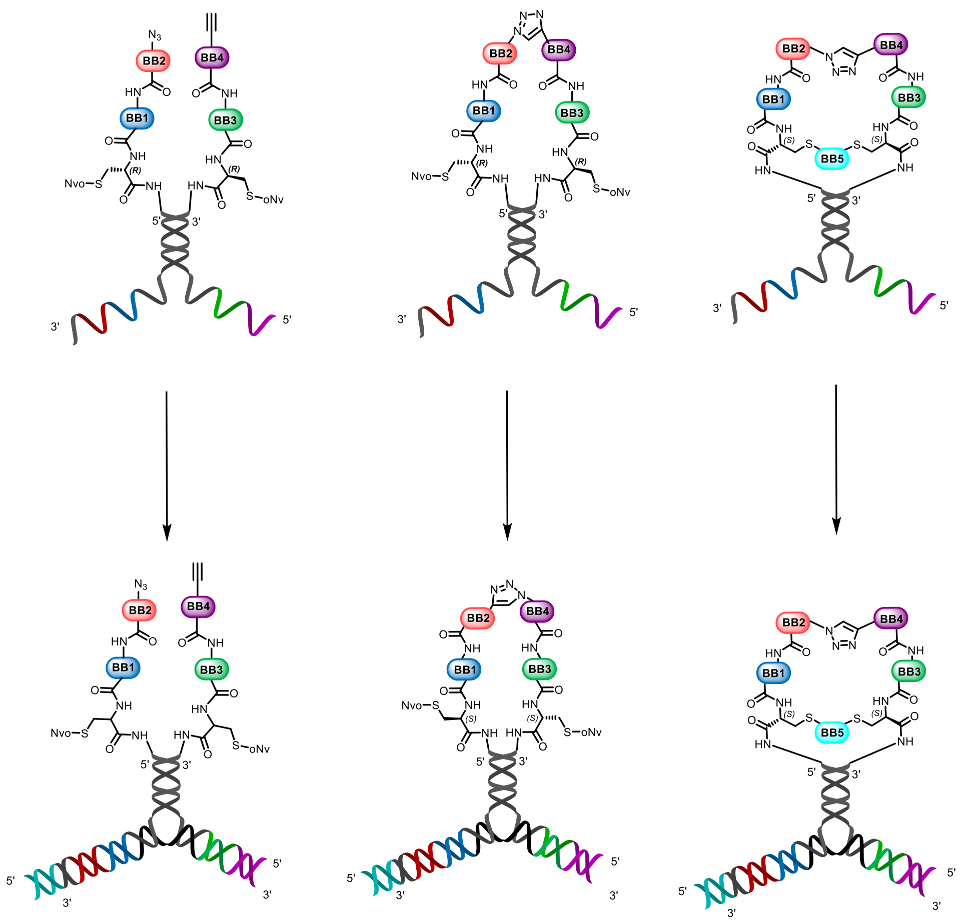

Figure S29: Scheme for encoding of library version through the introduction of 6 different terminal primers followed by polymerization of Y-shaped encoding

Terminal Primer Encoding

1 nmol batches of Versions 1-3 of DP-DEL were respectively taken and encoded according to terminal Primer Installation/ Polymerization of Large encoding design protocol in section 2.2. Version 3 of the library utilized 4 different primers. After encoding, all versions of DP-DEL were pooled to generate the master library.

Table S 10: List of library versions and oligonucleotide codons encoding them in the terminal primer position

BB # 5	Library Version	Terminal Primer Codon (5' – 3') Selection 1	Terminal Primer Codon (5' – 3') Selection 2
1	Version 1 open Library	GGTCG	-
2	Version 2 clicked Library	ATGCT	GGTCG
3	Version 3 "Disulfide" Library	ACCTA	ATGCT
4	Version 3 "Thioether 1" BrCC1=CC=CC(CBr)=C1	CTCAG	ACCTA
5	Version 3 "Thioether 2" ClC/C=C\CCl	TCCAG	CTCAG
6	Version 3 "Thioether 3" BrC/C=C/CBr	CTCAC	TCCAG

5. Affinity Selections

5.1. Selection Procedure

Affinity selections were performed according to established protocols by Decurtins et al. [9] using an automated KingFisher Flex magnetic particle processor. Selections were performed on two separate rounds. The first selection was performed using all 3 versions of the library with 10^7 copies for each compound. The second selection was performed using library versions 2-3 with $3*10^7$ copies for each compound. All selections were performed in triplicate.

5.2. PCR Amplification of Library

The following PCR protocol was utilized. All buffers and reagents were obtained from New England Biolabs. The number of amplification cycles were varied according to recommendations by Decurtins et al.

	50µL - ref	Master mix (x17)
DNA solution	5	x
HF buffer	10	170.0
dNTPs	1	17.0
Primer a	2.08	35.4
Primer b	2.23	37.9
Phusion HSF	0.25	4.25
mQ H_2O	29.44	500.5

1- 98°C-30s
2- 98°C-10s |
3- 61°C-30s |
4- 72°C-15s |(**34**x from step 2)
5- 72°C-5min
6- 4°C – pause

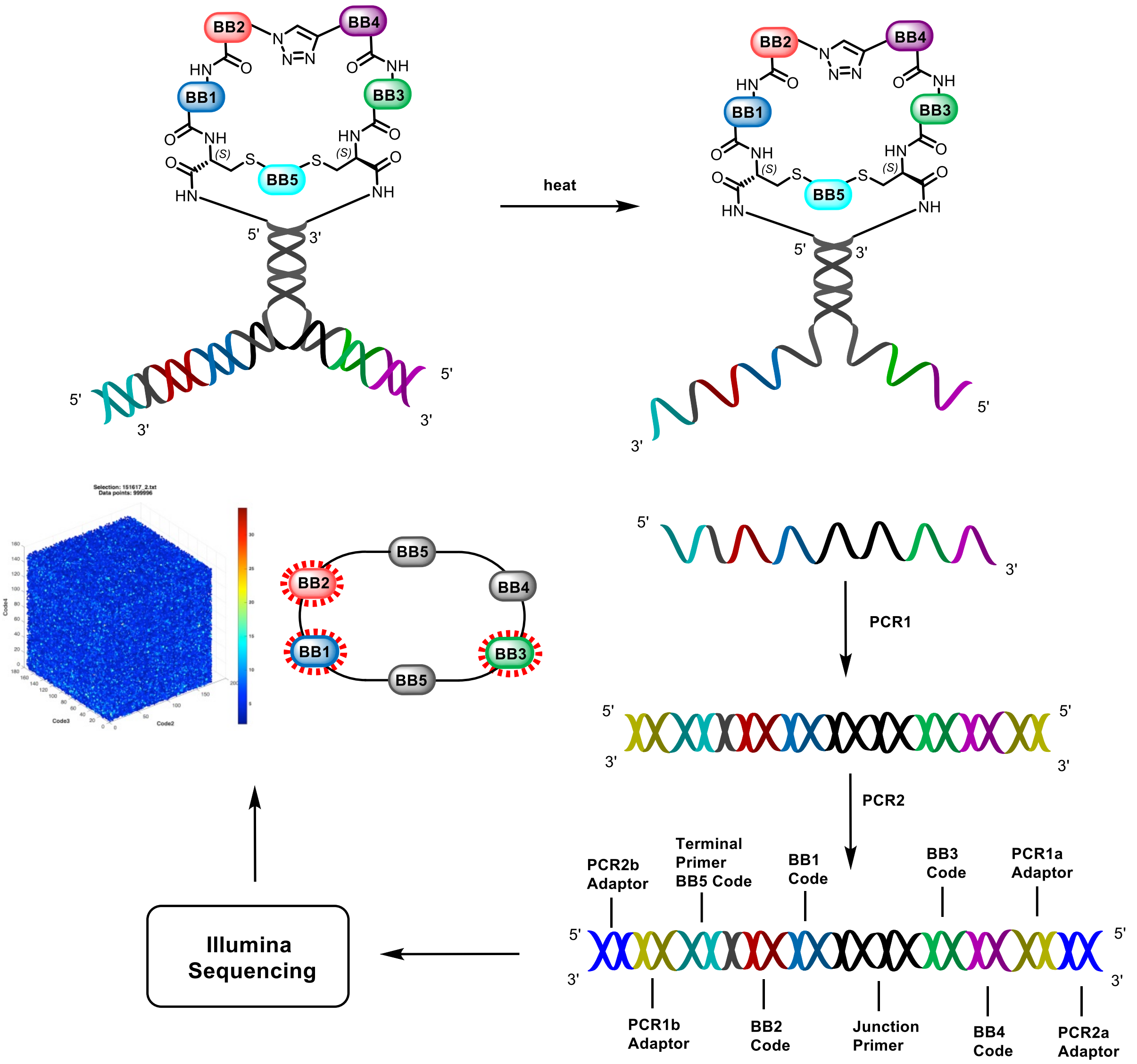

Figure S30: Post-selection work through for library processing prior to sequencing. After selecting the library, the Y shaped double stranded structure is disrupted by heating the library, thus releasing the complete encoding strand in a single DNA strand. This strand then multiplied and PCR1 & 2 adaptors are installed as detailed in Decurtins et al. and table S11.

PCR2 product

5’ CAAGCAGAAGACGGCATACGAGAT XXXXXXXX GTGACTGGAGTT CAG ACG TGT GCT CTT CCG XXXXX ATC TGC ATC AGT TCA TGG GTA X XXX XCA GGA CCG AGT TGA C X XXX XXX GTGTGAGCAA CGTACGACAG XXX XXX G AGA GAC TAC G AGT GAC CAG TT TG AGG TGA CT AGCGAGCGAA XXX XXX GTAGCCCTTC CACTCACTCA X XXX XXX CCATCGATCGTCAAGGATT CT XXXXX TC AGA TCG GAA GAG CGT CGT GT GGGAAAGAGTGT XXXXXXXX GTGTAGATCTCGGTGGTCGCCGTATCATT 3’

5’ AAT GAT ACG GCG ACC ACC GAG ATC TAC AC XXXXXXXX ACA CTC TTT CCC TAC ACG ACG CTC TTC CGA TCT GA XXXXX AGA ATC CTT GAC GAT CGA TGG X XXX XXX TGA GTG AGT GGA AGG GCT AC XXX XXX TTC GCT CGC TAG TCA CCT CAA ACT GGT CAC TCG TAG TCT CTC XXX XXX CTG TCG TAC GTT GCT CAC AC X XXX XXX GTC AAC TCG GTC CTG XXX XXT ACC CAT GAA CTG ATG CAG AT XXXXX CGG AAG AGC ACA CGT CTG AAC TCC AGT CAC XXXXXXXX A TCT CGT ATG CCG TCT TCT GCT TG 3’

XXXXXXXX – **PCR2a**
XXXXX – **PCR1a**
X XXX XXX **Code 4 (HP3) BB4**
XXX XXX **Code 3 (HP3) BB3**
XXX XXX **Code 1 (HP5) BB1**
X XXX XXX – **Code 2 (HP5) BB2**
XXX XX – **Terminal Primer (Sublibrary interlinker)**

XXXXX – PCR1b
XXXXXXXX – PCR2b

Table S11: PCR primers used for post-selection library amplification

PCR1a	5' TACACGACGCTCTTCCGATCTGA XXXXX AGAATCCTTGACGATCGATGG 3'
PCR1b	5’ CAGACGTGTGCTCTTCCG XXXXX ATCTGCATCAGTTCATGGGTA 3'
PCR2a	5’ AATGATACGGCGACCACCGAGATCTACAC XXXXXXXX ACACTCTTTCCCTACACGACGCTCTTCCGATCT 3'
PCR2b	5’ CAAGCAGAAGACGGCATACGAGAT XXXXXXXX GTGACTGGAGTTCAGACGTGTGCTCTTCCGATC 3'

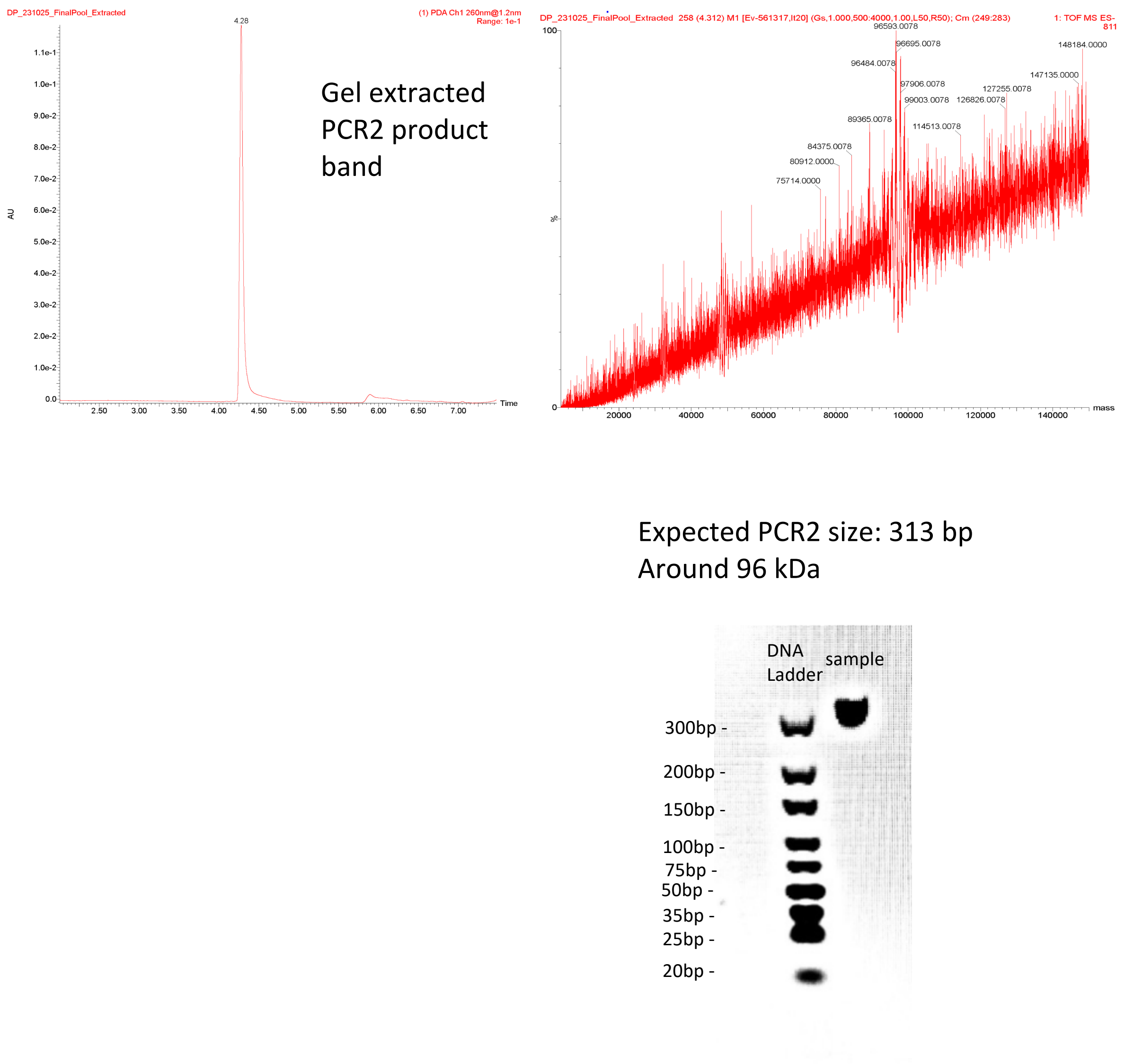

Figure S31: LC-MS and gel-based characterization of sequencing-ready selected DP-DEL

5.3. Proteins for Selections

Table S12: List of proteins used for selections

Protein	buffer	MW (Da)	tag	beads	Concentration
CAIX	PBS pH 7.4	62355 (dimer)	His-tag	HisPur Ni-NTA Magnetic Beads (ThermoFisher Scientific)	2 µM
NSP14	50mM Tris-HCl, 200mM NaCl, 5% (w/v) glycerol, 0.5 mM TCEP, pH8	61857	biotin	Dynabeads™ MyOne™ Streptavidin C1 (Invitrogen)	
Thrombin	aqueous buffer of pH 6.0	36700	biotin		
PLAP	PBS pH 7.4	52745	biotin		
Streptavidin	PBS pH 7.4	-	-		-

5.3.1. CAIX

The extracellular domain of human carbonic anhydrase IX (120-397) with a C-terminal hexahistidine-tag was produced according to procedure described in the Ahlskog et al. 2009 [10]

5.3.2. NSP14

The bacterial expression plasmid was kindly provided by the group of Prof. Nenad Ban at ETH Zürich. The protein was expressed for 20h at 16°C in a commercially available *E. Coli BL21(DE3)* strain carrying a second plasmid for in-vivo site-specific biotinylation (CVB-T7 POL, Avidity L.L.C.). Cleared cell-lysate was obtained by snap-freezing, sonication and ultracentrifugation. The protein was purified subsequently by Ni-NTA affinity chromatography and a Heparin column. Eluates were pooled and incubated for 1h at 30°C with 50 ug of SUMO protease ulp1 per mg of target protein to cleave His14-SUMO-nsp14 fusion protein. Final nsp14 was purified by reverse Ni-NTA affinity chromatography and desalted and concentrated by centrifugal filter. The functionality of the protein was assessed by SDS-PAGE and by an exonuclease activity assay

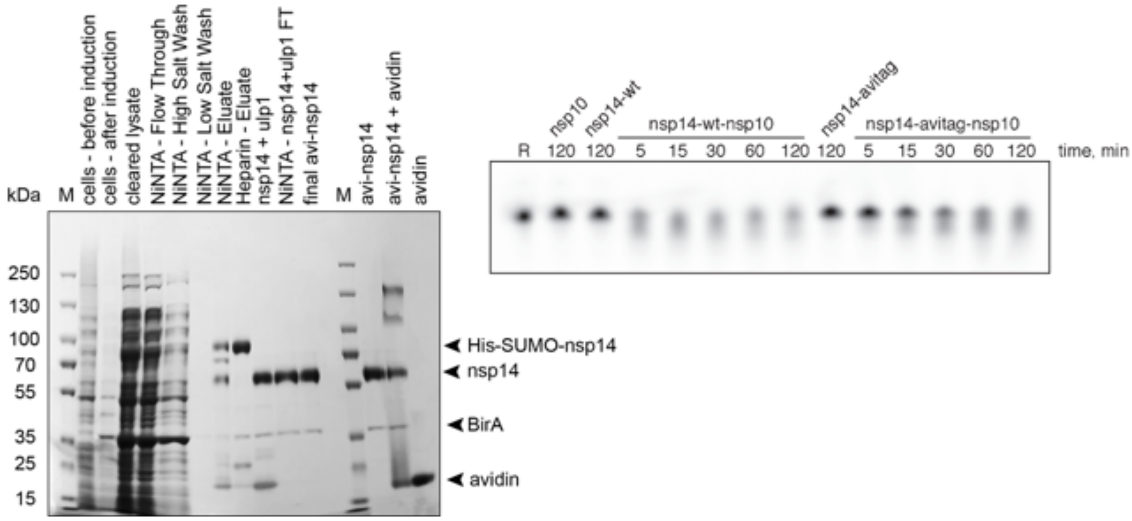

5.3.3. Thrombin

Human alpha-thrombin was obtained from Prolytix (Haematologic Technologies) - BioConnect Website and used in FP and inhibition assays. Biotinylated thrombin used for selections was obtained from Sigma Aldrich.

5.3.4. PLAP

The recombinant human placental alkaline phosphatase (hPLAP) labelled with AviTag™ and 6xHis-tag was produced in Chinese hamster ovary (CHO) cells and was purified by Ni-NTA affinity chromatography and size-exclusion chromatography. The *in-vitro* biotinylation of the protein is mediated by BirA protein system. “Early-fraction” is the final material used for fluorescence polarization and inhibition experiments.

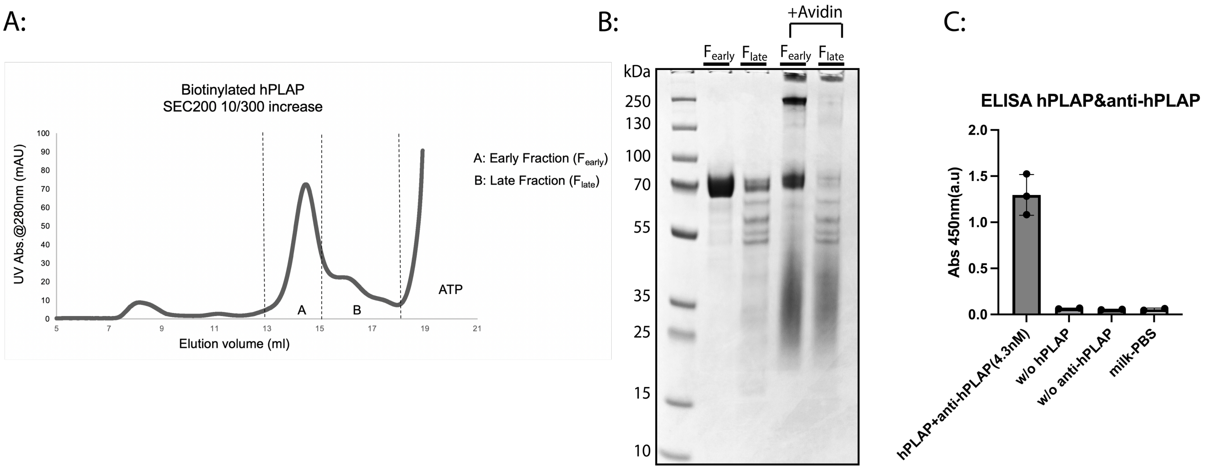

Figure S32: Quality characterization of recombinant hPLAP. A. The size exclusion chromatogram of purifying recombinant hPLAP expressed in CHO cells after Ni-NTA affinity chromatography purification and biotinylation. B. SDS-Page gel electrophoresis analysis of early fractions (F_{ealy}) and late fractions (F_{late}) from size exclusion chromatography with or without adding avidin. V. Qualitative ELISA to verify the recombinant hPLAP is able to bind with human PLAP antibody. The absorbances at 450nm were measured with/without the presence of hPLAP and/or human PLAP antibody. Figure adapted from Cai, J. (2023). ***A novel dual-display DNA-encoded library for ligand discovery of tumor-associated antigens*** **[Unpublished master's thesis]. ETH Zurich.**

5.4. Sequencing Fingerprints Analysis

5.4.1. Notes of Fingerprint Analysis

For easier visual interpretation, the counts of each replicate experiment were normalized and concatenated using a python script shown below. The concatenated results were used to generate the visual graphs shown in all main figures and fingerprints shown below. Enrichment factors were still calculated based per each individual experiment.
Code combinations are listed in the order they are read during sequencing, starting from PCR2a side. This means the 5 code combinations are listed as: (code4, code3, code1, code2, code5) in that order. When a code is entered as 0, this means a code is disregarded. Ex. 0;176;175;0;0 means the counts of all members are shown that possess code3-176 and code1-175 regardless of what other codes these members possess.

Results from first selection round are shown for Naive library, CAIX, NSP14 and version 1 of Thrombin, Streptavidin and PLAP. Results from second selection round are shown for Thrombin, Streptavidin, PLAP and naïve library.

Python script:

```
import pandas as pd

# Load data from the three text files into DataFrames
data1 =
pd.read_csv('/Users/dimitar.petrov/PycharmProjects/pythonProject1/selection
_12_2_.txt', delimiter='\t')  # Adjust the delimiter as needed
data2 =
pd.read_csv('/Users/dimitar.petrov/PycharmProjects/pythonProject1/selection
_13_2_.txt', delimiter='\t')
data3 =
pd.read_csv('/Users/dimitar.petrov/PycharmProjects/pythonProject1/selection
_14_2_.txt', delimiter='\t')

# Function to normalize counts in each document to a certain value
def normalize_counts(data):
    total_count = data['Count'].sum()
    scaling_factor = 3000000 / total_count  # Calculate the scaling factor
    data['Count'] = data['Count'] * scaling_factor  # Scale the counts
    return data

# Normalize counts in each dataset
data1 = normalize_counts(data1)
data2 = normalize_counts(data2)
data3 = normalize_counts(data3)

# Concatenate the three DataFrames
combined_data = pd.concat([data1, data2, data3], ignore_index=True)

# Reorder the columns so that 'Count' is the rightmost column
combined_data = combined_data[['Count', 'Code1', 'Code2', 'Code3', 'Code4',
'Code5']]

# Group by 'Code1', 'Code2', and 'Code3' and sum the 'Count' column
result = combined_data.groupby(['Code1', 'Code2', 'Code3', 'Code4',
'Code5'])['Count'].sum().reset_index()
```

```
# Reorder the columns so that 'Count' is the leftmost column
result = result[['Count', 'Code1', 'Code2', 'Code3', 'Code4', 'Code5']]

# Write the result to a new text file
result.to_csv('selection_121314_2.txt', sep='\t', index=False)  # Adjust
the delimiter as needed
```

Script was generated with help from ChatGPT (OpenAI)

Equations for Enrichment Calculation

$$TC_s = \sum_{i=1}^{175}\sum_{j=1}^{152}\sum_{k=1}^{176}\sum_{l=1}^{2}\sum_{m=1}^{6} SC_s\left(code1_i, code2_j, code3_k, code4_l, code5_m\right)$$

$$AC_s = \frac{TC_s}{i * j * k * l * m}$$

$$EF = \frac{SC_s\left(code1_i, code2_j, code3_k, code4_l, code5_m\right)}{AC_s}$$

Equation 1: Total counts (TC) are given for a specific selection s, where the letters i-m define the number of codes 1-5. Sequence counts (SC) are generated for each code combination based on illumina sequencing output. Average counts (AC) define the average number of counts each code combination should have assuming equal distribution of total counts. Enrichment factor (EF) is calculated for a specific code combination using its sequence and average counts.

5.4.2. Statistical Analysis

Table S13: Statistical evaluation of selection hit outcomes. Calculations are done based on given formulas in Equation 1. When certain codes are disregarded for certain combinations such as CAIX and NSP14, EF are calculated based on the correspondingly reduced number of members. The same is correspondingly done for hits enriched from selection 2 of the library (thrombin & streptavidin) where the open library (0;0;0;0;1) was not tested in selection

Replicate	Target	Combination	Average Counts (All codes)	Total Counts	Enrichment factor
1	Naive/None	-	0.0353104	1983710	-
2	Naive/None	-	0.0305264	1714951	-
3	Naive/None	-	0.0274994	1544897	-
1	CAIX	0;176;175;0;0	0.0345913	1943309	376
2	CAIX	0;176;175;0;0	0.0320322	1799542	283
3	CAIX	0;176;175;0;0	0.0317683	1784718	205
1	NSP14	0;137;119;0;0	0.026666	1498072	16
2	NSP14	0;137;119;0;0	0.0298615	1677593	14
3	NSP14	0;137;119;0;0	0.0288461	1620553	19.2
1	PLAP	2;16;81;47;1	0.0253397	1423566	632
		2;16;79;47;1			591
		1;91;78;96;1			788

2	PLAP	2;16;81;47;1	0.0289435	1626025	553
		2;16;79;47;1			553
		1;91;78;96;1			553
3	PLAP	2;16;81;47;1	0.0358198	2012325	837
		2;16;79;47;1			418
		1;91;78;96;1			642
1	Thrombin	2;84;150;56;2	0.0155248	726813	3419
		2;84;148;56;2			2580
		2;35;78;112;2			2322
2	Thrombin	2;84;150;56;2	0.0125530	587684	3166
		2;84;148;56;2			1333
		2;35;78;112;2			3250
3	Thrombin	2;84;150;56;2	0.0121638	569463	4166
		2;84;148;56;2			3833
		2;35;78;112;2			1000
1	Streptavidin	2;28;34;26;2	0.01618474	757705	6687
		2;28;34;8;2			5500
2	Streptavidin	2;28;34;26;2	0.0178301	834737	7022
		2;28;34;8;2			6011
3	Streptavidin	2;28;34;26;2	0.0144361	675844	5625
		2;28;34;8;2			2847

Table S14: Statistical evaluation of selection hit outcomes compared between 3 library cyclization versions. Calculations are done based on given formulas in Equation 1. Results are shown as average enrichment factors over 3 replicate experiments with standard deviation shown between the 3 results.

Target	Combination	Average Enrichment factor	STDEV between Replicates
Streptavidin open	2;28;34;26;1	14	20
	2;28;34;8;1	68	68
Streptavidin semi-closed	2;28;34;26;2	6444	595
	2;28;34;8;2	4786	1386
Streptavidin closed	2;28;34;26;3-6	190	101
	2;28;34;8;3-6	621	448
PLAP open	2;16;81;47;1	674	119
	2;16;79;47;1	520	74
	1;91;78;96;1	661	96
PLAP semi-closed	2;16;81;47;2	966	705
	2;16;79;47;2	251	354
	1;91;78;96;2	135	121
PLAP closed	2;16;81;47;3-6	23	32
	2;16;79;47;3-6	82	59
	1;91;78;96;3-6	0	0
Thrombin open	2;84;150;56;1	0	0
	2;84;148;56;1	12	6
	2;35;78;112;1	0	0
Thrombin semi-closed	2;84;150;56;2	3583	424
	2;84;148;56;2	2580	1020
	2;35;78;112;2	2190	923
Thrombin closed	2;84;150;56;3-6	130	104
	2;84;148;56;3-6	91	79
	2;35;78;112;3-6	53	75

5.4.3. Library Fingerprints

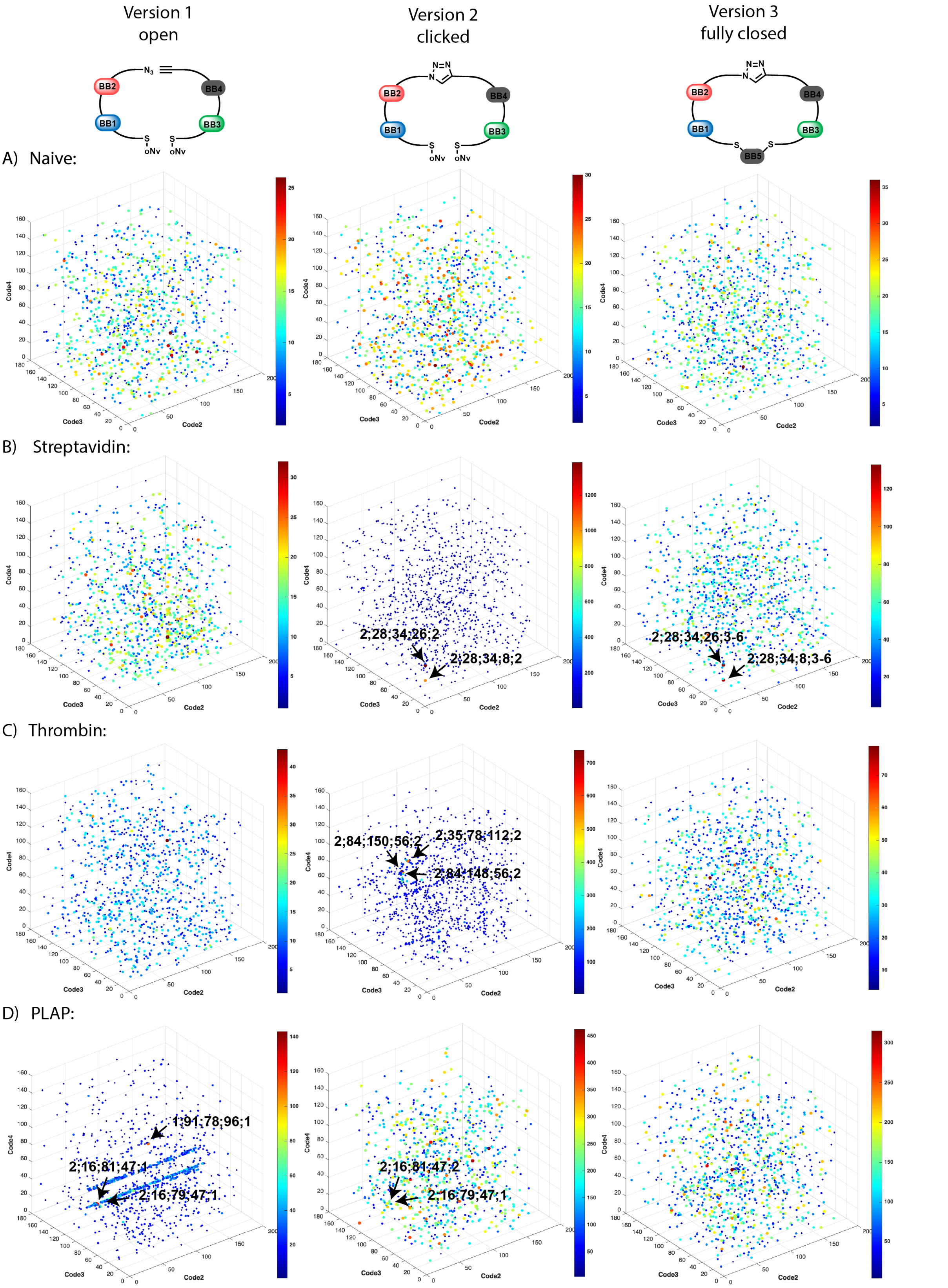

Figure S33: Fingerprints of Naïve library and all non-control selections for concatenated triplicates. Fingerprints show the distinguishing characteristics for different library versions for all proteins. Version 1 fingerprints are taken from selection 1, while version 2-3 selection for A-D are taken from selection 2. Setting max 1000 points per graph.

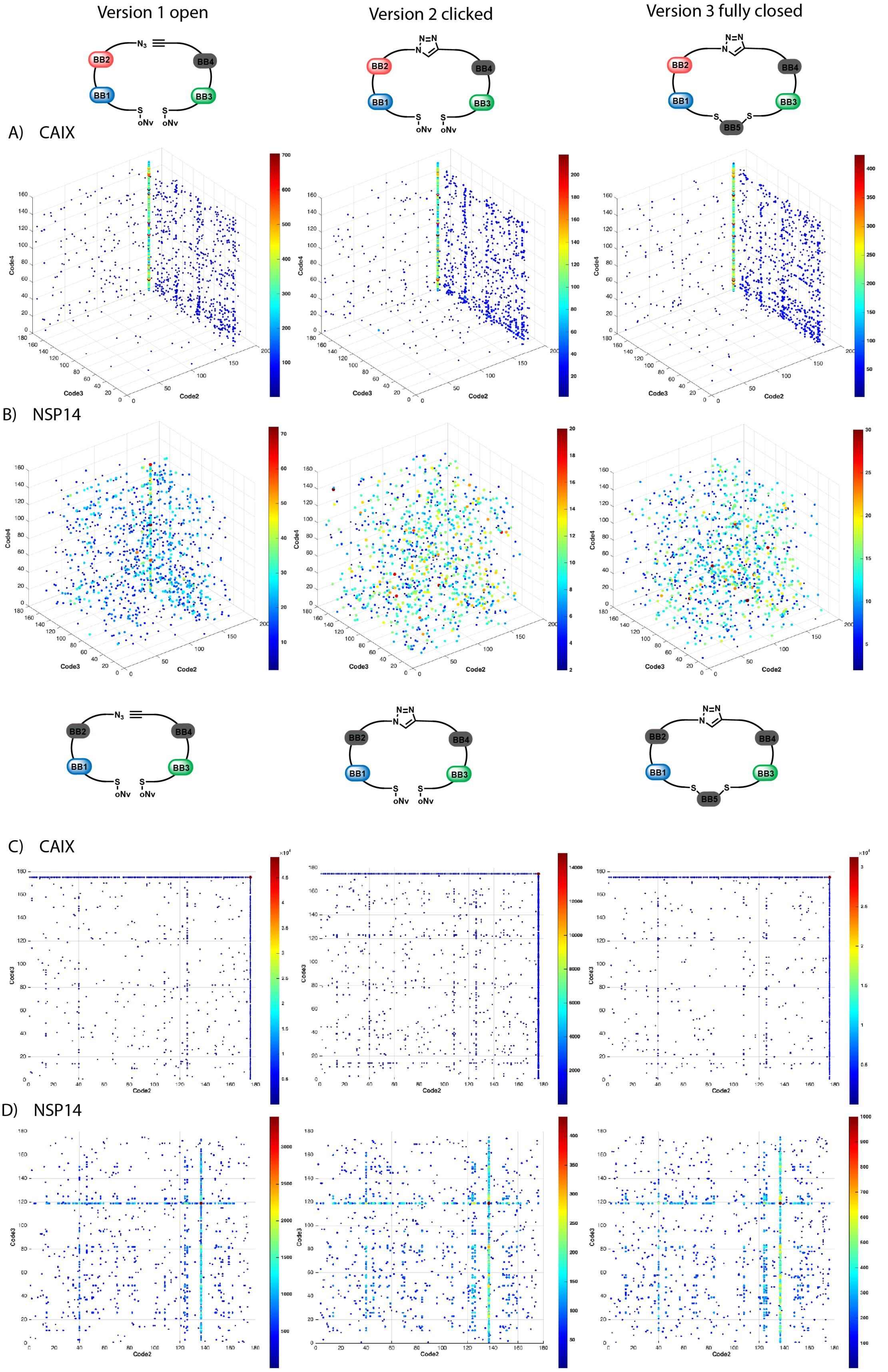

Figure S34: Fingerprints displaying positive control selections displaying different library versions in a 3 BB configuration as well as in 2 building block configurations for the 2 building block positions where the positive control was installed. All fingerprints are from selection 1. Setting max 1000 points per graph.

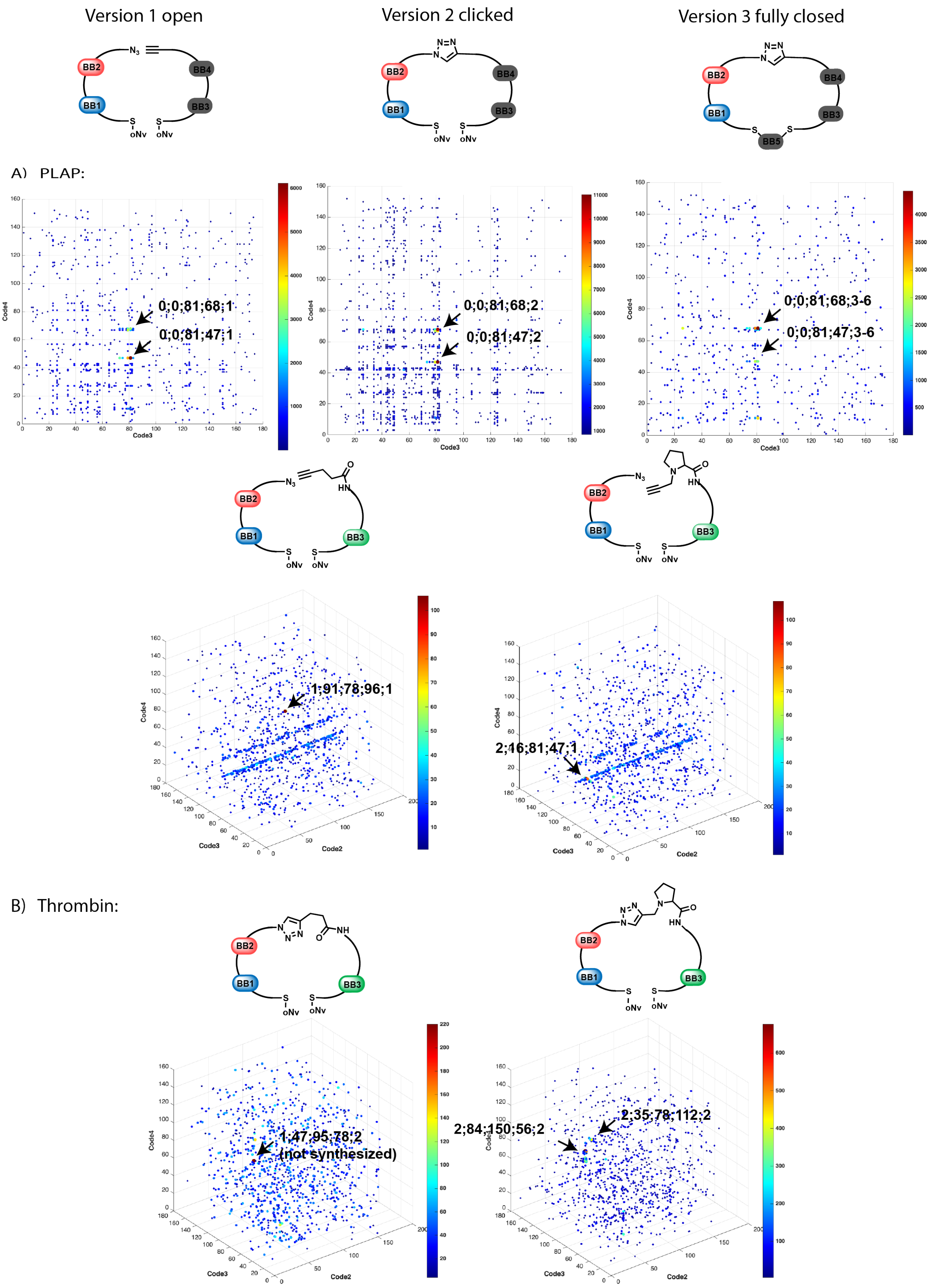

Figure S35: Concatenated triplicate fingerprints showing the 2-BB plane for the major contributing building block positions for the PLAP binder in different library versions. For thrombin different fingerprints are shown according to different selection of BB4 for version 2 of the library. Version 1 fingerprints are taken from selection 1, while version 2-3 selection are taken from selection 2. Setting max 1000 points per graph.

6. Hit Resynthesis

6.1. On-LNA Hit Resynthesis

Table S15: Locked nucleic acid (LNA) sequences used for on-LNA initial hit validation via fluorescence polarization

Name	Sequence	Mass (Da)
LNA5	5'-(Fluorescein dT) AG TAG CC-3'	3322
LNA3	d 5'-GG CTA CTA-3'	2839

The sequences of two complementary LNAs used for hit resynthesis are given in table S15. The LNA5 strand aimed to approximate the HP5 sublibrary during hit resynthesis and the LNA3 the HP3 sublibrary respectively. Scaffold attachment and acetylation of the amino functionality of both strands was performed using the scaffold coupling procedure in section 2.2, scaled down to 50 nmol batches. Acetylated LNA5/LNA3 were used as negative controls during hit resynthesis.

Hit conjugates for PLAP, Thrombin and Streptavidin were synthesized using general procedures on a 50 nmol scale per conjugate. Synthesized LNA conjugates were HPLC purified and precipitated.

If LNA5 and LNA3 conjugates had to participate in an interstrand click reaction, 0.5 nmol of each strand were mixed and the click reaction was performed as described in section 2.2. The reaction was then precipitated, redissolved in 500 µL mQ and centrifuged using an Amicon® Ultra Centrifugal Filter, 3 kDa. The concentrated conjugates were rediluted in mQ and the procedure was repeated 2-3 times in order to remove smaller contaminants.

6.1.1. Negative Control and Scaffold Attachment

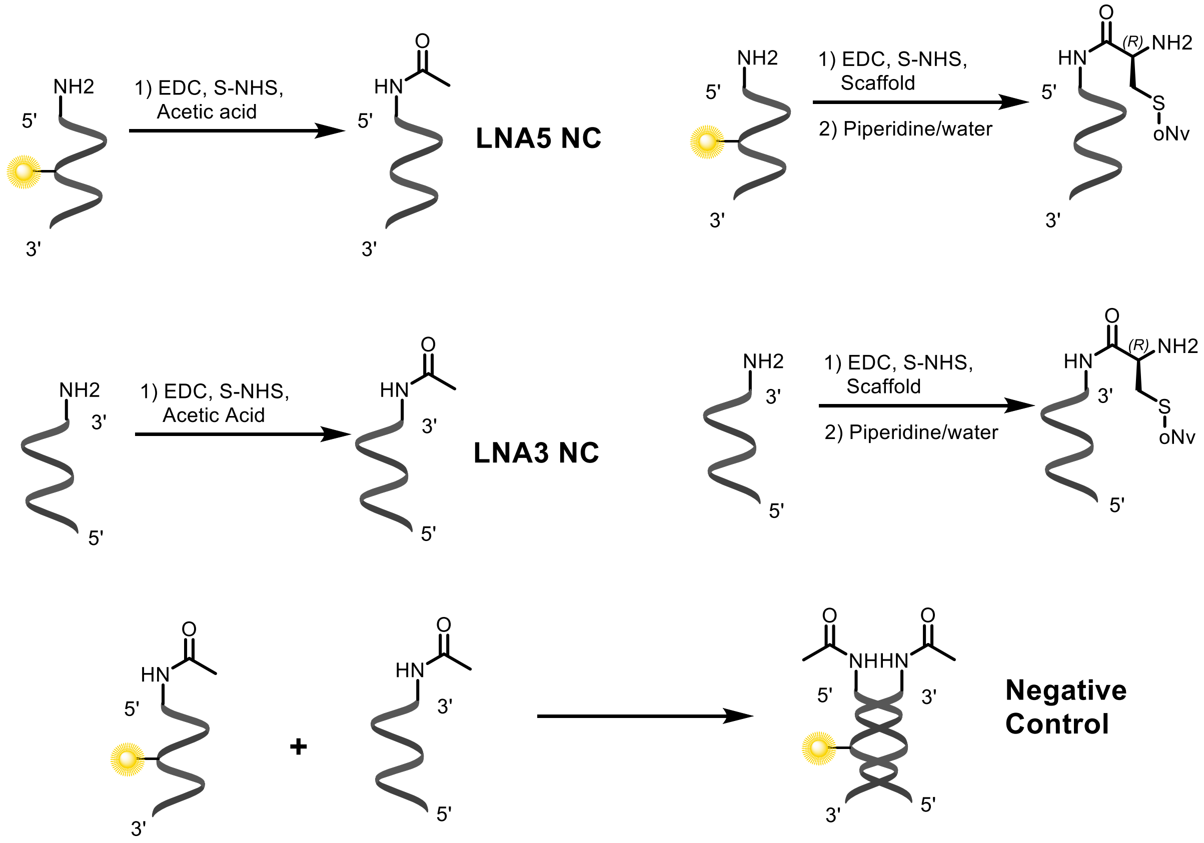

Figure S36: Scaffold attachment scheme and negative control synthesis scheme for LNA conjugates

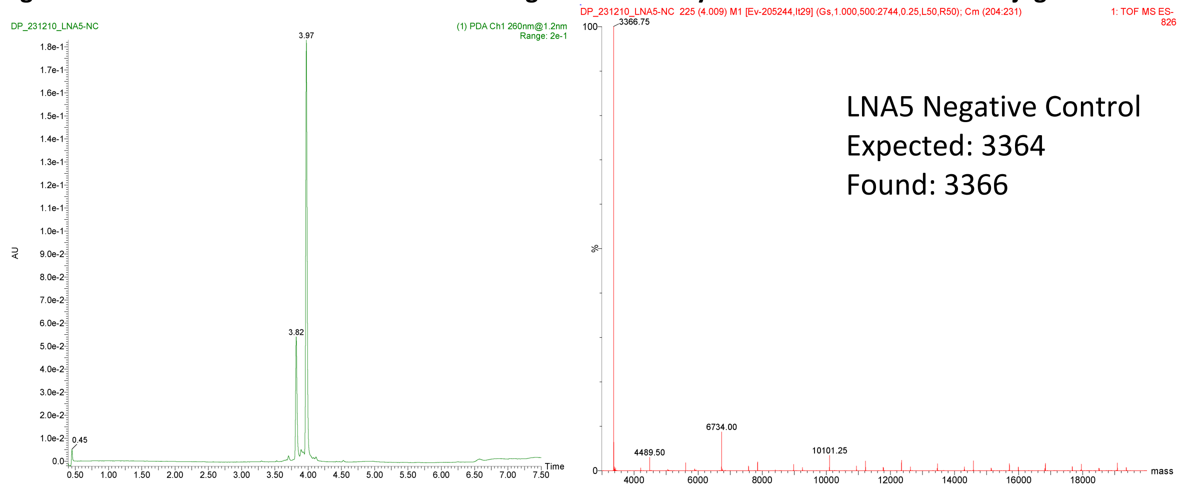

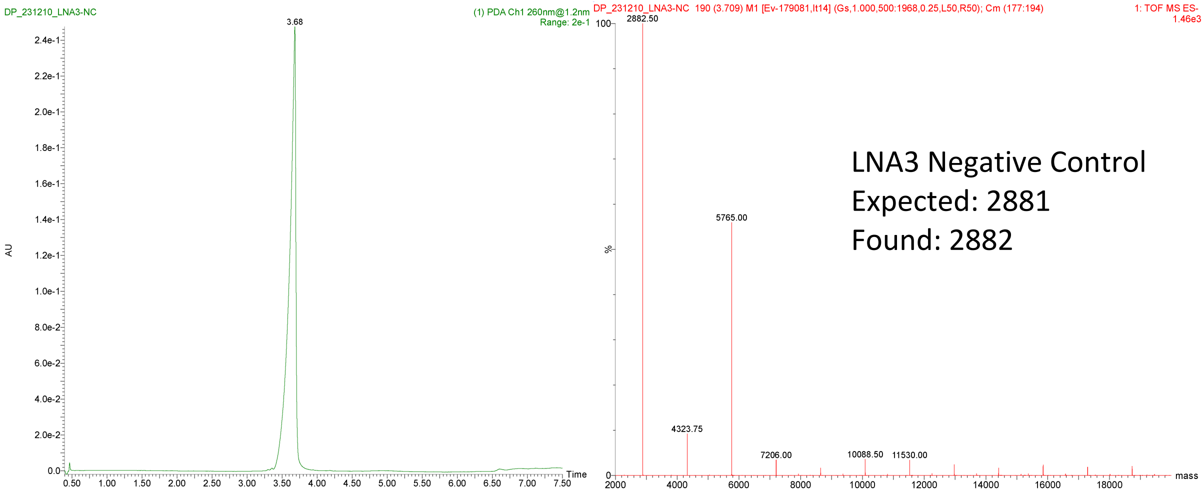

Figure S37: LC-MS chromatogram (left) and the corresponding deconvoluted MS spectra (right) of synthesized LNA negative controls

6.1.2. PLAP

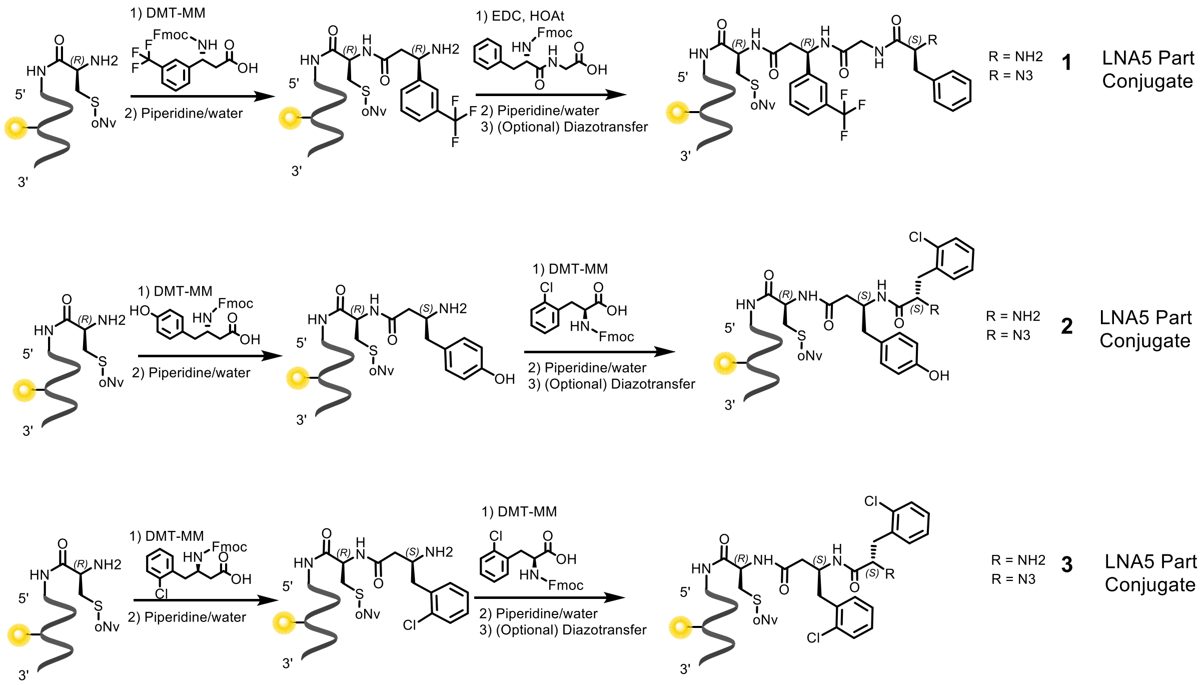

Figure S38: Resynthesis scheme of identified PLAP hits 1-3 on LNA5 strand.

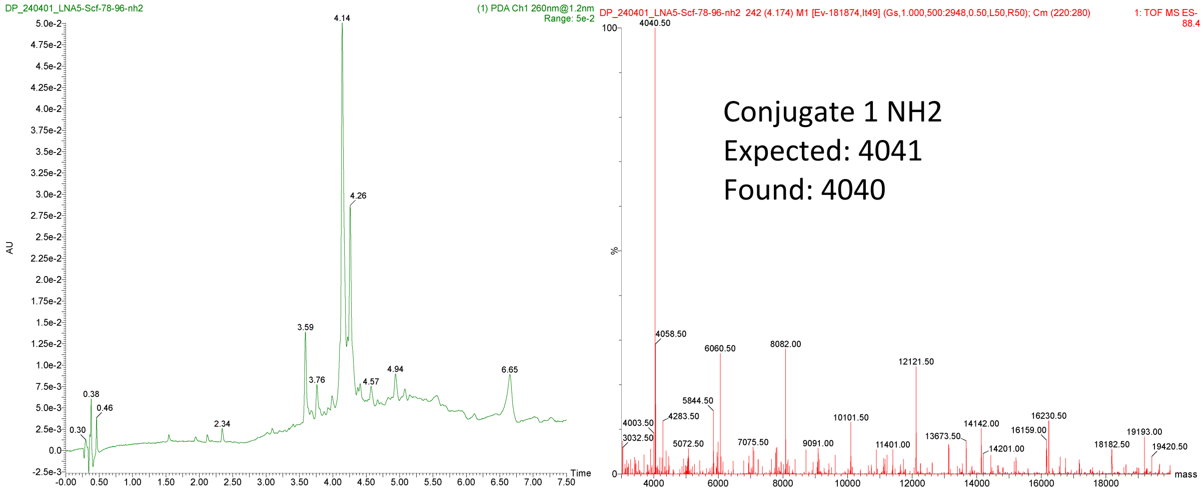
DP_240401_LNA5-Scf-78-96-nh2
(1) PDA Ch1 260nm@1.2nm
Range: 5e-2
DP_240401_LNA5-Scf-78-96-nh2 242 (4.174) M1 [Ev-181874,It49] (Gs,1.000,500:2948,0.50,L50,R50); Cm (220:280)
1: TOF MS ES-
88.4
Conjugate 1 NH2
Expected: 4041
Found: 4040

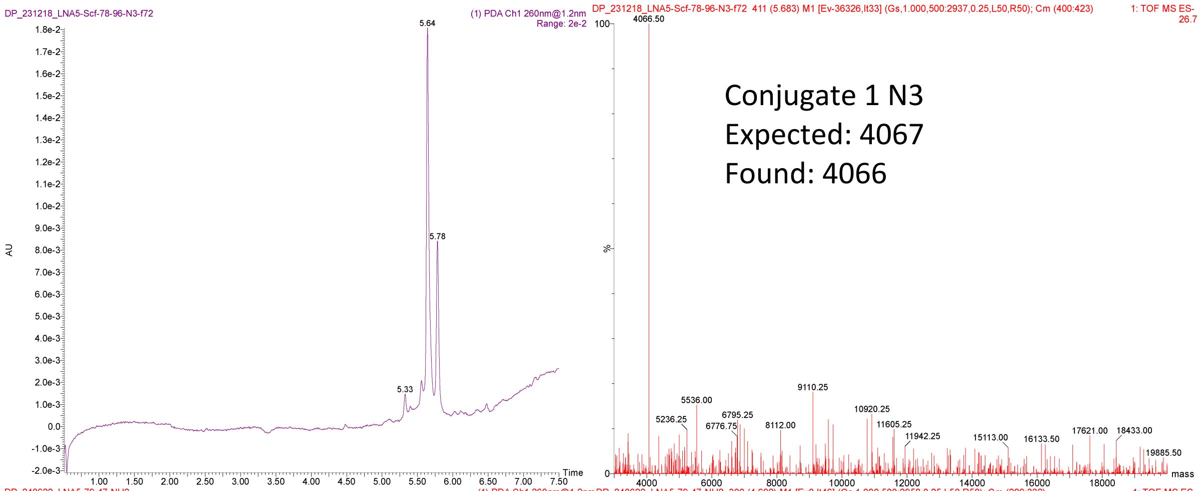
DP_231218_LNA5-Scf-78-96-N3-f72
(1) PDA Ch1 260nm@1.2nm
Range: 2e-2
DP_231218_LNA5-Scf-78-96-N3-f72 411 (5.683) M1 [Ev-36326,It33] (Gs,1.000,500:2937,0.25,L50,R50); Cm (400:423)
1: TOF MS ES-
26.7
Conjugate 1 N3
Expected: 4067
Found: 4066

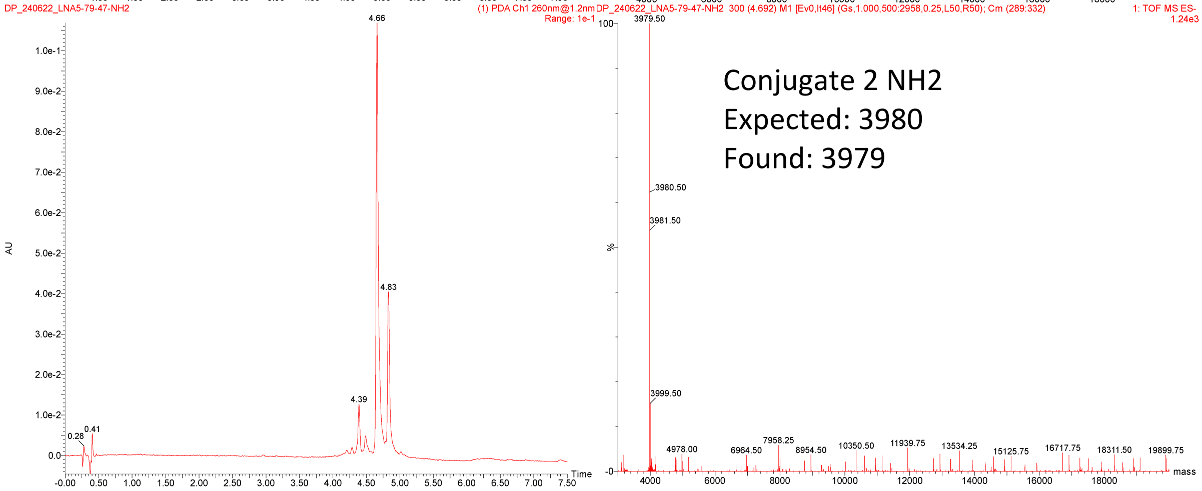
DP_240622_LNA5-79-47-NH2
(1) PDA Ch1 260nm@1.2nm
Range: 1e-1
DP_240622_LNA5-79-47-NH2 300 (4.692) M1 [Ev0,It46] (Gs,1.000,500:2958,0.25,L50,R50); Cm (289:332)
1: TOF MS ES-
1.24e3
Conjugate 2 NH2
Expected: 3980
Found: 3979

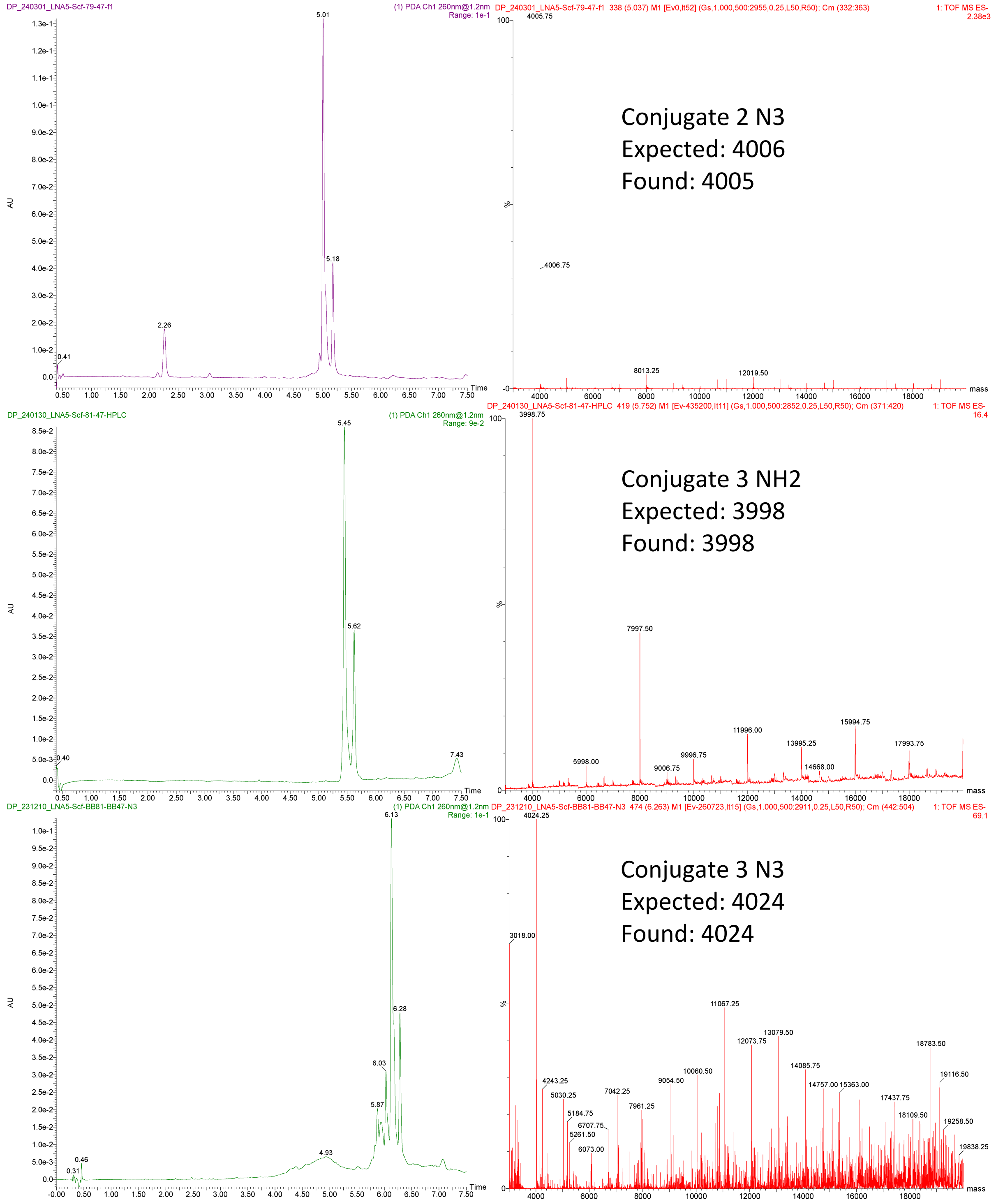

Figure S39: LC-MS chromatogram (left) and the corresponding deconvoluted MS spectra (right) of synthesized PLAP Hit LNA5 conjugates

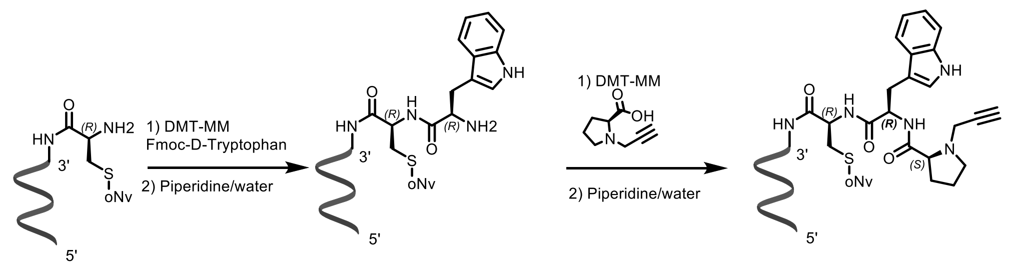

4 LNA3 Part Conjugate

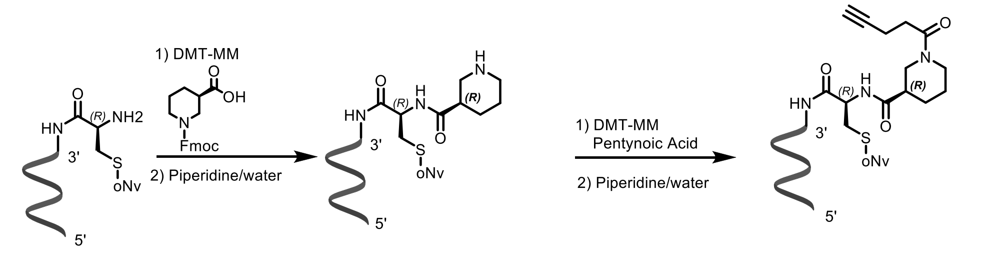

5 LNA3 Part Conjugate

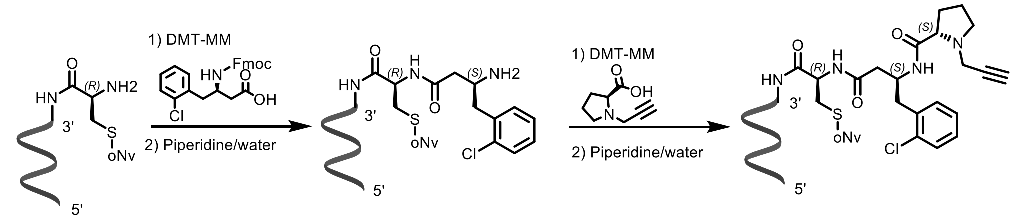

6 LNA3 Part Conjugate

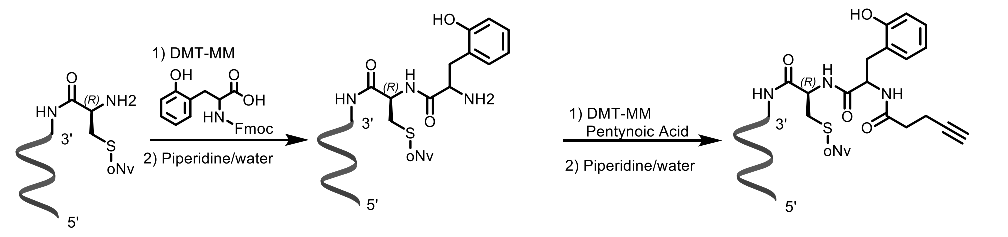

7 LNA3 Part Conjugate

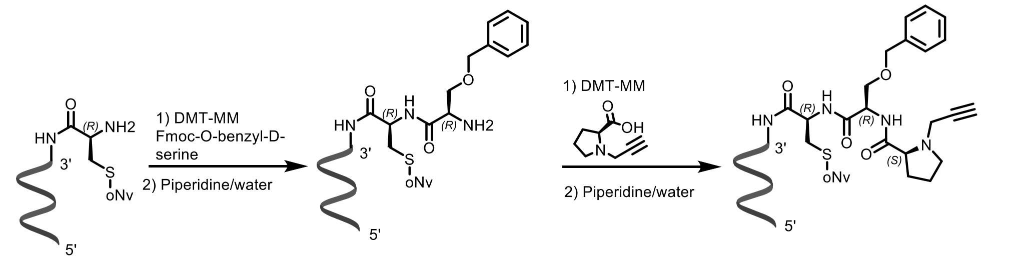

8 LNA3 Part Conjugate

Figure S40: Resynthesis scheme of identified PLAP hits 4-8 on LNA3 strand.

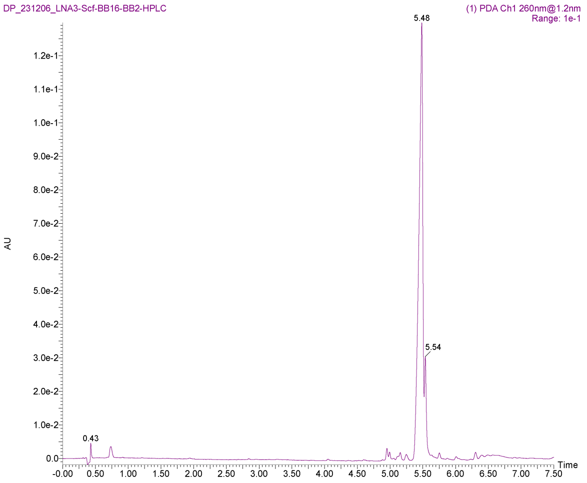
DP_231206_LNA3-Scf-BB16-BB2-HPLC
(1) PDA Ch1 260nm@1.2nm
Range: 1e-1
AU
Time
5.48
5.54
0.43

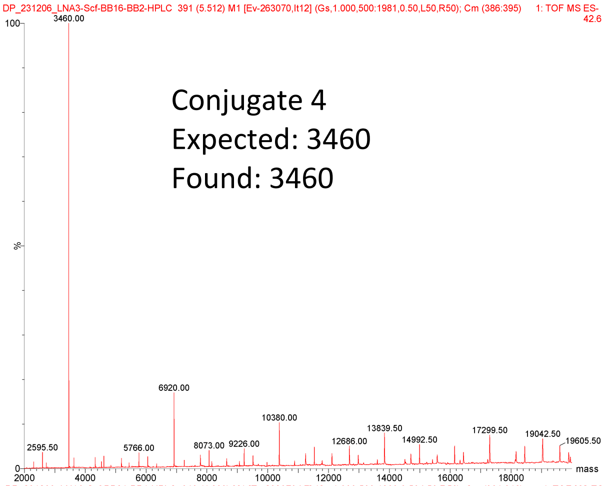
DP_231206_LNA3-Scf-BB16-BB2-HPLC 391 (5.512) M1 [Ev-263070,It12] (Gs,1.000,500:1981,0.50,L50,R50); Cm (386:395)
1: TOF MS ES-
42.6
%
mass
3460.00
6920.00
2595.50
5766.00
8073.00
9226.00
10380.00
12686.00
13839.50
14992.50
17299.50
19042.50
19605.50
Conjugate 4
Expected: 3460
Found: 3460

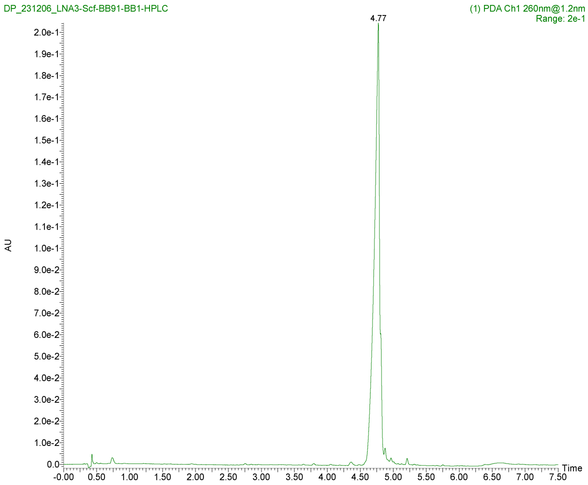
DP_231206_LNA3-Scf-BB91-BB1-HPLC
(1) PDA Ch1 260nm@1.2nm
Range: 2e-1
AU
Time
4.77

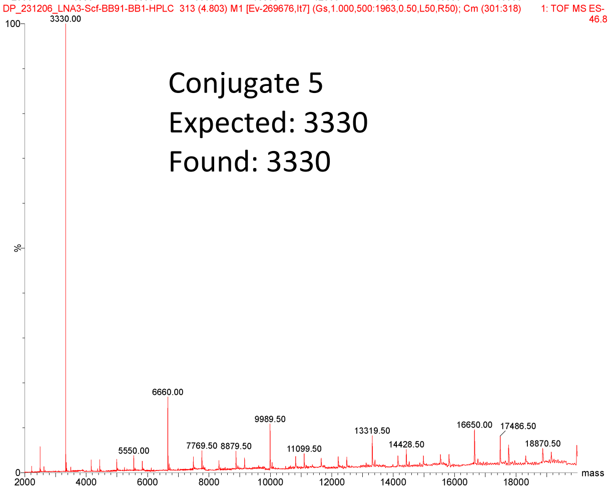
DP_231206_LNA3-Scf-BB91-BB1-HPLC 313 (4.803) M1 [Ev-269676,It7] (Gs,1.000,500:1963,0.50,L50,R50); Cm (301:318)
1: TOF MS ES-
46.8
%
mass
3330.00
6660.00
5550.00
7769.50
8879.50
9989.50
11099.50
13319.50
14428.50
16650.00
17486.50
18870.50
Conjugate 5
Expected: 3330
Found: 3330

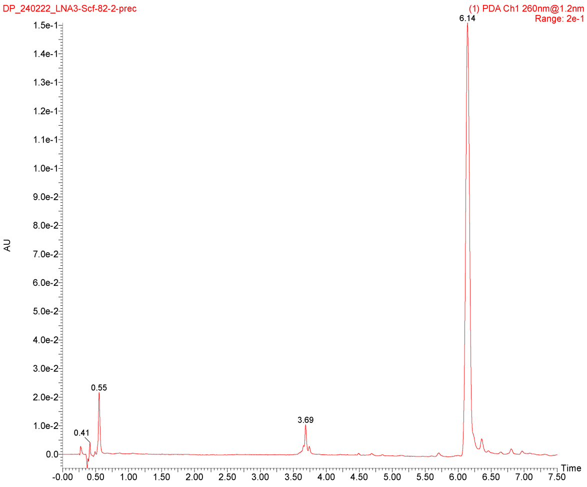
DP_240222_LNA3-Scf-82-2-prec
(1) PDA Ch1 260nm@1.2nm
Range: 2e-1
AU
Time
6.14
3.69
0.55
0.41

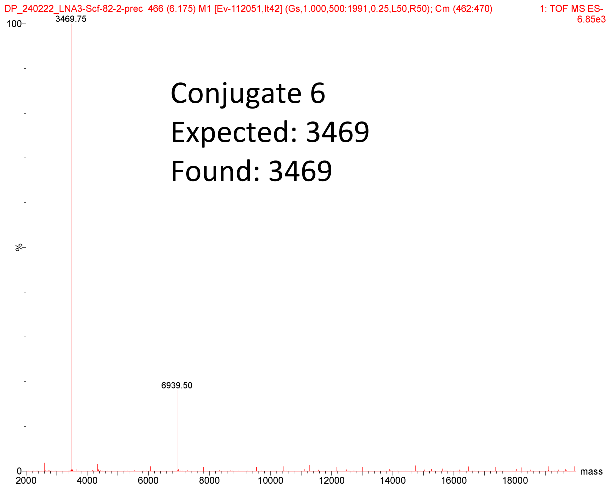
DP_240222_LNA3-Scf-82-2-prec 466 (6.175) M1 [Ev-112051,It42] (Gs,1.000,500:1991,0.25,L50,R50); Cm (462:470)
1: TOF MS ES-
6.85e3
%
mass
3469.75
6939.50
Conjugate 6
Expected: 3469
Found: 3469

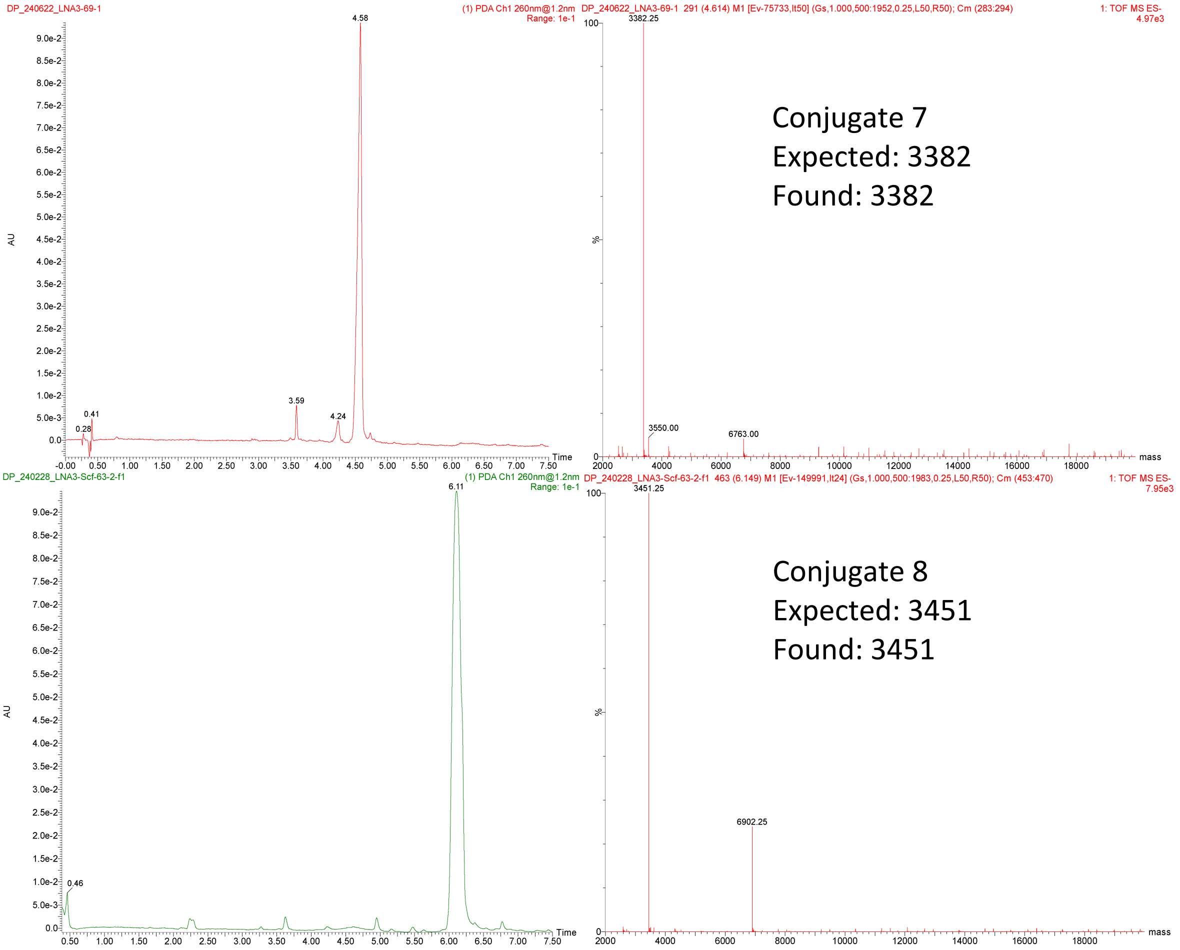

Figure S41: LC-MS chromatogram (left) and the corresponding deconvoluted MS spectra (right) of synthesized PLAP Hit LNA3 conjugates

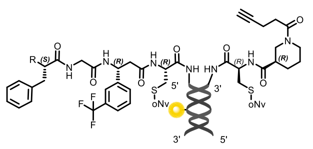

1 + 5

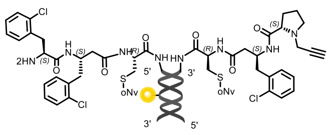

3 + 6

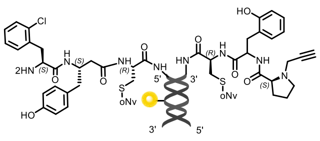

2 + 7

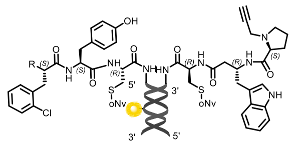

2 + 4

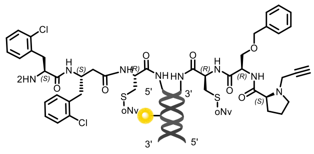

3 + 8

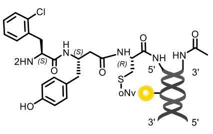

2 + LNA3 NC

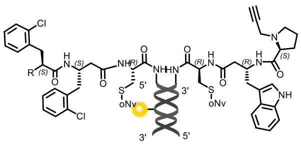

3 + 4

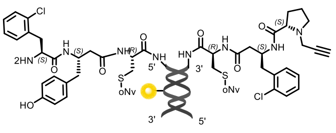

2 + 6

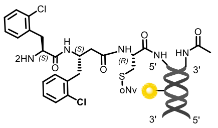

3 + LNA3 NC

Figure S42: Hybridization of PLAP conjugates 1-3 with conjugates 4-8 as well as negative controls as finally tested in fluorescence polarization experiments.

To generate the maleimide linked hits in figure S43, 0.5 nmol of conjugate 3 and 6 respectively were mixed and reacted with 1,1'-(ethane-1,2-diyl)bis(1H-pyrrole-2,5-dione) (Maleimide 2), 1,1'-(pentane-1,5-diyl)bis(1H-pyrrole-2,5-dione) (Maleimide 6) or 1,1'-((ethane-1,2-diylbis(oxy))bis(ethane-2,1-diyl))bis(1H-pyrrole-2,5-dione) (Maleimide 8) according the thioether formation protocol in section 2.2. The conjugates were then precipitated, redissolved in mQ and concentrated & rediluted 3x with Amicon® Ultra Centrifugal Filter, 3 kDa.

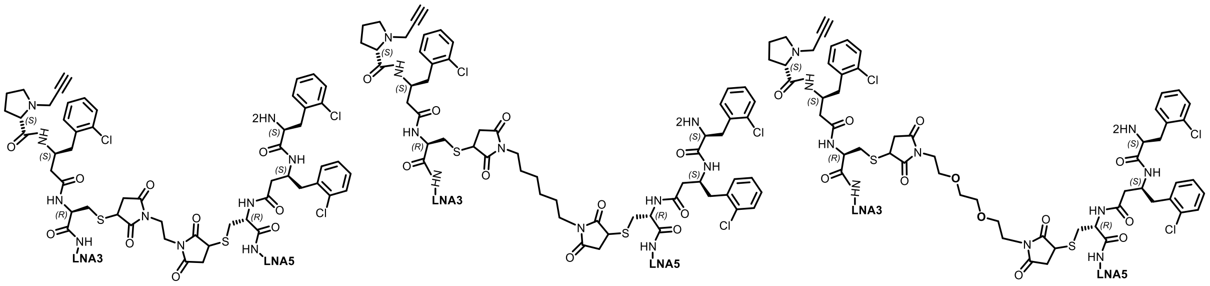

3 + 6
Maleimide 2

3 + 6
Maleimide 6

3 + 6
Maleimide 8

Figure S43: Exploring variable length maleimide linkers for PLAP conjugates 3 and 6 for testing in fluorescence polarization experiments.

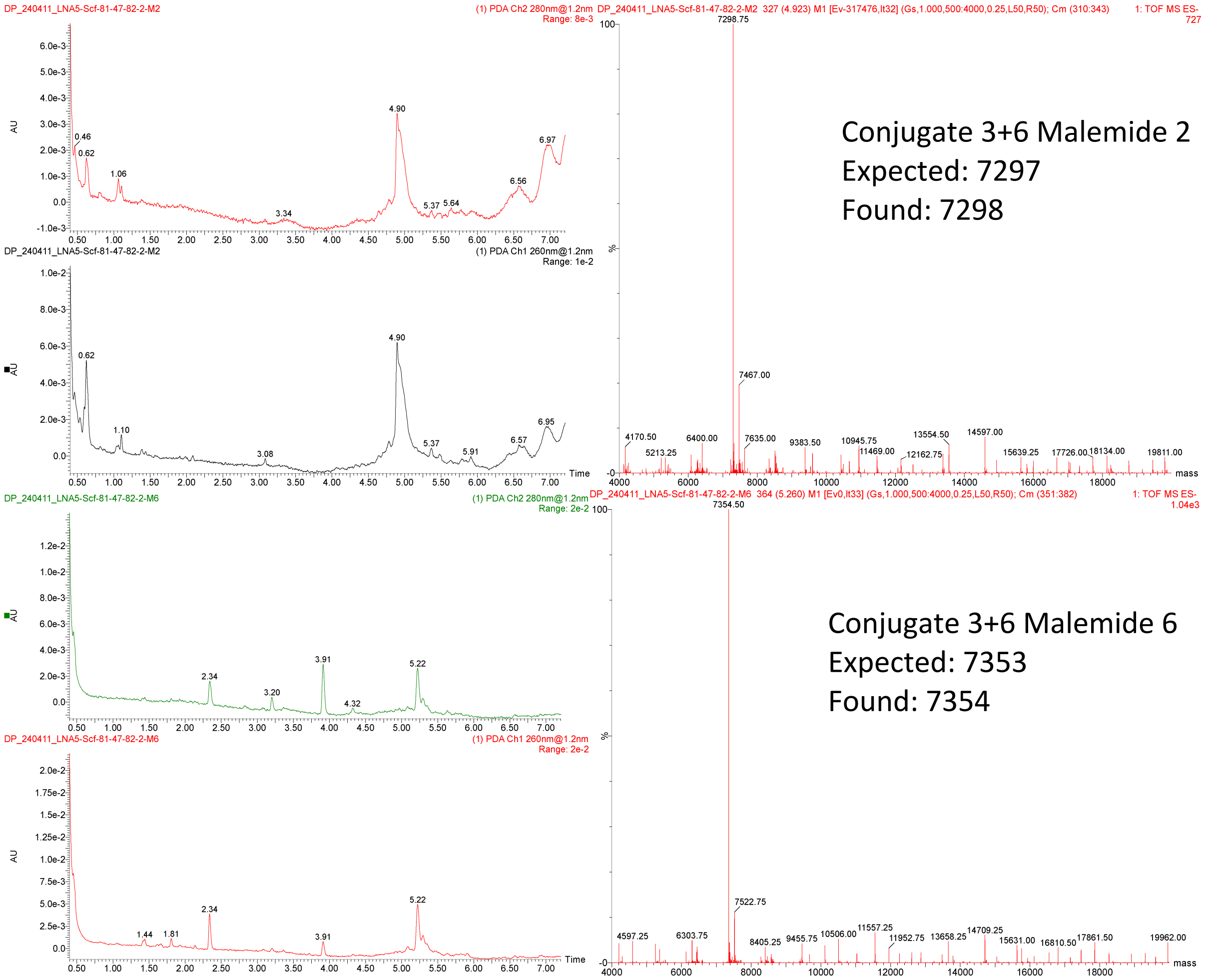

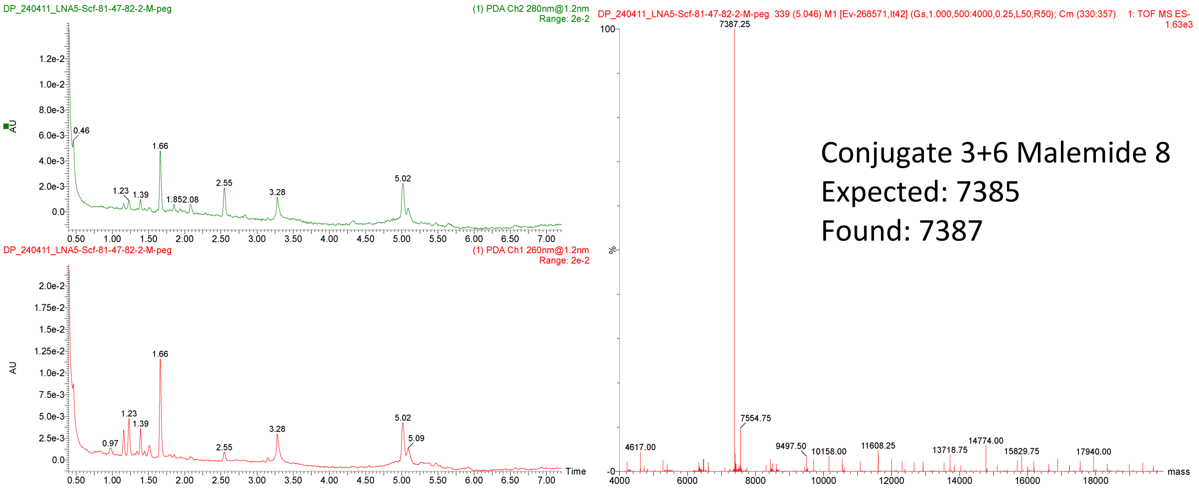

Figure S44: LC-MS chromatogram (left) and the corresponding deconvoluted MS spectra (right) of linked PLAP hits 3 and 6. LC chromatogram provided as a 260/280 nm ratio due to low intensity of product peak, making it possible to distinguish it from non-LNA contaminants.

6.1.3. Thrombin

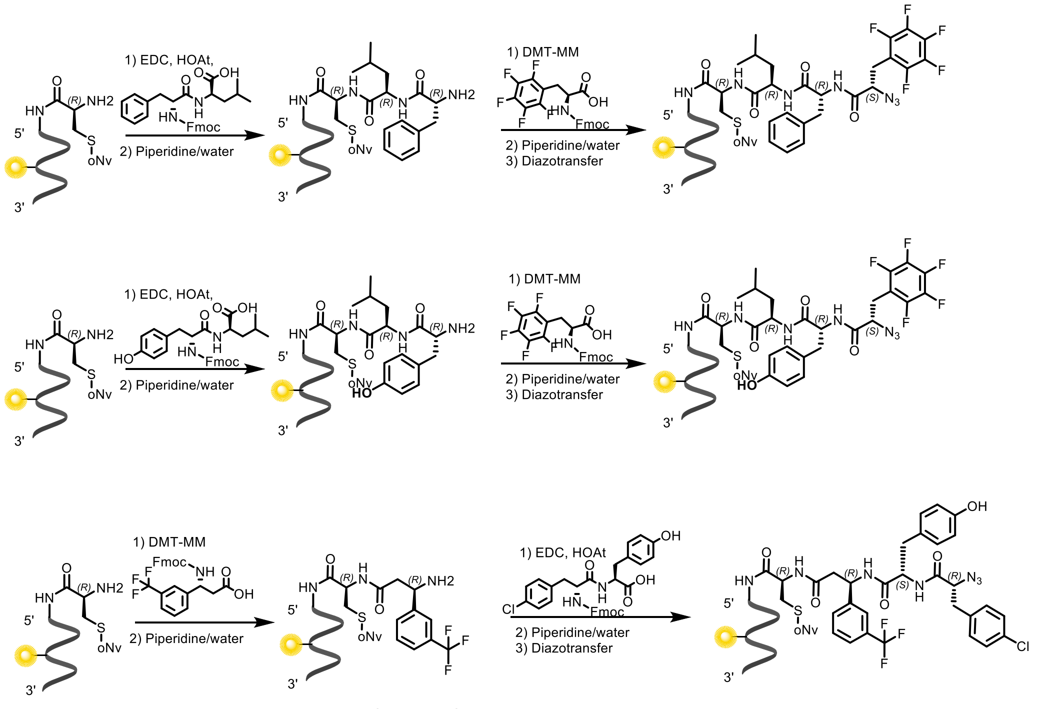

9 LNA5 Part Conjugate

10 LNA5 Part Conjugate

11 LNA5 Part Conjugate

Figure S45: Resynthesis scheme of identified Thrombin hits 9-11 on LNA5 strand.

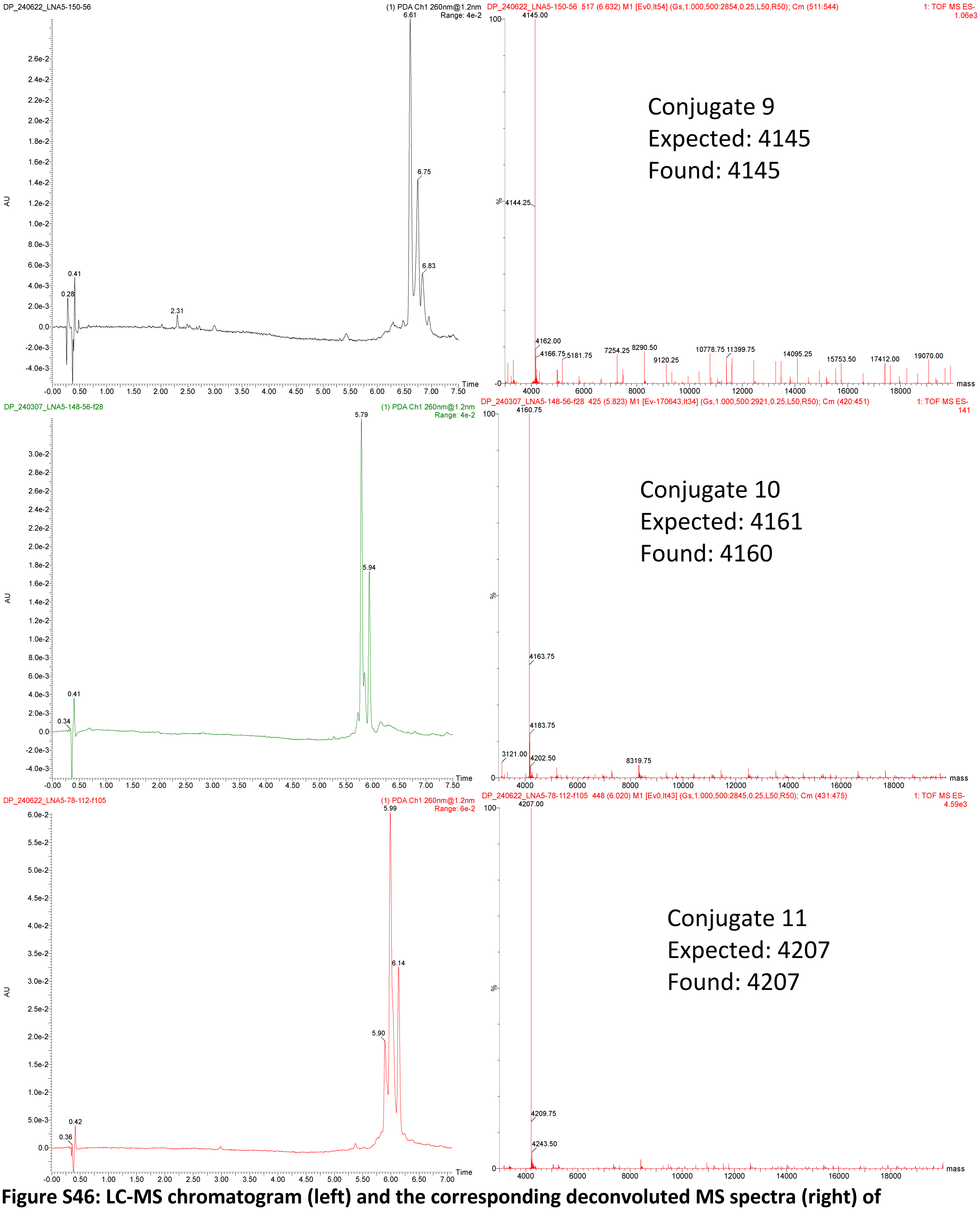

Figure S46: LC-MS chromatogram (left) and the corresponding deconvoluted MS spectra (right) of synthesized Thrombin Hit LNA5 conjugates

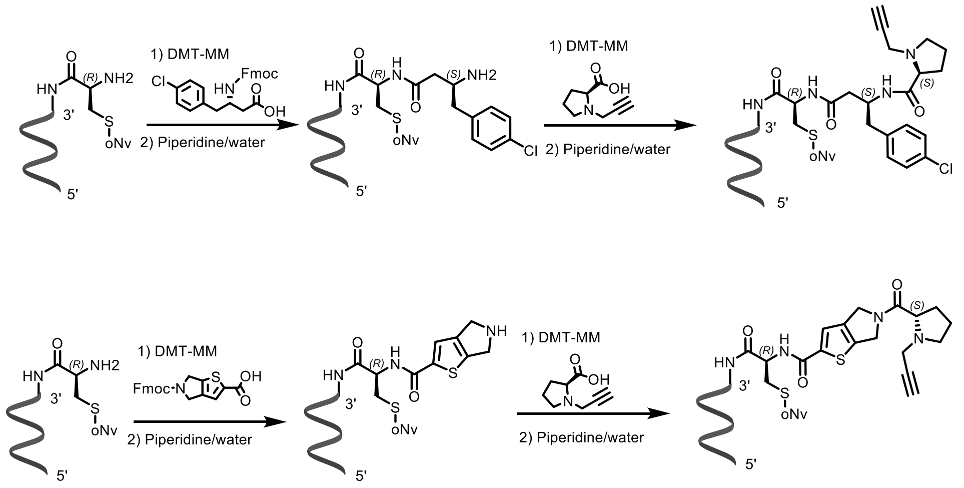

12 LNA3 Part Conjugate

13 LNA3 Part Conjugate

Figure S47: Resynthesis scheme of identified Thrombin hits 12-13 on LNA3 strand.

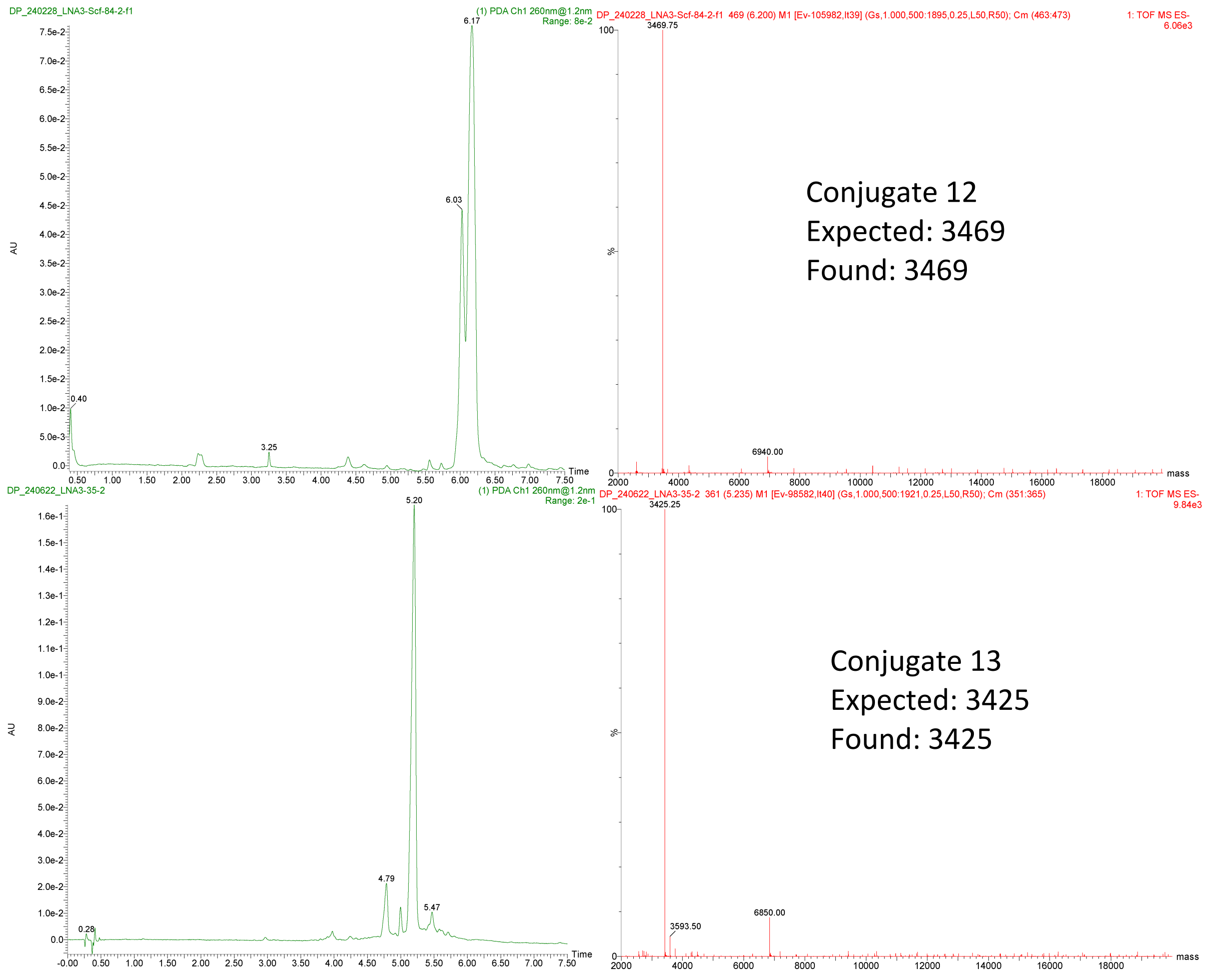

Figure S48: LC-MS chromatogram (left) and the corresponding deconvoluted MS spectra (right) of synthesized Thrombin Hit LNA3 conjugates

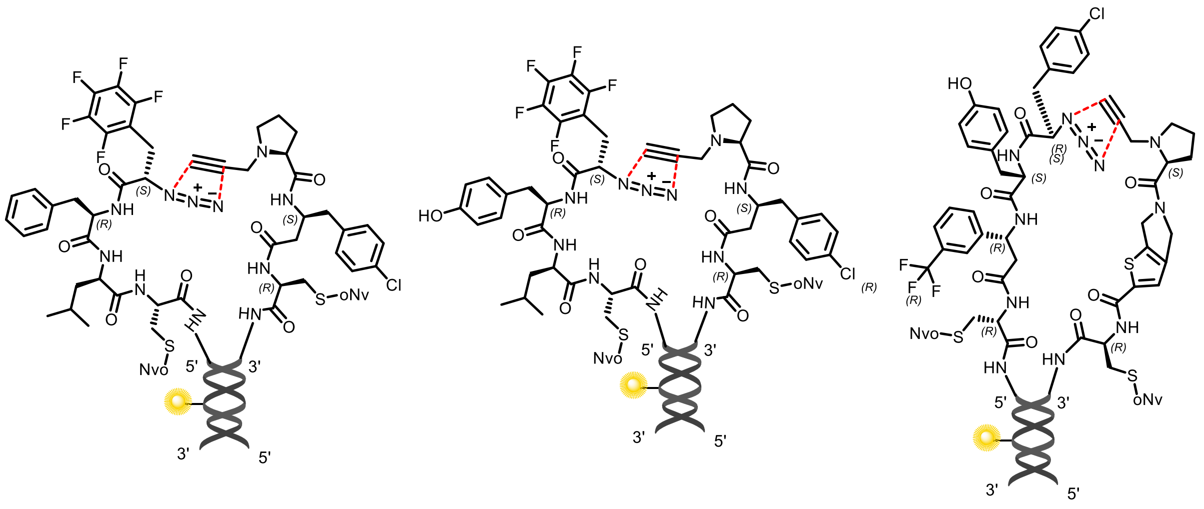

9 + 12 **10 + 12** **11 + 13**

Figure S49: Hybridization and click reaction of Thrombin conjugates 9-11 with conjugates 12-13 as finally tested in fluorescence polarization experiments.

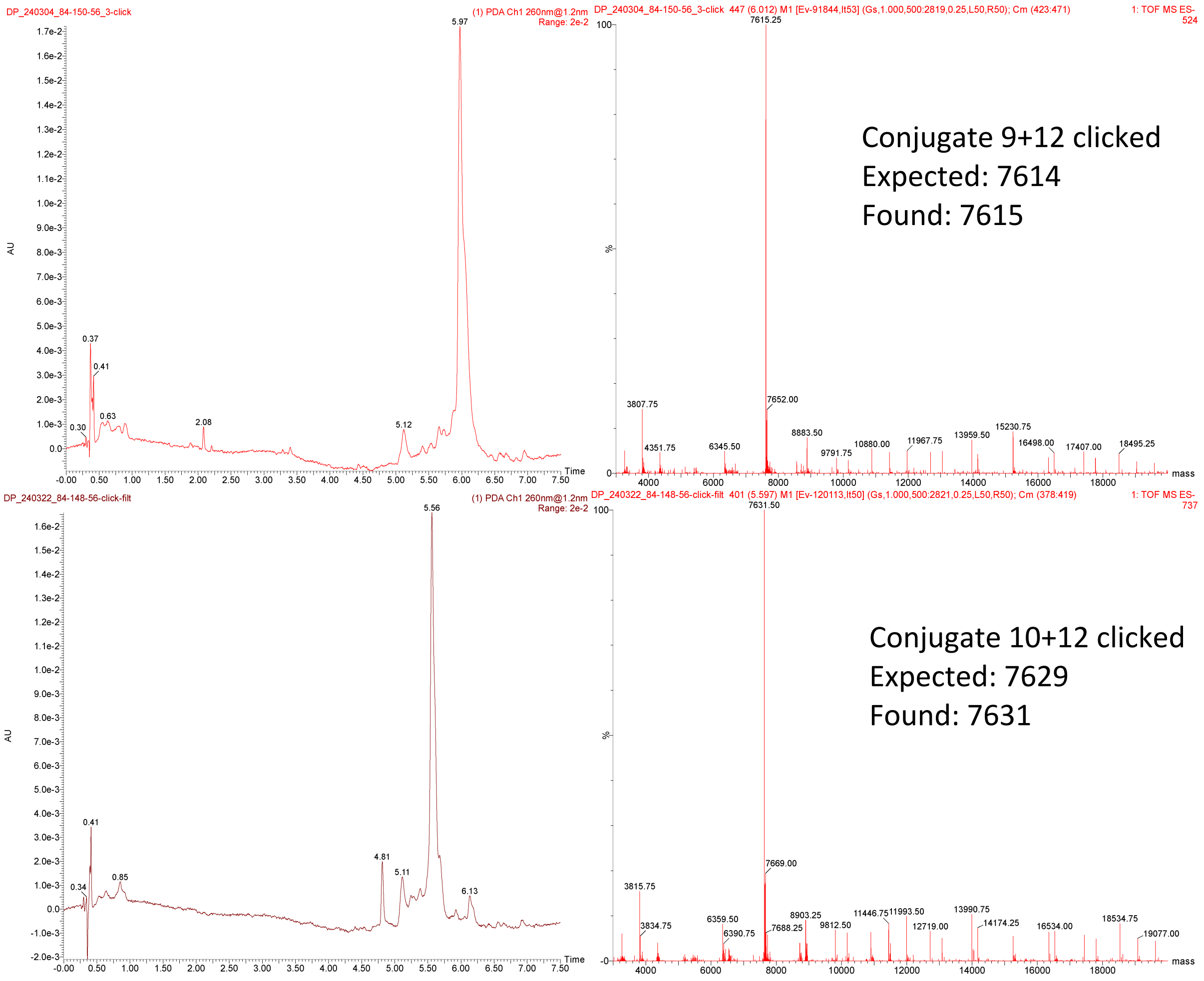

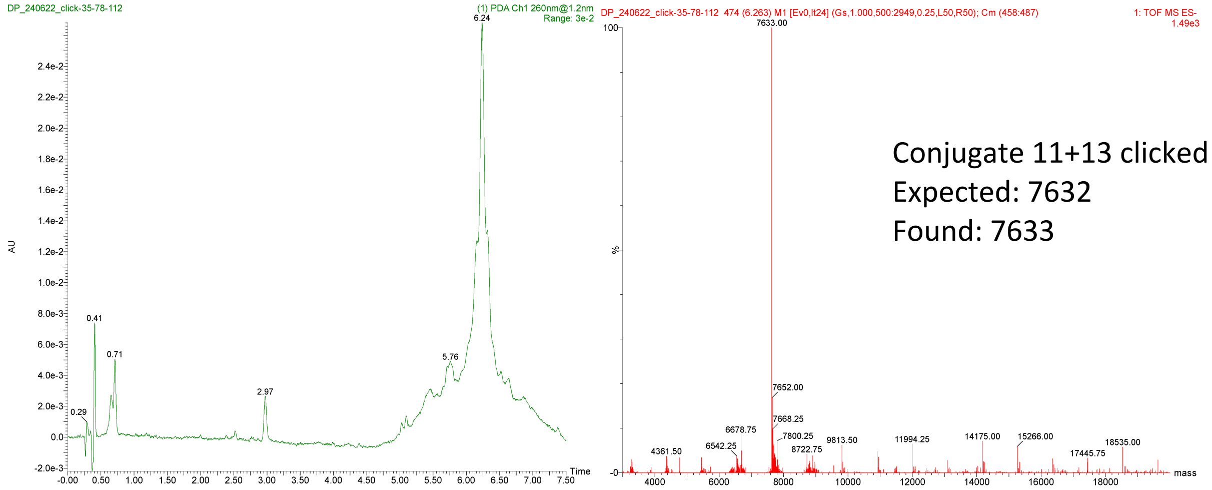

Figure S50: LC-MS chromatogram (left) and the corresponding deconvoluted MS spectra (right) of hybridized and clicked Thrombin LNA5 & LNA3 conjugates

6.1.4. Streptavidin

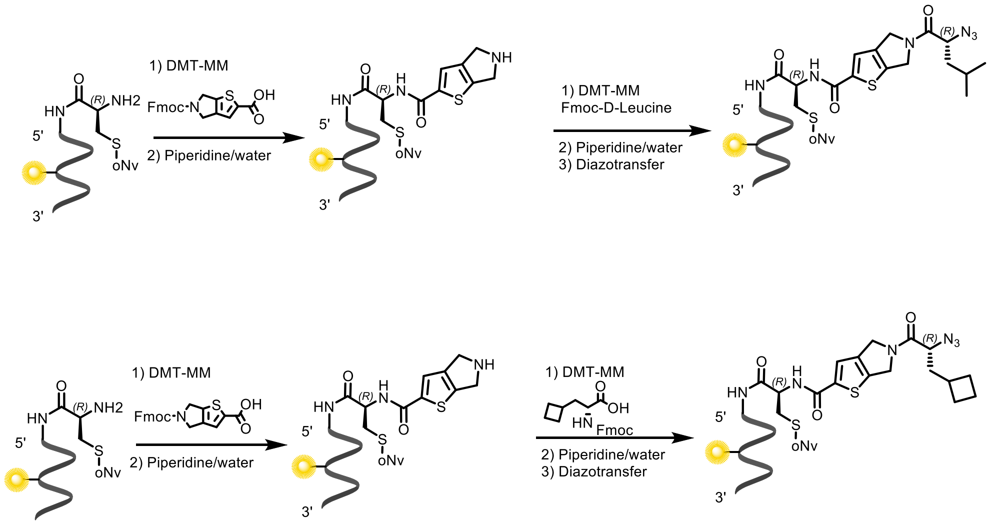

14 LNA5 Part Conjugate

15 LNA5 Part Conjugate

Figure S51: Resynthesis scheme of identified Streptavidin hits 14-15 on LNA5 strand.

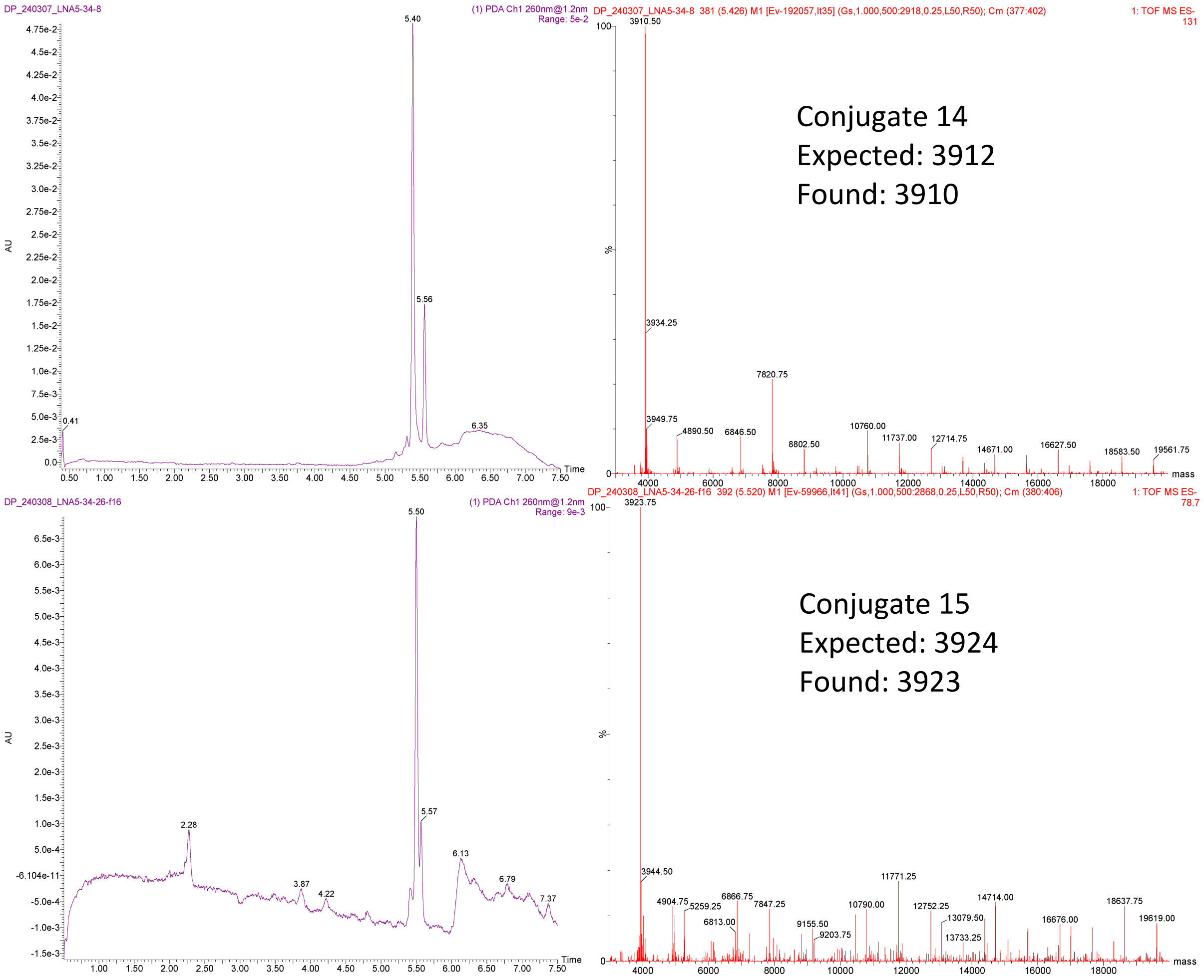

Figure S52: LC-MS chromatogram (left) and the corresponding deconvoluted MS spectra (right) of synthesized Streptavidin Hit LNA5 conjugates

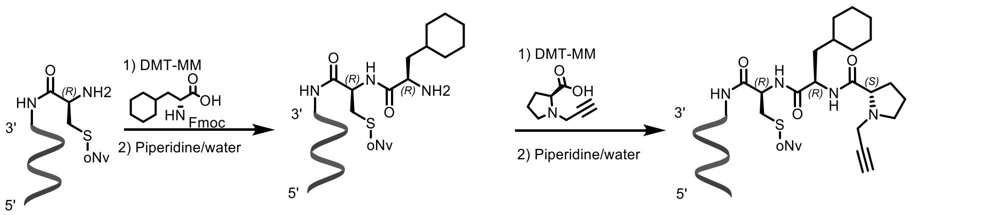

16 LNA3 Part Conjugate

Figure S53: Resynthesis scheme of identified Streptavidin hit 16 on LNA3 strand.

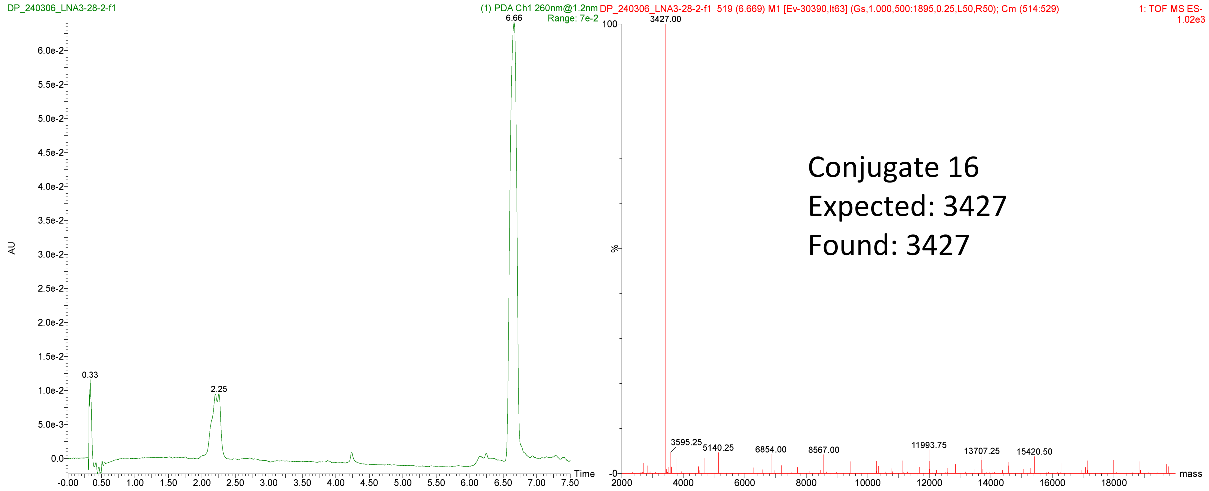

Figure S54: LC-MS chromatogram (left) and the corresponding deconvoluted MS spectra (right) of synthesized Streptavidin Hit LNA3 conjugate

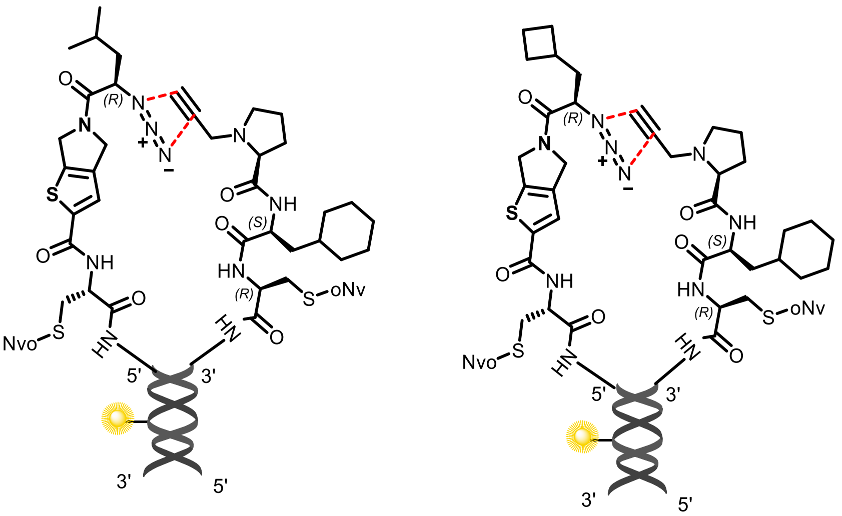

14 + 16 **15 + 16**

Figure S55: Hybridization and click reaction of Streptavidin conjugates 14-15 with conjugate 16 as finally tested in fluorescence polarization experiments.

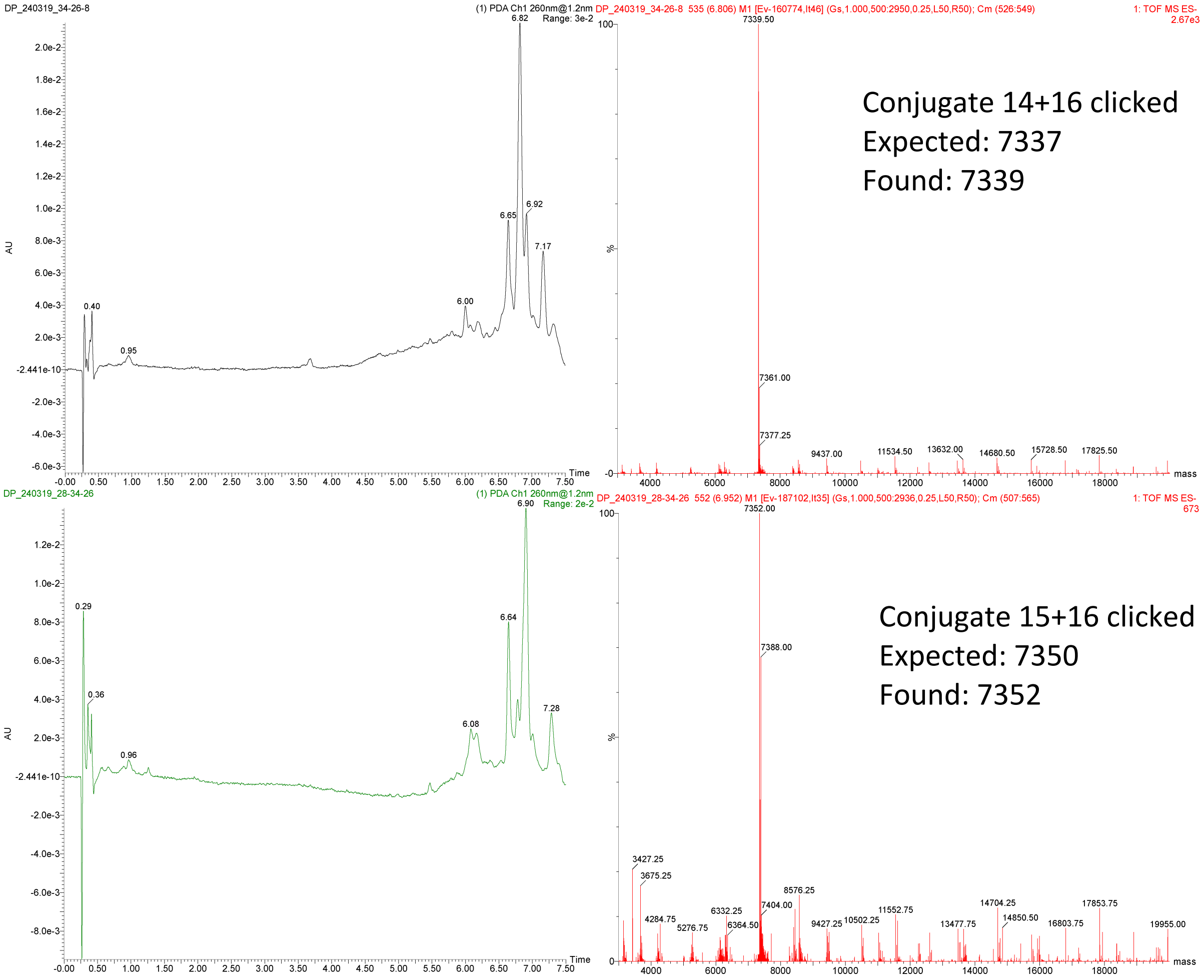

Figure S56: LC-MS chromatogram (left) and the corresponding deconvoluted MS spectra (right) of hybridized and clicked Streptavidin LNA5 & LNA3 conjugates

6.2. Off-DNA Hit Resynthesis

6.2.1. Absorbance-based Concentration Determination of Fluorescein Labeled Binders

Compound **41** was dissolved in a 1:1 DMSO: PBS solution and diluted to the concentrations given in table S16. Using the UV-Vis setting on a nanodrop spectrophotometer, absorbance at 507nm was recorded for the different dilutions. The linear correlation between absorbance & concentration was calculated and the slope equation was used to calculate concentration based on absorbance for all fluorescein labeled hits.

Table S16: Concentration and corresponding absorbance of a DMSO/PBS solution of 41.

Concentration (µM)	Absorbance (507nm)
**200**	**1.34**
**120**	**0.85**
**60**	**0.43**
**30**	**0.217**
**15**	**0.114**

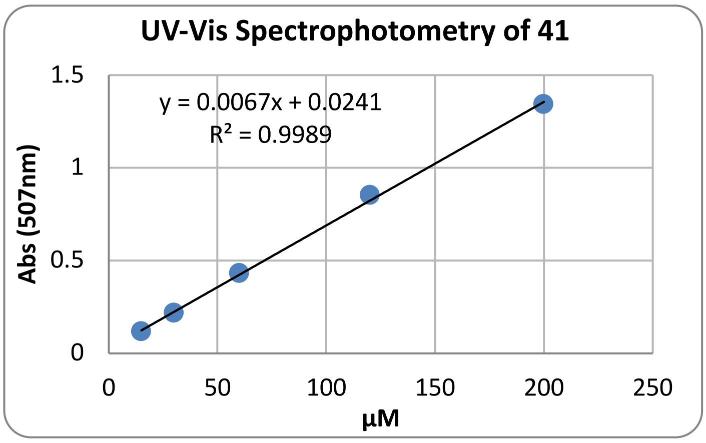

Figure S57: Linear correlation between absorbance and concentration of 41

6.2.2. General Synthesis Procedure

Tentagel® S NH2 base resin in 1.5g batches was modified according to the scheme in figure S58. Consequently, Rink Amide modified resin was used for hit resynthesis. All hits were resynthesized using the products of two separate solid phase syntheses, where the products of both are covalently linked in one of the latter synthesis steps. Thus, some synthesis schemes are termed "resin 1" and "resin 2", denoting the separate resins used for the convergent synthesis of a single final product. Protocols used for resynthesis of hits are given in section 2.3.

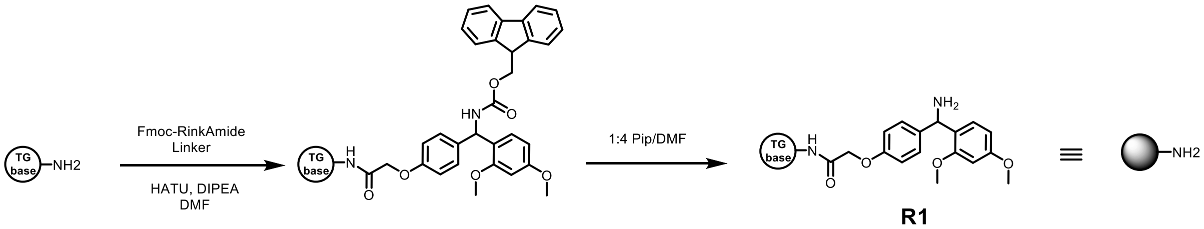

R1

Figure S58: Tentagel® base resin preparation for resynthesis of hit. Fmoc-Rink Amide linker was installed on base resin prior to starting synthesis of conjugates

6.2.3. Thrombin (Compounds 19-24)

Resin 1

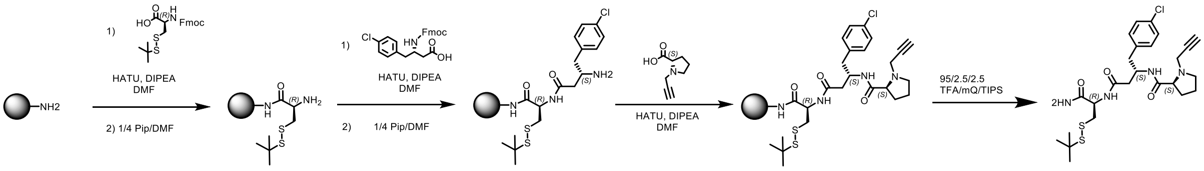

17

Synthesis of 17: 500 mg of resin **R1** was reacted in repeating amidation and Fmoc deprotection cycles with Fmoc-Cys(StBu)-OH, (S)-3-((((9H-fluoren-9-yl)methoxy)carbonyl)amino)-4-(4-chlorophenyl)butanoic acid and prop-2-yn-1-yl-L-proline

to generate intermediate **17.** The intermediate was cleaved off the resin, precipitated and used as is for resin 2 synthesis of **18.**

Compound **17** ESI-MS: [M+H]+ 539.15 g/mol , found 539.12

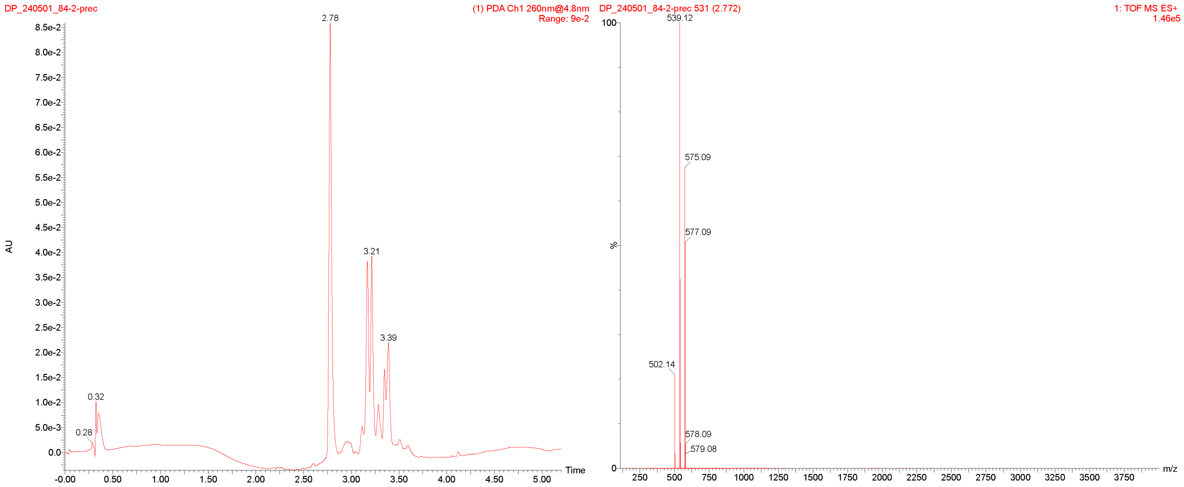

Figure S59: LC-MS chromatogram (left) and the corresponding deconvoluted MS spectra (right) of compound 17

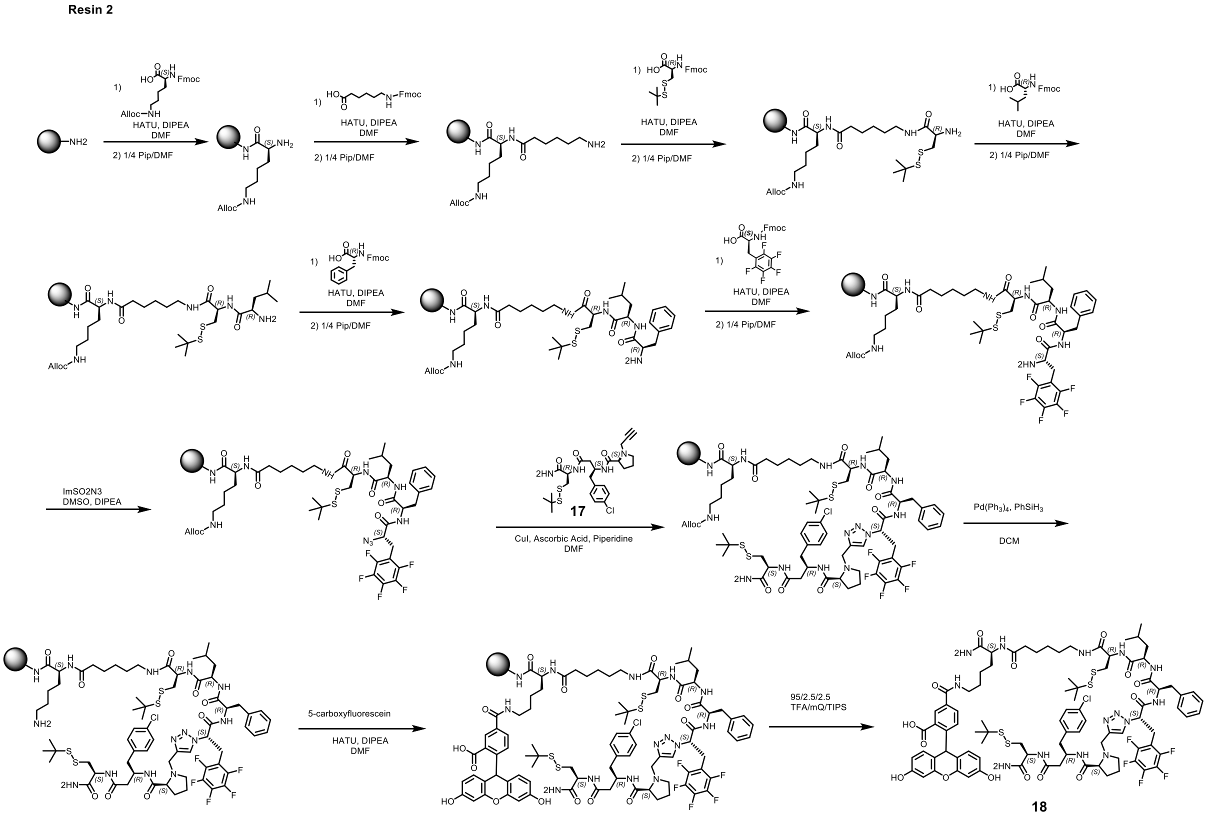

Synthesis of 18: 500 mg of resin **R1** was reacted in repeating amidation and Fmoc deprotection cycles with Fmoc-Lys(Alloc)-OH, Fmoc-6-Ahx-OH, Fmoc-Cys(StBu)-OH, Fmoc-D-Leucine, Fmoc-D-Phenylalanine and Fmoc-Phe(F5)-OH. The terminal amino group was then subjected to a diazotransfer reaction and reacted via CuAAC with intermediate **17**. The

lysine Alloc group was then deprotected and 5-carboxyfluorescein was attached to afford compound **18,** which was cleaved off the resin and HPLC purified.

Compound **18** (5mg, 2% yield) ESI-MS: [M+H]+ 1870.66 g/mol , found 1871.66

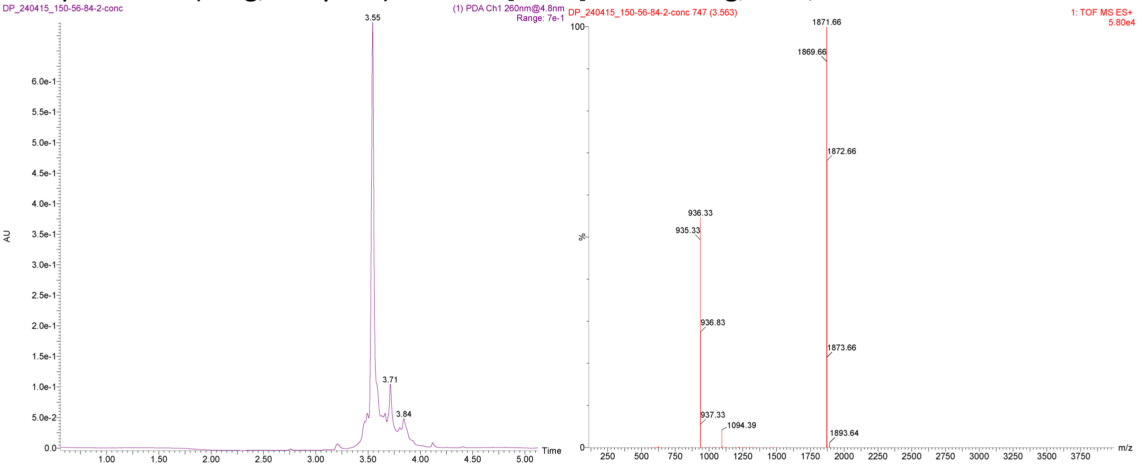

Figure S60: LC-MS chromatogram (left) and the corresponding deconvoluted MS spectra (right) of compound 18

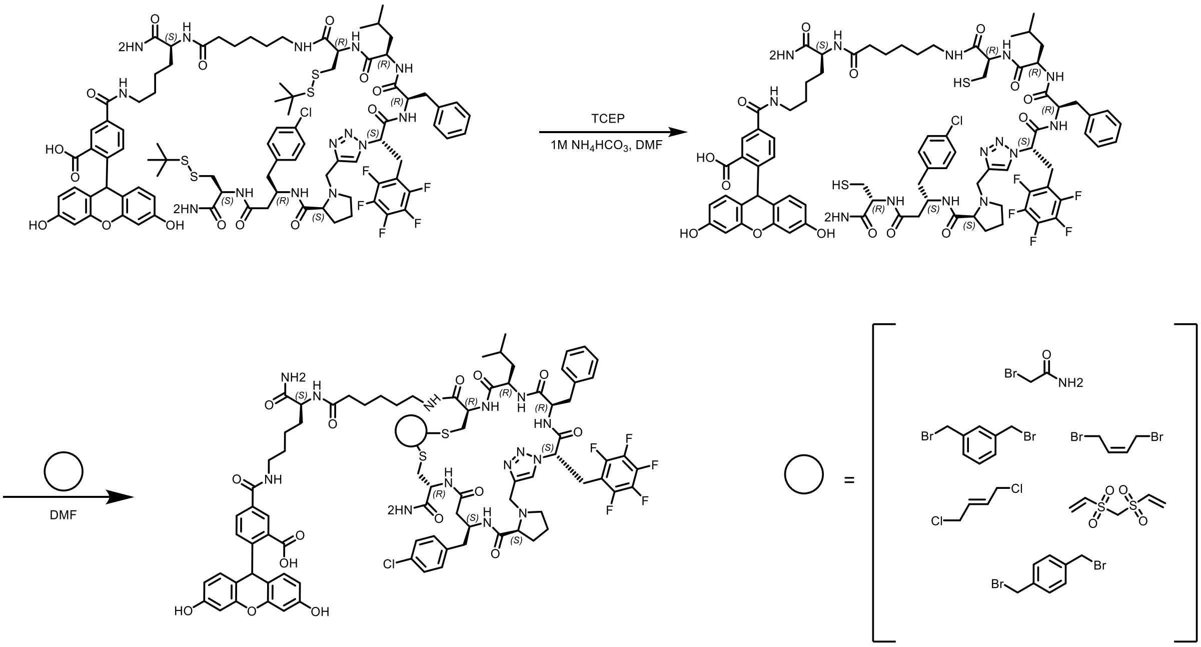

19-24

Synthesis of 19-24: Compound **18** (50 µL, 6.7 mM in DMSO) was treated with TCEP so that the two cysteine Stbu groups were deprotected and the cysteines were reacted with 1 of 6 different electrophiles according to general procedure, to generate 5 different macrocycles **19-23** and one open peptide **24**, which were all HPLC purified.

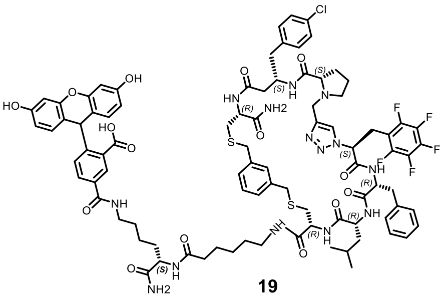

19

Compound **19** (0.0317 mg, 5% yield) ESI-MS: [M+H]+ 1796.64 g/mol , found 1796.64

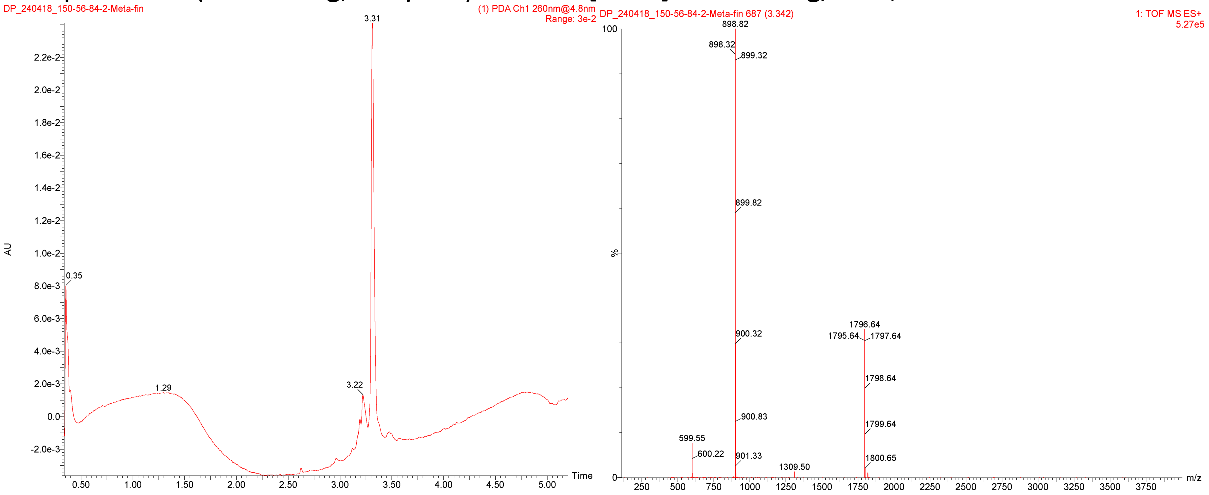

Figure S61: LC-MS chromatogram (left) and the corresponding deconvoluted MS spectra (right) of compound 19

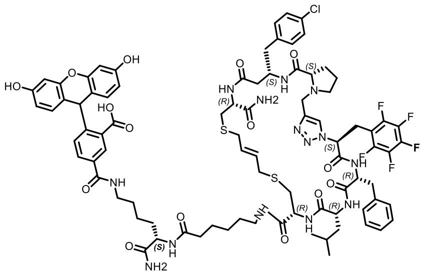

20

Compound **20** (0.0515 mg, 8% yield) ESI-MS: [M+H]+ 1746.62 g/mol , found 1746.63

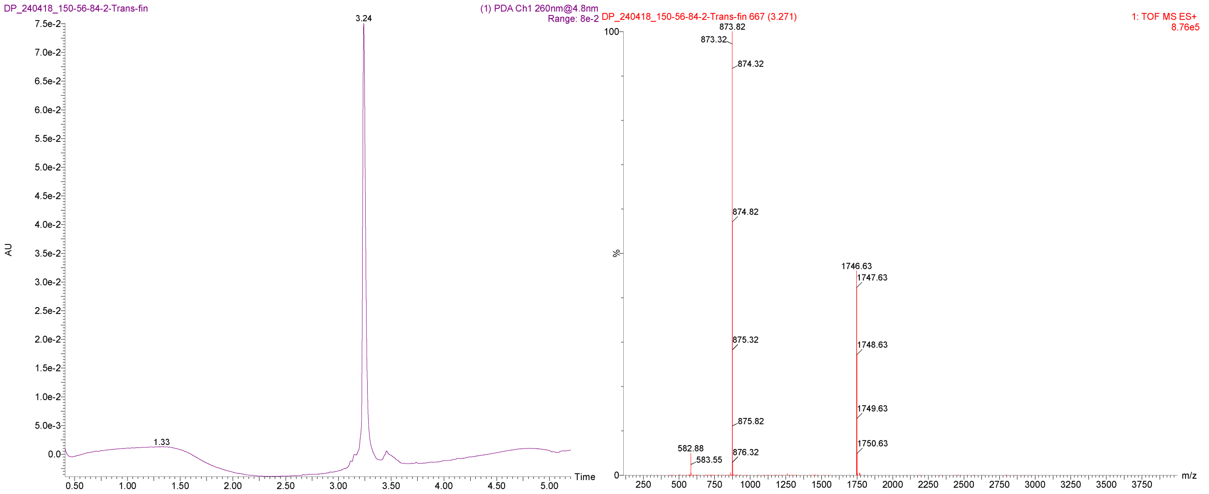

Figure S62: LC-MS chromatogram (left) and the corresponding deconvoluted MS spectra (right) of compound 20

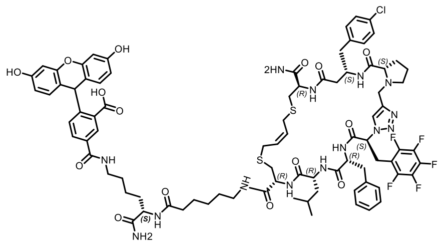

21

Compound **21** (0.0515 mg, 8% yield) ESI-MS: [M+H]+ 1746.62 g/mol , found 1746.63

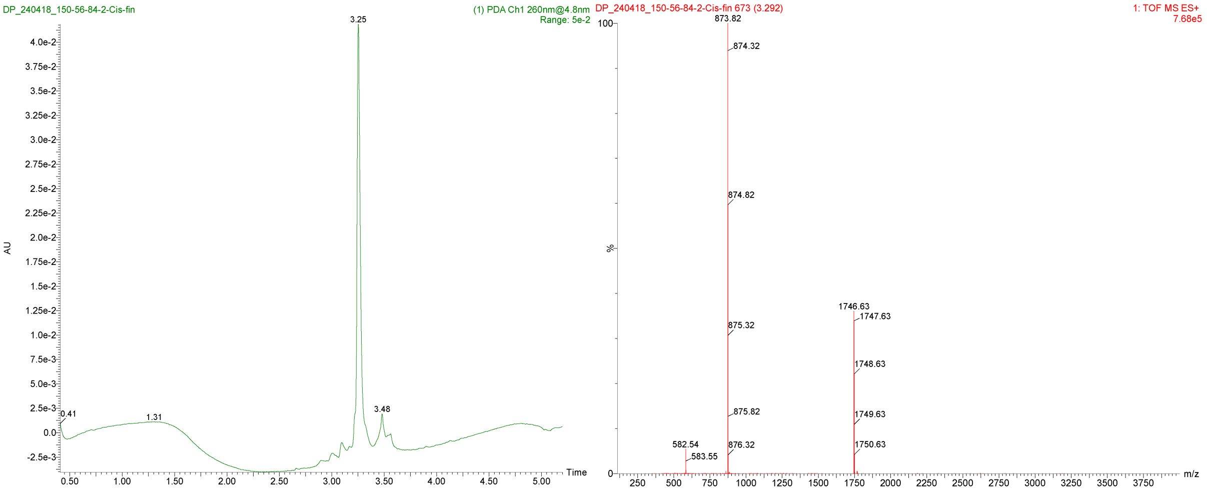

Figure S63: LC-MS chromatogram (left) and the corresponding deconvoluted MS spectra (right) of compound 21

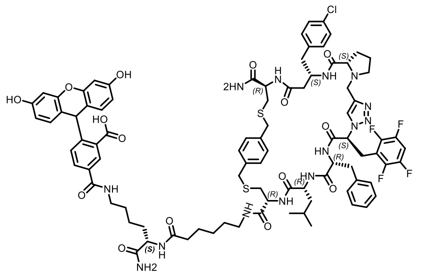

22

Compound **22** (0.0515 mg, 8% yield) ESI-MS: [M+H]+ 1796.64 g/mol , found 1796.64

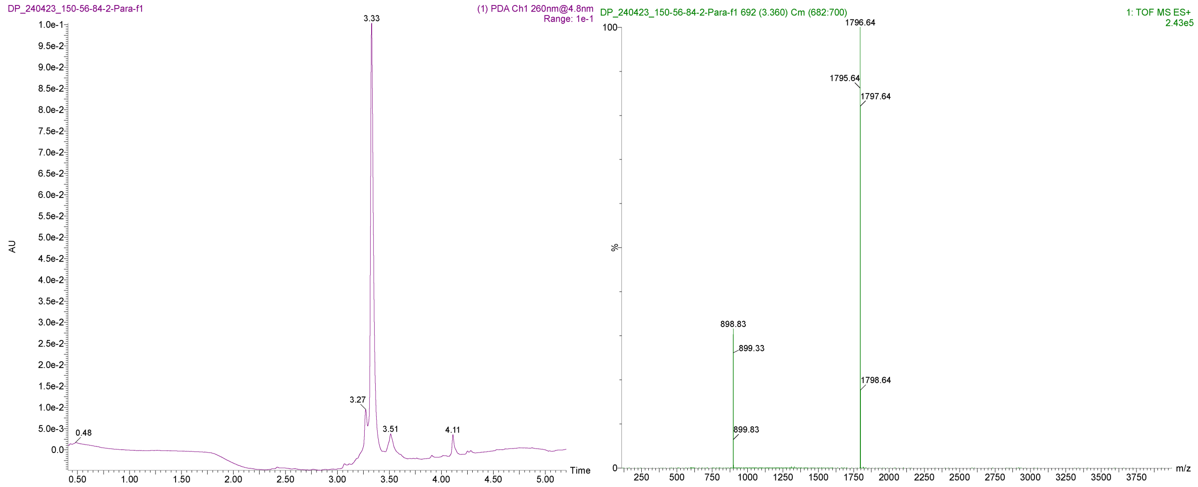

Figure S64: LC-MS chromatogram (left) and the corresponding deconvoluted MS spectra (right) of compound 22

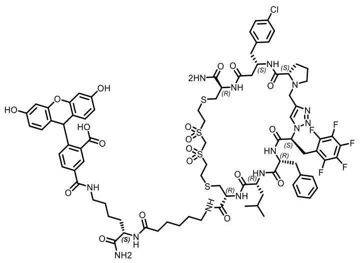

23

Compound **23** (0.0515 mg, 8% yield) ESI-MS: [M+H]+ 1890.58 g/mol , found 1891.59

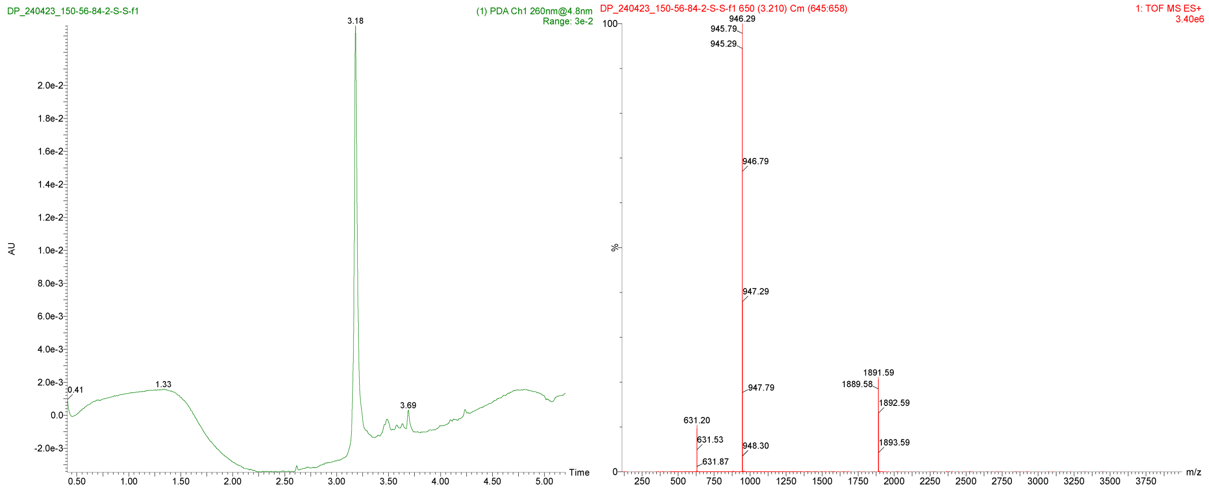

Figure S65: LC-MS chromatogram (left) and the corresponding deconvoluted MS spectra (right) of compound 23

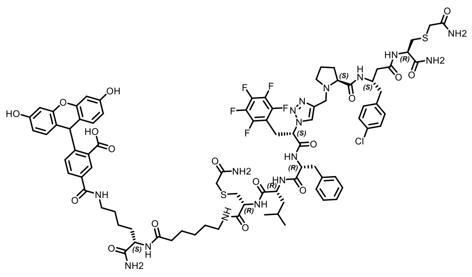

24

Compound **24** (0.0515 mg, 8% yield) ESI-MS: [M+H]+ 1808.63 g/mol , found 1808.47

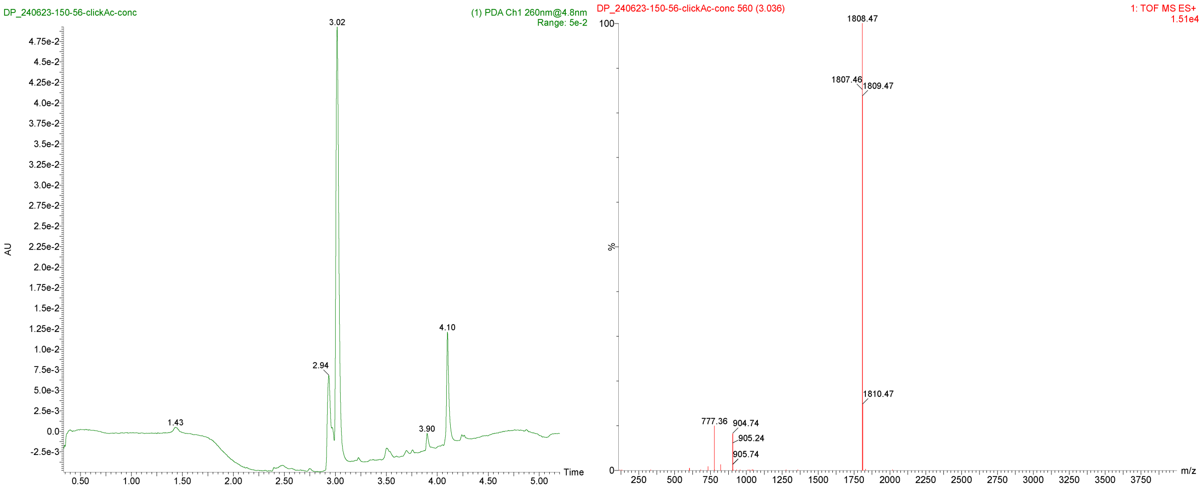

Figure S66: LC-MS chromatogram (left) and the corresponding deconvoluted MS spectra (right) of compound 24

6.2.4. PLAP (Compounds 29 & 30)

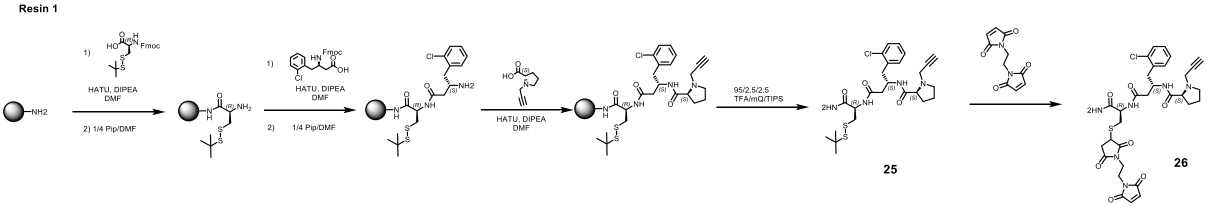

Synthesis of 26: 500 mg of resin **R1** was reacted in repeating amidation and Fmoc deprotection cycles with Fmoc-Cys(StBu)-OH, Fmoc-2-chloro-L-β-homophenylalanine and prop-2-yn-1-yl-L-proline to generate intermediate **25.** The intermediate was cleaved off the resin, precipitated and redissolved in DMSO. 50 µL of intermediate was added to 350 µL DMF, 150 µL 0.5M KH_2PO_4 pH8 and 50µL of 160 mM TCEP in 0.5M KH_2PO_4 pH8. The deprotection was carried out over 1h and checked via LC-MS for completion. Upon completion 100 µL of 160 mM azidobenzoic acid in DMF was added. Reaction was then incubated further for 120 min. 50 µL of 160 mM 1,1'-(ethane-1,2-diyl)bis(1H-pyrrole-2,5-dione) was then added. Reaction was monitored and directly purified via RP-HPLC upon completion to yield **26.**

Compound **25** ()ESI-MS: [M+H]+ 539.15 g/mol , found 539.16

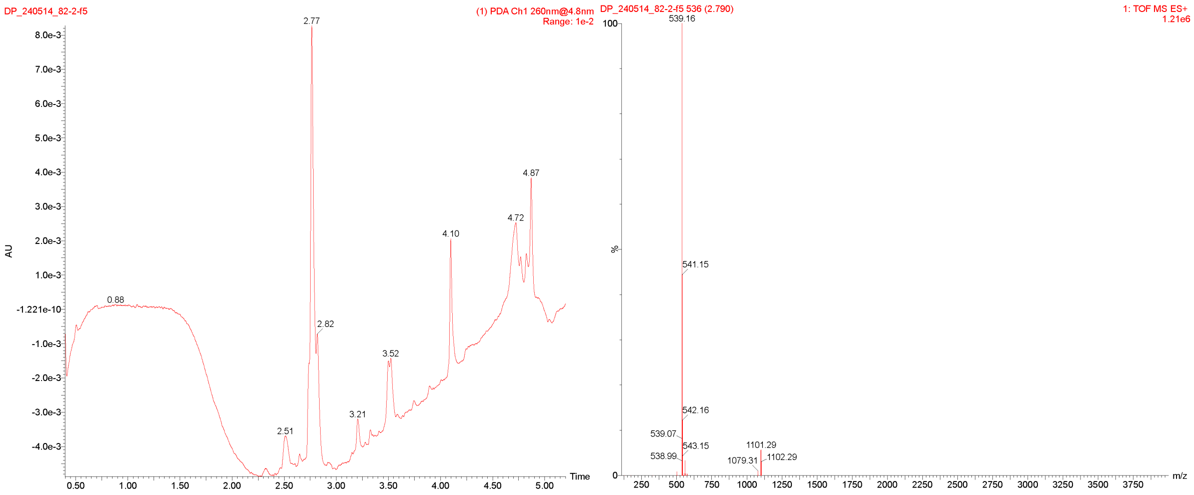

Figure S67: LC-MS chromatogram (left) and the corresponding deconvoluted MS spectra (right) of compound 25

Compound **26** (10 mg)ESI-MS: [M+H]+ 670.2 g/mol , found 671.18

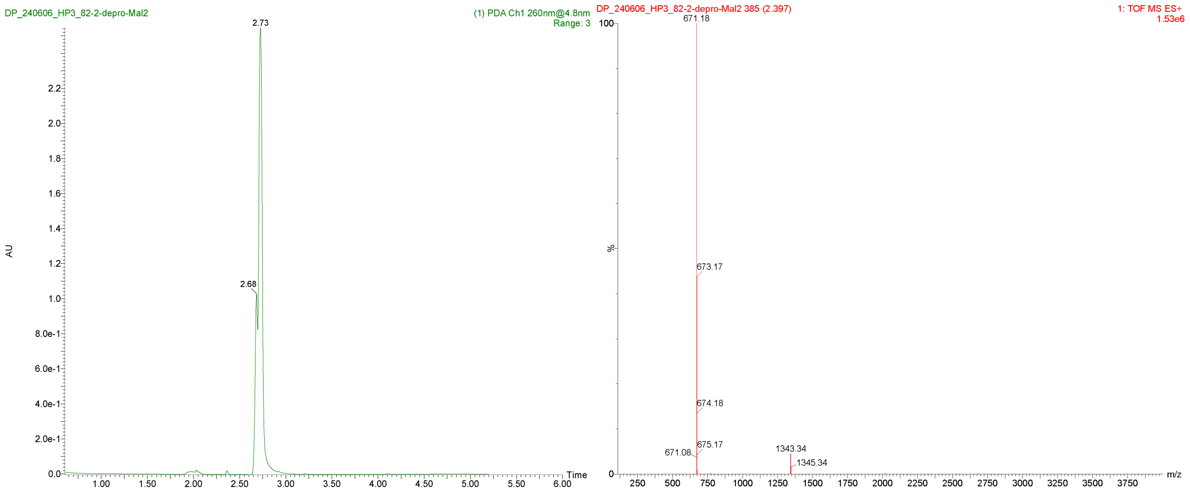

Figure S68: LC-MS chromatogram (left) and the corresponding deconvoluted MS spectra (right) of compound 26

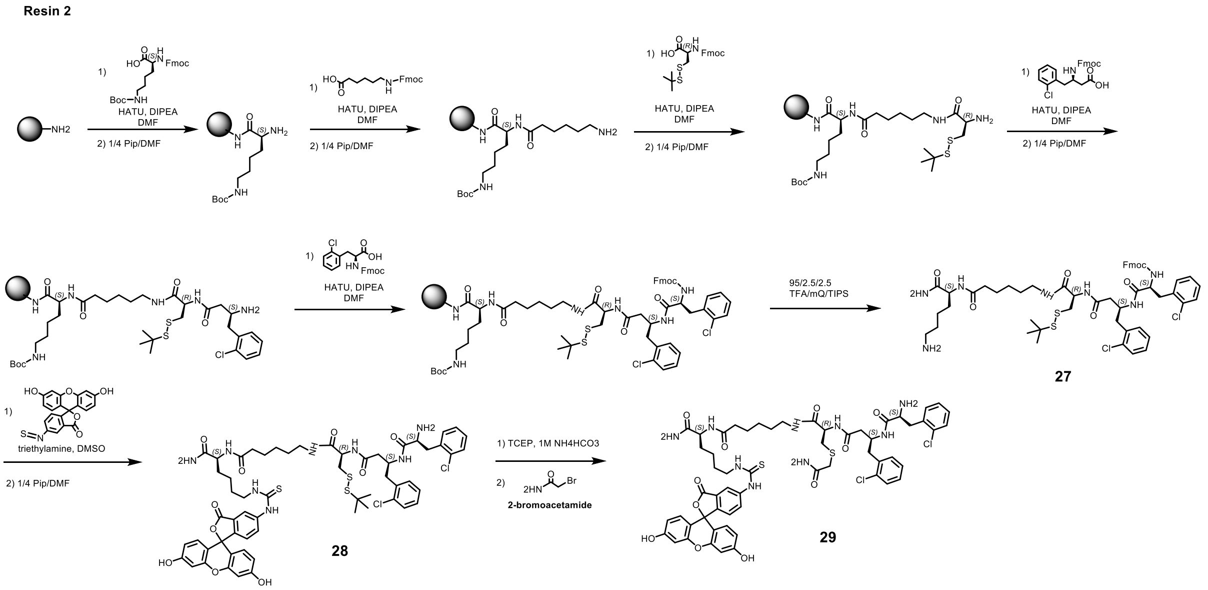

Synthesis of 29: 500 mg of resin **R1** was reacted in repeating amidation and Fmoc deprotection cycles with Fmoc-Lys(Boc)-OH, Fmoc-6-Ahx-OH, Fmoc-Cys(StBu)-OH, Fmoc-2-chloro-L-β-homophenylalanine and Fmoc-Phe(2-Cl)-OH. The peptide was then cleaved from the resin and precipitated to result in intermediate **27.**

Compound **27** ESI-MS: [M+H]+ 1049.18 g/mol , found 1048.33

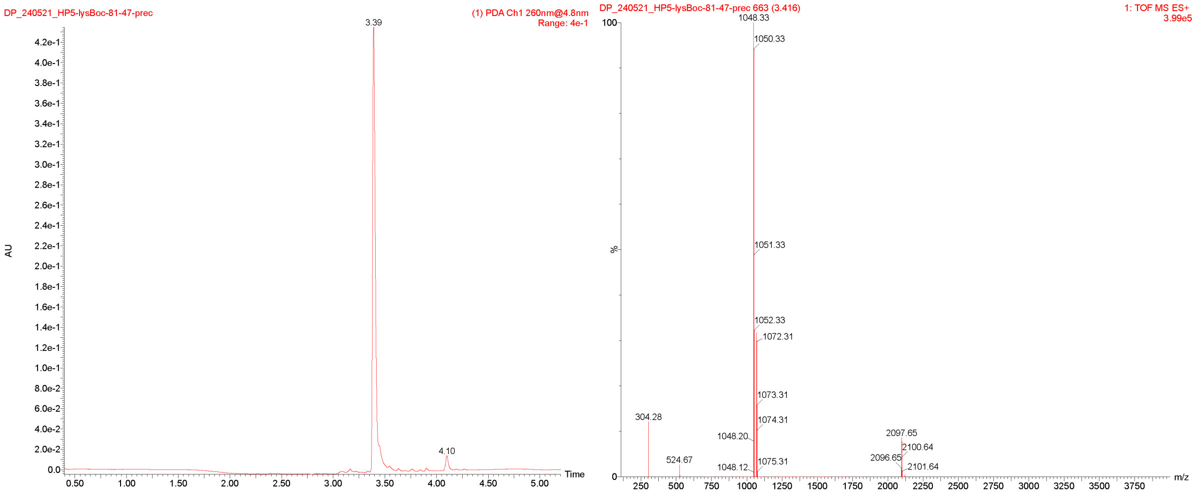

Figure S69: LC-MS chromatogram (left) and the corresponding deconvoluted MS spectra (right) of compound 27

The intermediate was then dissolved in DMSO, to which 1.1 equivalents of Fluorescein-5-Isothiocyanate and 10 equivalents of triethylamine were added. The reaction was monitored via LC-MS and upon completion, piperidine was added and reacted for 15-30 min, before diluted with sodium acetate, methanol and purified via HPLC to yield **28.**

Compound **28** (5.8 mg, 3% yield) ESI-MS: [M+H]+ 1216.32 g/mol , found 1215.28

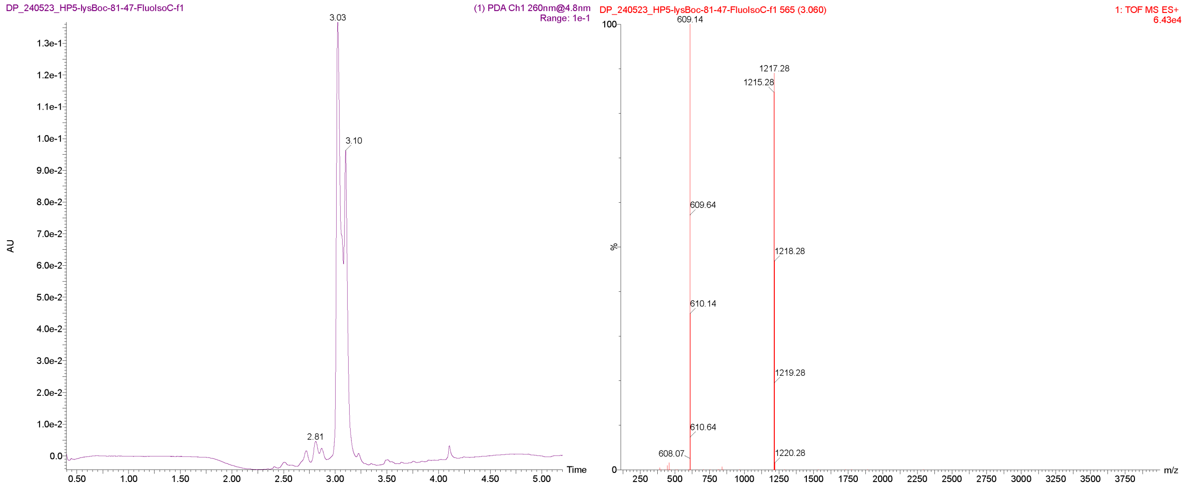

Figure S70: LC-MS chromatogram (left) and the corresponding deconvoluted MS spectra (right) of compound 28

1.9 mg compound **28** was dissolved in DMF, treated with TCEP and treated with 2-bromoacetamide according to listed procedures. The compound was then HPLC purified to yield **29.**

Compound **29** (0.24 mg, 12% yield) ESI-MS: [M+H]+ 1183.35 g/mol , found 1184.29

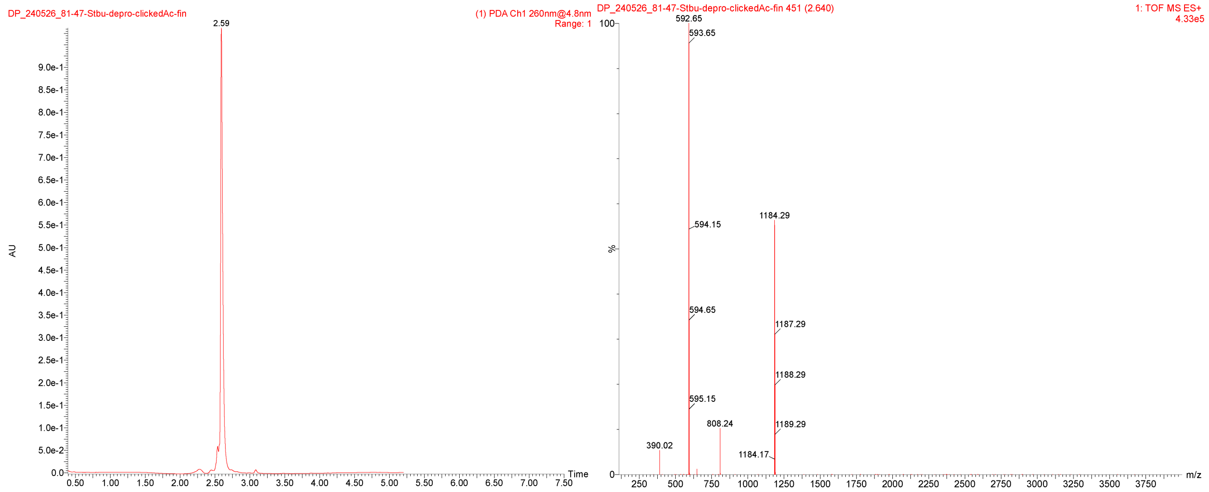

Figure S71: LC-MS chromatogram (left) and the corresponding deconvoluted MS spectra (right) of compound 29

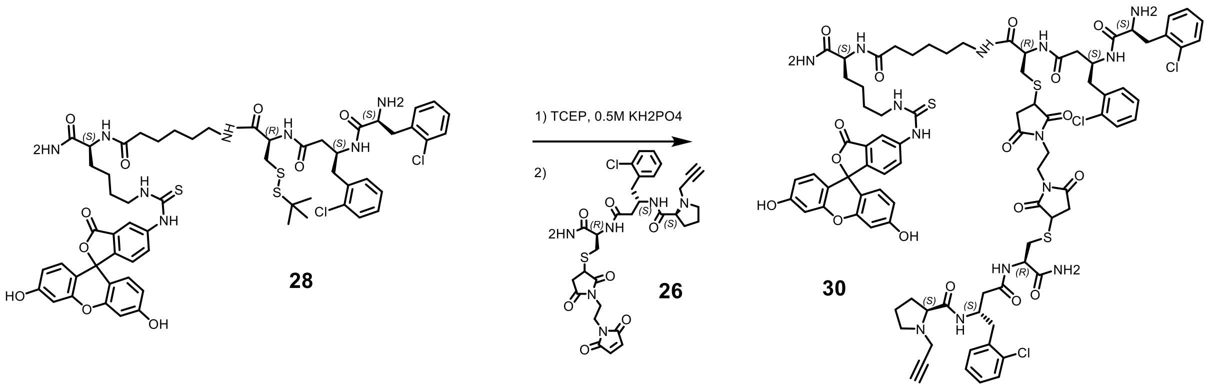

Synthesis of 30: 50 µL 2 mM of **28** was added to 350 µL DMF, 150 µL 0.5M KH_2PO_4 pH8 and 50µL of 160 mM TCEP in 0.5M KH_2PO_4 pH8. The deprotection was carried out over 1h and checked via LC-MS for completion. Upon completion 100 µL of 160 mM azidobenzoic acid in DMF was added. Reaction was then incubated further for 120 min. 50 µL of **26** (1.5 equiv.) in DMSO was then added. Reaction was monitored and directly purified via RP-HPLC upon completion to yield **30.**

Compound **30** (0.027 mg, 15% yield) ESI-MS: [M+H]+ 1796.52 g/mol , found 1799.48

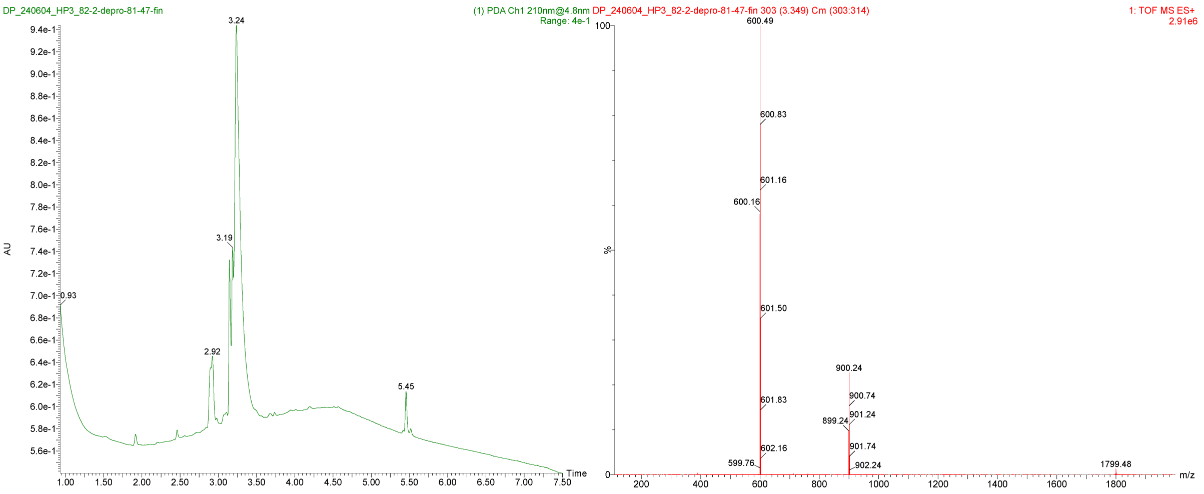

Figure S72: LC-MS chromatogram (left) and the corresponding deconvoluted MS spectra (right) of compound 30

6.2.5. Streptavidin (Compounds 34-40)

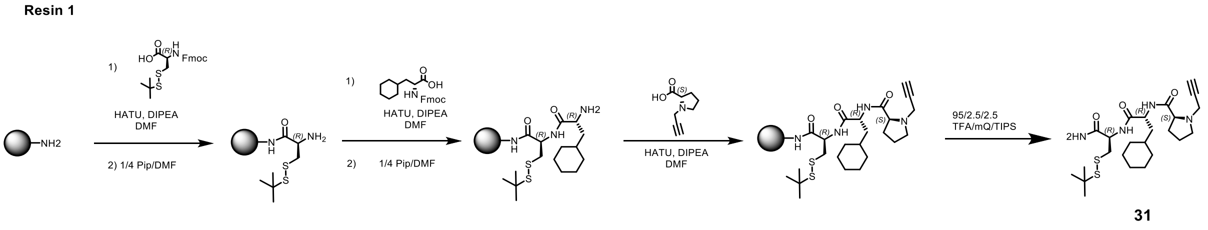

Synthesis of 31: 500 mg of resin **R1** was reacted in repeating amidation and Fmoc deprotection cycles with Fmoc-Cys(StBu)-OH, (R)-2-((((9H-fluoren-9-yl)methoxy)carbonyl)amino)-3-cyclohexylpropanoic acid and prop-2-yn-1-yl-L-proline to

generate intermediate **31.** The intermediate was cleaved off the resin, precipitated and used as is for resin 2 synthesis of **32.**

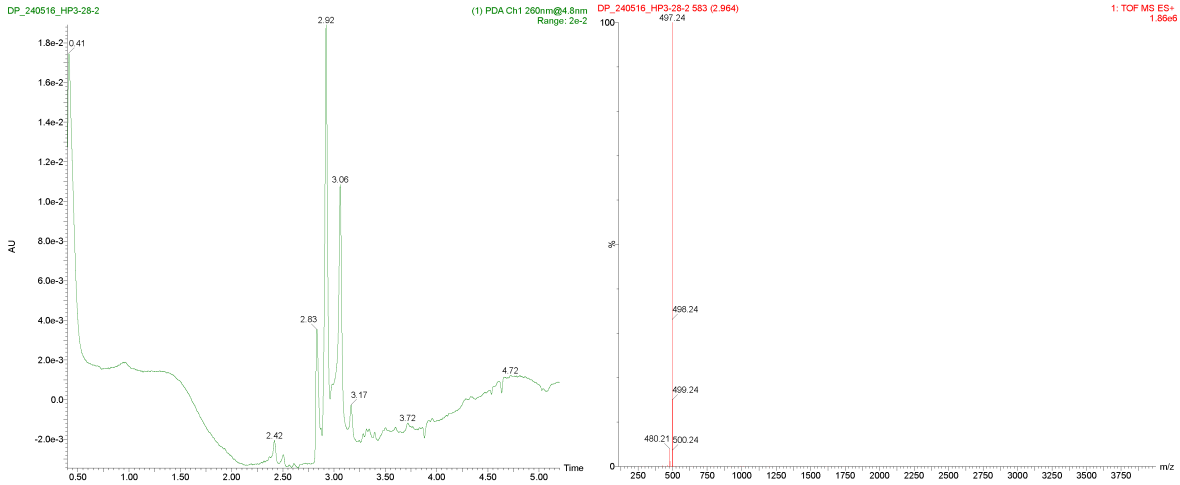

Figure S73: LC-MS chromatogram (left) and the corresponding deconvoluted MS spectra (right) of compound 31

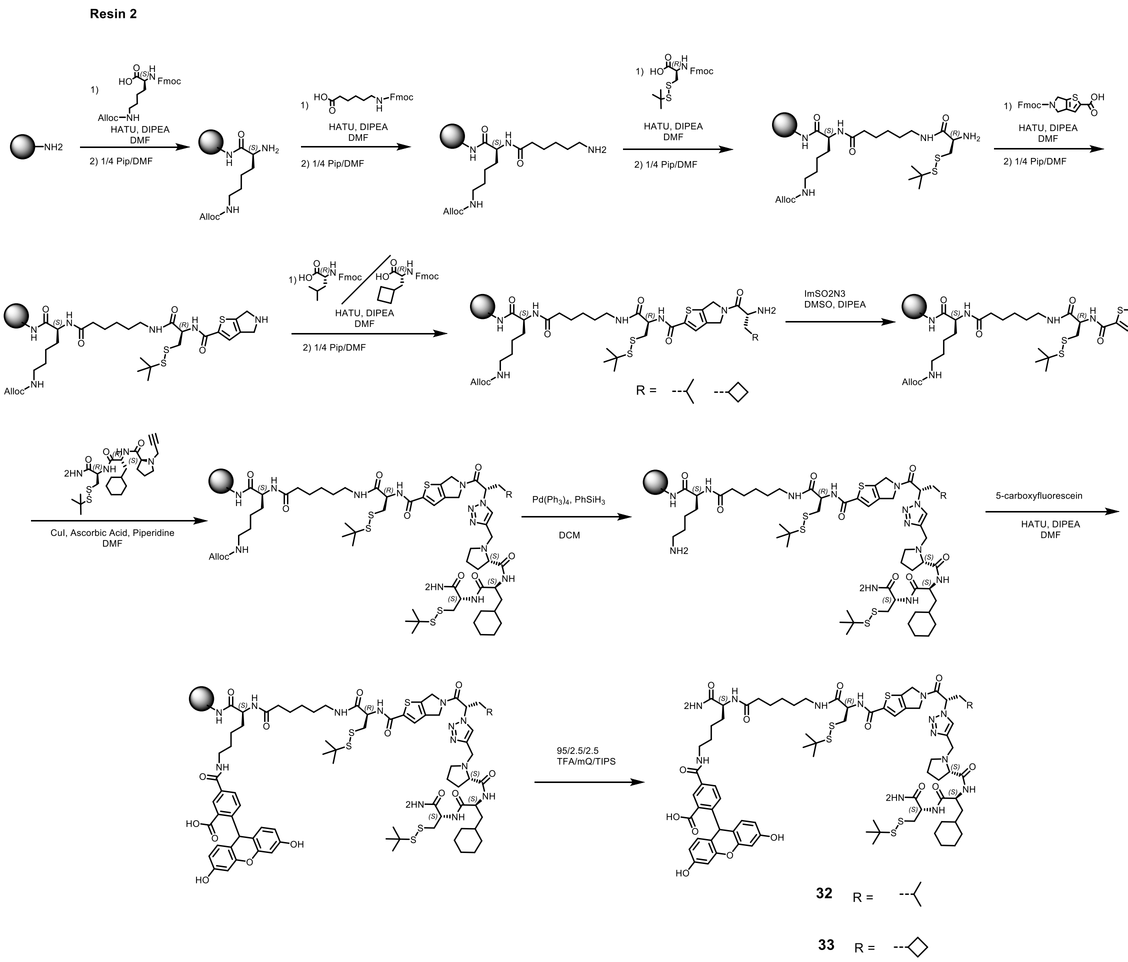

Synthesis of 32 & 33: 500 mg of resin **R1** was reacted in repeating amidation and Fmoc deprotection cycles with Fmoc-Lys(Alloc)-OH, Fmoc-6-Ahx-OH, Fmoc-Cys(StBu)-OH, 5-(((9H-fluoren-9-yl)methoxy)carbonyl)-5,6-dihydro-4H-thieno[2,3-c]pyrrole-2-carboxylic acid and Fmoc-D-Leucine (cmpd. **32**) or Fmoc-β-cyclobutyl-D-Ala-OH (cmpd. **33**). The terminal amino group was then subjected to a diazotransfer reaction and reacted via CuAAC with

intermediate **31**. The lysine Alloc group was then deprotected and 5-carboxyfluorescein was attached to afford compounds **32** and **33,** which were cleaved off the resin and HPLC purified.

Compound **32** (3.2 mg, 1% yield) ESI-MS: [M+H]+ 1595.65 g/mol , found 1594.65

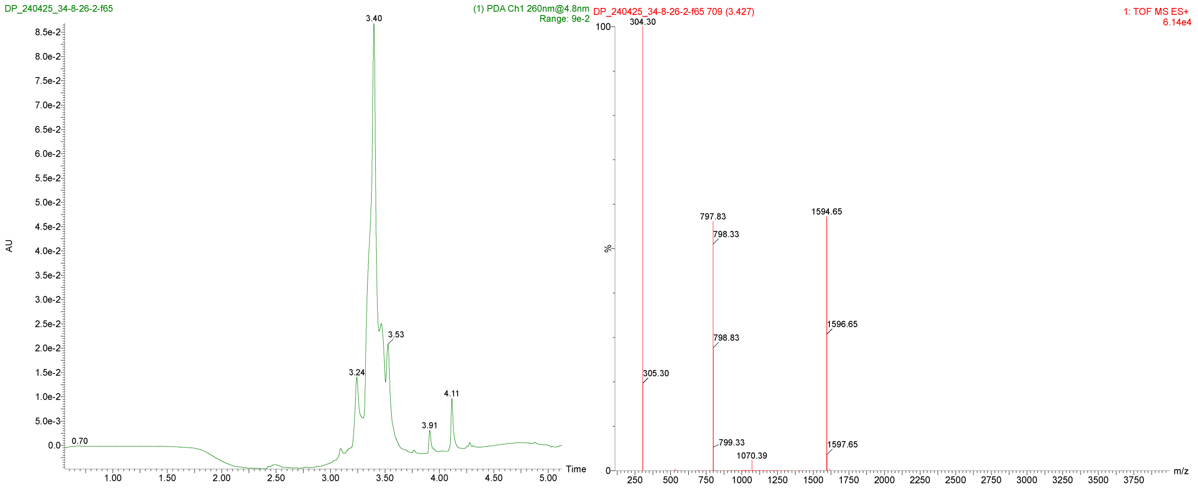

Figure S74: LC-MS chromatogram (left) and the corresponding deconvoluted MS spectra (right) of compound 32

Compound **33** (2.5 mg, 1% yield) ESI-MS: [M+H]+ 1607.65 g/mol , found

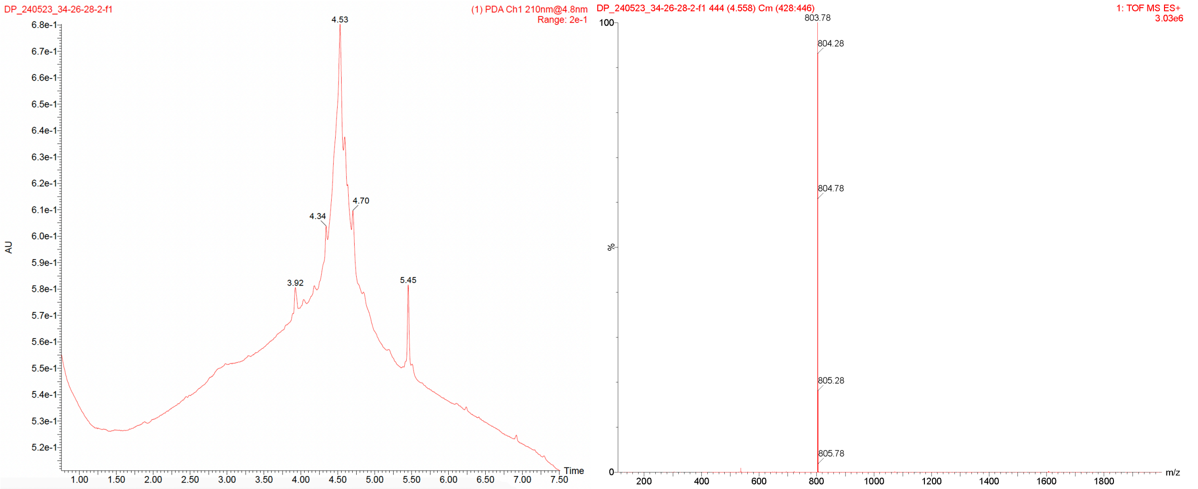

Figure S75: LC-MS chromatogram (left) and the corresponding deconvoluted MS spectra (right) of compound 33

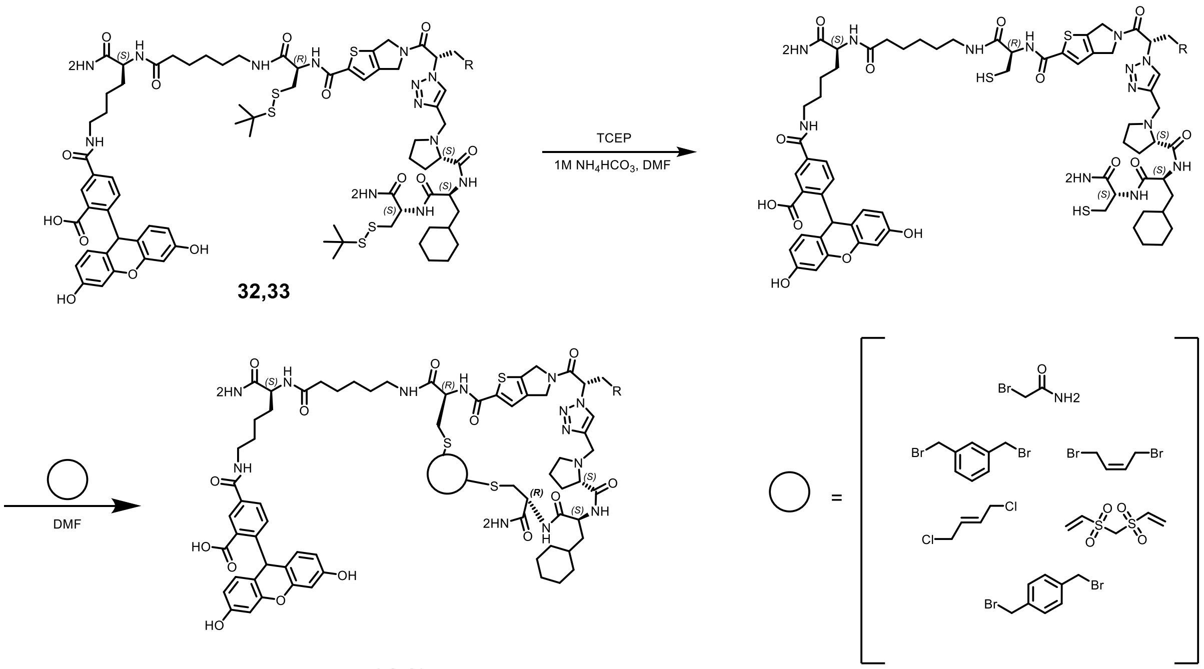

34-40

Synthesis of 34-39: Compound **32** (50 µL, 2.8 mM in DMSO) was treated with TCEP so that the two cysteine Stbu groups were deprotected and the cysteines were reacted with 1 of 6 different electrophiles according to general procedure, to generate 5 different macrocycles **34-38** and one open peptide **39**, which were all HPLC purified.

Synthesis of 40: Compound **33** (50 µL, 1.6 mM in DMSO) was treated with TCEP so that the two cysteine Stbu groups were deprotected and the cysteines were reacted with 1 electrophile according to general procedure, to one open peptide **40**, which was HPLC purified.

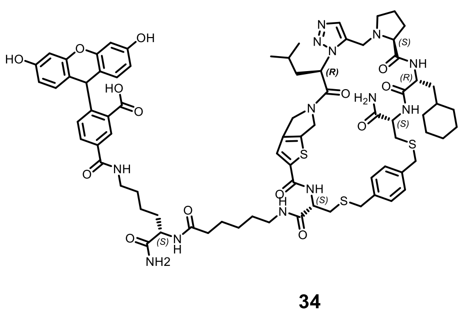

34

Compound **34** (0.031 mg, 16% yield) ESI-MS: [M+H]+ 1521.63 g/mol , found 1520.48

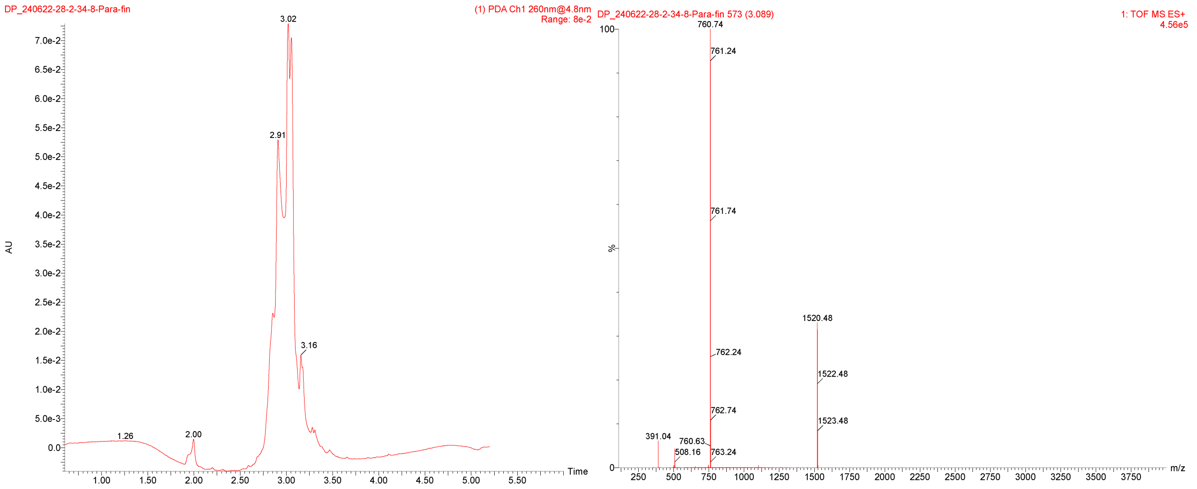

Figure S76: LC-MS chromatogram (left) and the corresponding deconvoluted MS spectra (right) of compound 34

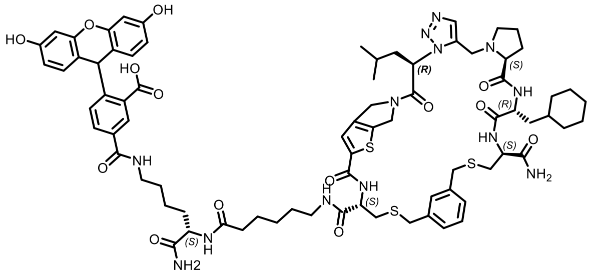

35

Compound **35** (0.0268 mg, 13% yield) ESI-MS: [M+H]+ 1521.63 g/mol , found 1520.46

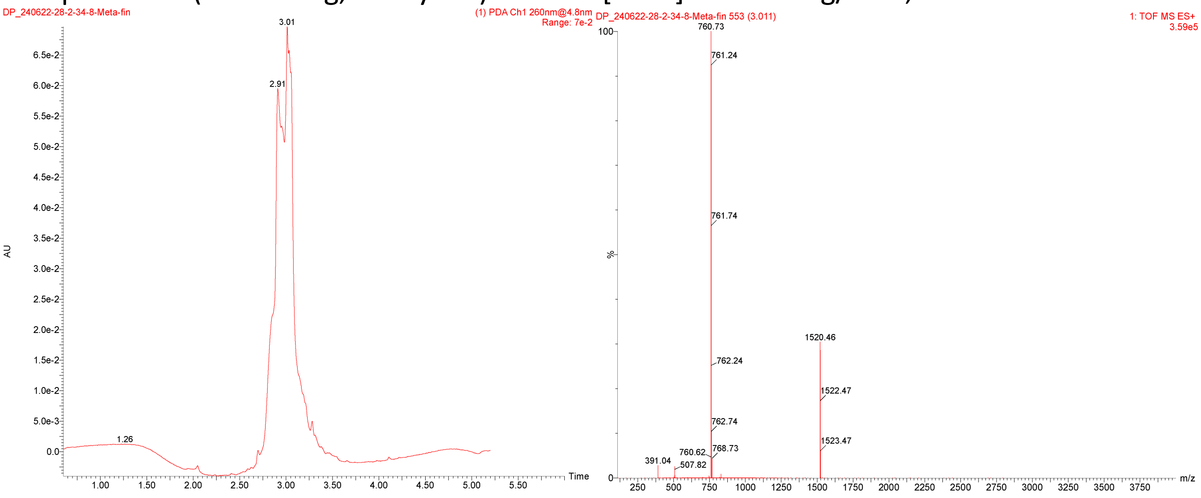

Figure S77: LC-MS chromatogram (left) and the corresponding deconvoluted MS spectra (right) of compound 35

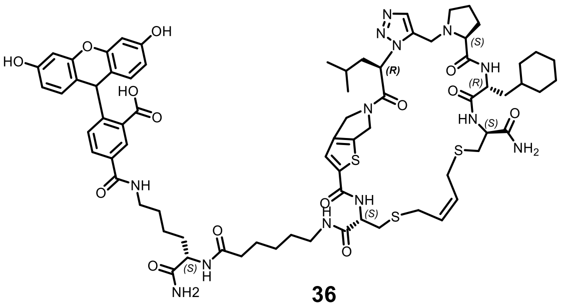

36

Compound **36** (0.0253 mg, 13% yield) ESI-MS: [M+H]+ 1471.61 g/mol , found 1470.50

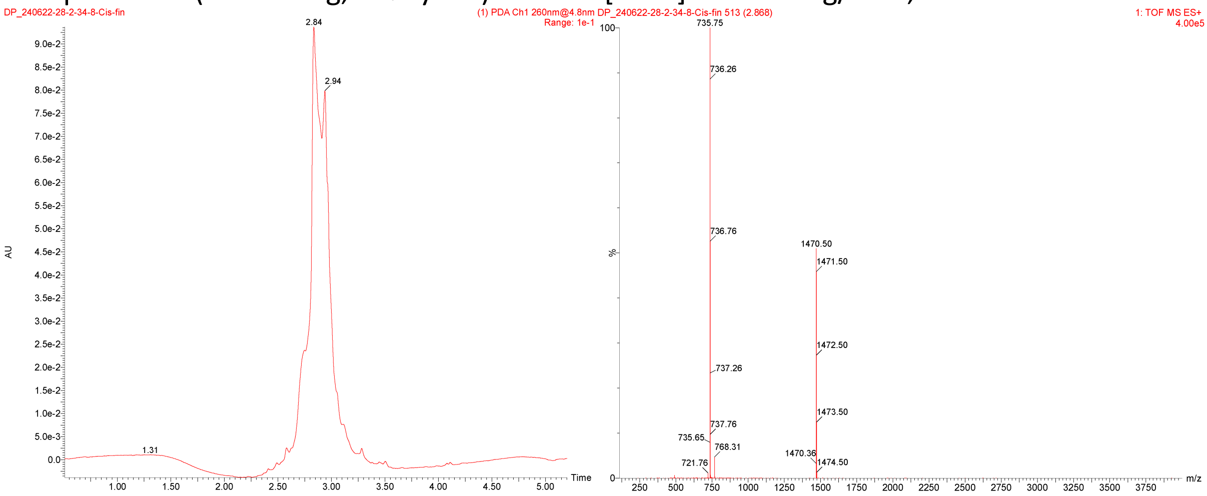

Figure S78: LC-MS chromatogram (left) and the corresponding deconvoluted MS spectra (right) of compound 36

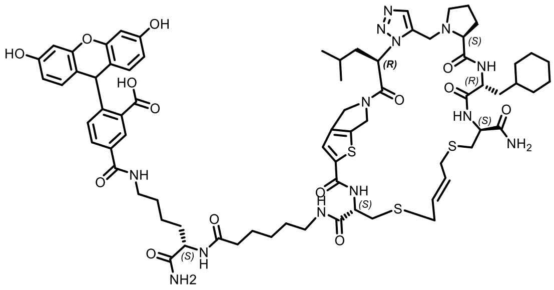

37

Compound **37** (0.028 mg, 15% yield) ESI-MS: [M+H]+ 1471.61 g/mol , found 1470.48

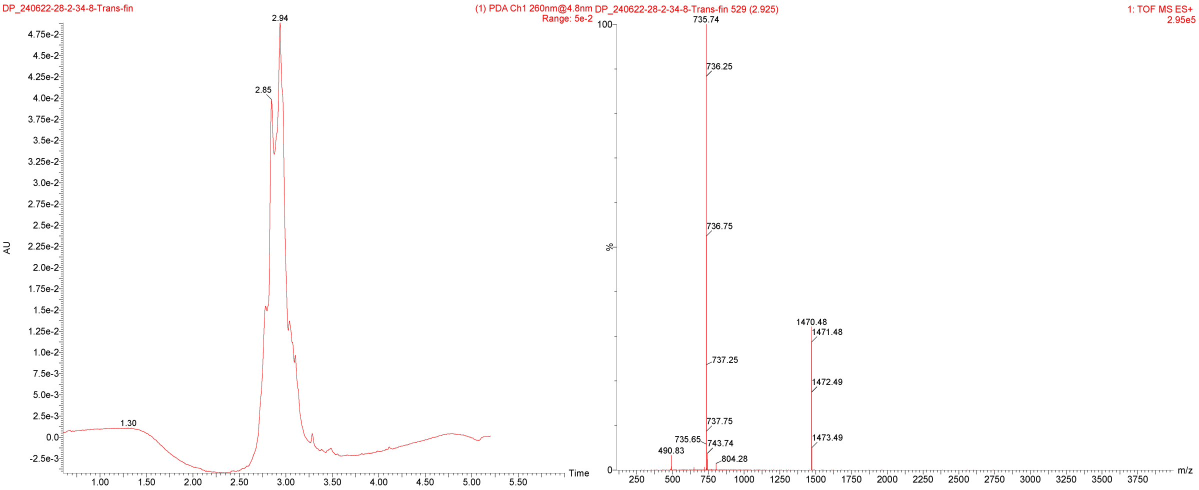

Figure S79: LC-MS chromatogram (left) and the corresponding deconvoluted MS spectra (right) of compound 37

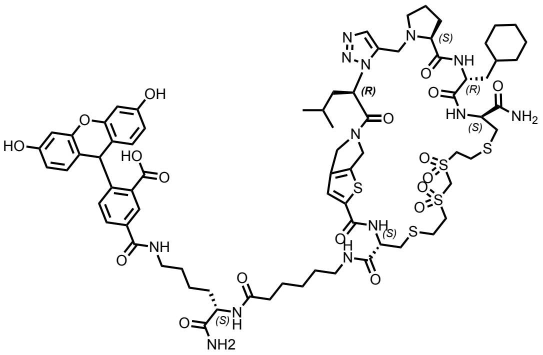

38

Compound **38** (0.0421 mg, 20% yield) ESI-MS: [M+H]+ 1616.96 g/mol , found 1614.44

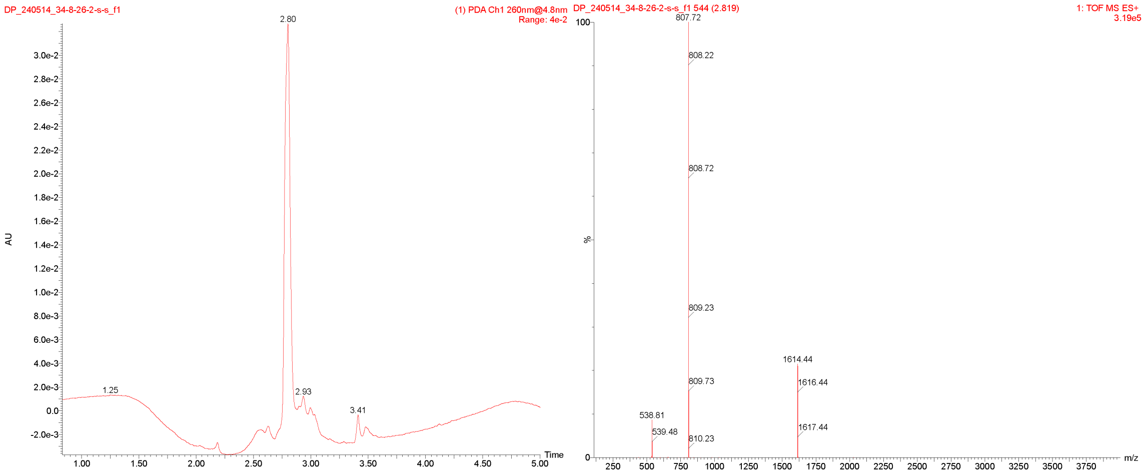

Figure S80: LC-MS chromatogram (left) and the corresponding deconvoluted MS spectra (right) of compound 38

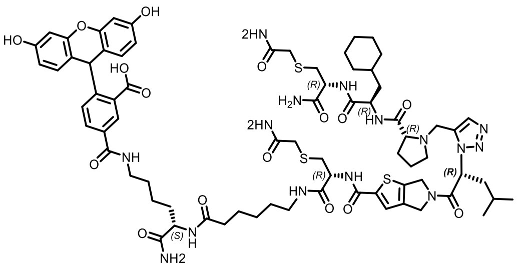

39

Compound **39** (0.0475 mg, 25% yield) ESI-MS: [M+H]+ 1533.62 g/mol , found 1532.52

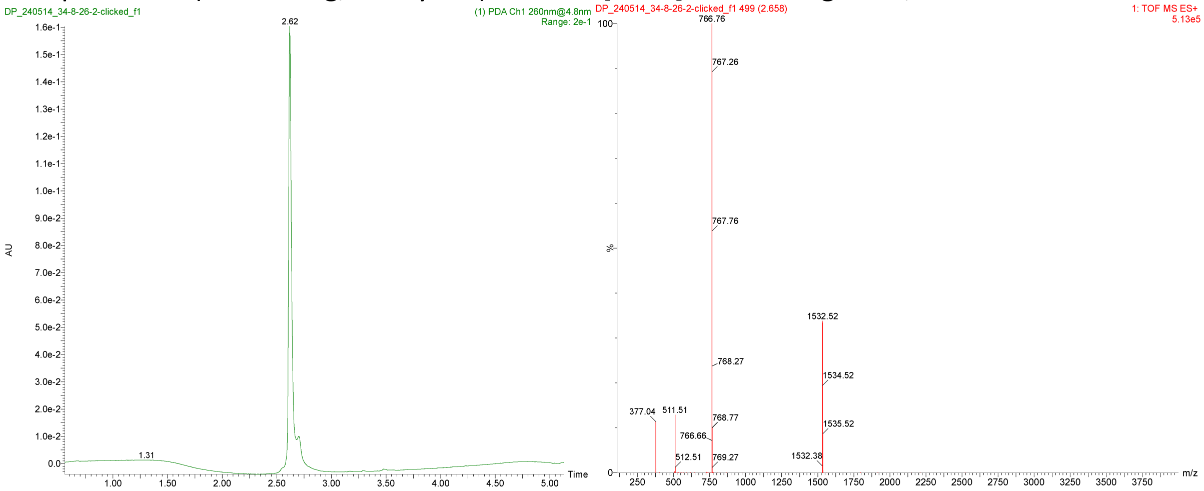

Figure S81: LC-MS chromatogram (left) and the corresponding deconvoluted MS spectra (right) of compound 39

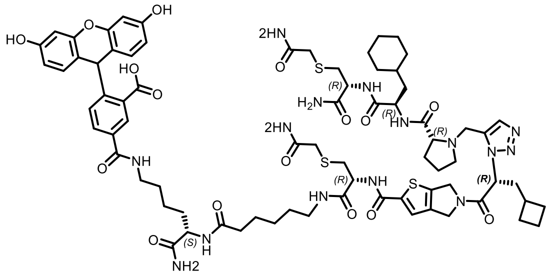

40

Compound **40** (0.0245 mg, 19% yield) ESI-MS: [M+H]+ 1545.62 g/mol , found 1545.50

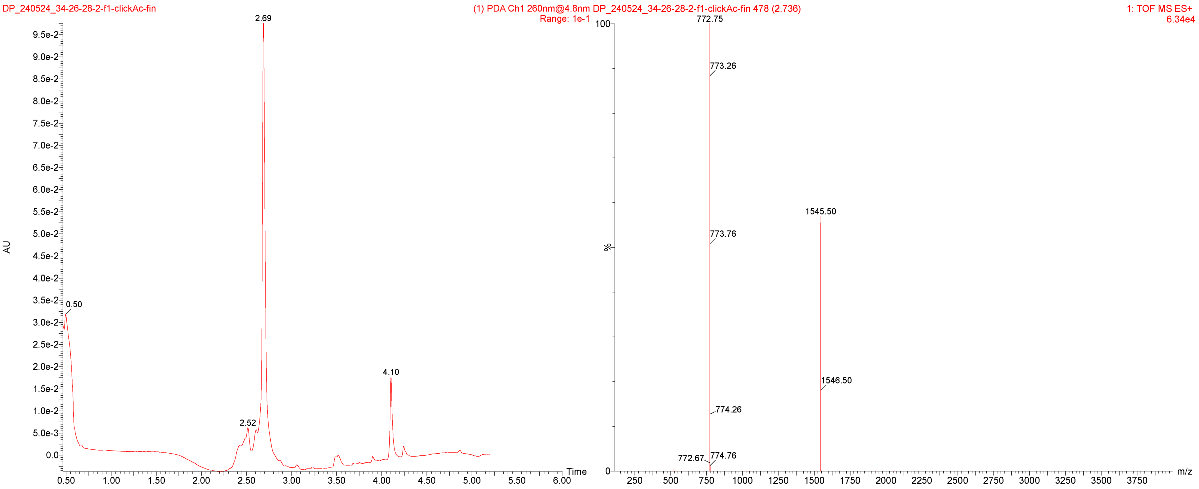

Figure S82: LC-MS chromatogram (left) and the corresponding deconvoluted MS spectra (right) of compound 40

6.2.6. Negative Control

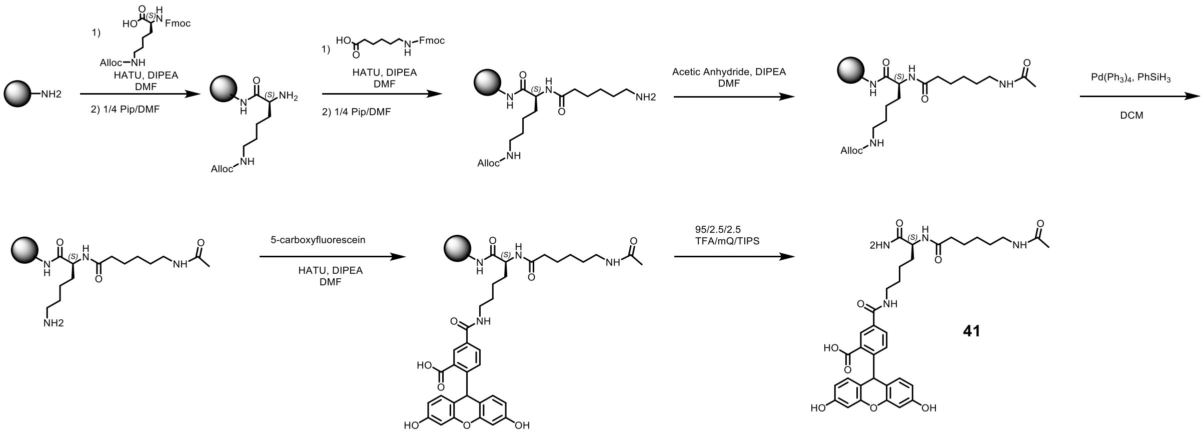

Compound **41** was assembled using H-Rink amide ChemMatrix® resin according to procedures in Onda et al. [11]

Compound **41** (21 mg, 31.9% yield) ESI-MS: [M+H]+ 660.28 g/mol , found 659.27

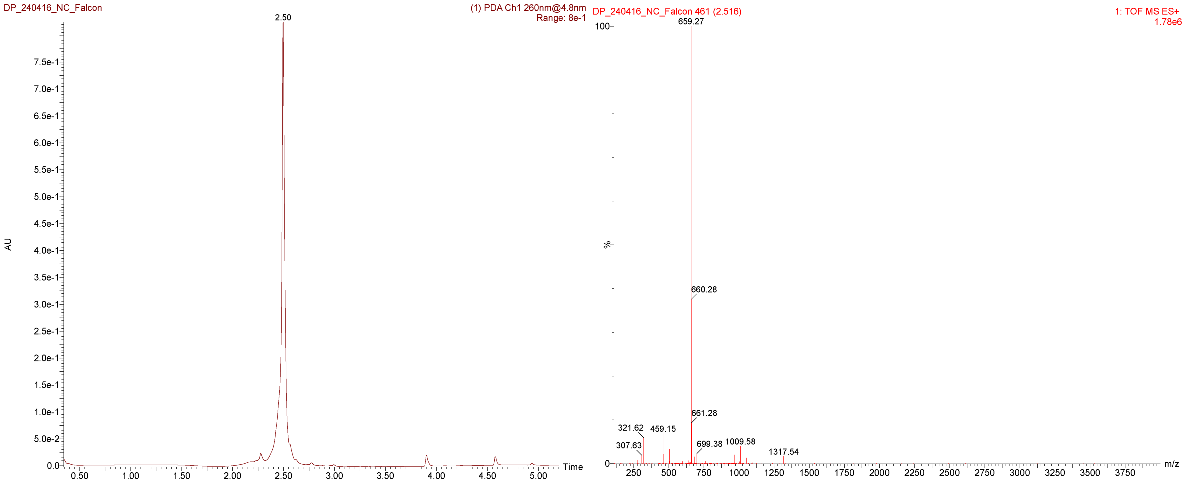

Figure S83: LC-MS chromatogram (left) and the corresponding deconvoluted MS spectra (right) of compound 41

7. Hit Validation

7.1. Fluorescence Polarization

Protocol

LNA5 and LNA3 conjugates were hybridized by mixing 25 µL of LNA5 (2 µM) and 25 µL of LNA3 (2 µM) conjugates in mQ and heating to 70 °C for 5-10 min. After cooling down the solution to room temperature, 950 µL of 1x PBS was added to the LNA solution. The hybridized LNA conjugates were then tested via fluorescence polarization experiment.

If the LNA conjugates were clicked together, they were diluted to 1 µM, 50 µL of a clicked conjugate could then be taken, diluted with 950 µL 1x PBS and tested via fluorescence polarization experiment.

All other off-DNA fluoresceinated compounds were diluted to 50 nM in 1xPBS containing 5% DMSO.

Separately, a 16-point serial dilution of protein was performed in PBS in PCR tubes. 4 µL of the 50 nM conjugate was mixed with 4 µL of each protein dilution in black 384-well plates to a final volume of 8 µL. The plate was incubated for 30 min in the dark and centrifuged briefly to remove bubbles. Fluorescence anisotropy was measured at 535 nm. Data was obtained and fitted using Prism 10.

Raw Data

Table S17: Raw data from main text figure 3

Molar	**1** Open			**1** Closed			Control		
0.00002	311	322		315	317	318	197	199	201
0.00001	298	313		314	314	299	185	194	189
0.000005	283	298		302	302	292	187	186	184
2.5E-06	254	281		294	293	289	179	180	176
1.25E-06	239	256		274	290	267	173	177	173
6.25E-07	230	236		256	274	257	173	175	168
3.13E-07	219	228		242	248	242	172	171	171
1.56E-07	219	221		235	241	235	168	173	175
7.81E-08	214	215		225	231	223	171	171	170
3.91E-08	213	216		224	241	218	170	156	168
1.95E-08	215	223		218	236	221	169	171	170
9.77E-09	213	225		215	223	219	170	168	168
4.88E-09	213	223		210	222	216	171	173	169
2.44E-09	214	225		211	221	210	167	169	165
1.22E-09	213	216		210	224	218	170	172	173
6.1E-10	213	215		212	208	209	164	164	164
Molar	**2** Open			**2** Closed			Control		
0.00002	290	292	296	300	292	300	197	199	201
0.00001	280	282	288	298	300	292	185	194	189
0.000005	273	274	276	283	273	275	187	186	184
2.5E-06	254	256	260	268	271	267	179	180	176
1.25E-06	239	240	242	254	252	253	173	177	173
6.25E-07	222	222	224	233	237	246	173	175	168
3.13E-07	216	217	218	230	229	227	172	171	171
1.56E-07	209	213	209	222	221	215	168	173	175
7.81E-08	209	210	208	215	209	212	171	171	170
3.91E-08	208	213	210	221	224	206	170	156	168

1.95E-08	206	210	209	211	217	207	169	171	170
9.77E-09	206	209	209	209	213	202	170	168	168
4.88E-09	207	208	209	206	215	207	171	173	169
2.44E-09	207	210	211	206	215	204	167	169	165
1.22E-09	207	209	211	202	200	207	170	172	173
6.1E-10	206	208	211	200	205	200	164	164	164

Molar	**3** Open			**3** Closed			Control		
0.00002	357	358	357	316	313	314	197	199	201
0.00001	358	355	348	312	308	314	185	194	189
0.000005	339	341	334	305	307	308	187	186	184
2.5E-06	321	326	317	299	299	299	179	180	176
1.25E-06	301	306	300	282	285	279	173	177	173
6.25E-07	290	287	279	269	277	271	173	175	168
3.13E-07	269	270	269	265	267	263	172	171	171
1.56E-07	261	264	266	261	260	260	168	173	175
7.81E-08	258	258	256	258	258	258	171	171	170
3.91E-08	256	256	254	262	258	254	170	156	168
1.95E-08	253	256	259	260	258	258	169	171	170
9.77E-09	258	258	258	254	257	264	170	168	168
4.88E-09	253	261	251	261	257	264	171	173	169
2.44E-09	261	259	257	255	263	259	167	169	165
1.22E-09	259	261	255	258	259	257	170	172	173
6.1E-10	258	255	257	257	250	252	164	164	164

Table S18: Raw data from main text figure 5 (non-normalized)

Molar	9			10			11			12			13			14			Control		
0.000024	386	382	352	384	379	383	293	290	292	260	261	264	393	391	393	335	334	331	171	171	168
0.000012	369	368	359	366	362	360	279	279	281	249	251	246	380	375	376	315	310	303	161	163	163
0.000006	350	350	335	333	334	335	266	267	262	236	230	235	365	371	365	253	274	275	158	157	159
0.000003	331	322	315	293	286	287	251	247	248	231	224	224	346	338	341	255	252	252	158	155	158
1.5E-06	303	305	297	273	273	270	235	231	237	227	226	224	326	319	318	225	225	222	161	157	160
7.5E-07	288	285	281	244	233	239	227	231	224	227	222	224	305	290	288	224	221	216	159	160	161
3.75E-07	272	270	271	255	249	245	227	223	220	224	217	223	285	280	269	213	212	215	160	160	163
1.88E-07	255	265	262	249	240	243	230	225	223	225	216	220	261	246	249	214	211	214	162	162	162
9.38E-08	247	248	254	241	238	236	215	223	217	219	219	216	256	249	249	210	210	221	161	162	161
4.69E-08	245	254	244	240	237	241	218	218	218	219	218	212	248	249	246	212	202	203	163	162	161
2.34E-08	240	246	243	244	240	241	222	219	222	225	216	216	246	249	247	210	210	227	163	162	162
1.17E-08	244	247	244	237	235	239	220	218	218	225	221	214	243	243	244	210	206	209	159	158	158
5.86E-09	239	244	241	240	240	241	213	221	221	218	215	219	245	235	238	223	225	224	161	163	162
2.93E-09	243	242	244	239	238	244	218	217	218	226	216	218	245	237	240	219	216	218	163	161	162
1.46E-09	240	240	246	241	241	240	216	217	218	224	216	221	251	250	247	218	215	217	165	163	161
7.32E-10	243	244	240	238	239	237	214	214	214	221	216	217	248	241	243	220	215	222	161	162	162

Molar	15			10			16			17		
0.000024	396	396	403	425	420	387	383	385	387	367	354	360
0.000012	395	391	397	406	405	395	378	379	385	369	359	359
0.000006	377	379	381	385	385	368	365	372	373	360	344	349
0.000003	355	351	355	364	354	347	339	348	338	347	335	337
1.5E-06	313	312	309	333	335	327	318	319	324	331	328	334
7.5E-07	276	268	258	317	313	309	280	290	294	314	319	308
3.75E-07	247	245	242	299	297	298	262	263	267	285	293	302
1.88E-07	227	226	219	281	292	288	241	248	281	260	278	281
9.38E-08	214	220	211	272	273	279	235	238	271	236	268	270
4.69E-08	201	199	207	269	279	268	233	231	248	238	253	245
2.34E-08	203	201	206	264	271	267	230	232	259	232	243	253
1.17E-08	212	207	207	268	272	268	223	223	234	233	246	243
5.86E-09	212	212	205	263	268	265	231	227	232	228	240	243
2.93E-09	212	211	210	267	266	268	226	229	233	234	248	239
1.46E-09	210	213	205	264	264	271	224	226	231	237	243	249
7.32E-10	214	209	210	267	268	264	227	230	232	235	235	234

Molar	18			12			19			20		
0.000024	434	436	433	393	391	393	397	393	373	374	371	373
0.000012	412	414	417	380	375	376	388	392	352	371	368	372
0.000006	400	399	400	365	371	365	378	382	361	364	359	359
0.000003	348	346	340	346	338	341	367	363	343	344	334	331
1.5E-06	329	328	324	326	319	318	338	341	332	327	316	312
7.5E-07	290	289	296	305	290	288	304	300	309	289	287	291
3.75E-07	267	266	271	285	280	269	282	279	268	278	268	273
1.88E-07	252	247	254	261	246	249	260	262	265	262	253	264

9.38E-08	231	236	232	256	249	249	251	252	260	260	251	256
4.69E-08	221	221	222	248	249	246	248	248	252	258	255	257
2.34E-08	233	228	224	246	249	247	253	252	254	256	260	255
1.17E-08	219	219	239	243	243	244	251	248	251	256	256	255
5.86E-09	238	234	236	245	235	238	249	245	256	263	252	257
2.93E-09	240	233	239	245	237	240	245	243	257	254	253	255
1.46E-09	232	236	238	251	250	247	247	246	247	257	254	256

Table S19: Raw data from main text figure 4

Molar	5 Open			5 Clicked			6 Open			6 Clicked			Control		
4.75E-06	341	337	336	347	353	340	349	354	356	355	363	359	258	249	247
2.38E-06	313	307	307	353	361	354	328	322	325	370	372	368	249	249	249
1.19E-06	288	287	286	346	344	356	299	301	299	363	371	365	255	248	247
5.94E-07	266	276	275	353	352	347	283	276	278	362	357	364	248	249	247
2.97E-07	270	271	267	336	340	331	274	278	276	353	368	348	253	224	259
1.48E-07	267	259	259	338	339	331	266	264	265	354	347	350	249	248	250
7.42E-08	256	254	258	320	320	325	263	266	263	349	336	327	250	249	253
3.71E-08	257	256	255	308	317	311	259	259	266	327	323	315	245	240	249
1.86E-08	256	255	257	302	306	303	267	263	262	315	300	316	246	249	247
9.28E-09	259	254	252	296	292	284	261	260	259	303	298	304	246	249	248
4.64E-09	253	259	260	286	281	291	263	266	266	295	298	298	251	247	251
2.32E-09	261	257	259	286	289	299	267	268	264	293	297	292	250	248	253
1.16E-09	260	254	252	289	279	289	264	265	262	287	293	295	249	249	254
5.8E-10	255	254	255	274	278	286	259	264	267	292	290	279	244	246	255
2.9E-10	252	257	250	284	284	294	259	266	266	281	280	287	243	249	254

Molar	8-L0 vs. Streptavidin			8-L0 vs. Neutravidin			8-L0 vs. Avidin			7-L0 vs. Streptavidin			7-L0 vs. Neutravidin			7-L0 vs. Avidin		
9.5E-06	209	208	211	154	155	153	150	148	151	232	230		149	149	148	146	145	149
4.75E-06	205	206	204	151	151	150	151	151	150	229	225		147	148	146	146	147	149
2.38E-06	201	202	206	152	148	149	148	149	149	221	212		144	143	142	144	141	146
1.19E-06	198	207	199	150	147	147	148	150	148	216	207		141	143	144	145	142	145
5.94E-07	201	198	195	149	148	149	152	150	148	208	201		141	144	146	142	141	147
2.97E-07	194	194	194	151	147	149	149	150	150	197	188		144	143	145	146	143	146
1.48E-07	185	183	181	149	146	147	148	149	150	186	180		144	143	142	141	143	143
7.42E-08	173	172	169	149	148	148	148	148	151	173	172		146	143	147	146	144	145
3.71E-08	160	157	160	149	147	148	150	148	149	164	163		143	141	143	142	139	144
1.86E-08	153	153	157	151	149	150	150	147	152	160	159		142	143	146	145	142	142
9.28E-09	153	152	152	149	149	150	151	147	147	157	155		142	145	146	141	139	143
4.64E-09	152	152	150	149	150	149	148	147	149	152	153		140	141	144	145	141	144
2.32E-09	149	150	151	151	151	149	151	146	149	152	152		143	141	143	141	139	141
1.16E-09	150	151	151	149	149	149	151	148	148	152	151		142	142	146	143	140	143
5.8E-10	152	149	152	149	149	151	149	149	148	152	151		145	144	146	144	145	148
2.9E-10	151	151	149	149	150	152	149	147	150	148	149		144	144	147	144	143	146

Molar	7-L0			7-L1			7-L2			7-L3			7-L4			7-L5		
9.5E-06	214	222	226	165	170	173	175	184	182	184	180	176	167	171	171	162	168	165
4.75E-06	213	214	219	164	162	163	164	164	169	173	168	168	160	162	169	153	160	161
2.38E-06	206	208	210	160	163	163	158	159	161	160	166	157	156	159	161	153	154	156
1.19E-06	205	202	205	155	160	156	150	154	151	157	156	153	154	159	156	153	149	150
5.94E-07	196	198	200	157	155	158	148	148	153	155	151	151	154	155	157	151	152	154
2.97E-07	190	193	193	151	155	153	150	150	152	155	153	151	153	153	158	149	152	154
1.48E-07	180	178	184	151	155	151	145	149	148	146	149	147	151	153	155	150	151	151
7.42E-08	160	162	163	150	154	156	146	144	147	147	148	147	151	151	153	151	149	154
3.71E-08	150	150	154	147	149	151	143	147	145	148	148	146	149	150	152	146	146	147
1.86E-08	148	149	149	150	152	153	146	146	149	150	149	144	149	152	151	147	148	150
9.28E-09	145	146	148	148	153	151	144	148	147	148	147	140	152	153	153	147	148	149
4.64E-09	149	147	149	151	151	150	146	145	145	148	146	148	149	151	149	149	151	149
2.32E-09	145	146	146	149	153	154	147	147	147	146	148	146	150	151	152	149	147	151
1.16E-09	147	148	148	152	154	153	147	144	147	144	147	148	151	151	155	149	150	151
5.8E-10	147	145	149	151	150	152	148	146	147	146	145	147	155	150	152	148	151	148
2.9E-10	148	146	149	146	150	153	147	146	148	147	144	146	152	149	153	150	151	147

7.2. Inhibition Assays

7.2.1. Thrombin

Protocol

Protocol was adapted from Habeshian et al. [12]. A 16-point dilution series of inhibitor was performed starting at 33 µM in Tris buffer (100 mM Tris-Cl, 150 mM NaCl, 10 mM $MgCl_2$, 1 mM $CaCl_2$, 2% DMSO). 9 µL from each dilution point was mixed with 3µL thrombin solution (9 nM, in Tris buffer) and 3 µL Z-Gly-Gly-Arg-AMC solution (225 µM, in Tris buffer) in black 384-well plates. Fluorescence (excitation at 360 nm, emission at 465 nm) was measured every 3 min for 30 min. The slopes were normalized & fitted, in order to calculate IC_{50} using Prism 10. Data was measured in triplicate.

Table S20: Raw data from main text figure 3 (normalized to 0-100)

Molar	4-L0			4-L1			4-L2		
0.00002	0	9.219858	3.76773	10	0.833333	3.466667	2.47	7	5.57
0.00001	12.36702	4.698582	4.609929	6.2	0	4.533333	1.53	0	3.27
0.000005	35.50532	38.07624	21.98582	7.566667	7.066667	4.3	6.8	5.83	6.7
2.5E-06	62.6773	93.88298	63.65248	8.5	4.866667	6.4	8.07	8.3	12.07
1.25E-06	73.00532	85.10638	74.64539	14.2	11.96667	19.73333	22.37	15.57	19.97
6.25E-07	74.42376	80.80674	94.41489	20.76667	27.56667	15.66667	32.87	30.93	42.23
3.13E-07	81.02837	76.46277	94.7695	44.1	55	47.6	57.77	50.83	57.5
1.56E-07	84.7961	76.24113	82.53546	64.06667	62.6	55.76667	66.53	71.97	57.6
7.81E-08	82.53546	93.61702	74.51241	73.56667	77.6	76.7	83.7	66.9	43.47
3.91E-08	84.35284	82.18085	79.16667	78.23333	79.56667	64.63333	85.9	83.63	53.5
1.95E-08	85.90426	92.64184	99.1578	78.36667	84.23333	78.93333	83.27	88.07	76.47
9.77E-09	87.6773	98.49291	88.82979	79.1	68.6	85.26667	87.07	80.4	66.33
4.88E-09	96.09929	80.93972	93.48404	87.23333	78.6	75.8	93.97	78.37	66.53
2.44E-09	79.92021	95.70035	97.78369	85.26667	79.46667	85.46667	90.6	85.5	76.03
1.22E-09	99.86702	86.17021	97.20745	100	81.76667	82.73333	100	77.43	73.8
6.1E-10	100	83.33333	98.71454	98.36667	93.23333	94.56667	97.8	85.2	86.43

Molar	4-L3			4-L4			4-L5			Control (Buffer)		
0.00002	3.966667	0	2.8	9.033333	0	2.533333	1.930164	0	0.737085	87.1789	83.87615	81.5367
0.00001	6.7	8.066667	5.6	5.733333	10.2	5.6	6.052845	3.991505	2.092573	71.76606	80.16055	73.55505
0.000005	7.133333	7.9	13.8	13	14.8	9.7	18.48335	23.48054	11.29989	88.41743	89.58716	84.63303
2.5E-06	27.06667	38.8	35.43333	40.63333	33.6	29.26667	23.7304	35.03654	22.4811	71.69725	73.89908	65.57339
1.25E-06	49.23333	49.83333	49.63333	60.66667	56.6	46.53333	57.96115	45.46817	71.39109	85.80275	82.22477	83.4633
6.25E-07	59.56667	55.7	63.53333	77.8	67.46667	62.8	66.45637	78.01237	54.58804	85.80275	73.48624	73.48624
3.13E-07	70.9	70.46667	75.7	92.23333	80.03333	98.63333	73.63983	100	64.0827	80.50459	100.3899	85.66514
1.56E-07	86.43333	84.13333	81	82.93333	77.5	81.33333	75.39447	73.88969	67.45581	73.62385	72.86697	78.50917
7.81E-08	81.9	90.13333	82.7	93.1	100	92.9	78.01237	82.01012	77.13786	86.00917	106.1697	107.8211
3.91E-08	93.13333	90.33333	84.03333	79.1	88.56667	88.1	92.82778	75.32638	83.70229	80.98624	81.46789	91.23853
1.95E-08	90.36667	100	82.1	77.53333	96.33333	91.4	77.70004	78.07483	79.76138	97.63761	87.11009	93.57798
9.77E-09	90.7	90.56667	94.96667	90.16667	74.73333	75.5	75.70117	85.39447	86.19527	89.65596	82.01835	83.53211
4.88E-09	95.3	94.2	79.93333	87.93333	79.2	86.76667	85.13336	95.13898	73.01518	97.01835	78.85321	92.8211
2.44E-09	75.4	89.7	77.56667	68.26667	77.3	97.9	80.01124	84.82104	86.25773	85.87156	87.52294	84.42661
1.22E-09	94.1	94.23333	79.86667	93.86667	93.56667	96.13333	86.63252	93.01518	90.00562	83.32569	75.55046	93.85321
6.1E-10	91.96667	94.5	82.53333	90.96667	83.83333	95.56667	88.38154	93.0664	83.94653	81.94954	88.69266	96.46789

7.2.2. PLAP

Protocol

500 µM of inhibitor was used as a starting point for a 16-point serial dilution in 10% DMSO/activation buffer in PCR tubes. Separately a 12.5 nM solution of PLAP and a 4.5 mM solution of para-Nitrophenylphosphate (pNPP) were prepared in activation buffer. 8 µL of each inhibitor dilution was mixed with 16 µL of enzyme solution and 16 µL of pNPP solution

and gently mixed. The assay was allowed to develop in the dark for 30 min (activation buffer pH 10) or 1 hour (activation buffer pH 9). 50 µL of 2M NaOH was then added to quench the reaction. Readout was performed to detect 405 nm vs 620 nm absorption. Data was fitted using Prism 10.

Activation buffer pH 10: 150 mM diethanolamine, 1 mM $MgCl_2$, 60 mM $ZnCl_2$ in mQ, pH 9.8

Activation buffer pH 9: 150 mM Tris, 1 mM $MgCl_2$, 60 mM $ZnCl_2$ in mQ, pH 9.0

Table S21: Raw data from main text figure 5

Molar	21			22			Control (Buffer)		
0.000094	2.160448	1.8882	1.7352	1.308566	1.412264	1.410981	10.1229	15.88783	12.78408
0.000047	2.053407	1.83402	1.7784	1.190431	1.356464	1.280874	9.508104	12.40108	10.44102
2.35E-05	2.181977	2.2113	1.9125	1.345884	1.294212	1.326716	10.5104	14.49151	10.13774
1.18E-05	2.528193	2.84445	2.28195	1.3961	1.473568	1.735486	10.92738	12.94216	9.645317
5.88E-06	3.371676	4.2435	3.65391	1.681483	1.861429	2.093343	10.7156	13.37392	10.93694
2.94E-06	5.854464	6.340168	5.60178	4.625252	3.0616	4.158456	11.66364	15.45775	10.12572
1.47E-06	9.641026	9.68014	8.5113	8.708464	6.984405	6.016603	11.73036	14.14994	11.49372
7.34E-07	12.61068	11.6154	11.4417	11.09918	8.475036	9.31695	12.12883	14.35842	11.08245
3.67E-07	10.20285	12.25529	10.6056	9.669974	9.319807	12.84496	10.32531	13.669	10.25505
1.84E-07	10.49401	10.5579	11.1006	12.37951	9.350328	9.015625	10.43914	11.69634	11.81269
9.18E-08	11.10922	12.1275	11.241	10.77756	11.83205	13.93002	11.97655	14.83844	13.02176
4.59E-08	11.01839	12.1374	12.4101	11.38569	12.93865	13.98826	12.86965	12.5491	12.50711
2.29E-08	11.97471	8.18055	12.8052	13.06459	11.71577	10.38361	12.58264	13.20697	12.31791
1.15E-08	12.27474	13.5081	12.8052	13.31693	11.18338	13.62262	12.08655	13.17061	11.77494
5.74E-09	11.92789	11.898	11.7099	13.76168	11.99613	12.82038	11.75358	11.69505	9.757454
2.87E-09	10.70161	11.8449	11.43	13.33578	12.26459	14.52854	10.96198	14.02257	12.20126

Inhibition Experiment performed at pH 10

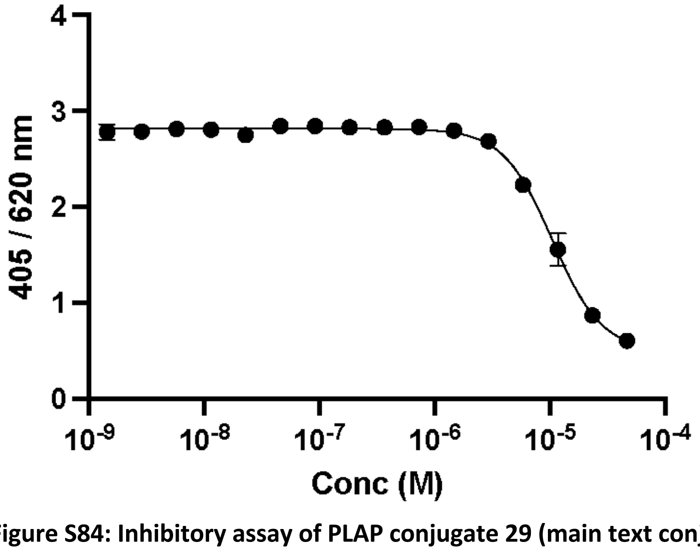

Figure S84: Inhibitory assay of PLAP conjugate 29 (main text conjugate 21) at pH 10

Determined fit: IC_{50} = 21 µM

0.000094	0.617	0.612	0.593
0.000047	0.856	0.909	0.853
2.35E-05	1.748	1.485	1.439
1.18E-05	2.288	2.24	2.167
5.88E-06	2.683	2.721	2.666
2.94E-06	2.753	2.841	2.804
1.47E-06	2.793	2.861	2.855

7.34E-07	2.785	2.861	2.847
3.67E-07	2.795	2.837	2.867
1.84E-07	2.794	2.88	2.858
9.18E-08	2.806	2.863	2.865
4.59E-08	2.7	2.736	2.816
2.29E-08	2.73	2.841	2.857
1.15E-08	2.764	2.833	2.844
5.74E-09	2.723	2.8	2.844
2.87E-09	2.732	2.743	2.876

8. Unprocessed Gel Images

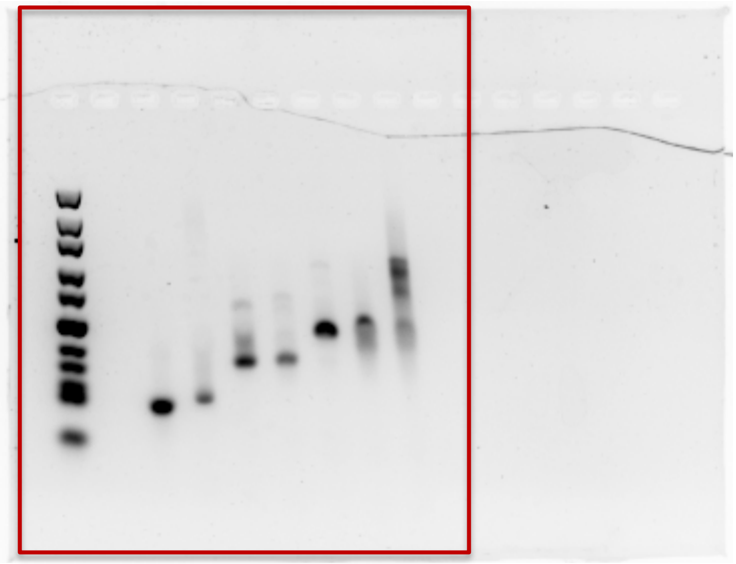

Figure S85: Unprocessed agarose gel electrophoresis of figure S27 panel A

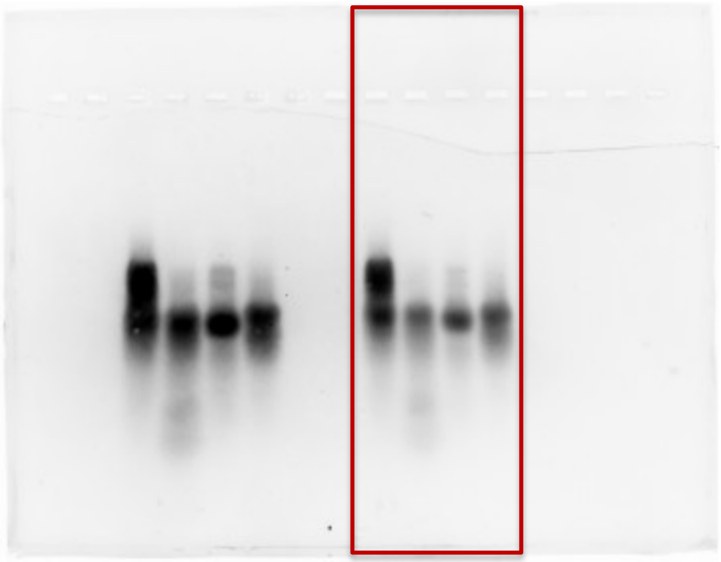

Figure S86: Unprocessed agarose gel electrophoresis of figure S27 panel B-1

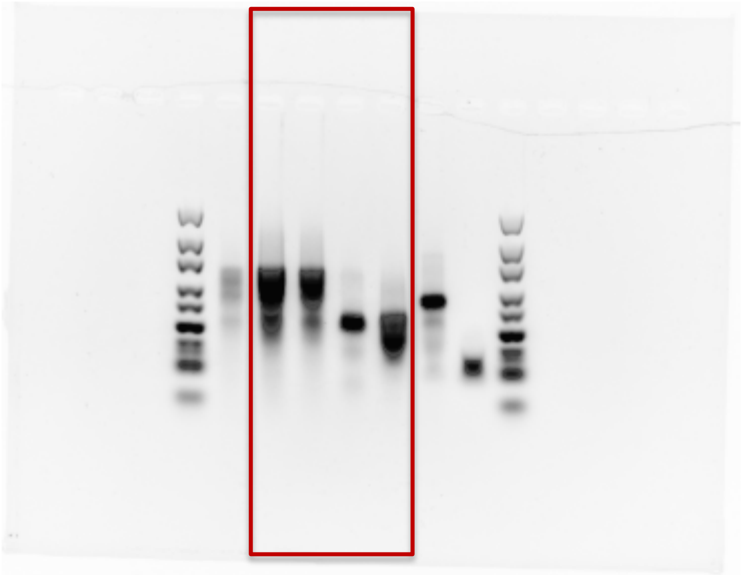

Figure S87: Unprocessed agarose gel electrophoresis of figure S27 panel B-2

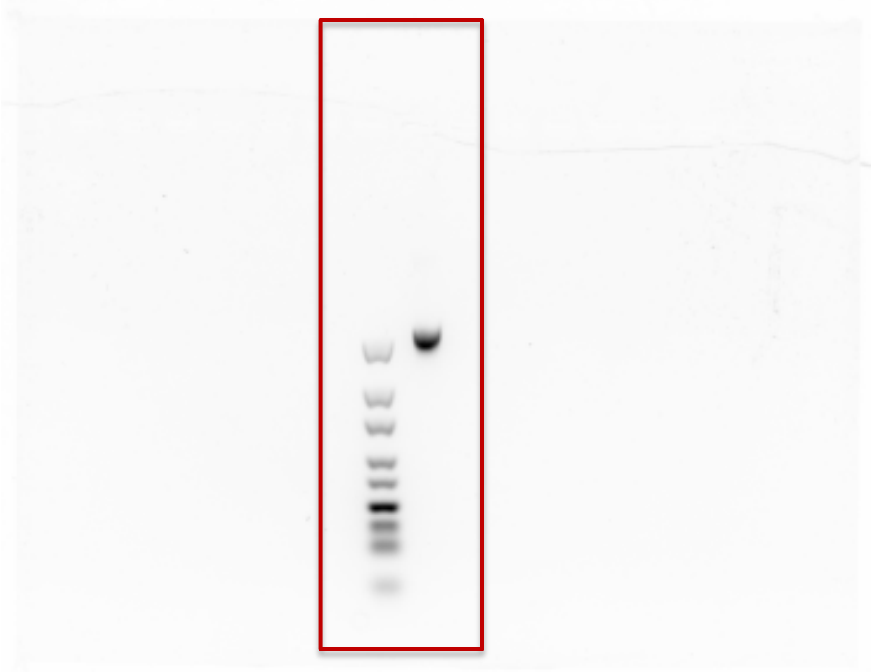

Figure S88: Unprocessed agarose gel electrophoresis of figure S31

References

1 *Alkaline Agarose Gel Electrophoresis*, <https://tools.thermofisher.com/content/sfs/manuals/MAN0011923_Alkaline_Agarose_Gel_Electrophoresis_UG.pdf> (2012).

2 Monty, O. B. C., Simmons, N., Chamakuri, S., Matzuk, M. M. & Young, D. W. Solution-Phase Fmoc-Based Peptide Synthesis for DNA-Encoded Chemical Libraries: Reaction Conditions, Protecting Group Strategies, and Pitfalls. *ACS Comb Sci* **22**, 833-843, doi:10.1021/acscombsci.0c00144 (2020).

3 Puglioli, S. *et al.* Selective tumor targeting enabled by picomolar fibroblast activation protein inhibitors isolated from a DNA-encoded affinity maturation library. *Chem*, doi:https://doi.org/10.1016/j.chempr.2022.10.006 (2022).

4 Pham, M. V., Bergeron-Brlek, M. & Heinis, C. Synthesis of DNA-Encoded Disulfide- and Thioether-Cyclized Peptides. *ChemBioChem* **21**, 543-549, doi:https://doi.org/10.1002/cbic.201900390 (2020).

5 Plais, L. *et al.* Universal encoding of next generation DNA-encoded chemical libraries. *Chem Sci* **13**, 967-974, doi:10.1039/d1sc05721a (2022).

6 Karas, J. A. *et al.* 2-Nitroveratryl as a Photocleavable Thiol-Protecting Group for Directed Disulfide Bond Formation in the Chemical Synthesis of Insulin. *Chemistry – A European Journal* **20**, 9549-9552, doi:https://doi.org/10.1002/chem.201403574 (2014).

7 Keller, M. *et al.* Highly pure DNA-encoded chemical libraries by dual-linker solid-phase synthesis. *Science* **384**, 1259-1265, doi:doi:10.1126/science.adn3412 (2024).

8 Binette, R., Desgagné, M., Theaud, C. & Boudreault, P.-L. Efficient Fmoc-Protected Amino Ester Hydrolysis Using Green Calcium(II) Iodide as a Protective Agent. *Molecules* **27**, 2788 (2022).

9 Decurtins, W. *et al.* Automated Screening for Small Organic Ligands Using DNA-Encoded Chemical Libraries. *Nat. Protoc.* **11**, 764 (2016).

10 Ahlskog, J. K. *et al.* Human monoclonal antibodies targeting carbonic anhydrase IX for the molecular imaging of hypoxic regions in solid tumours. *Br J Cancer* **101**, 645-657, doi:10.1038/sj.bjc.6605200 (2009).

11 Onda, Y. *et al.* A DNA-encoded chemical library based on peptide macrocycles. *Chem. - Eur. J.* **27**, 7160 (2021).

12 Habeshian, S. *et al.* Synthesis and direct assay of large macrocycle diversities by combinatorial late-stage modification at picomole scale. *Nature Communications* **13**, doi:10.1038/s41467-022-31428-8 (2022).
