# Supplementary material for: Flexibility-tuning of dual-display DNA-encoded chemical libraries facilitates cyclic peptide ligand discovery
Source: Nat Commun. 2025 Apr 5;16:3273. doi: 10.1038/s41467-025-58507-w (PMC11972359; doi:10.1038/s41467-025-58507-w)
Supplement: Supplementary file 2 — Reporting Summary [file 41467_2025_58507_MOESM2_ESM.pdf]

nature portfolio

Corresponding author(s): Jörg Scheuermann

Last updated by author(s): Dec 12, 2024

Reporting Summary

Nature Portfolio wishes to improve the reproducibility of the work that we publish. This form provides structure for consistency and transparency in reporting. For further information on Nature Portfolio policies, see our Editorial Policies and the Editorial Policy Checklist.

Statistics

For all statistical analyses, confirm that the following items are present in the figure legend, table legend, main text, or Methods section.

n/a	Confirmed	
[ ]	[x]	The exact sample size (*n*) for each experimental group/condition, given as a discrete number and unit of measurement
[ ]	[x]	A statement on whether measurements were taken from distinct samples or whether the same sample was measured repeatedly
[x]	[ ]	The statistical test(s) used AND whether they are one- or two-sided<br>*Only common tests should be described solely by name; describe more complex techniques in the Methods section.*
[x]	[ ]	A description of all covariates tested
[x]	[ ]	A description of any assumptions or corrections, such as tests of normality and adjustment for multiple comparisons
[ ]	[x]	A full description of the statistical parameters including central tendency (e.g. means) or other basic estimates (e.g. regression coefficient) AND variation (e.g. standard deviation) or associated estimates of uncertainty (e.g. confidence intervals)
[x]	[ ]	For null hypothesis testing, the test statistic (e.g. *F*, *t*, *r*) with confidence intervals, effect sizes, degrees of freedom and *P* value noted<br>*Give P values as exact values whenever suitable.*
[x]	[ ]	For Bayesian analysis, information on the choice of priors and Markov chain Monte Carlo settings
[x]	[ ]	For hierarchical and complex designs, identification of the appropriate level for tests and full reporting of outcomes
[x]	[ ]	Estimates of effect sizes (e.g. Cohen's *d*, Pearson's *r*), indicating how they were calculated

Our web collection on statistics for biologists contains articles on many of the points above.

Software and code

Policy information about availability of computer code

Data collection	Fluorescence Polarization and inhibition assay data was collected on Spectra Max Paradigm multimode plate reader. The raw data collected from each measurement can be found in the supplementary information. DEL selections data was recorded using Novaseq illumina sequencing
Data analysis	Selection Fingerprint data was displayed and evaluated using MATLAB version R2023b (Mathworks) using a previously reported software (https://doi.org/10.1038/nprot.2016.039). Selection triplicate count data was concatenated using a Python script (given in the Supplementary Information). Fluorescence polarization and Inhibition Assay data was fitted and displayed using PRISM 10. NMR was evaluated using Mestrenova 14.2 software

For manuscripts utilizing custom algorithms or software that are central to the research but not yet described in published literature, software must be made available to editors and reviewers. We strongly encourage code deposition in a community repository (e.g. GitHub). See the Nature Portfolio guidelines for submitting code & software for further information.

Data

Policy information about availability of data

All manuscripts must include a data availability statement. This statement should provide the following information, where applicable:

- Accession codes, unique identifiers, or web links for publicly available datasets
- A description of any restrictions on data availability
- For clinical datasets or third party data, please ensure that the statement adheres to our policy

The main data related to the study is located in the article and associated supplementary information. Raw data relating to experimental affinity/inhibiton measurements is listed in section 7 of the supplementary information document. Information relating to encoding and building blocks included in the library is listed in section 4 of the supplementary information. Additional data can be made available upon additional request.

Research involving human participants, their data, or biological material

Policy information about studies with human participants or human data. See also policy information about sex, gender (identity/presentation), and sexual orientation and race, ethnicity and racism.

Reporting on sex and gender	No data from human participants was collected over the course of this study.
Reporting on race, ethnicity, or other socially relevant groupings	No data from human participants was collected over the course of this study.
Population characteristics	No data from human participants was collected over the course of this study.
Recruitment	No data from human participants was collected over the course of this study.
Ethics oversight	No data from human participants was collected over the course of this study.

Note that full information on the approval of the study protocol must also be provided in the manuscript.

Field-specific reporting

Please select the one below that is the best fit for your research. If you are not sure, read the appropriate sections before making your selection.

☒ Life sciences ☐ Behavioural & social sciences ☐ Ecological, evolutionary & environmental sciences

For a reference copy of the document with all sections, see nature.com/documents/nr-reporting-summary-flat.pdf

Life sciences study design

All studies must disclose on these points even when the disclosure is negative.

Sample size	All DNA encoded library selections were performed in triplicate. Hit validation was performed in either duplicate or triplicate experiments.
Data exclusions	No data exclusion
Replication	Selections were performed in triplicate. Inhibition experiments were performed in triplicate. Fluorescence polarization experiments were performed in duplicate or triplicate.
Randomization	not relevant
Blinding	not relevant

Reporting for specific materials, systems and methods

We require information from authors about some types of materials, experimental systems and methods used in many studies. Here, indicate whether each material, system or method listed is relevant to your study. If you are not sure if a list item applies to your research, read the appropriate section before selecting a response.

Materials & experimental systems

n/a	Involved in the study
☒	☐ Antibodies
☐	☒ Eukaryotic cell lines
☒	☐ Palaeontology and archaeology
☒	☐ Animals and other organisms
☒	☐ Clinical data
☒	☐ Dual use research of concern
☒	☐ Plants

Methods

n/a	Involved in the study
☒	☐ ChIP-seq
☒	☐ Flow cytometry
☒	☐ MRI-based neuroimaging

Eukaryotic cell lines

Policy information about cell lines and Sex and Gender in Research

Cell line source(s)	Chinbese hamster overy (CHO) cells of our lab (purchased from Invitrogen/Thermo Fisher) were use for the production of PLAP protein (see Supplementary Information).
Authentication	none
Mycoplasma contamination	not tested
Commonly misidentified lines (See ICLAC register)	*Name any commonly misidentified cell lines used in the study and provide a rationale for their use.*

Plants

Seed stocks	not relevant
Novel plant genotypes	not relevant
Authentication	not relevant
